# Supplementary material for: Identification of novel breast cancer susceptibility loci in meta-analyses conducted among Asian and European descendants
Source: Nat Commun. 2020 Mar 5;11:1217. doi: 10.1038/s41467-020-15046-w (PMC7057957; doi:10.1038/s41467-020-15046-w)
Supplement: Supplementary file 1 — Supplementary Information [file 41467_2020_15046_MOESM1_ESM.pdf]

Supplementary Table 1. Studies contributing to the current analysis

Study	Cases	Controls	Sub-total	Genotyping platform
**Asian ancestry (discovery)**				
SBCGS				
	2,511	2,135	4,646	Affymetrix GenomeWide Human SNP Array 6.0
	1,563	2,396	3,959	Illumina HumanExome-12v1_A Beadchip
	1,794	2,059	3,853	Illumina Multi-Ethnic Genotyping Array
HCES-Br	274	273	547	Illumina Multi-Ethnic Genotyping Array
KPOP	963	921	1,884	Illumina Multi-Ethnic Genotyping Array
BBJ1	2,642	2,099	4,741	Illumina OmniExpress BeadChip
SeBCS	2,246	2,052	4,298	Affymetrix Genome-Wide Human SNP Array 6.0
BCAC iCOGS	4,759	5,957	10,716	Illumina iSelect Genotyping Array
BCAC Oncoarray	7,454	6,883	14,337	Illumina Infinium OncoArray-500K BeadChip
**Sub-total**	**24,206**	**24,775**	**48,981**	
**European ancestry (discovery)**				
BCAC GWAS	14,910	17,588	32,498	Illumina 370K/550K/610K/670K/1.2M, Affymetrix 5.0/6.0
BCAC iCOGS	46,785	42,892	89,677	Illumina iSelect Genotyping Array
BCAC Oncoarray	61,282	45,494	106,776	Illumina Infinium OncoArray-500K BeadChip
**Sub-total**	**122,977**	**105,974**	**228,951**	
**Total (discovery)**	**147,183**	**130,749**	**277,932**	
**Asian ancestry (validation)**				
HCES1	3,387	3,172	6,559	iPLEX Sequenom MassArray
HCES2	2,187	621	2,808	iPLEX Sequenom MassArray
Hong Kong	476	278	754	iPLEX Sequenom MassArray
KNCC	505	503	1,008	iPLEX Sequenom MassArray
KOHBRA	1,397	3,209	4,606	iPLEX Sequenom MassArray
SeBCS	775	1,103	1,878	iPLEX Sequenom MassArray
NGOBCS	400	401	801	iPLEX Sequenom MassArray
HERPACC-II	644	644	1,288	iPLEX Sequenom MassArray
TWBCS	1,058	1,065	2,123	iPLEX Sequenom MassArray
MYBRCA & SGBCC	5,958	5,684	11,642	Illumina Infinium OncoArray-500K BeadChip
**Sub-total**	**16,787**	**16,680**	**33,467**	
**Total (discovery+replication)**	**163,970**	**147,429**	**311,399**	

Supplementary Table 2. Pleiotropy of primary hits in the GWAS Catalog

SNP	SNP in LD	r^2	Effect allele	Other allele	Disease/trait	OR/Beta	P	Study	Journal
rs3790585	rs1707322	0.58 (East Asian); 0.30 (EA)	G	A	Body mass index	0.02	2×10^{-8}	Genome-wide physical activity interactions in adiposity - A meta-analysis of 200,452 adults.	PLoS Genet
	rs2275426	0.52 (East Asian); 0.12 (EA)	A	G	Body mass index	0.02	8×10^{-10}	The Influence of Age and Sex on Genetic Associations with Adult Body Size and Shape: A Large-Scale Genome-Wide Interaction Study.	PLoS Genet
	rs1538970	0.57 (East Asian); 0.43 (EA)	A	G	Platelet count	0.04	4×10^{-19}	The Allelic Landscape of Human Blood Cell Trait Variation and Links to Common Complex Disease.	Cell
	rs2230657	0.69 (East Asian); 0.15 (EA)	A	G	Hemoglobin concentration	0.03	3×10^{-13}	The Allelic Landscape of Human Blood Cell Trait Variation and Links to Common Complex Disease.	Cell
	rs2991971	0.79 (East Asian); 0.13 (EA)	G	C	High light scatter reticulocyte count	0.03	4×10^{-12}	The Allelic Landscape of Human Blood Cell Trait Variation and Links to Common Complex Disease.	Cell
	rs61784824	0.76 (East Asian); 0.36 (EA)	Not Reported (NR)	NR	Platelet count	0.03	2×10^{-10}	Genetic analysis of quantitative traits in the Japanese population links cell types to complex human diseases.	Nat Genet
	rs7538978	0.58 (East Asian); 0.31 (EA)			Sodium levels	0.03	5×10^{-12}	Genetic analysis of quantitative traits in the Japanese population links cell types to complex human diseases.	Nat Genet
rs2758598	rs2072499	0.99 (East Asian); 0.93 (EA)	G	A	Testicular germ cell tumor	1.18-1.20	3×10^{-8} - 2×10^{-10}	Meta-analysis of five genome-wide association studies identifies multiple new loci associated with testicular germ cell tumor.<br>Identification of 19 new risk loci and potential regulatory mechanisms influencing susceptibility to testicular germ cell tumor.<br>Identification of nine new susceptibility loci for testicular cancer, including variants near DAZL and PRDM14.	Nat Genet
	rs2984613	0.33 (East Asian); 0.91 (EA)	C	T	White matter hyperintensity burden	NR	2×10^{-8}	Multiethnic genome-wide association study of cerebral white matter hyperintensities on MRI.	Circ Cardiovasc Genet
	rs2984613	0.33 (East Asian); 0.91 (EA)	C	T	Intracerebral hemorrhage	1.33	2×10^{-10}	Meta-analysis of genome-wide association studies identifies 1q22 as a susceptibility locus for intracerebral hemorrhage.	Am J Hum Genet
rs6756513	-	-	A	G	Platelet count	0.03	7×10^{-10}	The Allelic Landscape of Human Blood Cell Trait Variation and Links to Common Complex Disease.	Cell
	rs3771529	0.40 (East Asian); 0.56 (EA)	A	G	Mean platelet volume	0.04	9×10^{-17}	The Allelic Landscape of Human Blood Cell Trait Variation and Links to Common Complex Disease.	Cell
	rs11686934	0.25 (East Asian); 0.40 (EA)	G	A	High light scatter reticulocyte count	0.02	7×10^{-9}	The Allelic Landscape of Human Blood Cell Trait Variation and Links to Common Complex Disease.	Cell
rs2901157	rs12570134	0.65 (East Asian); 0.63 (EA)	T	G	Double-edged eyelids	1.41	8×10^{-15}	Genome-wide association study in Japanese females identifies fifteen novel skin-related trait associations.	Sci Rep
rs855596	rs703556	0.43 (East Asian); 0.94 (EA)	A	G	Mammographic density (dense area)	0.41	4×10^{-10}	Genome-wide association study identifies multiple loci associated with both mammographic density and breast cancer risk.	Nat Commun
rs8027365	rs7184046	0.97 (East Asian); 0.90 (EA)	C	G	Height	0.03	2×10^{-10}	Meta-analysis of genome-wide association studies of adult height in East Asians identifies 17 novel loci.	Hum Mol Genet
rs34331122	rs1978060	0.61 (East Asian); 0.40 (EA)	G	A	Childhood ear infection, Myringotomy	1.09-1.17	3×10^{-10} - 1×10^{-19}	Genome-wide association and HLA region fine-mapping studies identify susceptibility loci for multiple common infections.	Nat Commun
			NR	NR	Childhood ear infection	1.09	1×10^{-19}	Detection and interpretation of shared genetic influences on 42 human traits.	Nat Genet
	rs2238776	0.43 (East Asian); <0.2 (EA)	G	A	Prostate cancer	1.08	2×10^{-8}	A meta-analysis of 87,040 individuals identifies 23 new susceptibility loci for prostate cancer.	Nat Genet
			A	G	Interleukin-10 levels	-0.06	4×10^{-9}	Genome-wide associated loci influencing interleukin (IL)-10, IL-1Ra, and IL-6 levels in African Americans.	Immunogenetics
	rs41298830	0.43 (East Asian); <0.2 (EA)	A	C	Tonsillectomy	1.06	5×10^{-8}	Genome-wide association and HLA region fine-mapping studies identify susceptibility loci for multiple common infections.	Nat Commun
			NR	NR				Detection and interpretation of shared genetic influences on 42 human traits.	Nat Genet

Supplementary Table 3. Results of replication study 28 newly-associated risk variants using additional Asian samples

	Sequenom replication									MYBRCA & SGBCC									Meta-analysis			
SNP	Test	Index	EAS, r2	MAF, case	MAF, control	N, case	N, control	OR	P	SNP	Test	MAF, case	MAF, control	N, case	N, control	Info score	OR	P	OR	P	P_{het}	Consistent with main results
rs4442172	A	rs142360995	1	0.069	0.067	9845	9612	1.03 (0.90-1.17)	0.683	rs142360995	A	0.113	0.124	5958	5684	0.995	1.03 (0.94-1.11)	0.550	1.03 (0.96-1.10)	0.469	0.981	Yes
rs2901157	G	NA	NA	0.323	0.320	10108	9558	1.00 (0.94-1.05)	0.931	rs2901157	G	0.193	0.186	5958	5684	1	0.96 (0.90-1.03)	0.271	0.98 (0.94-1.03)	0.442	0.428	Yes
rs75004998	G	NA	NA	0.442	0.420	9868	9731	1.08 (1.04-1.13)	2.95×10^{-4}	rs75004998	G	0.564	0.576	5958	5684	0.998	1.02 (0.97-1.07)	0.513	1.06 (1.02-1.09)	1.27×10^{-3}	0.076	Yes
rs73006998	A	NA	NA	0.338	0.362	10311	10271	0.90 (0.86-0.94)	8.98×10^{-7}	rs73006998	A	0.278	0.277	5958	5684	0.993	0.92 (0.86-0.98)	4.50×10^{-3}	0.90 (0.87-0.94)	1.66×10^{-8}	0.557	Yes
rs10838267	A	NA	NA	0.327	0.320	9944	9546	1.03 (0.99-1.08)	0.176	rs10838267	A	0.324	0.340	5958	5684	0.993	0.98 (0.93-1.04)	0.552	1.01 (0.98-1.05)	0.498	0.189	-
rs6756513	A	NA	NA	0.315	0.316	10112	9787	1.00 (0.95-1.04)	0.835	rs6756513	A	0.273	0.286	5958	5684	0.949	0.98 (0.92-1.04)	0.565	0.99 (0.96-1.03)	0.610	0.735	Yes
rs35418111	A	NA	NA	0.240	0.226	8959	8450	1.07 (1.01-1.13)	0.014	rs35418111	A	0.181	0.170	5958	5684	0.963	1.05 (0.98-1.12)	0.150	1.06 (1.02-1.11)	4.69×10^{-3}	0.753	Yes
rs13212889	A	rs6940159	0.8	0.130	0.120	10335	10217	1.11 (1.04-1.18)	1.48×10^{-3}	rs6940159	C	0.187	0.192	5958	5684	0.958	1.09 (1.01-1.16)	0.019	1.10 (1.05-1.15)	8.31×10^{-5}	0.718	Yes
rs9460685	G	rs7765429	1	0.082	0.077	10204	10215	1.03 (0.95-1.11)	0.470	rs7765429	C	0.179	0.200	5958	5684	0.997	1.03 (0.96-1.10)	0.400	1.03 (0.98-1.08)	0.268	0.975	Yes
rs1962901	G	rs2849506	1	0.472	0.479	9699	9581	0.97 (0.93-1.01)	0.184	rs2849506	C	0.484	0.484	5958	5684	0.987	0.98 (0.93-1.03)	0.436	0.97 (0.94-1.01)	0.127	0.834	Yes
rs7768862	A	NA	NA	0.281	0.296	10405	10324	0.93 (0.89-0.97)	9.45×10^{-4}	rs7768862	A	0.282	0.299	5958	5684	0.995	0.99 (0.93-1.04)	0.627	0.95 (0.92-0.98)	3.67×10^{-3}	0.098	Yes
rs3790585	T	NA	NA	0.274	0.283	10150	10036	0.94 (0.90-0.99)	0.011	rs3790585	T	0.346	0.353	5958	5684	1	0.94 (0.89-0.99)	0.032	0.94 (0.91-0.98)	8.68×10^{-4}	0.995	Yes
rs453974	C	rs144145984	0.9	0.394	0.393	10416	10366	0.99 (0.95-1.03)	0.621	rs144145984	CT	0.421	0.434	5958	5684	0.983	0.91 (0.85-0.96)	4.22×10^{-4}	0.96 (0.93-0.99)	0.010	0.013	Yes
rs6555134	T	NA	NA	0.261	0.269	9958	9718	0.96 (0.92-1.01)	0.130	rs6555134	T	0.256	0.285	5958	5684	0.971	0.99 (0.93-1.05)	0.829	0.97 (0.94-1.01)	0.180	0.460	Yes
rs11947923	T	NA	NA	0.282	0.288	10262	10031	0.98 (0.94-1.03)	0.443	rs11947923	T	0.302	0.300	5958	5684	0.904	1.03 (0.97-1.09)	0.299	1.00 (0.96-1.04)	0.990	0.197	-
rs1060604	C	rs2758598	1	0.149	0.147	10379	10318	1.03 (0.97-1.09)	0.392	rs2758598	A	0.179	0.188	5958	5684	0.996	1.03 (0.96-1.10)	0.395	1.03 (0.98-1.07)	0.229	0.919	Yes
rs855596	T	NA	NA	0.067	0.073	10358	10247	0.90 (0.83-0.98)	0.013	rs855596	T	0.071	0.081	5958	5684	0.975	0.88 (0.78-0.98)	0.014	0.89 (0.84-0.95)	5.22×10^{-4}	0.697	Yes
rs9316500	T	NA	NA	0.332	0.321	9290	8179	1.06 (1.01-1.11)	0.022	rs9316500	T	0.373	0.400	5958	5684	1	1.04 (0.98-1.09)	0.183	1.05 (1.01-1.09)	9.06×10^{-3}	0.623	Yes
rs11281251	DEL	NA	NA	0.187	0.188	10021	9993	0.99 (0.94-1.04)	0.717	rs11281251	DEL	0.175	0.193	5958	5684	0.994	0.99 (0.92-1.06)	0.765	1.01 (0.97-1.05)	0.737	0.550	-
rs34331122	DEL	NA	NA	0.470	0.463	10286	10164	1.03 (0.98-1.07)	0.233	rs34331122	DEL	0.454	0.443	5958	5684	0.94	1.04 (0.99-1.10)	0.146	1.03 (1.00-1.07)	0.068	0.657	Yes
rs78588049	DEL	NA	NA	0.142	0.149	10108	9558	0.92 (0.87-0.98)	7.11×10^{-3}	rs78588049	DEL	0.172	0.181	5958	5684	0.985	0.97 (0.91-1.04)	0.460	0.94 (0.90-0.99)	0.011	0.241	Yes
rs4322627	T	rs8027365	0.99	0.382	0.388	10108	9558	1.00 (0.94-1.06)	0.948	rs8027365	C	0.338	0.362	5958	5684	0.991	0.94 (0.89-0.99)	0.032	0.97 (0.93-1.01)	0.116	0.142	Yes

Supplementary Table 4. Conditional analysis to search for secondary signals for newly associated loci

SNP	Chr	BP	Test	Other	Adjusted SNP	EAF	OR (95% CI)	P
rs1911669	4	48065981	A	G	rs11944638	0.25	0.97 (0.96-0.98)	1.37×10^{-5}
rs1567217	4	48068783	A	G	rs11944638	0.25	0.97 (0.96-0.98)	1.29×10^{-5}
rs34900354	4	48072160	T	TC	rs11944638	0.25	0.97 (0.96-0.98)	1.44×10^{-5}
rs62301688	4	48073073	T	C	rs11944638	0.25	0.97 (0.96-0.98)	1.43×10^{-5}
rs2013231	4	48089458	A	T	rs11944638	0.75	1.03 (1.01-1.04)	4.96×10^{-5}
rs7693779	4	48186152	A	G	rs11944638	0.35	1.04 (1.02-1.05)	4.55×10^{-5}

The estimates for each individual study were obtained from conditional analysis with the additional adjustment of rs11944638 in the regression model or by GCTA -COJO.

Supplementary Table 5. Candidate genes identified by expression quantative loci anlaysis

					SBCS (N=151)					GTEx (N=85)			TCGA (N=672)			Metabric (N=1904)		
SNP	Gene	Gene location	Chr	BP	Test	Other	Beta	SE	P	Beta	SE	P	Beta	SE	P	Beta	SE	P
rs35418111	*YBEY*	Chr21:47706250-47717665	21	47856670	G	A	-0.33	0.12	9.6×10^{-3}	-0.84	0.21	1.0×10^{-4}	-0.27	0.10	5.2×10^{-3}	-0.2	0.02	3.7×10^{-20}
rs8027365	*SNUPN*	Chr15:75890423-75918810	15	75808740	A	C	-0.17	0.08	0.02	-0.27	0.11	0.015	-0.24	0.06	9.6×10^{-5}	-0.05	0.008	4.6×10^{-10}
rs8027365	*MAN2C1*	Chr15:75647547-75660971	15	75808740	A	C	-0.21	0.03	2.0×10^{-11}	-0.68	0.10	3.2×10^{-9}	-0.14	0.07	0.04	-0.13	0.008	4.2×10^{-58}
rs11281251	*LINC00886*	Chr3:156465134-156534851	3	156519412	T	TTGTGAC	0.47	0.10	1.0×10^{-5}	0.77	0.09	3.9×10^{-13}	NA	NA	NA	NA	NA	NA
rs34331122	*TBX1*	Chr22:19744225-19771116	22	19762428	C	CTT	-0.26	0.09	5.5×10^{-3}	0.16	0.12	0.175	-0.15	0.04	2.0×10^{-4}	-0.12	0.01	2.8×10^{-17}
rs144145984	*LOXL2*	Chr8:23154701-23282841	8	23644003	CT	C	0.16	0.05	4.3×10^{-3}	NA	NA	NA	0.01	0.06	0.817	0.01	0.004	0.013
rs144145984	*STC1*	Chr8:23699427-23712320	8	23644003	CT	C	0.14	0.09	0.11	NA	NA	NA	0.17	0.06	3.4×10^{-3}	0.36	0.03	4.1×10^{-25}
rs2758598	*SEMA4A*	Chr1:156117156-156147543	1	156194339	G	A	-0.03	0.10	0.73	-0.13	0.05	0.021	-0.06	0.05	0.254	-0.03	0.01	0.0497
rs3790585	*MUTYH*	Chr1:45794834-45806142	1	46023356	A	T	0.02	0.07	0.81	0.15	0.13	0.251	0.21	0.08	8.4×10^{-3}	0.08	0.01	6.5×10^{-16}

Supplementary Table 6. Transcriptome-wide association analysis for candidate genes identified by eQTL analysis

Gene	Location	Breast-tissue model								Cross-tissue model							
				ABCC			BCAC					ABCC			BCAC		
		R^2	No. of SNP $_{model}$	No. of SNP $_{used}$	Z	P	No. of SNP $_{used}$	Z	P	R^2	No. of SNP $_{model}$	No. of SNP $_{used}$	Z	P	No. of SNP $_{used}$	Z	P
LINC00886	Chr3:156465134-156534851	0.48	89	89	-0.54	0.59	88	-2.93	3.4×10^{-3}	0.52	109	105	-1.14	0.25	107	-4.07	4.7×10^{-5}
YBEY	Chr21:47706250-47717665	0.40	27	27	2.22	0.03	27	4.26	2.0×10^{-5}	0.77	88	87	1.72	0.09	87	3.69	2.2×10^{-4}
MAN2C1	Chr15:75647547-75660971	0.39	27	27	-3.34	8.5×10^{-4}	27	-5.32	1.0×10^{-7}	0.73	43	42	-3.05	2.3×10^{-3}	42	-4.99	6.0×10^{-7}
SEMA4A	Chr1:156117156-156147543	0.14	14	14	2.00	0.05	14	3.19	1.4×10^{-3}	0.27	76	76	2.23	0.03	76	5.49	4.1×10^{-8}
SNUPN	Chr15:75890423-75918810	0.03	4	4	0.01	0.99	4	-3.63	2.9×10^{-4}	0.41	38	37	-1.48	0.14	38	-4.49	7.1×10^{-6}
MUTYH	Chr1:45794834-45806142	0.04	12	12	-1.62	0.11	12	3.51	4.5×10^{-4}	0.14	34	32	2.91	3.6×10^{-3}	33	2.98	2.9×10^{-3}
STC1	Chr8:23699427-23712320	-	-	-	-	-	-	-	-	0.03	67	64	-0.26	0.80	65	-3.54	4.1×10^{-4}

No. of SNP $_{model}$: number of SNPs included in the prediction model.

No. of SNP $_{used}$: number of SNPs included in the association analysis.

Supplementary Table 7. Summary of association directions

SNP	Gene	Closest gene	Allele	BC risk	eQTL analysis	Gene-based analysis
rs35418111	*YBEY*	*PCNT, intron*	G	-	-	Oncogenic
rs8027365	*SNUPN*	*PTPN9, intron*	A	+	-	Tumor suppressing
rs8027365	*MAN2C1*	*PTPN9, intron*	A	+	-	Tumor suppressing
rs11281251	*LINC00886*	*LINC00886,intron*	T	-	+	Tumor suppressing
rs34331122	*TBX1*	*TBX1, intron*	C	+	-	NA
rs144145984	*LOXL2*	*LOC107986930, intergenic*	CT	-	+	NA
rs144145984	*STC1*	*LOC107986930, intergenic*	CT	-	+	Tumor suppressing
rs2758598	*SEMA4A*	*PMF1, intron*	G	-	-	Oncogenic
rs3790585	*MUTYH*	*AKR1A1, intron*	A	+	+	Oncogenic

Supplementary Table 8. Additional significant loci uncovered by cross-ancestry Meta-analysis

SNP	Chr	BP	Test	Other	Locus	Nearest Genes	Asian-specific Meta-analysis			European-specific Meta-analysis			Cross-ancestry Meta-analysis			I^2, %	$P_{heterogeneity}$
							AF	OR (95% CI)	P	AF	OR (95% CI)	P	AF	OR (95% CI)	P		
rs62134416	2	69389757	A	T	2p13.3	*ANTXR1*	0.77	1.03 (1.00-1.06)	0.086	0.44	1.03 (1.02-1.05)	1.39E-07	0.48	1.03 (1.02-1.04)	2.76E-08	0	0.793
rs3829964	6	36644498	T	C	6p21.2	*CDKN1A*	0.29	0.97 (0.94-1.00)	0.039	0.51	0.97 (0.96-0.98)	9.38E-08	0.48	0.97 (0.96-0.98)	1.05E-08	0	0.871
rs218872	8	23655784	T	G	8p21.2	*STC1*	0.25	1.04 (1.01-1.07)	0.016	0.41	1.03 (1.02-1.05)	8.93E-08	0.38	1.03 (1.02-1.05)	4.88E-09	0	0.824

Supplementary Table 9. Results of MR-MEGA for rs2758598 and rs142360995

SNP	Chr	BP	Test	Other	Locus	Meta-analysis			P heterogeneity
						AF	OR (95% CI)	P	
rs2758598	1	156194339	A	G	1q22				
Asian-Specific						0.16	1.07 (1.03-1.11)	1.8×10^{-4}	
European-specific						0.33	1.03 (1.02-1.05)	8.4×10^{-7}	
Cross-ancestry						0.31	1.04 (1.02-1.05)	3.6×10^{-9}	
MR-MEGA								9.9×10^{-10}	0.007
rs142360995	8	118205719	A	G	8q24.11				
Asian-Specific						0.09	1.13 (1.07-1.18)	4.1×10^{-6}	
European-specific						0.20	1.03 (1.02-1.05)	1.0×10^{-5}	
Cross-ancestry						0.19	1.04 (1.03-1.06)	3.0×10^{-8}	
MR-MEGA								8.1×10^{-9}	0.007

Supplementary Table 10. Evaluate the association of polygenic risk soc
with breast cancer risk in Shanghai Women's Health Study

	Asian-specific weights				
PRS	case/control	OR	L95	U95	P
Continous, 1 SD	-	1.44	1.28	1.61	4.62E-10
AUC: 0.601 (A); 0.602 (C)					
Decile 1	19/206	Ref.			
Decile 2	25/206	1.32	0.70	2.46	0.391
Decile 3	24/206	1.26	0.67	2.38	0.469
Decile 4	31/206	1.63	0.89	2.98	0.111
Decile 5	42/206	2.21	1.24	3.93	0.007
Decile 6	27/205	1.43	0.77	2.65	0.258
Decile 7	36/206	1.89	1.05	3.41	0.033
Decile 8	36/206	1.89	1.05	3.41	3.32E-02
Decile 9	59/206	3.11	1.79	5.39	5.69E-05
Decile 10	69/206	3.63	2.11	6.25	3.26E-06
AUC: 0.610 (A); 0.611 (C)					

AUC (A) or (C): Area under the curve, estimated using PRS weighted b
effect estimates from Asian-specific or cross-ethnic meta-analysis.

Supplementary Table 11. Associations between the 28 novel risk SNPs and breast cancer risk with adjustment of 10 PCs

SNP	EAF	OR (95% CI), 2 PCs	OR (95% CI), 10 PCs
rs72906468	0.76	1.04 (1.03-1.05)	1.04 (1.02-1.05)
rs3790585	0.81	1.04 (1.03-1.06)	1.04 (1.03-1.06)
rs2758598	0.31	1.04 (1.02-1.05)	1.04 (1.02-1.05)
rs6756513	0.29	0.96 (0.95-0.98)	0.97 (0.95-0.98)
rs73006998	0.22	0.93 (0.90-0.95)	0.92 (0.90-0.95)
rs11281251	0.37	0.97 (0.95-0.98)	0.97 (0.95-0.98)
rs11944638	0.85	1.06 (1.04-1.08)	1.06 (1.04-1.08)
rs11947923	0.36	0.97 (0.96-0.98)	0.97 (0.96-0.98)
rs6555134	0.54	0.97 (0.95-0.98)	0.97 (0.95-0.98)
rs7765429	0.49	0.97 (0.96-0.98)	0.97 (0.96-0.98)
rs7768862	0.48	0.97 (0.96-0.98)	0.97 (0.96-0.98)
rs6940159	0.43	0.96 (0.95-0.98)	0.97 (0.96-0.98)
rs144145984	0.55	0.97 (0.96-0.98)	0.97 (0.96-0.98)
rs2849506	0.41	0.97 (0.96-0.98)	0.97 (0.96-0.98)
rs142360995	0.19	1.04 (1.03-1.06)	1.04 (1.03-1.06)
rs10820600	0.48	0.97 (0.96-0.98)	0.97 (0.96-0.98)
rs541079479	0.39	1.03 (1.02-1.05)	1.03 (1.02-1.05)
rs2901157	0.85	1.05 (1.03-1.07)	1.05 (1.03-1.07)
rs10838267	0.51	1.04 (1.03-1.05)	1.04 (1.02-1.05)
rs78588049	0.19	0.96 (0.95-0.97)	0.96 (0.95-0.97)
rs855596	0.04	0.91 (0.89-0.94)	0.91 (0.89-0.94)
rs9316500	0.64	1.03 (1.02-1.05)	1.03 (1.02-1.05)
rs75004998	0.36	0.97 (0.96-0.98)	0.97 (0.96-0.98)
rs8027365	0.71	1.04 (1.03-1.05)	1.04 (1.03-1.05)
rs76535198	0.83	1.05 (1.04-1.07)	1.05 (1.03-1.07)
rs12481286	0.26	1.04 (1.03-1.06)	1.04 (1.03-1.06)
rs35418111	0.12	1.07 (1.05-1.09)	1.07 (1.05-1.09)
rs34331122	0.47	0.97 (0.96-0.98)	0.97 (0.96-0.98)

Supplementary Table 12. Statistical power for the association between SNP and breast cancer risk under varous senarios

	Effect allele frequency	Effect size (OR)	Power
Overall breast cancer, N cases= 147,183, N control= 130,749			
	0.05	1.08	0.815
	0.10	1.06	0.870
	0.20	1.05	0.969
	0.25	1.05	0.993
ER-positive breast cancer, N cases= 80,428, N control= 125,677			
	0.05	1.10	0.886
	0.10	1.07	0.841
	0.20	1.06	0.972
	0.25	1.06	0.994
ER-positive breast cancer, N cases= 26,948, N control= 125,677			
	0.05	1.15	0.888
	0.10	1.11	0.905
	0.20	1.08	0.870
	0.25	1.08	0.951

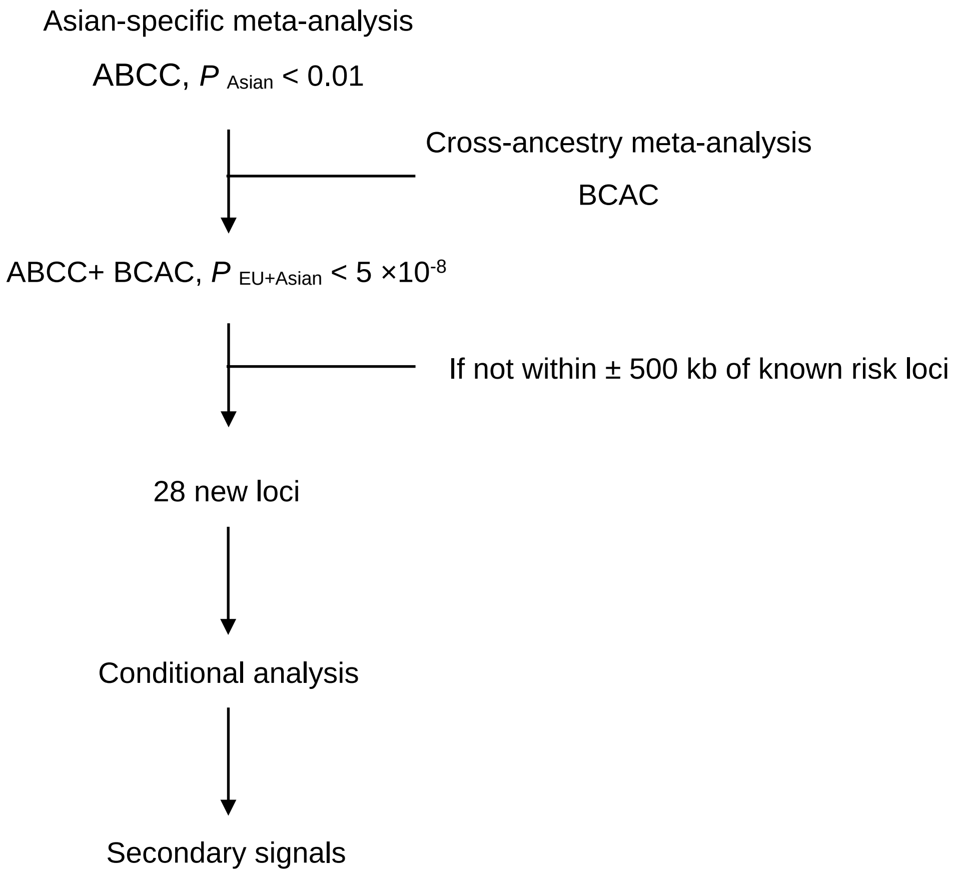

Supplementary Figure 1. Flowchart of identification of novel susceptibility loci for breast cancer.

Additional three novel loci were identified from the same procedures when loosen the $P_{Asian} <$ 0.1.

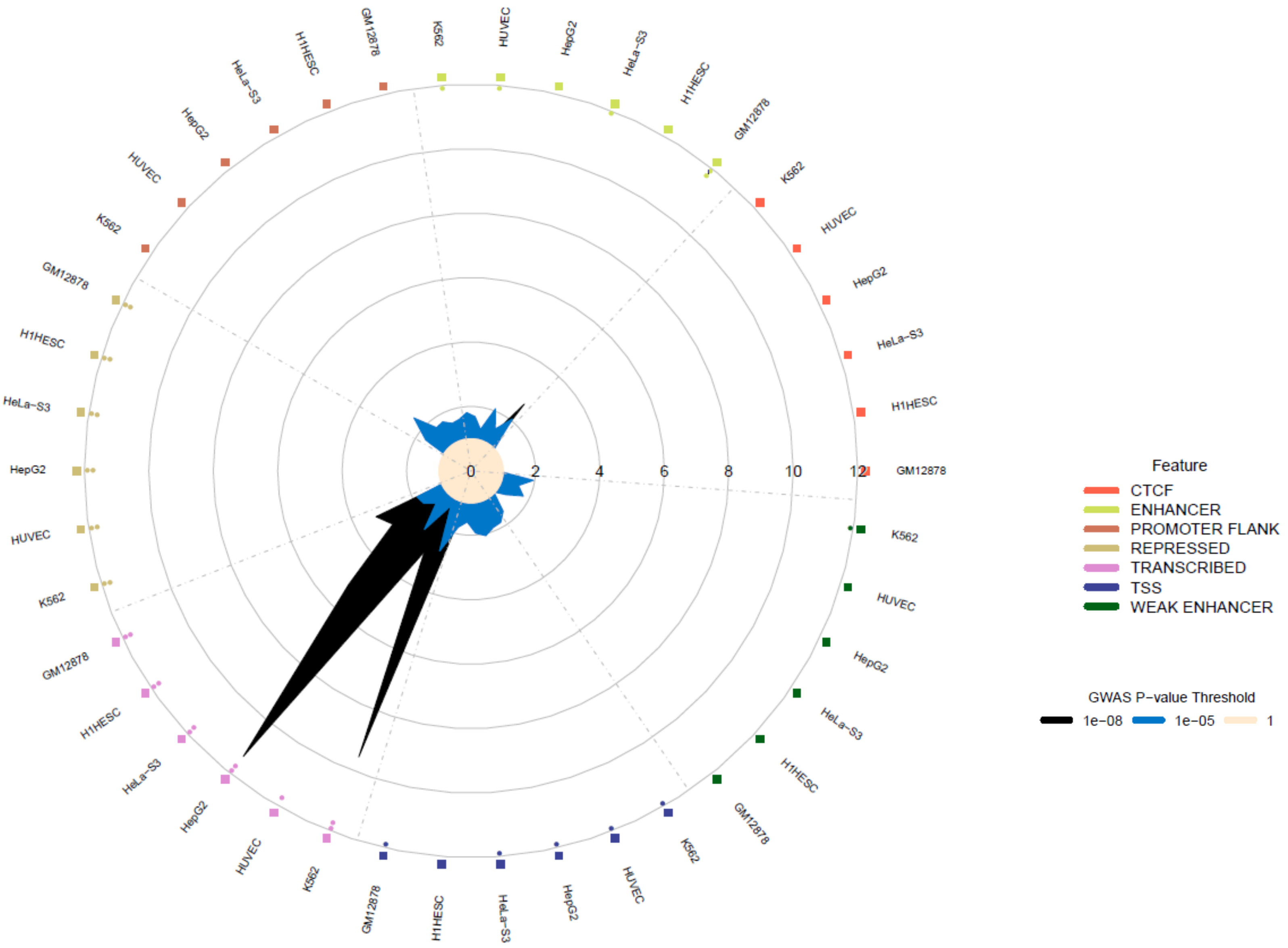

0
2
4
6
8
10
12
CTCF: GM12878, H1HESC, HeLa-S3, HepG2, HUVEC, K562
ENHANCER: GM12878, H1HESC, HeLa-S3, HepG2, HUVEC, K562
PROMOTER FLANK: GM12878, H1HESC, HeLa-S3, HepG2, HUVEC, K562
REPRESSED: GM12878, H1HESC, HeLa-S3, HepG2, HUVEC, K562
TRANSCRIBED: GM12878, H1HESC, HeLa-S3, HepG2, HUVEC, K562
TSS: GM12878, H1HESC, HeLa-S3, HepG2, HUVEC, K562
WEAK ENHANCER: GM12878, H1HESC, HeLa-S3, HepG2, HUVEC, K562
Feature
CTCF
ENHANCER
PROMOTER FLANK
REPRESSED
TRANSCRIBED
TSS
WEAK ENHANCER
GWAS P−value Threshold
1e−08
1e−05
1

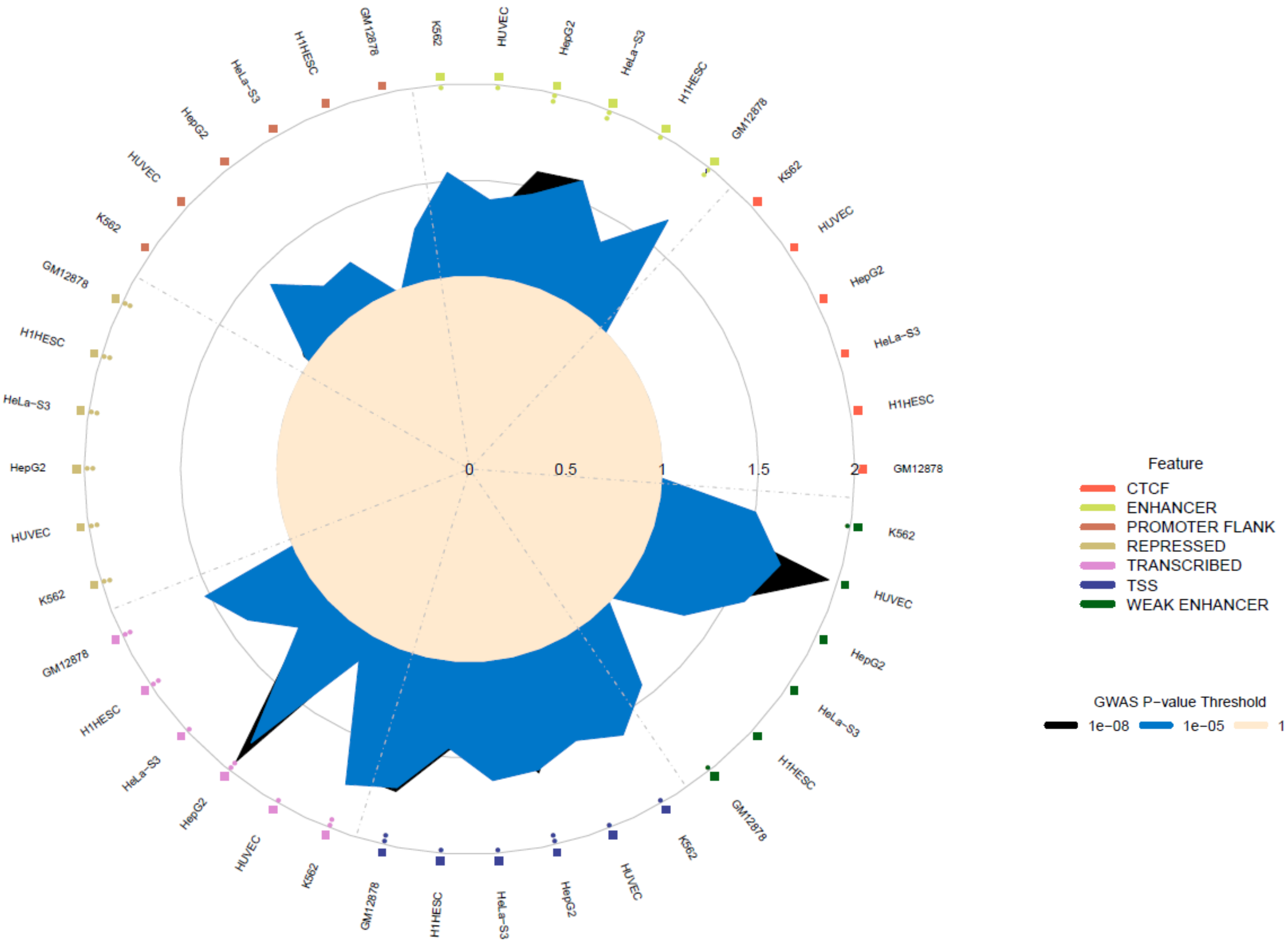
0
0.5
1
1.5
2
CTCF: GM12878, H1HESC, HeLa-S3, HepG2, HUVEC, K562
ENHANCER: GM12878, H1HESC, HeLa-S3, HepG2, HUVEC, K562
PROMOTER FLANK: GM12878, H1HESC, HeLa-S3, HepG2, HUVEC, K562
REPRESSED: GM12878, H1HESC, HeLa-S3, HepG2, HUVEC, K562
TRANSCRIBED: GM12878, H1HESC, HeLa-S3, HepG2, HUVEC, K562
TSS: GM12878, H1HESC, HeLa-S3, HepG2, HUVEC, K562
WEAK ENHANCER: GM12878, H1HESC, HeLa-S3, HepG2, HUVEC, K562
Feature
CTCF
ENHANCER
PROMOTER FLANK
REPRESSED
TRANSCRIBED
TSS
WEAK ENHANCER
GWAS P−value Threshold
1e−08
1e−05
1

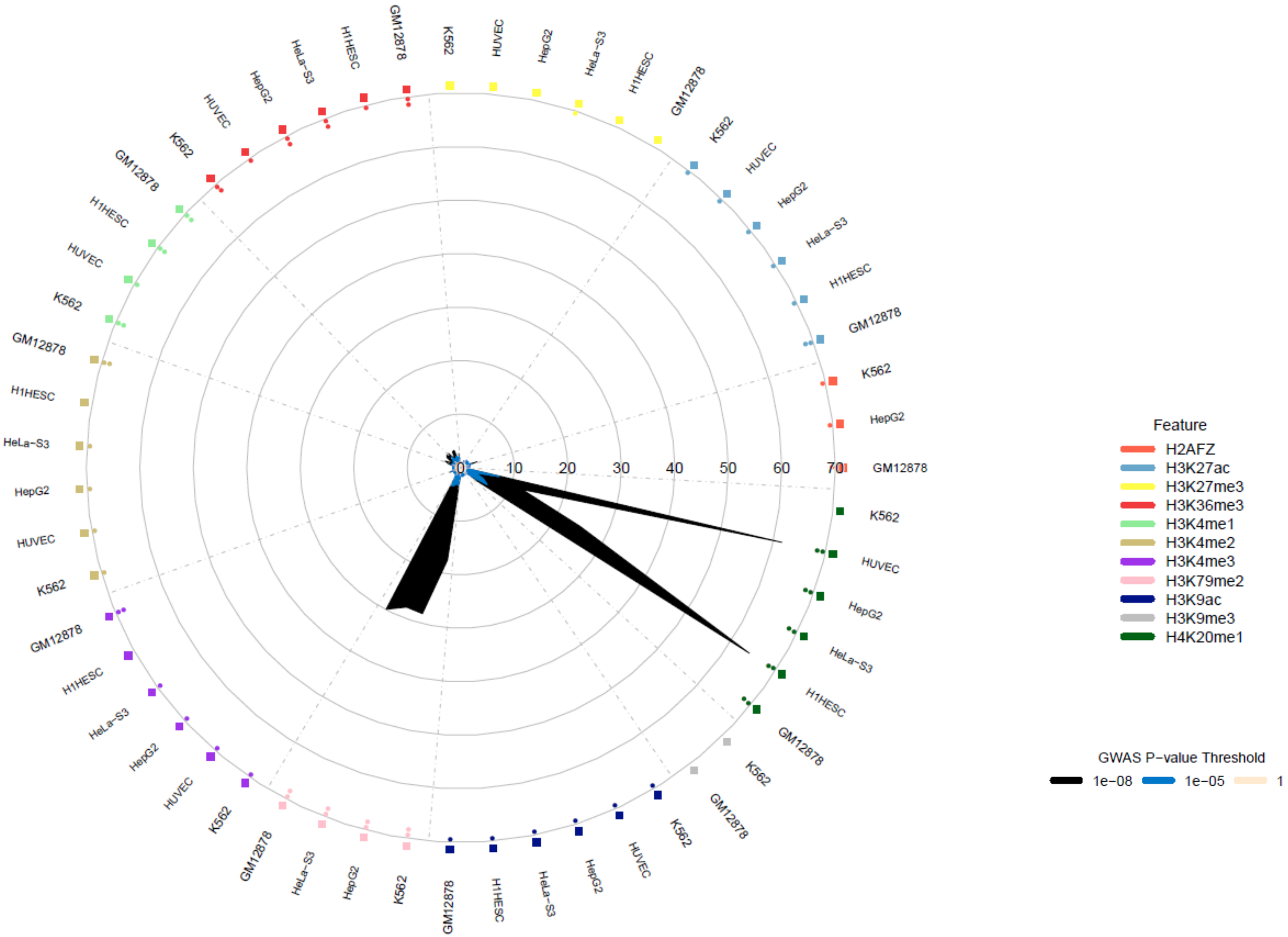

0
10
20
30
40
50
60
70
Feature
H2AFZ
H3K27ac
H3K27me3
H3K36me3
H3K4me1
H3K4me2
H3K4me3
H3K79me2
H3K9ac
H3K9me3
H4K20me1
GWAS P−value Threshold
1e−08
1e−05
1
GM12878
H1HESC
HeLa−S3
HepG2
HUVEC
K562

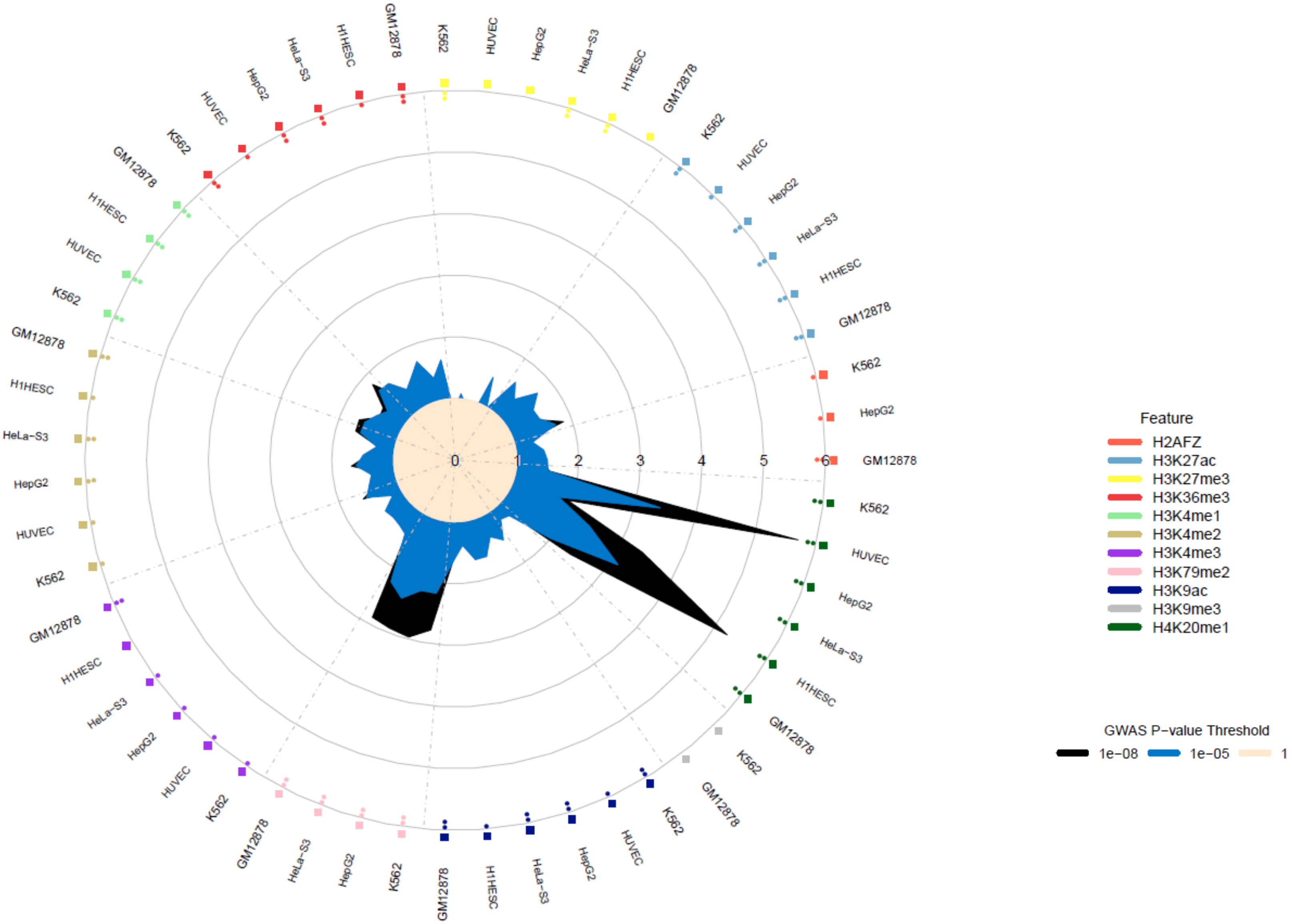

Feature
H2AFZ
H3K27ac
H3K27me3
H3K36me3
H3K4me1
H3K4me2
H3K4me3
H3K79me2
H3K9ac
H3K9me3
H4K20me1
GWAS P-value Threshold
1e-08
1e-05
1
0
1
2
3
4
5
6
GM12878
K562
HUVEC
HepG2
HeLa-S3
H1HESC

Supplementary Figure 2. Enrichment analysis of chromatin states and histone markers.

A. Enrichment analysis of chromatin states for newly identified association signals

B. Enrichment analysis of chromatin states for overall association signals

C. Enrichment analysis of histone markers for newly identified signals

D. Enrichment analysis of histone markers for overall association signals

Supplementary Note 1.

1. Description of Studies of the Asia Breast Cancer Consortium (ABCC)

Shanghai Breast Cancer Genetics Study (SBCGS)

The Chinese participants were drawn from Shanghai Breast Cancer Genetics Study (SBCGS), which consists of the **Shanghai Breast Cancer Study** (SBCS), **Shanghai Breast Cancer Survival Study** (SBCSS), **Shanghai Endometrial Cancer Study** (SECS, contributed control data only), and the **Shanghai Women's Health Study** (SWHS), four large population-based studies in urban Shanghai. All participants provided written informed consent prior to interview, and institutional review boards of all institutes in both China and the United States approved the study.

Shanghai Breast Cancer Study (SBCS):

The SBCS is a two-phase (SBCS-I and SBCS-II) population-based case-control study that recruited incident patients with breast cancer and controls in urban Shanghai, the largest commercial center in China.[1,2] In the initial phase (SBCS-I), subjects were recruited between August 1996 and March 1998. Through a rapid case-ascertainment system and the population-based Shanghai Cancer Registry, 1,602 eligible breast cancer cases diagnosed during the study period were identified, of which 1,459 cases (91.1%) completed in-person interviews. Two senior pathologists reviewed and confirmed cancer diagnoses for all patients. Controls were randomly selected from the general population using the Shanghai Resident Registry, a population registry containing demographic information for all residents of urban Shanghai. The inclusion criteria for controls were identical to those for cases, with the exception of a breast

cancer diagnosis. Among the 1,724 eligible controls, 1,556 (90.3%) completed in-person interviews. Our study used a structured questionnaire to elicit detailed information on demographic factors, and known/suspected risk factors for breast cancer. All participants were measured for their current weight, height, and circumference of the waist and hips. All interviews were tape-recorded and reviewed by the field supervisor and quality control staff to monitor the quality of interview data. For both cases and controls, blood samples (10 ml from each woman) were obtained who completed the in-person interview (1,193 (82%) cases and 1,310 (84%) controls). Using cotton swabs, a sample of exfoliated buccal cells was obtained from virtually all study participants who did not provide a blood sample. The second round of subject recruitment (SBCS-II) occurred between April 2002 and February 2005 using a protocol similar to the one used in the initial phase. Similar to the SBCS-I subjects, the majority of newly-recruited cases (n=1,932, 97.1%) and controls (n=1,857, 93.4%) provided a blood sample or an exfoliated buccal cell sample to the study. Our study used modified mouthwash method from initially reported by Lum A *et al.*,[3] and provided, on average, approximately 34 µg of DNA per sample. Eligibility criteria for study participation were identical for SBCS-I and SBCS-II except age. The age ranged from 25 to 65 years for SBCS-I, and from 25 to 70 years in SBCS-II.

Shanghai Breast Cancer Survival Study (SBCSS):

The SBCSS included newly diagnosed 6,303 breast cancer cases ascertained via the population-based Shanghai Cancer Registry between April 2002 and December 2006.[1] In-person interviews were conducted to collect information on known breast cancer risk factors as well as anthropometrics using a protocol and questionnaire similar to that

used in the SBCS. Patient medical charts were also reviewed to obtain detailed information on disease related characteristics and cancer treatment. Using the modified mouthwash method, buccal cell samples were collected from 96% of study participants. Total 1,469 breast cancer patients participated in both studies (SBCS-II and SBCSS) due to the time overlap in the participant recruitment period.

Shanghai Endometrial Cancer Study (SECS):

The SECS is a population-based, case-control study of endometrial cancer conducted between January 1997 and December 2003 using a protocol similar to the SBCS; only community controls from the SECS were included in the present study.[1] Except a few questionnaires related specifically to breast or endometrial cancer risk, the questionnaires used in the SECS and the SBCS were virtually identical. Eligible cases were identified through the population-based Shanghai Cancer Registry and controls were randomly selected from the general population of Shanghai using the Shanghai Resident Registry and were age frequency matched to cases. Women with a history of cancer or hysterectomy were not eligible. Trained interviewers conducted in-person interviews to collect detailed information on demographic factors, menstrual and reproductive history, hormone use, prior disease history, physical activity, tobacco and alcohol use, weight, and family history of cancer. Anthropometrics measurements were taken. A total of 1,039 controls provided a blood sample or buccal cell sample using the mouthwash method, and these women were included.

Shanghai Women's Health Study (SWHS):

The SWHS is a population-based cohort study of approximately 75,000 adult women who were recruited from urban Shanghai between 1997 and 2000.[4] Among those who

completed baseline survey through an in-person interview, 56,831 (75.8%) donated a blood sample, and 65,754 (87.7%) donated a urine sample. An exfoliated buccal cell sample was collected from an additional 8,934 (49.3%) of the 18,111 subjects who did not provide a blood sample at baseline. Therefore, we have genomic DNA from about 88% of cohort members. The cohort has been followed by a combination of record linkage and active follow-ups.[4] The first follow-up survey was conducted from 2000 to 2002. Approximately 99.8% of cohort members (or their next of kin, if subjects were deceased) were interviewed. The response rates were 98.7% for the second follow-up survey (2002-2004), 96.7% for the third follow-up survey (2004-2007), and approximately 93% for the fourth follow-up (2007-2010). For non-respondents, cancer diagnosis and vital status can still be identified through the linkage of data from cancer and vital statistics registries, and thus ascertainment for cancer outcomes and total mortality is virtually complete in this cohort. In the current study, breast cancer patients identified in the SWHS and non-cases were included.

Participants in SBCGS have been genotyped by Affymetrix Genome-Wide Human SNP Array 6.0,[1] the Asian ExomeChip,[5] and the Multi-Ethnic Global Array (MEGA), which includes approximately 2 million variants, including ~80k custom content for a large study including multiple complex traits. Similar genotyping and QC procedures have been described previously.[1,5] After imputation and QC exclusions, the final data set included 2,511 cases and 2,135 controls for 11.1 million markers for the Affy6 dataset, 1,563 cases and 2,396 controls for 2.95 million markers for the ExomeChip dataset, and 1,794 cases and 2,059 controls for 14.1 million markers for the MEGA dataset.

Hwasun Cancer Epidemiology Study-Breast (HCES-Br):

The Hwasun Cancer Epidemiology Study (HCES-Br) is a hospital-based case-control study whose goal is to identify factors of the cancer development and clinical progression in a Korean population.[6,7] Included in this project were 3,387 female breast cancer cases, who were newly diagnosed between April 2004 and February 2013 at Chonnam National University Hwasun Hospital, a cancer specified hospital in Jeollanam-do province, South Korea. Patients with secondary or recurrent tumor were excluded. Controls were 3,186 women who were randomly selected from among women with no previous cancer diagnosis at enrollment in the Namwon Study and the Dong-gu study, ongoing community-based cohort studies in South Korea.[8] Genomic DNA was extracted from their peripheral blood. Demographics data and conventional factors of breast cancer were collected by structured questionnaire and review of medical records. All cases and control subjects provided the informed consent to participate in the study and Institutional Review Board of Chonnam National University Hwasun Hospital approved this study. In the HCES-Br, 274 cases and 273 controls were genotyped by MEGA. This study also contributed 5,574 cases and 3,793 controls for the replication study genotyped by Sequenom.

Seoul Breast Cancer Study (SeBCS):

The SeBCS is a hospital-based case-control study conducted in two teaching hospitals in Seoul.[9,10] Total 2,342 incident breast patients histopathologically diagnosed with primary breast cancer were included in this project and they were consecutively recruited between 2001 and 2007. In-person interviews were conducted to collect information on known breast cancer risk factors and anthropometrics by using a protocol and questionnaire. Medical charts were reviewed to verify clinical information.

Eligible controls were derived from a large urban cohort that is participating in the Korea Genome Epidemiology Study (KoGES), which is an ongoing cohort study that has sought to understand the causes and risk factors of disease in South Korea. Total 2,052 controls were selected between May 2006 and December 2007. They were frequency-matched to cases on the case's age at diagnosis in five-year intervals. Trained interviewers using a structured questionnaire determined the demographic characteristics of the controls, their family histories with regard to breast cancer in first-degree relatives, reproductive and menstrual factors, and life-style habits, using a protocol similar to the SeBCS. The SeBCS did not include women with a history of cancer. For the SeBCS1, Affymetrix 6.0 array was used for genotyping. After imputation and QC, the final data set included 2,246 cases and 2,052 controls for 7.3 million markers[11]. Additional 775 cases and 1,103 controls were included in the replication stage.

The Biobank Japan Project 1 (BBJ1):

The DNA samples were recruited from the Biobank Japan Project (http://biobankjp.org).[12,13] A total of 2,642 individuals were included in BBJ1 who registered as breast cancer patients from the Biobank Japan. For controls, a total of 2,099 for BBJ1 females consisting of healthy volunteers from Midosuji Rotary Club, Osaka, Japan, Health Science Research Resource Bank and individuals in the Biobank who do not have history of cancer for BBJ1 were included. For the BBJ1, Illumina OmniExpress BeadChiparray was used for genotyping. After imputation and QC, the final data set included 11.1 million markers.[11]

2. Description of Studies of the Breast Cancer Association Consortium (BCAC):

BCAC (Asian): The studies included in the BCAC that contributed individual-level data to the Asian-specific meta-analysis were listed as Study, Country and BCAC project(s): ACP, Thailand, Oncoarray and iCOGS; CBCS, Canada, Oncoarray; HERPACC, Japan, Oncoarray and iCOGS; HKHBCFR, Hong Kong, Oncoarray; KOHBRA, Korea, Oncoarray; LAABC, USA, iCOGS; MYBRCA, Malaysia, Oncoarray and iCOGS; NC-BCFR, USA, Oncoarray; NGOBCS, Japan, Oncoarray; SBCGS, China, Oncoarray and iCOGS; SeBCS, Korea, Oncoarray and iCOGS; SGBCC, Singapore, Oncoarray and iCOGS; TWBCS, Taiwan, Oncoarray and iCOGS.

Asia Cancer Program (ACP):

The ACP is a hospital-based case-control study conducted in Thailand. Breast cancer cases were recruited between 1999-2000, and 2008-present at The National Cancer Institute (Central region), The Prince Songkla University Research Centre (South region), The HRH Princess Maha Chakri Sirindhorn Medical Centre (MSMC)-Srinakarinviroj University (Eastern region), Khon-Kaen University Cancer Centre (North-eastern region).Women who were less than 71 years of age and underwent biopsy were eligible to participate in the study. All cases were pathologically diagnosed as having breast cancer. Women resided in the same geographic area, younger than 71 years old, and reported no prior history of cancer were recruited as controls. In total, 944 invasive cases and 1,382 controls were included in the BCAC.

Canadian Breast Cancer Study (CBCS): The CBCS is a population-based case-control study conducted in Canada.[14-17] Incident cases diagnosed between 2005 and 2009 were recruited from two areas, Vancouver, British Columbia and Kingston,

Ontario. The cases were ascertained either from the population cancer registry (Vancouver, British Columbia) or participants of the Hotel Dieu Breast Assessment Program (Kingston, Ontario). Cancer-free controls were recruited through the Screening Mammography Program of British Columbia or the Hotel Dieu Breast Assessment Program in Kingston, Ontario. Controls were frequency matched by 5-year age groups.

Hospital-based Epidemiologic Research Program at Aichi Cancer Center (HERPACC): The participants were recruited from a hospital-based case-control study conducted in Aichi, Japan.[18] All incident breast cancer cases were newly diagnosed within 1 year from the first visit to the Aichi Cancer Center between 2001 and 2013. Controls were selected from pool of non-cancer patients who firstly visited Aichi Cancer Center between 2001 and 2011. Subjects with previous cancer history were excluded.

Hong Kong Hereditary Breast Cancer (HKHBCFR):

Genetic screening of high risk breast cancer patients were approached for the study enrollment from all hospitals in Hong Kong, China between 2006 and 2014.[19-21] Controls were selected from pool of non-cancer patients who visited hospitals in Hong Kong during the same period of recruitment as cases.

Korean Hereditary Breast Cancer (KOHBRA):

The KOHBRA study is an ongoing cohort study since 2007 to examine high risk groups for hereditary breast cancer such as female breast cancer patients with a family history, ovarian cancer, or other coincidental cancers, male breast cancer patients, and family members of breast cancer patients with BRCA1/2 mutation4. Final dataset included

selected 1,397 female cancer patients without BRCA1, 2 mutation among KOHBRA subjects recruited in 2007-2009.[22]

Los Angeles County Asian-American Breast Cancer Case-Control Study (LAABC):

The LAABC is a population-based case-control study of incident breast cancer among Asian American women in Los Angeles County. Breast cancer cases were ascertained through the Los Angeles Cancer Surveillance Program. The included women were identified as Chinese, Japanese or Filipino women (aged 25-74 years) with a histologically confirmed primary breast cancer diagnosed between 1996 and 2006.[23-25] Controls were recruited from the same neighborhood as where cancer cases resided at the time of diagnosis. Cases and controls were frequency-matched on specific Asian ethnicities and 5-year age groups.

Malaysian Breast Cancer Genetic Study (MYBRCA):

Prevalent or incident breast cancer cases identified at the Breast Cancer Clinic in University Malaya Medical Centre from January 2003 to July 2014 and Subang Jaya Medical Centre from September 2012 to Sept 2014.[26] Controls are cancer-free individuals (37-74 years) selected from women attending mammographic screening at the same hospitals.

Northern California Breast Cancer Family Registry (NC-BCFR):

Incident breast cancer cases included women aged <65 years diagnosed from 1995-2009, identified through the SEER cancer registry of the Greater San Francisco Bay Area. All cases with indicators of increased genetic risk were eligible to enroll (diagnosed at age <35 years, personal history of ovarian or childhood cancer, bilateral

breast cancer with 1st diagnosis at age <50, family history of breast or ovarian cancer in first-degree relatives).[27,28] Cases not meeting these criteria were randomly sampled (2.5% of non-Hispanic whites, 32% of other race/ethnicities). Incident cases also included men aged <80 years diagnosed from 1995-1998. Controls were those unaffected family members enrolled from 1995-2011 or unaffected unrelated subjects identified through random digit dialing conducted from 1999-2000 in the San Francisco Bay Area. Controls were frequency matched to cases diagnosed from 1995-1998 on 5-year age group and race/ethnicity, at a ratio of 1 control per 2 cases. Only women were included in the current analysis.

Nagano Breast Cancer Study (NGOBCS):

The Nagano Breast Cancer Study is a multicenter, hospital-based case-control study which was conducted from May 2001 to September 2005 at four hospitals in Nagano Prefecture, Japan.[29,30] Cases were admitted to the four hospitals during the survey period who are a consecutive series of women ages 20-74 years with newly diagnosed, histologically confirmed invasive breast cancer. Among the 412 eligible patients, 405 (98%) agreed to participate. Controls were selected from medical checkup examinees in two of the hospitals who were confirmed having no cancer, with one control matched for each case by age (within three years) and residential area during the study period. Only one declined to participate among potential control subjects. Written informed consent was obtained from 405 matched pairs. Since two controls refused to provide blood samples, the analysis was restricted to 403 matched pairs. Participants completed a self-administered questionnaire, which included questions on demographic characteristics, anthropometric factors, smoking habits, family history of cancer,

physical activity, medical history, and menstrual and reproductive history. Dietary habits were investigated using a 136- item semi-quantitative food-frequency questionnaire (FFQ), which was developed and validated in the Japanese population. The ER status of the patient's breast cancer tissue was obtained from medical records. Hormone receptor positivity values were determined either as specified by the laboratory that performed the assay, in accordance with the laboratory's written interpretation thereof, or both. The study protocol was approved by the Institutional Review Board of the National Cancer Center (Tokyo, Japan).

Shanghai Breast Cancer Genetics Study (SBCGS)

The SBCGS has been described above, which also contributed samples to ABCC

Seoul Breast Cancer Study (SeBCS):

The SeBCS has been described above, which also contributed samples to ABCC.

Singapore Breast Cancer Cohort (SGBCC):

The SGBCC is an open cohort with a recruitment target of 16,000 patients diagnosed with either breast carcinoma in situ or invasive breast cancer. Recruitment started in 2010. All breast cancer patients who are at least 21 years of age at diagnosis, who are citizens or permanent residents of Singapore and who are attending any of the seven tertiary hospitals are invited to participate in SGBCC. Cases are a mixture of prevalent and incident cases. Three main ethnic groups are represented, namely, Chinese, Malays and Indians. Controls matched by age and ethnicity were selected from the Multi-ethnic Cohort (Phase 2, part of the Singapore Population Health Studies (SPHS)[31]. Exclusion criteria for controls included a medical history of cancer, acute

myocardial infarction or stroke, or major psychiatric morbidity including schizophrenia, psychotic depression, and advanced Alzheimer's disease.

Taiwanese Breast Cancer Study (TWBCS):

The study is a part of an ongoing collaborative study with a focus on understanding the cause of breast cancer among Taiwanese.[32,33] Breast cancer patients were recruited from those who were diagnosed and treated at the Tri-Service General Hospital or the Changhua Christian Hospital between March 2002 and August 2005. The controls were randomly selected from women who attended the same hospitals for a comprehensive health examination during the same period. If any evidence of breast cancer, precancerous lesions of breast or other cancers was found, the subject was excluded from the control group. Epidemiologic data was collected from the participants via a structured questionnaire by research nurses. Blood biospecimen was also collected. All the participants provided their informed consent before the data and sample collection.

BCAC (European):

Summary statistics data of European descendants from studies involved in the BCAC OncoArray, iCOGS, and GWAS projects were obtained and utilized in the cross-ancestry meta-analysis. As described in details elsewhere, 61,282 female cases with breast cancer and 45,494 female controls of European ancestry were genotyped using the OncoArray.[34] The Collaborative Oncological Gene-environment Study (iCOGS) included 46,785 breast cancer cases and 42,892 controls.[35] In addition, summary statistics from 11 other breast cancer genome-wide association studies were also used in the meta-analysis with a combined sample of 14,910 cases and 17,588 controls. The

genotyping data were imputed by IMPUTE version 2[36] with the 1000 Genomes Project Phase III as the reference panel.

3. Description of Studies included in the Sequenom replication

Korea Genome Epidemiology Study (KoGES):

The KoGES is ongoing study since 2001 to investigate major genetic and environmental factors for common diseases in the Korean population.[32] Total 1,536 women with sufficient DNA concentrations were analyzed among 10,038 subjects surveyed at baseline enrollment in 2001. Of 7,861 subjects recruited from 2005 to 2006, 1,673 women were analyzed. A total of 3,209 control subjects were selected and analyzed.

Korean Hereditary Breast Cancer (KOHBRA):

The KOHBRA study[22] as described earlier has contributed samples to both the BCAC OncoArray project and the replication stage of current study.

Korean National Cancer Cohort (KNCC)

Korea NCC study**:** Newly diagnosed breast cancer patients (cases, n=505) were recruited from the Breast Cancer Clinic at the Asan Medical Center in Seoul, Korea between February 2006 and July 2010.[37] Each case member had received a histologically confirmed diagnosis of their first primary breast cancer and participated in the study before the treatment was started. Ineligibility criteria were a previous malignancy (at either the same site or a different site) and an age greater than 80 years. The hospital controls (n=505) were women free of any malignant neoplasms and free of any clinical, biochemical, or hematological manifestations of cardiovascular, hepatic,

renal, or endocrinal disorders. All case and control subjects completed a questionnaire on lifestyle and dietary intake and provided blood samples. Informed consent was obtained from all subjects after a full explanation of the study, which had been previously approved by the institutional review board of the Korea National Cancer Center. Both case and control subjects were interviewed by one trained interviewer who was unaware of the subject's status. Using both a non-dietary questionnaire and a 95-item semi-quantitative food frequency questionnaire, information was collected on socio-demographic characteristics, anthropometric measures, individual medical history, family cancer history, and dietary factors detailing their usual food intake over the year prior to enrolment in the study. Socio-demographic characteristics included education level, occupation, cigarette smoking status, alcohol consumption, and physical activity. Pathological and laboratory data for each subject were collected, recorded, and entered into an epidemiological database. Medical charts and pathology reports were examined to ensure that control subjects had no known history of cancer. A peripheral venous blood sample (20 ml aliquot in an anticoagulant tube) was obtained from each enrolled subject. Laboratory assays of the blood samples were performed before the initiation of any treatment or therapy. Blood samples were wrapped in aluminum foil to protect against photo-oxidation, and transported to the laboratory without revealing the subject's case/control status prior to performing the antioxidant micronutrient assay. After separating plasma, samples were stored at -80°C until assayed.

Hong Kong Study:

This is a hospital-based case-control study conducted in Hong Kong, China.[38] Incident breast cancer cases were recruited during the period of 2003 to 2011 from three major

public hospitals, i.e. Queen Mary Hospital, Queen Elisabeth Hospital, and Kwong Wah Hospital. Control participants free of cancer history were recruited from the outpatients who attended the general gynaecological clinic at Queen Mary Hospital and from the Well-Women Clinic at Kwong Wah Hospital. Institutional Review Board Approval was obtained from the University of Hong Kong Hospital Authority. Consent was obtained from all the study participants for blood collection. Data of 476 cases and 278 controls were analyzed in the current study.

Taiwanese Breast Cancer Study (TWBCS):

The description of study participants of TWBCS[32,33] can be found above.

Nagoya Study (Hospital-based Epidemiologic Research Program at Aichi Cancer Center, HERPACC-II):

The HERPACC-II study is a hospital-based, comprehensive epidemiologic research program at the Aichi Cancer Center (ACC), Japan.[39] All first-visit outpatients 20-79 years of age at the ACC from December 2000 to November 2005 were asked to participate in this study. A total of 29,736 eligible patients were approached, and 28,766 participated in the study, with a response rate of 96.7%. Subjects completed a self-administered questionnaire about their lifestyle and demographic characteristics and to provide blood samples. Dietary habits were investigated using a 47-item semi-quantitative food frequency questionnaire. For cases, ER status was retrieved from medical records. ER status is routinely determined by pathologists by using commercially based immunohistochemistry tests at the ACC. Case status was confirmed by linkage of the HERPACC-II database and the hospital-based cancer registry database. A total of 1,850 histologically-confirmed breast cancer cases were

identified, and 644 were selected for the Asia Breast Cancer Consortium analysis based on availability of DNA samples. Among 14,260 non-cancer subjects in the HERPACC-II database, 644 subjects matched for age and menopausal status were randomly selected. The study protocol was approved by the Institutional Review Board at the ACC (Nagoya, Japan).

Nagano Breast Cancer Study (NGOBCS):

The description of study participants of NGOBCS[29,30] can be found above.

Supplementary Note 2.

Expression quantitative loci (eQTL) analysis

To identify robust eQTL across racial groups and different types of breast tissues, we utilized several independent sets of whole transcriptome RNA sequencing or RNA microarray and genotyping data derived from normal breast, breast tumor and adjacent normal tissues. The data of normal breast tissue were obtained from the GTEx (version 7, N = 85, women of European ancestry). We focused on *cis*-eQTL analyses for genes residing ± 500 Kb of each newly associated lead SNP. Study subjects included in the GTEx were genotyped using the Illumina OMNI 5M or 2.5M SNP Array. We excluded variants with a genotyping call rate < 98%, with differential missingness between Omni 2.5M and Omni 5M arrays, with a Hardy-Weinberg equilibrium $P < 10^{-6}$ (for subjects of European ancestry). Genotype data were imputed to the Haplotype Reference Consortium reference panel using minimac3 for imputation and SHAPEIT for prephasing. Variants with high imputation quality ($R^2 \geq 0.8$), with a MAF ≥ 0.05 were analyzed. The kilobase per million (RPKM) values of each gene from RNA-seq were log2 transformed. We removed genes with a median of 0 reads per RPKM units cross samples from the eQTL analysis. We first performed quantile normalization to ensure that the expression profile of each sample was comparable on the same scale. We then performed inverse quantile normalization for each gene to transform its expression distribution to a standard normal. To account for batch effects and experimental confounders, we followed probabilistic estimation of expression residual (PEER) protocol to generate the top 15 PEER factors and residuals[40]. We included the top ten

principal components derived from genotype data in the PEER procedure. Residuals were modeled in the downstream eQTL analysis.

At Vanderbilt, we also generated whole transcriptome RNA sequencing data (Illumina HiSeq) using adjacent normal breast tissues obtained from 200 subjects diagnosed with benign breast diseases or breast cancer (all women of East Asian ancestry). Of these, 151 subjects had genome-wide genotyping data available for eQTL analysis. We followed our previously developed pipeline to process the raw RNA-seq reads[41]. All sequencing reads were mapped to the human genome (hg19) using the Bowtie2 tool[42]. The mapped RNA-seq reads were then used to compute expression values for all coding genes and noncoding RNAs by Cufflinks (version 2.2.1)[43]. The most recent Gencode (release 27) was used for annotating coding genes and noncoding RNAs[44]. We derived fragments per kilobase of transcript per million mapped reads (FPKM) for expression levels of coding genes and lncRNAs. We performed multiple processing steps by filtering lowly-expressed genes (median FPKM =0), log2 transforming, and performing rank-based inverse normalization to transform gene expression values across samples. We additionally performed a PEER analysis to generate the top 30 PEER factors for the adjustment of batch and other potential confounding factors. Residuals from the PEER analysis were used for the downstream eQTL analysis.

We obtained genotyping and RNA-seq/microarray data measured on breast tumor tissues from the Cancer Genome Atlas project (TCGA, $N_{tumor} = 672$) and the Molecular Taxonomy of Breast Cancer International Consortium (METABRIC, $N_{tumor} =$ 1,904) project. For TCGA samples, gene expression had been measured on the

Illumina HiSeq 2000 RNA-Seq platform (gene-level RSEM normalized counts). Similar bioinformatics processes were conducted, as described above, including the removal of lowly-expressed genes, log2 transformation, and rank-based inverse normalization. Copy-number estimates were derived from the Affymetrix SNP 6.0 using the GISTIC2 algorithm[45], and methylation beta values were measured on the Illumina Infinium HumanMethylation450. Germline genotyping for the METABRIC study was done on the Affymetrix SNP 6.0 array, and gene expression in the METABRIC study was measured using the Illumina HT12 microarray platform (probe-level estimates).

A linear regression model was used to perform an eQTL analysis to estimate the additive effect of genetic variants on gene expression levels. We additionally adjusted for copy number alteration and methylation levels in the model for TCGA data. We only adjusted for copy number alteration in the analysis for the METABRIC set.

Reference

1. Zheng, W. *et al.* Genome-wide association study identifies a new breast cancer susceptibility locus at 6q25.1. *Nat Genet* **41**, 324-8 (2009).
2. Gao, Y.T. *et al.* Association of menstrual and reproductive factors with breast cancer risk: results from the Shanghai Breast Cancer Study. *Int J Cancer* **87**, 295-300 (2000).
3. Lum, A. & Le Marchand, L. A simple mouthwash method for obtaining genomic DNA in molecular epidemiological studies. *Cancer Epidemiol Biomarkers Prev* **7**, 719-24 (1998).
4. Zheng, W. *et al.* The Shanghai Women's Health Study: rationale, study design, and baseline characteristics. *Am J Epidemiol* **162**, 1123-31 (2005).
5. Zhang, Y. *et al.* Rare coding variants and breast cancer risk: evaluation of susceptibility Loci identified in genome-wide association studies. *Cancer Epidemiol Biomarkers Prev* **23**, 622-8 (2014).
6. Song, H.R. *et al.* Sex-specific differences in the association between ABO genotype and gastric cancer risk in a Korean population. *Gastric Cancer* **16**, 254-60 (2013).
7. Shim, H.J., Lee, R., Shin, M.H., Kim, H.N. & Kweon, S.S. Association between the TCF7L2 polymorphism and colorectal cancer does not differ by diabetes and obesity statuses. *Cancer Epidemiol* **45**, 108-111 (2016).
8. Kweon, S.S. *et al.* Cohort Profile: The Namwon Study and the Dong-gu Study. *Int J Epidemiol* **43**, 558-67 (2014).
9. Cho, Y.S. *et al.* A large-scale genome-wide association study of Asian populations uncovers genetic factors influencing eight quantitative traits. *Nat Genet* **41**, 527-34 (2009).
10. Han, S. *et al.* CASP8 polymorphisms, estrogen and progesterone receptor status, and breast cancer risk. *Breast Cancer Res Treat* **110**, 387-93 (2008).
11. Han, M.R. *et al.* Genome-wide association study in East Asians identifies two novel breast cancer susceptibility loci. *Hum Mol Genet* **25**, 3361-3371 (2016).
12. Elgazzar, S. *et al.* A genome-wide association study identifies a genetic variant in the SIAH2 locus associated with hormonal receptor-positive breast cancer in Japanese. *J Hum Genet* **57**, 766-71 (2012).
13. Low, S.K. *et al.* Genome-wide association study of breast cancer in the Japanese population. *PLoS One* **8**, e76463 (2013).
14. Grundy, A. *et al.* Shift work, circadian gene variants and risk of breast cancer. *Cancer Epidemiol* **37**, 606-12 (2013).
15. Kobayashi, L.C. *et al.* Moderate-to-vigorous intensity physical activity across the life course and risk of pre- and post-menopausal breast cancer. *Breast Cancer Res Treat* **139**, 851-61 (2013).
16. Grundy, A. *et al.* Increased risk of breast cancer associated with long-term shift work in Canada. *Occup Environ Med* **70**, 831-8 (2013).
17. Kobayashi, L.C. *et al.* A case-control study of lifetime light intensity physical activity and breast cancer risk. *Cancer Causes Control* **25**, 133-40 (2014).
18. Kawase, T. *et al.* FGFR2 intronic polymorphisms interact with reproductive risk factors of breast cancer: results of a case control study in Japan. *Int J Cancer* **125**, 1946-52 (2009).
19. Kwong, A. *et al.* Novel BRCA1 and BRCA2 genomic rearrangements in Southern Chinese breast/ovarian cancer patients. *Breast Cancer Res Treat* **136**, 931-3 (2012).

20. Kwong, A. *et al.* Identification of BRCA1/2 founder mutations in Southern Chinese breast cancer patients using gene sequencing and high resolution DNA melting analysis. *PLoS One* **7**, e43994 (2012).
21. Kwong, A. *et al.* Detection of Germline Mutation in Hereditary Breast and/or Ovarian Cancers by Next-Generation Sequencing on a Four-Gene Panel. *J Mol Diagn* **18**, 580-94 (2016).
22. Han, S.A. *et al.* The Korean Hereditary Breast Cancer (KOHBRA) study: protocols and interim report. *Clin Oncol (R Coll Radiol)* **23**, 434-41 (2011).
23. Wu, A.H., Yu, M.C., Tseng, C.C., Stanczyk, F.Z. & Pike, M.C. Dietary patterns and breast cancer risk in Asian American women. *Am J Clin Nutr* **89**, 1145-54 (2009).
24. Wu, A.H., McKean-Cowdin, R. & Tseng, C.C. Birth weight and other prenatal factors and risk of breast cancer in Asian-Americans. *Breast Cancer Res Treat* **130**, 917-25 (2011).
25. Wu, A.H., Vigen, C., Butler, L.M. & Tseng, C.C. Metabolic conditions and breast cancer risk among Los Angeles County Filipina Americans compared with Chinese and Japanese Americans. *Int J Cancer* **141**, 2450-2461 (2017).
26. Phuah, S.Y. *et al.* Triple-negative breast cancer and PTEN (phosphatase and tensin homologue) loss are predictors of BRCA1 germline mutations in women with early-onset and familial breast cancer, but not in women with isolated late-onset breast cancer. *Breast Cancer Res* **14**, R142 (2012).
27. John, E.M. *et al.* The Breast Cancer Family Registry: an infrastructure for cooperative multinational, interdisciplinary and translational studies of the genetic epidemiology of breast cancer. *Breast Cancer Res* **6**, R375-89 (2004).
28. Terry, M.B. *et al.* Cohort Profile: The Breast Cancer Prospective Family Study Cohort (ProF-SC). *Int J Epidemiol* **45**, 683-92 (2016).
29. Itoh, H. *et al.* Serum organochlorines and breast cancer risk in Japanese women: a case-control study. *Cancer Causes Control* **20**, 567-80 (2009).
30. Shimada, N. *et al.* Genetic polymorphisms in estrogen metabolism and breast cancer risk in case-control studies in Japanese, Japanese Brazilians and non-Japanese Brazilians. *J Hum Genet* **54**, 209-15 (2009).
31. Tan, K.H.X. *et al.* Cohort Profile: The Singapore Multi-Ethnic Cohort (MEC) study. *Int J Epidemiol* (2018).
32. Hsu, H.M. *et al.* Breast cancer risk is associated with the genes encoding the DNA double-strand break repair Mre11/Rad50/Nbs1 complex. *Cancer Epidemiol Biomarkers Prev* **16**, 2024-32 (2007).
33. Ding, S.L. *et al.* Genetic variants of BLM interact with RAD51 to increase breast cancer susceptibility. *Carcinogenesis* **30**, 43-9 (2009).
34. Michailidou, K. *et al.* Association analysis identifies 65 new breast cancer risk loci. *Nature* **551**, 92-94 (2017).
35. Michailidou, K. *et al.* Large-scale genotyping identifies 41 new loci associated with breast cancer risk. *Nat Genet* **45**, 353-61, 361e1-2 (2013).
36. Howie, B.N., Donnelly, P. & Marchini, J. A flexible and accurate genotype imputation method for the next generation of genome-wide association studies. *PLoS Genet* **5**, e1000529 (2009).
37. Long, J. *et al.* Genome-wide association study in east Asians identifies novel susceptibility loci for breast cancer. *PLoS Genet* **8**, e1002532 (2012).

38. Chan, K.Y. *et al.* Functional polymorphisms in the BRCA1 promoter influence transcription and are associated with decreased risk for breast cancer in Chinese women. *J Med Genet* **46**, 32-9 (2009).
39. Hamajima, N. *et al.* Gene-environment Interactions and Polymorphism Studies of Cancer Risk in the Hospital-based Epidemiologic Research Program at Aichi Cancer Center II (HERPACC-II). *Asian Pac J Cancer Prev* **2**, 99-107 (2001).
40. Stegle, O., Parts, L., Piipari, M., Winn, J. & Durbin, R. Using probabilistic estimation of expression residuals (PEER) to obtain increased power and interpretability of gene expression analyses. *Nat Protoc* **7**, 500-7 (2012).
41. Guo, X., Lin, M., Rockowitz, S., Lachman, H.M. & Zheng, D. Characterization of human pseudogene-derived non-coding RNAs for functional potential. *PLoS One* **9**, e93972 (2014).
42. Langmead, B. & Salzberg, S.L. Fast gapped-read alignment with Bowtie 2. *Nat Methods* **9**, 357-9 (2012).
43. Trapnell, C. *et al.* Differential gene and transcript expression analysis of RNA-seq experiments with TopHat and Cufflinks. *Nat Protoc* **7**, 562-78 (2012).
44. Harrow, J. *et al.* GENCODE: the reference human genome annotation for The ENCODE Project. *Genome Res* **22**, 1760-74 (2012).
45. Mermel, C.H. *et al.* GISTIC2.0 facilitates sensitive and confident localization of the targets of focal somatic copy-number alteration in human cancers. *Genome Biol* **12**, R41 (2011).
