# Supplementary material for: Identification of novel breast cancer susceptibility loci in meta-analyses conducted among Asian and European descendants
Source: Nat Commun. 2020 Mar 5;11:1217. doi: 10.1038/s41467-020-15046-w (PMC7057957; doi:10.1038/s41467-020-15046-w)
Supplement: Supplementary file 3 — Description of Additional Supplementary Files [file 41467_2020_15046_MOESM3_ESM.pdf]

Description of Additional Supplementary files

File name: Supplementary Data 1
Description: Association results of 28 loci in each Asian study

File Name: Supplementary Data 2
Description: Association results of 28 loci in each dataset of BCAC Europeans

File Name: Supplementary Data 3
Description: Regulatory functional annotation of SNPs in high LD (r2>0.8) with newly associated SNPs in Asian populations using Haploreg

File Name: Supplementary Data 4
Description: Regulatory functional annotation of SNPs in high LD (r2>0.8) with newly-associated SNPs in European populations using Haploreg

File Name: Supplementary Data 5
Description: Replication of breast cancer susceptibility SNPs previously reported

File name: Supplementary Data 6
Description: Independent secondary associations found within previously known loci: A conditional analysis
