# Supplementary material for: Identification of novel breast cancer susceptibility loci in meta-analyses conducted among Asian and European descendants
Source: Nat Commun. 2020 Mar 5;11:1217. doi: 10.1038/s41467-020-15046-w (PMC7057957; doi:10.1038/s41467-020-15046-w)
Supplement: Supplementary file 4 — Supplementary Data 1 [file 41467_2020_15046_MOESM4_ESM.pdf]

Supplementary Data 1. Association results of 28 loci in each Asian study

SNP	Chr	BP	Test	Other	Dataset	EAF	OR (95% CI)	P	Imputation R^2	I^2, %	$P_{heterogeneity}$
rs72906468	1	17772093	A	T	BCAC Asian iCOGs	0.69	1.03 (0.97-1.10)	0.381	0.88	0	0.708
			A	T	BCAC Asian OncoArray	0.69	1.06 (1.00-1.11)	0.045	0.94		
			A	T	Japanese GWAS	0.68	1.06 (0.97-1.16)	0.196	0.91		
			A	T	Korean GWAS	0.70	1.16 (1.05-1.29)	0.005	0.81		
			A	T	MEGA HCES-1	0.72	0.96 (0.71-1.29)	0.774	0.89		
			A	T	MEGA KPOP-BRCA	0.71	1.10 (0.94-1.28)	0.220	0.89		
			A	T	MEGA SH	0.67	1.07 (0.97-1.19)	0.174	0.89		
			A	T	Shanghai BC GWAS	0.67	1.05 (0.96-1.16)	0.276	0.84		
rs3790585	1	46023356	A	T	Asian ExomeChip	0.77	0.93 (0.81-1.07)	0.303	0.58	15.2%	0.307
			A	T	BCAC Asian iCOGs	0.68	1.09 (1.02-1.15)	0.007	1.00		
			A	T	BCAC Asian OncoArray	0.68	1.01 (0.96-1.06)	0.799	1.00		
			A	T	Japanese GWAS	0.70	1.06 (0.97-1.16)	0.172	0.99		
			A	T	Korean GWAS	0.75	1.12 (1.01-1.24)	0.031	0.97		
			A	T	MEGA HCES-1	0.71	1.20 (0.90-1.58)	0.209	0.99		
			A	T	MEGA KPOP-BRCA	0.73	1.02 (0.89-1.18)	0.739	0.99		
			A	T	MEGA SH	0.67	1.05 (0.95-1.15)	0.357	0.99		
			A	T	Shanghai BC GWAS	0.67	1.07 (0.98-1.17)	0.139	0.98		
rs2758598	1	156194339	A	G	Asian ExomeChip	0.20	1.02 (0.89-1.18)	0.760	0.64	54.2%	0.025
			A	G	BCAC Asian iCOGs	0.16	1.01 (0.94-1.09)	0.783	0.99		
			A	G	BCAC Asian OncoArray	0.16	1.12 (1.05-1.20)	4.83×10^{-4}	1.00		
			A	G	Japanese GWAS	0.17	1.15 (1.03-1.28)	0.014	0.99		
			A	G	Korean GWAS	0.06	1.29 (0.97-1.71)	0.082	0.41		
			A	G	MEGA HCES-1	0.14	1.11 (0.78-1.58)	0.567	0.98		
			A	G	MEGA KPOP-BRCA	0.15	1.33 (1.11-1.60)	0.002	0.98		
			A	G	MEGA SH	0.16	1.00 (0.88-1.13)	1.000	0.98		
			A	G	Shanghai BC GWAS	0.16	0.97 (0.86-1.09)	0.561	0.90		
rs6756513	2	70172587	A	G	Asian ExomeChip	0.28	0.94 (0.84-1.06)	0.325	0.81	0	0.711
			A	G	BCAC Asian iCOGs	0.30	0.96 (0.90-1.03)	0.303	0.79		
			A	G	BCAC Asian OncoArray	0.29	0.96 (0.91-1.01)	0.137	0.95		
			A	G	Japanese GWAS	0.36	0.95 (0.88-1.04)	0.271	1.00		
			A	G	Korean GWAS	0.32	0.95 (0.87-1.05)	0.322	1.00		

			A	G	MEGA HCES-1	0.33	1.07 (0.82-1.40)	0.599	0.97		
			A	G	MEGA KPOP-BRCA	0.32	0.95 (0.83-1.10)	0.492	0.97		
			A	G	MEGA SH	0.26	1.07 (0.96-1.18)	0.229	0.97		
			A	G	Shanghai BC GWAS	0.27	0.92 (0.84-1.01)	0.082	0.99		
rs73006998	3	150464271	A	G	BCAC Asian iCOGs	0.30	0.97 (0.91-1.04)	0.415	0.86	4.6%	0.394
			A	G	BCAC Asian OncoArray	0.32	0.89 (0.85-0.94)	1.84×10^{-5}	0.99		
			A	G	Japanese GWAS	0.31	0.90 (0.83-0.99)	0.024	1.00		
			A	G	Korean GWAS	0.36	0.97 (0.88-1.06)	0.484	0.99		
			A	G	MEGA HCES-1	0.39	0.88 (0.68-1.12)	0.295	1.00		
			A	G	MEGA KPOP-BRCA	0.36	0.90 (0.79-1.03)	0.114	1.00		
			A	G	MEGA SH	0.35	0.90 (0.82-0.99)	0.033	1.00		
			A	G	Shanghai BC GWAS	0.33	0.87 (0.79-0.95)	0.001	0.98		
rs11281251	3	156519412	T	TTGTGAC	BCAC Asian iCOGs	0.19	0.97 (0.90-1.04)	0.372	0.99	15.4%	0.315
			T	TTGTGAC	BCAC Asian OncoArray	0.17	0.90 (0.85-0.96)	0.001	0.99		
			T	TTGTGAC	MEGA HCES-1	0.20	1.13 (0.82-1.55)	0.464	0.96		
			T	TTGTGAC	MEGA KPOP-BRCA	0.21	1.02 (0.87-1.20)	0.831	0.96		
			T	TTGTGAC	MEGA SH	0.17	0.89 (0.79-1.01)	0.061	0.96		
			T	TTGTGAC	Shanghai BC GWAS	0.17	0.98 (0.88-1.10)	0.762	0.98		
rs11944638	4	48227719	T	C	Asian ExomeChip	0.81	1.16 (0.96-1.39)	0.119	0.41	0	0.898
			T	C	BCAC Asian iCOGs	0.76	1.09 (1.01-1.17)	0.022	0.87		
			T	C	BCAC Asian OncoArray	0.77	1.05 (0.99-1.11)	0.111	0.97		
			T	C	Japanese GWAS	0.68	1.06 (0.97-1.16)	0.183	0.97		
			T	C	Korean GWAS	0.67	1.08 (0.98-1.19)	0.133	0.94		
			T	C	MEGA HCES-1	0.65	1.16 (0.89-1.49)	0.273	0.97		
			T	C	MEGA KPOP-BRCA	0.67	1.14 (1.00-1.31)	0.056	0.97		
			T	C	MEGA SH	0.76	1.04 (0.93-1.15)	0.518	0.97		
			T	C	Shanghai BC GWAS	0.75	1.11 (1.01-1.22)	0.034	0.92		
rs11947923	4	53911337	T	C	BCAC Asian iCOGs	0.28	0.96 (0.90-1.02)	0.174	0.91	0	0.687
			T	C	BCAC Asian OncoArray	0.29	0.99 (0.93-1.04)	0.597	0.90		
			T	C	Japanese GWAS	0.26	0.96 (0.88-1.05)	0.403	0.98		
			T	C	Korean GWAS	0.29	0.97 (0.88-1.08)	0.601	0.89		
			T	C	MEGA HCES-1	0.27	0.73 (0.54-1.00)	0.053	0.79		
			T	C	MEGA KPOP-BRCA	0.29	0.99 (0.85-1.17)	0.943	0.79		
			T	C	MEGA SH	0.29	0.94 (0.84-1.05)	0.261	0.79		

			T	C	Shanghai BC GWAS	0.29	0.92 (0.84-1.01)	0.094	0.92		
rs6555134	5	2776483	T	C	BCAC Asian iCOGs	0.26	0.97 (0.91-1.04)	0.382	0.97	0	0.806
			T	C	BCAC Asian OncoArray	0.25	0.95 (0.90-1.00)	0.057	0.97		
			T	C	Japanese GWAS	0.26	1.00 (0.91-1.09)	0.946	0.98		
			T	C	Korean GWAS	0.29	0.96 (0.87-1.06)	0.434	0.97		
			T	C	MEGA HCES-1	0.27	0.91 (0.69-1.20)	0.502	0.99		
			T	C	MEGA KPOP-BRCA	0.29	0.88 (0.77-1.02)	0.081	0.99		
			T	C	MEGA SH	0.25	0.94 (0.85-1.05)	0.264	0.99		
			T	C	Shanghai BC GWAS	0.24	0.90 (0.82-1.00)	0.042	0.94		
rs7765429	6	21904169	T	C	BCAC Asian iCOGs	0.88	0.97 (0.89-1.06)	0.447	1.00	0	0.444
			T	C	BCAC Asian OncoArray	0.88	0.93 (0.87-1.01)	0.075	1.00		
			T	C	Japanese GWAS	0.95	0.96 (0.80-1.16)	0.675	1.00		
			T	C	Korean GWAS	0.92	0.87 (0.73-1.03)	0.112	0.89		
			T	C	MEGA HCES-1	0.92	0.87 (0.54-1.40)	0.558	0.99		
			T	C	MEGA KPOP-BRCA	0.92	1.22 (0.95-1.56)	0.118	0.99		
			T	C	MEGA SH	0.91	0.91 (0.78-1.06)	0.231	0.99		
			T	C	Shanghai BC GWAS	0.91	0.87 (0.75-1.01)	0.060	0.92		
rs7768862	6	85088846	A	T	Asian ExomeChip	0.35	0.87 (0.75-1.02)	0.084	0.37	0	0.745
			A	T	BCAC Asian iCOGs	0.29	0.95 (0.90-1.02)	0.141	0.98		
			A	T	BCAC Asian OncoArray	0.29	0.93 (0.88-0.98)	0.006	1.00		
			A	T	Japanese GWAS	0.29	1.02 (0.93-1.11)	0.698	1.00		
			A	T	Korean GWAS	0.28	0.96 (0.87-1.06)	0.397	0.99		
			A	T	MEGA HCES-1	0.29	0.85 (0.64-1.13)	0.252	0.93		
			A	T	MEGA KPOP-BRCA	0.28	0.92 (0.79-1.06)	0.246	0.93		
			A	T	MEGA SH	0.29	0.95 (0.86-1.06)	0.376	0.93		
			A	T	Shanghai BC GWAS	0.29	0.93 (0.85-1.02)	0.142	0.98		
rs6940159	6	170332621	T	C	BCAC Asian iCOGs	0.82	0.97 (0.90-1.04)	0.412	0.95	0	0.526
			T	C	BCAC Asian OncoArray	0.83	0.96 (0.90-1.03)	0.249	0.96		
			T	C	Japanese GWAS	0.81	0.86 (0.78-0.96)	0.006	1.00		
			T	C	Korean GWAS	0.82	0.88 (0.78-0.99)	0.037	0.91		
			T	C	MEGA HCES-1	0.82	0.96 (0.70-1.32)	0.796	1.00		
			T	C	MEGA KPOP-BRCA	0.83	1.02 (0.86-1.20)	0.849	1.00		
			T	C	MEGA SH	0.82	0.91 (0.81-1.03)	0.124	1.00		
			T	C	Shanghai BC GWAS	0.82	0.93 (0.83-1.03)	0.158	0.97		

rs144145984	8	23644003	CT	C	Asian ExomeChip	0.47	0.86 (0.77-0.96)	0.009	0.66	0	0.502
			CT	C	BCAC Asian iCOGs	0.42	0.97 (0.92-1.03)	0.382	0.99		
			CT	C	BCAC Asian OncoArray	0.43	0.98 (0.93-1.03)	0.377	0.98		
			CT	C	Japanese GWAS	0.44	1.01 (0.93-1.09)	0.867	0.97		
			CT	C	Korean GWAS	0.41	0.97 (0.87-1.07)	0.522	0.91		
			CT	C	MEGA HCES-1	0.41	0.89 (0.69-1.15)	0.365	0.97		
			CT	C	MEGA KPOP-BRCA	0.39	0.92 (0.80-1.05)	0.220	0.97		
			CT	C	MEGA SH	0.43	0.93 (0.85-1.02)	0.142	0.97		
			CT	C	Shanghai BC GWAS	0.42	0.93 (0.86-1.01)	0.101	0.97		
rs2849506	8	101329134	C	G	Asian ExomeChip	0.50	0.98 (0.89-1.07)	0.643	0.94	0	0.967
			C	G	BCAC Asian iCOGs	0.48	0.94 (0.88-0.99)	0.025	0.96		
			C	G	BCAC Asian OncoArray	0.48	0.96 (0.92-1.01)	0.111	0.99		
			C	G	Japanese GWAS	0.51	0.97 (0.89-1.05)	0.443	0.95		
			C	G	Korean GWAS	0.40	0.97 (0.87-1.08)	0.581	0.81		
			C	G	MEGA HCES-1	0.49	1.10 (0.85-1.41)	0.479	0.99		
			C	G	MEGA KPOP-BRCA	0.48	0.94 (0.83-1.07)	0.358	0.99		
			C	G	MEGA SH	0.50	0.96 (0.88-1.05)	0.414	0.99		
			C	G	Shanghai BC GWAS	0.50	0.94 (0.86-1.02)	0.112	1.00		
rs142360995	8	118205719	A	G	Asian ExomeChip	0.14	1.10 (0.89-1.36)	0.370	0.39	47.2%	0.056
			A	G	BCAC Asian iCOGs	0.09	1.19 (1.06-1.33)	0.003	0.79		
			A	G	BCAC Asian OncoArray	0.09	1.07 (0.98-1.16)	0.131	1.00		
			A	G	Japanese GWAS	0.05	1.00 (0.84-1.21)	0.965	0.95		
			A	G	Korean GWAS	0.05	1.19 (0.93-1.54)	0.164	0.72		
			A	G	MEGA HCES-1	0.09	0.74 (0.48-1.15)	0.185	0.99		
			A	G	MEGA KPOP-BRCA	0.07	1.57 (1.22-2.02)	4.71×10^{-4}	0.99		
			A	G	MEGA SH	0.10	1.22 (1.04-1.42)	0.013	0.99		
			A	G	Shanghai BC GWAS	0.09	1.12 (0.97-1.30)	0.115	0.98		
rs10820600	9	106856692	T	C	BCAC Asian iCOGs	0.80	0.99 (0.92-1.07)	0.837	1.00	34.1%	0.156
			T	C	BCAC Asian OncoArray	0.81	0.95 (0.89-1.01)	0.078	1.00		
			T	C	Japanese GWAS	0.86	1.07 (0.95-1.20)	0.247	1.00		
			T	C	Korean GWAS	0.84	0.87 (0.77-0.98)	0.028	0.94		
			T	C	MEGA HCES-1	0.83	0.80 (0.58-1.11)	0.182	0.99		
			T	C	MEGA KPOP-BRCA	0.83	0.90 (0.76-1.06)	0.215	0.99		
			T	C	MEGA SH	0.82	0.88 (0.78-0.99)	0.032	0.99		

			T	C	Shanghai BC GWAS	0.83	0.96 (0.86-1.07)	0.469	0.98		
rs541079479	10	22861533	CA	C	BCAC Asian iCOGs	0.13	1.08 (0.99-1.18)	0.067	0.92	0	0.884
			CA	C	BCAC Asian OncoArray	0.13	1.07 (0.99-1.15)	0.079	0.90		
			CA	C	Japanese GWAS	0.08	1.10 (0.93-1.29)	0.262	0.89		
			CA	C	MEGA HCES-1	0.14	1.06 (0.72-1.56)	0.764	0.89		
			CA	C	MEGA KPOP-BRCA	0.12	0.97 (0.78-1.20)	0.774	0.89		
			CA	C	MEGA SH	0.15	1.06 (0.93-1.21)	0.352	0.89		
			CA	C	Shanghai BC GWAS	0.16	0.99 (0.88-1.12)	0.891	0.87		
rs2901157	10	119262365	A	G	BCAC Asian iCOGs	0.77	1.06 (0.99-1.14)	0.094	0.89	0	0.998
			A	G	BCAC Asian OncoArray	0.76	1.06 (1.00-1.12)	0.037	1.00		
			A	G	Japanese GWAS	0.69	1.08 (0.99-1.17)	0.094	1.00		
			A	G	Korean GWAS	0.71	1.07 (0.97-1.17)	0.198	1.00		
			A	G	MEGA HCES-1	0.72	1.06 (0.79-1.42)	0.695	0.88		
			A	G	MEGA KPOP-BRCA	0.74	1.05 (0.90-1.22)	0.562	0.88		
			A	G	MEGA SH	0.80	1.01 (0.90-1.14)	0.808	0.88		
			A	G	Shanghai BC GWAS	0.78	1.05 (0.95-1.16)	0.318	1.00		
rs10838267	11	44368892	A	G	BCAC Asian iCOGs	0.32	1.04 (0.98-1.11)	0.182	0.99	0	0.548
			A	G	BCAC Asian OncoArray	0.32	1.09 (1.04-1.15)	0.000	0.99		
			A	G	Japanese GWAS	0.28	1.07 (0.98-1.17)	0.154	1.00		
			A	G	MEGA HCES-1	0.32	1.13 (0.87-1.47)	0.374	0.97		
			A	G	MEGA KPOP-BRCA	0.32	1.05 (0.91-1.20)	0.520	0.97		
			A	G	MEGA SH	0.36	1.09 (0.99-1.20)	0.071	0.97		
			A	G	Shanghai BC GWAS	0.37	0.99 (0.91-1.08)	0.809	0.99		
rs78588049	12	69180907	A	ATTTT	BCAC Asian iCOGs	0.15	0.97 (0.90-1.05)	0.432	0.97	0	0.523
			A	ATTTT	BCAC Asian OncoArray	0.16	0.89 (0.83-0.95)	3.85×10^{-4}	0.98		
			A	ATTTT	Japanese GWAS	0.15	1.00 (0.89-1.12)	0.956	0.99		
			A	ATTTT	MEGA HCES-1	0.15	0.86 (0.59-1.24)	0.409	0.92		
			A	ATTTT	MEGA KPOP-BRCA	0.13	0.93 (0.77-1.14)	0.501	0.92		
			A	ATTTT	MEGA SH	0.16	0.91 (0.80-1.04)	0.165	0.92		
			A	ATTTT	Shanghai BC GWAS	0.16	0.97 (0.87-1.09)	0.631	0.95		
rs855596	12	103045519	T	C	BCAC Asian iCOGs	0.07	0.85 (0.76-0.95)	0.003	1.00	11.9%	0.338
			T	C	BCAC Asian OncoArray	0.07	0.94 (0.85-1.03)	0.160	0.97		
			T	C	Japanese GWAS	0.07	0.94 (0.80-1.10)	0.423	1.00		
			T	C	Korean GWAS	0.08	0.88 (0.75-1.04)	0.139	0.98		

			T	C	MEGA HCES-1	0.07	1.59 (0.96-2.65)	0.072	0.99		
			T	C	MEGA KPOP-BRCA	0.08	0.97 (0.76-1.24)	0.821	0.99		
			T	C	MEGA SH	0.08	0.87 (0.73-1.04)	0.120	0.99		
			T	C	Shanghai BC GWAS	0.07	0.85 (0.72-0.99)	0.041	1.00		
rs9316500	13	51094114	T	G	Asian ExomeChip	0.44	0.98 (0.85-1.14)	0.827	0.38	0.4%	0.431
			T	G	BCAC Asian iCOGs	0.37	1.10 (1.03-1.16)	0.002	0.99		
			T	G	BCAC Asian OncoArray	0.35	1.03 (0.98-1.08)	0.286	1.00		
			T	G	Japanese GWAS	0.42	1.07 (0.98-1.16)	0.134	1.00		
			T	G	Korean GWAS	0.33	1.12 (1.02-1.22)	0.021	1.00		
			T	G	MEGA HCES-1	0.32	1.21 (0.93-1.57)	0.161	1.00		
			T	G	MEGA KPOP-BRCA	0.33	1.04 (0.91-1.19)	0.590	1.00		
			T	G	MEGA SH	0.33	1.02 (0.92-1.12)	0.721	1.00		
			T	G	Shanghai BC GWAS	0.33	1.00 (0.92-1.09)	0.984	1.00		
rs75004998	14	77517786	A	G	BCAC Asian iCOGs	0.49	0.93 (0.88-0.98)	0.010	1.00	0	0.878
			A	G	BCAC Asian OncoArray	0.50	0.96 (0.92-1.01)	0.116	1.00		
			A	G	Japanese GWAS	0.50	0.97 (0.90-1.06)	0.513	1.00		
			A	G	Korean GWAS	0.54	0.99 (0.90-1.08)	0.762	0.90		
			A	G	MEGA HCES-1	0.54	0.93 (0.72-1.19)	0.552	0.96		
			A	G	MEGA KPOP-BRCA	0.56	0.98 (0.86-1.12)	0.785	0.96		
			A	G	MEGA SH	0.52	0.98 (0.90-1.07)	0.680	0.96		
			A	G	Shanghai BC GWAS	0.52	1.00 (0.92-1.09)	0.975	0.97		
rs8027365	15	75808740	A	C	Asian ExomeChip	0.64	1.02 (0.91-1.15)	0.707	0.67	16.8%	0.293
			A	C	BCAC Asian iCOGs	0.63	1.08 (1.02-1.14)	0.011	0.98		
			A	C	BCAC Asian OncoArray	0.64	1.02 (0.97-1.07)	0.544	0.99		
			A	C	Japanese GWAS	0.57	1.10 (1.02-1.20)	0.021	0.98		
			A	C	Korean GWAS	0.61	0.96 (0.87-1.06)	0.440	0.85		
			A	C	MEGA HCES-1	0.62	1.08 (0.84-1.40)	0.547	0.98		
			A	C	MEGA KPOP-BRCA	0.61	1.00 (0.88-1.15)	0.953	0.98		
			A	C	MEGA SH	0.64	1.03 (0.94-1.14)	0.478	0.98		
			A	C	Shanghai BC GWAS	0.63	1.12 (1.02-1.22)	0.013	0.93		
rs76535198	16	71892498	A	C	Asian ExomeChip	0.76	1.07 (0.94-1.21)	0.303	0.72	0	0.626
			A	C	BCAC Asian iCOGs	0.71	1.13 (1.06-1.20)	1.06×10^{-4}	1.00		
			A	C	BCAC Asian OncoArray	0.73	1.03 (0.97-1.08)	0.363	1.00		
			A	C	Japanese GWAS	0.66	1.08 (0.99-1.17)	0.089	1.00		

			A	C	Korean GWAS	0.71	1.09 (0.98-1.22)	0.117	0.79		
			A	C	MEGA HCES-1	0.71	1.10 (0.84-1.44)	0.487	0.99		
			A	C	MEGA KPOP-BRCA	0.69	1.05 (0.91-1.22)	0.482	0.99		
			A	C	MEGA SH	0.76	1.09 (0.99-1.22)	0.093	0.99		
			A	C	Shanghai BC GWAS	0.76	1.10 (1.00-1.22)	0.051	0.94		
rs12481286	20	52287610	T	G	BCAC Asian iCOGs	0.32	1.06 (0.99-1.13)	0.078	0.93	1.7%	0.416
			T	G	BCAC Asian OncoArray	0.32	1.04 (0.98-1.09)	0.212	0.87		
			T	G	Japanese GWAS	0.27	1.04 (0.95-1.14)	0.426	0.97		
			T	G	Korean GWAS	0.29	0.96 (0.87-1.06)	0.449	0.99		
			T	G	MEGA HCES-1	0.30	1.29 (0.97-1.71)	0.076	0.99		
			T	G	MEGA KPOP-BRCA	0.28	1.03 (0.90-1.19)	0.643	0.99		
			T	G	MEGA SH	0.34	1.04 (0.95-1.15)	0.394	0.99		
			T	G	Shanghai BC GWAS	0.34	1.11 (1.02-1.22)	0.017	0.97		
rs35418111	21	47856670	A	G	Asian ExomeChip	0.19	1.04 (0.88-1.23)	0.649	0.46	0	0.962
			A	G	BCAC Asian iCOGs	0.20	1.09 (1.02-1.17)	0.016	0.96		
			A	G	BCAC Asian OncoArray	0.20	1.05 (0.99-1.12)	0.092	0.97		
			A	G	Japanese GWAS	0.22	1.05 (0.95-1.16)	0.384	0.95		
			A	G	Korean GWAS	0.20	1.08 (0.96-1.21)	0.214	0.84		
			A	G	MEGA HCES-1	0.21	1.08 (0.79-1.47)	0.645	0.94		
			A	G	MEGA KPOP-BRCA	0.21	1.02 (0.87-1.20)	0.789	0.94		
			A	G	MEGA SH	0.22	1.12 (1.01-1.26)	0.039	0.94		
			A	G	Shanghai BC GWAS	0.21	1.12 (1.00-1.24)	0.045	0.88		
rs34331122	22	19762428	CTT	C	BCAC Asian iCOGs	0.55	0.94 (0.88-1.00)	0.034	0.91	0	0.846
			CTT	C	BCAC Asian OncoArray	0.55	0.96 (0.91-1.01)	0.089	0.94		
			CTT	C	Japanese GWAS	0.53	0.91 (0.83-0.99)	0.031	0.89		
			CTT	C	MEGA HCES-1	0.58	0.98 (0.74-1.28)	0.870	0.89		
			CTT	C	MEGA KPOP-BRCA	0.55	0.88 (0.76-1.01)	0.062	0.89		
			CTT	C	MEGA SH	0.60	0.91 (0.82-1.00)	0.058	0.89		
			CTT	C	Shanghai BC GWAS	0.61	0.95 (0.85-1.07)	0.402	0.56		
