# Supplementary material for: Identification of novel breast cancer susceptibility loci in meta-analyses conducted among Asian and European descendants
Source: Nat Commun. 2020 Mar 5;11:1217. doi: 10.1038/s41467-020-15046-w (PMC7057957; doi:10.1038/s41467-020-15046-w)
Supplement: Supplementary file 5 — Supplementary Data 2 [file 41467_2020_15046_MOESM5_ESM.pdf]

Supplementary Data 2. Association results of 28 loci in each dataset of BCAC Europeans

SNP	Chr	BP	Test	Other	Dataset	EAF	OR (95% CI)	P
rs72906468	1	17772093	A	T	Meta	0.77	1.04(1.02-1.05)	2.3×10^{-6}
					GWAS	0.78	1.08(1.03-1.12)	4.3×10^{-4}
					iCOGS	0.78	1.02(0.99-1.04)	0.16
					OncoArray	0.77	1.04(1.02-1.06)	1.6×10^{-4}
rs3790585	1	46023356	A	T	Meta	0.84	1.03(1.02-1.06)	8.8×10^{-7}
					GWAS	0.85	1.03(0.98-1.08)	0.31
					iCOGS	0.85	1.04(1.02-1.08)	1.9×10^{-3}
					OncoArray	0.85	1.05(1.02-1.08)	1.6×10^{-4}
rs2758598	1	156194339	A	G	Meta	0.33	1.03(1.02-1.05)	8.4×10^{-7}
					GWAS	0.33	1.03(1.00-1.03)	0.09
					iCOGS	0.33	1.03(1.01-1.05)	5.2×10^{-3}
					OncoArray	0.33	1.04(1.02-1.06)	2.1×10^{-4}
rs6756513	2	70172587	A	G	Meta	0.29	0.96(0.95-0.98)	4.2×10^{-7}
					GWAS	0.28	0.95(0.92-0.99)	0.01
					iCOGS	0.29	0.97(0.94-0.99)	2.9×10^{-3}
					OncoArray	0.28	0.97(0.95-0.99)	1.0×10^{-3}
rs73006998	3	150464271	A	G	Meta	0.03	0.94(0.91-0.98)	5.8×10^{-3}
					GWAS	0.03	0.96(0.85-1.09)	0.56
					iCOGS	0.03	0.92(0.87-0.98)	0.01
					OncoArray	0.02	0.96(0.91-1.02)	0.18
rs11281251	3	156519412	T	TTGTGAC	Meta	0.39	0.97(0.96-0.98)	4.2×10^{-7}
					GWAS	0.38	0.97(0.93-1.00)	0.07
					iCOGS	0.39	1.04(1.01-1.06)	5.6×10^{-4}
					OncoArray	0.39	1.03(1.01-1.05)	1.2×10^{-3}
rs11947923	4	53911337	T	C	Meta	0.37	0.97(0.96-0.98)	1.0×10^{-6}
					GWAS	0.38	0.96(0.93-0.99)	0.02
					iCOGS	0.37	0.98(0.96-1.00)	0.04
					OncoArray	0.37	0.96(0.94-0.98)	5.5×10^{-5}
rs11944638	4	48227719	T	C	Meta	0.93	1.05(1.02-1.08)	3.1×10^{-4}
					GWAS	0.93	1.07(1.00-1.15)	0.04
					iCOGS	0.93	1.05(1.01-1.10)	0.01
					OncoArray	0.93	1.04(1.00-1.08)	0.06
rs6555134	5	2776483	T	C	Meta	0.58	0.97(0.96-0.98)	3.6×10^{-7}
					GWAS	0.58	0.97(0.93-1.01)	0.11
					iCOGS	0.58	0.97(0.95-0.99)	0.01
					OncoArray	0.58	0.96(0.94-0.98)	2.0×10^{-5}
rs7765429	6	21904169	T	C	Meta	0.46	0.97(0.96-0.98)	3.3×10^{-7}
					GWAS	0.46	0.97(0.94-1.01)	0.12

					iCOGS	0.46	0.97(0.95-0.98)	3.1×10^{-4}
					OncoArray	0.47	0.97(0.95-0.99)	1.0×10^{-3}
rs7768862	6	85088846	A	T	Meta	0.51	0.97(0.96-0.98)	6.5×10^{-6}
					GWAS	0.51	0.97(0.93-1.00)	0.06
					iCOGS	0.51	0.97(0.95-0.98)	4.0×10^{-4}
					OncoArray	0.50	0.98(0.96-1.00)	0.02
rs6940159	6	170332621	T	C	Meta	0.38	0.97(0.96-0.98)	2.7×10^{-7}
					GWAS	0.39	1.00(0.97-1.04)	0.98
					iCOGS	0.38	0.96(0.94-0.98)	1.6×10^{-4}
					OncoArray	0.39	0.96(0.94-0.98)	6.2×10^{-5}
rs144145984	8	23644003	CT	C	Meta	0.57	0.97(0.96-0.98)	1.7×10^{-6}
					GWAS	0.57	0.96(0.93-1.00)	0.06
					iCOGS	0.58	0.98(0.96-0.99)	0.01
					OncoArray	0.58	0.97(0.95-0.98)	2.6×10^{-4}
rs2849506	8	101329134	C	G	Meta	0.40	0.97(0.96-0.98)	7.5×10^{-6}
					GWAS	0.40	0.96(0.93-1.00)	0.06
					iCOGS	0.40	0.98(0.96-1.00)	0.02
					OncoArray	0.40	0.97(0.95-0.99)	6.8×10^{-4}
rs142360995	8	118205719	A	G	Meta	0.20	1.03(1.02-1.05)	1.0×10^{-5}
					GWAS	0.21	1.04(1.00-1.09)	0.03
					iCOGS	0.20	1.03(1.01-1.06)	8.4×10^{-3}
					OncoArray	0.21	1.03(1.01-1.06)	4.1×10^{-3}
rs10820600	9	106856692	T	C	Meta	0.44	0.97(0.96-0.98)	1.8×10^{-7}
					GWAS	0.45	0.94(0.91-0.97)	5.0×10^{-4}
					iCOGS	0.45	0.97(0.95-0.99)	3.5×10^{-3}
					OncoArray	0.44	0.97(0.95-0.99)	1.9×10^{-3}
rs541079479	10	22861533	CA	C	Meta	0.42	1.03(1.02-1.05)	6.9×10^{-7}
					GWAS	0.41	1.09(1.05-1.14)	3.8×10^{-6}
					iCOGS	0.42	1.02(1.00-1.04)	0.09
					OncoArray	0.41	1.03(1.01-1.05)	6.4×10^{-4}
rs2901157	10	119262365	A	G	Meta	0.89	1.05(1.03-1.07)	2.3×10^{-6}
					GWAS	0.88	1.08(1.03-1.15)	4.8×10^{-3}
					iCOGS	0.89	1.04(1.01-1.08)	0.01
					OncoArray	0.89	1.05(1.02-1.07)	2.0×10^{-3}
rs10838267	11	44368892	A	G	Meta	0.54	1.03(1.02-1.05)	3.2×10^{-7}
					GWAS	0.53	1.04(1.01-1.08)	0.01
					iCOGS	0.54	1.03(1.01-1.05)	7.1×10^{-4}
					OncoArray	0.55	1.03(1.01-1.05)	2.5×10^{-3}
rs78588049	12	69180907	A	ATTTT	Meta	0.20	0.96(0.95-0.98)	3.3×10^{-6}
					GWAS	0.20	0.94(0.89-0.98)	7.4×10^{-3}

					iCOGS	0.20	0.97(0.95-1.00)	0.04
					OncoArray	0.20	0.96(0.94-0.98)	4.7×10^{-4}
rs855596	12	103045519	T	C	Meta	0.03	0.92(0.89-0.96)	1.9×10^{-5}
					GWAS	0.04	0.81(0.73-0.90)	1.6×10^{-4}
					iCOGS	0.03	0.97(0.92-1.02)	0.28
					OncoArray	0.03	0.90(0.85-0.96)	5.7×10^{-4}
rs9316500	13	51094114	T	G	Meta	0.71	1.03(1.02-1.05)	6.7×10^{-6}
					GWAS	0.71	1.03(0.99-1.07)	0.14
					iCOGS	0.71	1.04(1.02-1.06)	3.3×10^{-4}
					OncoArray	0.70	1.02(1.01-1.04)	0.01
rs75004998	14	77517786	A	G	Meta	0.33	0.97(0.96-0.98)	1.8×10^{-6}
					GWAS	0.33	0.97(0.93-1.00)	0.06
					iCOGS	0.33	0.97(0.95-0.99)	1.3×10^{-3}
					OncoArray	0.33	0.97(0.95-0.99)	2.5×10^{-3}
rs8027365	15	75808740	A	C	Meta	0.73	1.04(1.02-1.05)	9.7×10^{-8}
					GWAS	0.74	1.02(0.98-1.06)	0.32
					iCOGS	0.74	1.04(1.02-1.06)	5.6×10^{-4}
					OncoArray	0.72	1.04(1.02-1.06)	4.9×10^{-5}
rs76535198	16	71892498	A	C	Meta	0.86	1.04(1.03-1.06)	2.3×10^{-6}
					GWAS	0.87	1.05(1.00-1.11)	0.06
					iCOGS	0.87	1.05(1.02-1.08)	8.2×10^{-4}
					OncoArray	0.86	1.04(1.01-1.06)	4.6×10^{-3}
rs12481286	20	52287610	T	G	Meta	0.24	1.04(1.03-1.06)	1.0×10^{-7}
					GWAS	0.23	1.06(1.01-1.11)	0.01
					iCOGS	0.24	1.04(1.02-1.07)	3.6×10^{-4}
					OncoArray	0.24	1.04(1.01-1.06)	1.6×10^{-3}
rs35418111	21	47856670	A	G	Meta	0.07	1.06(1.04-1.09)	6.1×10^{-7}
					GWAS	0.08	1.11(1.04-1.18)	2.3×10^{-3}
					iCOGS	0.08	1.04(1.01-1.08)	0.02
					OncoArray	0.07	1.07(1.03-1.10)	3.3×10^{-4}
rs34331122	22	19762428	CTT	C	Meta	0.46	0.97(0.96-0.98)	7.2×10^{-6}
					GWAS	0.45	0.99(0.96-1.03)	0.73
					iCOGS	0.45	0.97(0.95-0.99)	3.6×10^{-3}
					OncoArray	0.46	0.97(0.95-0.98)	2.9×10^{-4}
