# Supplementary material for: Identification of novel breast cancer susceptibility loci in meta-analyses conducted among Asian and European descendants
Source: Nat Commun. 2020 Mar 5;11:1217. doi: 10.1038/s41467-020-15046-w (PMC7057957; doi:10.1038/s41467-020-15046-w)
Supplement: Supplementary file 6 — Supplementary Data 3 [file 41467_2020_15046_MOESM6_ESM.pdf]

Supplementary Data 3. Regulatory functional annotation of SNPs in high LD ($r^2$>0.8) with newly associated SNPs in Asian populations using Haploreg

| SNP | Chr | BP | LD $r^2$ | D' | Ref | Alt | AFR | AMR | ASN | EUR | GERP cons | SiPhy cons | Promoter (ENCODE) | Enhancer (ENCODE) | Promoter (Roadmap) | Enhancer (Roadmap) | DNAse | Gene | dbSNP functional annotation |
|---|---|---|---|---|---|---|---|---|---|---|---|---|---|---|---|---|---|---|---|
| **rs72906468** | **1** | **17772093** | - | - | A | T | 0.21 | 0.26 | 0.36 | 0.2 | 0 | 0 | . | . | . | . | . | *RCC2* | . |
| rs111631670 | 1 | 17772112 | 0.88 | 1 | A | T | 0.22 | 0.28 | 0.39 | 0.25 | 0 | 0 | . | . | . | . | . | *RCC2* | . |
| **rs3790585** | **1** | **46023356** | - | - | A | T | 0.21 | 0.22 | 0.31 | 0.15 | 0 | 0 | . | HMEC,5_Strong_Enhancer;NHEK,5_Strong_Enhancer;GM12878,4_Strong_Enhancer;Huvec,7_Weak_Enhancer | . | CD34.MBP1480,11_EnhWk1;ESO,14_Enh;PFK.3,11_EnhWk | AWG,GM12878,None;AWG,HSMMtube,None;AWG,HeLa-S3,None;AWG,LNCaP,None;AWG,NHEK,None;Duke,LNCaP,androgen;D | *AKR1A1* | Intronic |
| rs11211137 | 1 | 46013892 | 0.82 | 0.96 | T | A | 0.21 | 0.27 | 0.29 | 0.2 | 0 | 0 | . | . | . | . | . | *AKR1A1* | . |
| rs12077621 | 1 | 46015005 | 0.82 | 0.96 | G | A | 0.23 | 0.27 | 0.29 | 0.21 | 0 | 0 | . | . | . | SK.MUS,14_Enh | . | *AKR1A1* | . |
| rs11211139 | 1 | 46015751 | 0.82 | 0.96 | G | T | 0.2 | 0.27 | 0.29 | 0.21 | 0 | 0 | . | K562,7_Weak_Enhancer;GM12878,7_Weak_Enhancer;HepG2,7_Weak_Enhancer;Huvec,7_Weak_Enhancer;NHEK,6_Wea | BN.HM150,4_TssWk;BR.MYO,4_TssWk | SK.MUS,14_Enh;PFM.2,14_Enh;H1.BMP4DT,14_Enh;CD34. | . | *AKR1A1* | . |
| rs34768551 | 1 | 46020408 | 0.82 | 0.96 | G | A | 0.22 | 0.27 | 0.29 | 0.21 | 0 | 0 | . | . | . | ST.MUC,11_EnhWk1;CD34.C,9_TxEnhG1;H1.DMSC,9_TxE | . | *AKR1A1* | Intronic |
| rs666720 | 1 | 46020795 | 0.92 | -0.99 | T | C | 0.64 | 0.52 | 0.67 | 0.46 | 0 | 0 | . | . | . | CD34.C,9_TxEnhG1;CD34.MBP1480,11_EnhWk1;LIV.A,9 | . | *AKR1A1* | Intronic |
| rs12049503 | 1 | 46021556 | 0.83 | 0.96 | C | A | 0.24 | 0.27 | 0.29 | 0.2 | 0 | 0 | . | . | . | CD34.C,9_TxEnhG1;CD34.MBP1508,11_EnhWk1;CD34.MI | UW,CMK,None | *AKR1A1* | Intronic |
| rs512083 | 1 | 46027355 | 0.9 | -0.95 | T | C | 0.68 | 0.55 | 0.68 | 0.47 | 0 | 0 | . | . | . | DUO.MUC61,9_TxEnhG1 | . | *AKR1A1* | Intronic |
| rs514595 | 1 | 46036960 | 0.86 | -0.95 | T | C | 0.7 | 0.77 | 0.7 | 0.85 | 0 | 0 | . | . | . | SK.MUS,14_Enh | . | *AKR1A1* | . |
| rs499600 | 1 | 46039077 | 0.86 | -0.95 | T | G | 0.75 | 0.78 | 0.7 | 0.85 | 0 | 0 | . | H1,6_Weak_Enhancer;K562,4_Strong_Enhancer | . | HUES48,11_EnhWk1;HUES6,11_EnhWk1;IPS.15,11_EnhWk | AWG,H1-hESC,None;AWG,K562,None;UW,H7-hESC,None;UW,HAEpiC,None;UW,HCF,None;UW,HCPEpiC,None;UW,HMVEC-LL | *AKR1A1* | . |
| rs1084086 | 1 | 46041100 | 0.86 | -0.95 | T | G | 0.72 | 0.68 | 0.7 | 0.71 | 0 | 0 | . | K562,7_Weak_Enhancer | . | CD15.P,11_EnhWk1 | . | *AKR1A1* | . |
| rs3014213 | 1 | 46045522 | 0.86 | -0.95 | T | C | 0.73 | 0.68 | 0.7 | 0.71 | 0 | 0 | . | . | . | . | . | *NASP* | . |
| **rs2758598** | **1** | **156194339** | - | - | G | A | 0.18 | 0.28 | 0.15 | 0.35 | 0 | 0 | . | . | . | SK.MUS,9_TxEnhG1;H1.BMP4DM,14_Enh;BN.AG,14_Enh;l | . | *PMF1* | Intronic |
| rs6427304 | 1 | 156156789 | 0.84 | 0.92 | A | G | 0.71 | 0.32 | 0.15 | 0.36 | 0 | 0 | . | . | . | . | . | *SLC25A44* | . |
| rs11587860 | 1 | 156156951 | 0.84 | 0.92 | G | C | 0.67 | 0.32 | 0.15 | 0.36 | 0 | 0 | . | . | . | . | . | *SLC25A44* | . |
| rs2758615 | 1 | 156158367 | 0.84 | 0.94 | T | C | 0.7 | 0.32 | 0.15 | 0.35 | 0 | 0 | . | . | . | . | . | *SLC25A44* | . |
| rs2842864 | 1 | 156159217 | 0.87 | 0.94 | G | A | 0.67 | 0.32 | 0.15 | 0.35 | 0 | 0 | . | HepG2,7_Weak_Enhancer | . | . | . | *SLC25A44* | . |
| rs2842865 | 1 | 156159532 | 0.89 | 0.94 | G | A | 0.64 | 0.32 | 0.15 | 0.35 | 0 | 0 | . | HepG2,7_Weak_Enhancer | . | . | . | *SLC25A44* | . |
| rs2758616 | 1 | 156160358 | 0.87 | 0.94 | T | C | 0.67 | 0.32 | 0.15 | 0.35 | 0 | 0 | . | HepG2,7_Weak_Enhancer | . | . | . | *SLC25A44* | . |
| rs2842869 | 1 | 156160696 | 0.87 | 0.94 | A | G | 0.65 | 0.31 | 0.15 | 0.35 | 0 | 0 | . | HepG2,7_Weak_Enhancer | . | . | . | *SLC25A44* | . |
| rs2758618 | 1 | 156160892 | 0.87 | 0.94 | T | C | 0.64 | 0.31 | 0.15 | 0.35 | 0 | 0 | . | HepG2,7_Weak_Enhancer | . | . | . | *SLC25A44* | . |
| rs2758619 | 1 | 156162559 | 0.87 | 0.94 | A | G | 0.67 | 0.32 | 0.15 | 0.35 | 0 | 0 | . | K562,4_Strong_Enhancer;GM12878,7_Weak_Enhancer;Huvec,7_Weak_Enhancer | PFM.2,4_TssWk | CD19.P,14_Enh;CD34.C,11_EnhWk1;CD34.MBP1562,14_Er | . | *SLC25A44* | . |
| rs2540183 | 1 | 156164885 | 0.87 | 0.94 | C | G | 0.18 | 0.28 | 0.15 | 0.35 | 0 | 0 | GM12878,2_Weak_Promoter;K562 | NHLF,4_Strong_Enhancer;HepG2,6_Weak_Enhancer;NHEK,6_Weak_Enhancer | DUO.MUC61,2_TssF;SK.MUS62,6_TssD2;SK.MUS63,2_TssF;SK.MUS,2_TssF;CD15 | SPL,11_EnhWk1;H1.DMSC,11_EnhWk1;CCCRA.NP,11_Enh | . | *SLC25A44* | Intronic |
| rs3001790 | 1 | 156165290 | 0.87 | 0.94 | T | G | 0.67 | 0.32 | 0.15 | 0.35 | 0 | 0 | HSMM,2_Weak_Promoter;GM128 | HepG2,7_Weak_Enhancer;HMEC,7_Weak_Enhancer | HD.CD184EC,4_TssWk;H1.BMP4DM,4_TssWk;IPS.18,4_TssWk;LNG.FE,4_TssWk;M | H1.DMSC,11_EnhWk1;CCCRA.NP,11_EnhWk1;CCCRO.MI | Duke,Medullo,None | *SLC25A44* | Intronic |
| rs2984615 | 1 | 156165317 | 0.87 | 0.94 | G | C | 0.67 | 0.32 | 0.15 | 0.35 | 1 | 1 | HSMM,2_Weak_Promoter;GM128 | HepG2,7_Weak_Enhancer;HMEC,7_Weak_Enhancer | HD.CD184EC,4_TssWk;H1.BMP4DM,4_TssWk;IPS.18,4_TssWk;LNG.FE,4_TssWk;M | H1.DMSC,11_EnhWk1;CCCRA.NP,11_EnhWk1;CCCRO.MI | Duke,Medullo,None | *SLC25A44* | Intronic |
| rs2842882 | 1 | 156165342 | 0.87 | 0.94 | T | G | 0.67 | 0.32 | 0.15 | 0.35 | 1 | 1 | HSMM,2_Weak_Promoter;GM128 | HepG2,7_Weak_Enhancer;HMEC,7_Weak_Enhancer | HD.CD184EC,4_TssWk;H1.BMP4DM,4_TssWk;IPS.18,4_TssWk;LNG.FE,4_TssWk;M | H1.DMSC,11_EnhWk1;CCCRA.NP,11_EnhWk1;CCCRO.MI | Duke,Medullo,None | *SLC25A44* | Intronic |
| rs2842857 | 1 | 156168736 | 0.88 | 0.96 | T | C | 0.68 | 0.32 | 0.15 | 0.36 | 0 | 0 | . | GM12878,4_Strong_Enhancer;HepG2,5_Strong_Enhancer | . | HD.CD184EC,9_TxEnhG1;CD15.P,9_TxEnhG1;CD4.NP,11 | AWG,GM12878,None;AWG,Th1,None;UW,Adult_CD4_Th0,None;UW,GM12864,None;UW,GM12865,None;UW,Jurkat,None | *SLC25A44* | Intronic |
| rs2072499 | 1 | 156169610 | 0.89 | 0.97 | A | G | 0.67 | 0.33 | 0.15 | 0.36 | 0 | 0 | . | GM12878,4_Strong_Enhancer | . | CD15.P,9_TxEnhG1;CD4.NP,11_EnhWk1;BN.CC,9_TxEnhC | Duke,GM12891,None | *SLC25A44* | Intronic |
| rs2253677 | 1 | 156171296 | 0.88 | 0.96 | C | G | 0.67 | 0.32 | 0.15 | 0.35 | 0 | 0 | GM12878,2_Weak_Promoter | . | CD15.P,2_TssF | CD4.NP,11_EnhWk1;LIV.A,9_TxEnhG1;SK.MUS,9_TxEnhG | . | *SLC25A44* | Intronic |
| rs2253809 | 1 | 156172125 | 0.89 | 0.97 | T | C | 0.67 | 0.32 | 0.15 | 0.36 | 0 | 0 | GM12878,2_Weak_Promoter | . | CD15.P,2_TssF | LIV.A,9_TxEnhG1;CC.TPC,9_TxEnhG1;BN.GM2,9_TxEnhG | . | *SLC25A44* | Intronic |
| rs2266514 | 1 | 156173685 | 0.89 | 0.97 | T | C | 0.67 | 0.32 | 0.15 | 0.35 | 0 | 0 | . | Huvec,7_Weak_Enhancer | . | COL.SMUS,11_EnhWk1;ADI.NUC,11_EnhWk1;CD15.P,13 | . | *SLC25A44* | Intronic |
| rs2736605 | 1 | 156174475 | 0.89 | 0.97 | T | C | 0.64 | 0.32 | 0.15 | 0.35 | 0 | 0 | . | . | . | ADI.NUC,13_EnhA;MSC.ADIPC,13_EnhA;BN.HM150,9_Tx | . | *SLC25A44* | Intronic |
| rs200096331 | 1 | 156174831 | 0.82 | 0.94 | GA | G | 0.64 | 0.31 | 0.14 | 0.35 | 0 | 0 | . | . | . | MSC.ADIPC,13_EnhA;CD15.P,9_TxEnhG1;ADI.NUC,14_Er | . | *SLC25A44* | Intronic |
| rs2736606 | 1 | 156174837 | 0.86 | 0.96 | A | T | 0.55 | 0.3 | 0.16 | 0.34 | 0 | 0 | . | . | . | MSC.ADIPC,13_EnhA;CD15.P,9_TxEnhG1;ADI.NUC,14_Er | . | *SLC25A44* | Intronic |
| rs2241108 | 1 | 156181007 | 0.91 | 0.99 | C | G | 0.67 | 0.33 | 0.16 | 0.36 | 0 | 0 | . | . | . | IPS.DF19,14_Enh;PFF.2,9_TxEnhG1 | . | *SLC25A44* | 3'-UTR |
| rs2853641 | 1 | 156181770 | 0.91 | 0.99 | A | G | 0.67 | 0.32 | 0.16 | 0.36 | 0 | 0 | . | . | . | IPS.DF19,14_Enh;PFF.2,10_TxEnhG2;PFM.1,9_TxEnhG1;BN | . | *SLC25A44* | 3'-UTR |
| rs2241107 | 1 | 156182710 | 0.91 | 0.99 | T | C | 0.67 | 0.32 | 0.16 | 0.36 | 0 | 0 | NHLF,1_Active_Promoter;HSMM, | . | COL.MUC32,4_TssWk;BR.MYO,4_TssWk;PANC.I,4_TssWk;H9,3_TssA;BN.HM150, | SPL,14_Enh;H1.DMSC,14_Enh;ESO,14_Enh;H1.BMP4DT,14 | AWG,A549,None;AWG,GM12878,None;AWG,H1-hESC,None;AWG,HSMM,None;AWG,HSMMtube,None;AWG,HUVEC,None;AW | *PMF1* | . |
| rs1060604 | 1 | 156184268 | 0.91 | 0.99 | T | C | 0.19 | 0.29 | 0.16 | 0.35 | 0 | 0 | . | GM12878,4_Strong_Enhancer;K562,4_Strong_Enhancer;H1,6_Weak_Enhancer;HepG2,4_Strong_Enhancer;Huvec,5_Strong | CD34.MBP1562,2_TssF;SK.MUS62,6_TssD2;CHON.BMMSC,6_TssD2;LIV.A,2_TssF | H1.BMP4DM,14_Enh;BN.SN,13_EnhA;CD4.NP,11_EnhWk1 | AWG,A549,None;AWG,HMEC,None;AWG,HeLa-S3,None;AWG,HepG2,None;AWG,K562,None;AWG,MCF-7,None;AWG,NHEK,N | *PMF1* | Intronic |
| rs1137703 | 1 | 156184458 | 0.91 | 0.99 | T | C | 0.67 | 0.32 | 0.16 | 0.36 | 0 | 0 | . | K562,4_Strong_Enhancer;HMEC,5_Strong_Enhancer;NHEK,5_Strong_Enhancer;GM12878,5_Strong_Enhancer;HepG2,6_W | CD34.MBP1562,6_TssD2;PFF.2,4_TssWk;LIV.A,6_TssD2;SK.MUS,2_TssF | CD4.NP,11_EnhWk1;CD8.NP,11_EnhWk1;BR.H35,12_EnhW | UW,HRGEC,None | *PMF1* | Intronic |
| rs2244144 | 1 | 156184598 | 0.91 | 0.99 | G | A | 0.19 | 0.29 | 0.16 | 0.35 | 0 | 0 | . | HMEC,5_Strong_Enhancer;NHEK,5_Strong_Enhancer;K562,7_Weak_Enhancer | CD34.MBP1562,6_TssD2;PFF.2,4_TssWk;LIV.A,6_TssD2;SK.MUS,2_TssF | CD4.NP,11_EnhWk1;CD8.NP,11_EnhWk1;BR.H35,12_EnhW | . | *PMF1* | Intronic |
| rs2540175 | 1 | 156184747 | 0.92 | 0.96 | T | C | 0.61 | 0.32 | 0.15 | 0.36 | 0 | 0 | . | HMEC,5_Strong_Enhancer;NHEK,5_Strong_Enhancer;K562,7_Weak_Enhancer | . | CD8.NP,11_EnhWk1;PANC,11_EnhWk1;GAS,11_EnhWk1;E | . | *PMF1* | Intronic |
| rs2853643 | 1 | 156184831 | 0.91 | 0.99 | G | A | 0.19 | 0.29 | 0.16 | 0.35 | 0 | 0 | . | NHEK,5_Strong_Enhancer;K562,7_Weak_Enhancer;HMEC,7_Weak_Enhancer | . | CD8.NP,11_EnhWk1;PANC,11_EnhWk1;GAS,11_EnhWk1;C | . | *PMF1* | Intronic |
| rs7534434 | 1 | 156186615 | 0.9 | 0.99 | A | G | 0.63 | 0.31 | 0.16 | 0.35 | 0 | 0 | . | . | HRT.FE,2_TssF;LIV.A,1_TssP | CD3.P,10_TxEnhG2;H1.DNP,10_TxEnhG2;IPS.DF19,10_TxI | Duke,Chorion,None;Duke,T-47D,None | *PMF1* | Intronic |
| rs2475757 | 1 | 156186634 | 0.87 | 0.96 | C | G | 0.42 | 0.26 | 0.15 | 0.32 | 0 | 0 | . | . | HRT.FE,2_TssF;LIV.A,1_TssP | CD3.P,10_TxEnhG2;H1.DNP,10_TxEnhG2;IPS.DF19,10_TxI | . | *PMF1* | Intronic |
| rs2540173 | 1 | 156188214 | 0.93 | 1 | A | G | 0.67 | 0.32 | 0.16 | 0.36 | 0 | 0 | . | HMEC,5_Strong_Enhancer;NHEK,4_Strong_Enhancer;GM12878,5_Strong_Enhancer;Huvec,5_Strong_Enhancer | CD15.P,2_TssF;CD34.MBP1562,2_TssF;CD34.MBP1480,6_TssD2 | SPL,14_Enh;CCIP.LSMPTP,13_EnhA;CC.TPC,11_EnhWk1;( | AWG,HUVEC,None;AWG,Th1,None;Duke,8988T,None;Duke,GM19239,None;Duke,GM19240,None;Duke,Gliobla,None;UW,Monocy | *PMF1* | Intronic |
| rs2853646 | 1 | 156189626 | 0.92 | 0.99 | C | T | 0.19 | 0.28 | 0.15 | 0.35 | 0 | 0 | . | GM12878,7_Weak_Enhancer | . | SPL,14_Enh;CD34.MBP1480,11_EnhWk1;CD34.MBP1562,1 | . | *PMF1* | Intronic |
| rs7529564 | 1 | 156189793 | 0.91 | 1 | T | C | 0.62 | 0.31 | 0.16 | 0.35 | 0 | 0 | . | . | . | SPL,14_Enh;CD34.MBP1480,11_EnhWk1;CD34.MBP1562,1 | . | *PMF1* | Intronic |
| rs6427307 | 1 | 156190083 | 0.93 | 1 | A | G | 0.68 | 0.33 | 0.16 | 0.37 | 0 | 0 | . | . | . | CD34.MBP1562,14_Enh;CD34.MBP1536,9_TxEnhG1 | . | *PMF1* | Intronic |
| rs887953 | 1 | 156191149 | 0.93 | 1 | T | C | 0.46 | 0.3 | 0.16 | 0.36 | 0 | 0 | . | . | . | CD34.MBP1536,9_TxEnhG1;CD34.MBP1562,9_TxEnhG1;C | . | *PMF1* | Intronic |
| rs2247937 | 1 | 156194363 | 0.99 | 1 | C | T | 0.16 | 0.28 | 0.15 | 0.33 | 0 | 0 | . | . | . | SK.MUS,9_TxEnhG1;H1.BMP4DM,14_Enh;BN.AG,14_Enh;l | . | *PMF1* | Intronic |
| rs2758600 | 1 | 156195205 | 0.96 | 1 | C | T | 0.18 | 0.28 | 0.15 | 0.35 | 0 | 0 | . | GM12878,7_Weak_Enhancer | IMR90,4_TssWk | H1.BMP4DM,14_Enh;BN.AG,14_Enh;BN.CC,14_Enh;BN.SN | . | *PMF1* | Intronic |
| rs2248074 | 1 | 156195888 | 0.93 | 1 | C | T | 0.39 | 0.3 | 0.16 | 0.35 | 0 | 0 | . | GM12878,4_Strong_Enhancer;HSMM,7_Weak_Enhancer | . | BN.MFL,14_Enh;LIV.A,11_EnhWk1;CCC.TREGP,14_Enh;Cl | . | *PMF1* | Intronic |
| rs2248080 | 1 | 156195971 | 0.91 | 0.97 | T | A | 0.17 | 0.27 | 0.15 | 0.34 | 0 | 0 | . | GM12878,4_Strong_Enhancer;HSMM,7_Weak_Enhancer | . | BN.MFL,14_Enh;LIV.A,11_EnhWk1;CCC.TREGP,14_Enh;Cl | . | *PMF1* | Intronic |
| rs2736613 | 1 | 156196016 | 0.93 | 1 | T | C | 0.42 | 0.3 | 0.16 | 0.35 | 0 | 0 | . | HSMM,7_Weak_Enhancer;GM12878,5_Strong_Enhancer | . | BN.MFL,14_Enh;LIV.A,11_EnhWk1;CCC.TREGP,14_Enh;Cl | . | *PMF1* | Intronic |
| **rs6756513** | **2** | **70172587** | - | - | G | A | 0.32 | 0.18 | 0.28 | 0.29 | 0 | 0 | . | HMEC,5_Strong_Enhancer;NHEK,4_Strong_Enhancer;K562,5_Strong_Enhancer;Huvec,5_Strong_Enhancer | CD34.MBP1508,2_TssF;CD15.P,2_TssF;CD34.MBP1562,2_TssF | CD34.MBP1480,11_EnhWk1;CD34.P,13_EnhA;CD34.MBP1 | Duke,Osteobl,None;Duke,Stellate,None;UW,HCFaa,None | *MXD1* | . |
| **rs73006998** | **3** | **150464271** | - | - | G | A | 0.32 | 0.11 | 0.32 | 0.02 | 0 | 0 | . | . | . | CD4.MP,11_EnhWk1;GAS,11_EnhWk1;LIV.A,13_EnhA;ESC | . | *SIAH2* | Intronic |
| rs56725170 | 3 | 150450857 | 0.84 | 0.93 | C | T | 0.13 | 0.1 | 0.31 | 0.0026 | 0 | 0 | . | K562,5_Strong_Enhancer | . | . | . | *SIAH2* | . |
| rs939476 | 3 | 150451759 | 0.86 | -0.93 | T | A | 0.74 | 0.77 | 0.68 | 0.78 | 0 | 0 | . | K562,4_Strong_Enhancer;NHEK,5_Strong_Enhancer;HepG2,4_Strong_Enhancer;HMEC,7_Weak_Enhancer | . | NCC.COR2,11_EnhWk1;HRT.FE,11_EnhWk1;H1.BMP4DT, | AWG,HMEC,None;AWG,NHEK,None;Duke,pHTE,None;UW,HA-h,None;UW,HCF,None;UW,HMF,None;UW,HPAF,None;UW,SKMC | *SIAH2* | . |
| rs10513377 | 3 | 150452729 | 0.87 | 0.94 | C | T | 0.18 | 0.11 | 0.32 | 0.0026 | 0 | 0 | . | HepG2,4_Strong_Enhancer;NHEK,7_Weak_Enhancer;K562,7_Weak_Enhancer | . | NCC.COR2,11_EnhWk1;HRT.FE,11_EnhWk1;PFK.3,13_Enh | . | *SIAH2* | . |
| rs502941 | 3 | 150455755 | 0.8 | -0.98 | G | A | 0.62 | 0.85 | 0.64 | 0.87 | 0 | 0 | . | HepG2,4_Strong_Enhancer;NHLF,7_Weak_Enhancer;K562,4_Strong_Enhancer;GM12878,5_Strong_Enhancer;H1,7_Weak | . | ES.I3,11_EnhWk1;LIV.A,13_EnhA;DUO.MUC61,11_EnhWk | Duke,AoSMC,None;UW,HConF,None | *SIAH2* | . |
| rs594926 | 3 | 150456626 | 0.8 | -0.98 | C | T | 0.63 | 0.85 | 0.64 | 0.87 | 0 | 0 | . | HepG2,4_Strong_Enhancer;NHLF,7_Weak_Enhancer;K562,4_Strong_Enhancer;GM12878,5_Strong_Enhancer;NHEK,5_Str | CCIP.LSMPTP,2_TssF | ES.I3,11_EnhWk1;LIV.A,13_EnhA;GAS,11_EnhWk1;ADI.MS | UW,H7-hESC,None;UW,HFF,None;UW,HPF,None;UW,Jurkat,None;UW,WI-38,None | *SIAH2* | . |
| rs481236 | 3 | 150458543 | 0.8 | -0.98 | G | T | 0.49 | 0.83 | 0.64 | 0.87 | 0 | 0 | . | HepG2,4_Strong_Enhancer;K562,7_Weak_Enhancer;GM12878,5_Strong_Enhancer | . | LIV.A,13_EnhA;CD4.MP,11_EnhWk1;CCCRA.NP,11_EnhW | UW,HRPEpiC,None | *SIAH2* | . |
| rs6789306 | 3 | 150461224 | 0.82 | 0.99 | A | G | 0.36 | 0.12 | 0.36 | 0.02 | 0 | 0 | . | GM12878,5_Strong_Enhancer;NHEK,4_Strong_Enhancer;HMEC,4_Strong_Enhancer;HSMM,7_Weak_Enhancer | PFK.2,2_TssF;PFK.3,2_TssF | LIV.A,13_EnhA;CD4.MP,11_EnhWk1;CCIP.LSTP,14_Enh;LV | Duke,Fibrobl,None | *SIAH2* | Intronic |
| rs7636866 | 3 | 150461568 | 0.82 | 0.99 | A | T | 0.18 | 0.1 | 0.36 | 0.02 | 0 | 0 | . | GM12878,5_Strong_Enhancer;NHEK,4_Strong_Enhancer;Huvec,7_Weak_Enhancer;NHLF,6_Weak_Enhancer;HMEC,5_St | PFK.3,3_TssA | CD4.MP,11_EnhWk1;LV,14_Enh;GAS,11_EnhWk1;PANC,11 | AWG,HMEC,None;AWG,MCF-7,None;Duke,Gliobla,None;Duke,HPDE6-E6E7,None;Duke,HSMM_emb,None;Duke,Myometr,None;I | *SIAH2* | Intronic |
| rs7615125 | 3 | 150461900 | 0.99 | 1 | C | T | 0.32 | 0.11 | 0.32 | 0.02 | 0 | 0 | . | GM12878,5_Strong_Enhancer;NHEK,4_Strong_Enhancer;HMEC,5_Strong_Enhancer;HSMM,6_Weak_Enhancer;NHLF,7_W | . | CD4.MP,11_EnhWk1;GAS,11_EnhWk1;PFF.1,11_EnhWk1;B | . | *SIAH2* | Intronic |
| rs7615292 | 3 | 150461933 | 0.99 | 1 | G | A | 0.32 | 0.11 | 0.32 | 0.02 | 0 | 0 | . | GM12878,5_Strong_Enhancer;NHEK,4_Strong_Enhancer;HSMM,6_Weak_Enhancer;NHLF,7_Weak_Enhancer;HMEC,4_S | . | CD4.MP,11_EnhWk1;GAS,11_EnhWk1;PFF.1,11_EnhWk1;B | UW,WERI-Rb-1,None | *SIAH2* | Intronic |
| rs73006999 | 3 | 150465010 | 0.81 | 0.99 | G | A | 0.36 | 0.12 | 0.36 | 0.02 | 1 | 0 | . | . | . | GAS,11_EnhWk1;LIV.A,13_EnhA;ESO,14_Enh;CCIP.LSTP,1 | Duke,FibroP,None;UW,HFF,None;UW,HRE,None | *SIAH2* | Intronic |
| rs73008903 | 3 | 150465053 | 0.81 | 0.99 | C | T | 0.36 | 0.12 | 0.36 | 0.02 | 0 | 0 | . | . | . | GAS,11_EnhWk1;LIV.A,13_EnhA;ESO,14_Enh;CCIP.LSTP,1 | Duke,FibroP,None;UW,HFF,None;UW,HRE,None | *SIAH2* | Intronic |
| rs73008905 | 3 | 150465435 | 0.81 | 0.99 | T | C | 0.35 | 0.12 | 0.36 | 0.02 | 0 | 0 | . | . | . | GAS,11_EnhWk1;LIV.A,13_EnhA;CCIP.LSTP,11_EnhWk1;R | AWG,MCF-7,None | *SIAH2* | Intronic |
| rs73008907 | 3 | 150465456 | 0.81 | 0.99 | G | A | 0.36 | 0.11 | 0.36 | 0.02 | 0 | 0 | . | . | . | GAS,11_EnhWk1;LIV.A,13_EnhA;CCIP.LSTP,11_EnhWk1;R | . | *SIAH2* | Intronic |
| rs55899470 | 3 | 150465739 | 0.81 | 0.99 | G | A | 0.35 | 0.12 | 0.36 | 0.02 | 0 | 0 | . | . | . | GAS,11_EnhWk1;LIV.A,13_EnhA;CCIP.LSTP,11_EnhWk1;R | . | *SIAH2* | Intronic |
| rs57824095 | 3 | 150465887 | 0.81 | 0.99 | G | A | 0.35 | 0.12 | 0.36 | 0.02 | 0 | 0 | . | . | . | GAS,11_EnhWk1;LIV.A,13_EnhA;CCIP.LSTP,11_EnhWk1;R | AWG,HSMM,None;UW,AG04449,None;UW,AoAF,None;UW,HAEpiC,None;UW,HCF,None;UW,HCFaa,None;UW,HEEpiC,None;UW | *SIAH2* | Intronic |
| rs1879420 | 3 | 150466564 | 0.98 | 0.99 | T | C | 0.1 | 0.09 | 0.32 | 0.02 | 0 | 0 | . | HSMM,7_Weak_Enhancer | . | GAS,11_EnhWk1;LIV.A,13_EnhA;CCIP.LSTP,11_EnhWk1;R | UW,AG04449,None;UW,BJ,None;UW,HA-h,None;UW,HCFaa,None;UW,HCPEpiC,None;UW,HIPEpiC,None;UW,HNPCEpiC,None;U | *SIAH2* | Intronic |
| rs1879421 | 3 | 150466575 | 0.81 | 0.99 | G | A | 0.53 | 0.14 | 0.36 | 0.02 | 0 | 0 | . | HSMM,7_Weak_Enhancer | . | GAS,11_EnhWk1;LIV.A,13_EnhA;CCIP.LSTP,11_EnhWk1;R | UW,AG04449,None;UW,BJ,None;UW,HA-h,None;UW,HCFaa,None;UW,HCPEpiC,None;UW,HIPEpiC,None;UW,HNPCEpiC,None;U | *SIAH2* | Intronic |
| rs1879422 | 3 | 150466940 | 0.81 | 0.99 | G | C | 0.47 | 0.13 | 0.36 | 0.02 | 0 | 0 | . | HSMM,7_Weak_Enhancer;HepG2,5_Strong_Enhancer | . | GAS,11_EnhWk1;LIV.A,13_EnhA;R.MUC29,11_EnhWk1;ST | . | *SIAH2* | Intronic |
| rs6788895 | 3 | 150467808 | 0.81 | 0.99 | G | T | 0.47 | 0.14 | 0.36 | 0.02 | 0 | 0 | . | . | DUO.MUC61,6_TssD2 | LIV.A,13_EnhA;R.MUC29,11_EnhWk1;ST.MUC,11_EnhWk | . | *SIAH2* | Intronic |
| rs1991781 | 3 | 150468433 | 0.81 | 0.99 | G | A | 0.32 | 0.12 | 0.36 | 0.02 | 0 | 0 | . | NHEK,7_Weak_Enhancer | . | LIV.A,13_EnhA;R.MUC29,11_EnhWk1;ST.MUC,11_EnhWk | . | *SIAH2* | Intronic |
| rs73010930 | 3 | 150469735 | 0.81 | 0.99 | G | A | 0.49 | 0.14 | 0.36 | 0.02 | 1 | 0 | . | HMEC,7_Weak_Enhancer;H1,7_Weak_Enhancer;NHLF,7_Weak_Enhancer;NHEK,5_Strong_Enhancer | R.MUC29,6_TssD2 | LIV.A,13_EnhA;PFK.3,9_TxEnhG1;CD4.MP,11_EnhWk1;CC | Duke,Ishikawa,Estradiol_100nM_1hr;Duke,MCF-7,Hypoxia_LacAcid;Duke,pHTE,None | *SIAH2* | Intronic |
| rs16862837 | 3 | 150469858 | 0.81 | 0.99 | G | T | 0.49 | 0.14 | 0.36 | 0.02 | 1 | 0 | . | HMEC,7_Weak_Enhancer;H1,7_Weak_Enhancer;NHLF,7_Weak_Enhancer;NHEK,5_Strong_Enhancer | R.MUC29,6_TssD2 | LIV.A,13_EnhA;PFK.3,9_TxEnhG1;CD4.MP,11_EnhWk1;CC | AWG,LNCaP,None;AWG,MCF-7,None;Duke,HPDE6-E6E7,None;Duke,LNCaP,androgen;Duke,T-47D,None;Duke,pHTE,None;UW,C | *SIAH2* | Intronic |
| rs1533711 | 3 | 150469996 | 0.81 | 0.99 | A | G | 0.49 | 0.14 | 0.36 | 0.02 | 0 | 0 | . | HMEC,7_Weak_Enhancer;NHEK,5_Strong_Enhancer | R.MUC29,6_TssD2 | LIV.A,13_EnhA;PFK.3,9_TxEnhG1;CD4.MP,11_EnhWk1;CC | AWG,HepG2,None;AWG,NHEK,None;Duke,Ishikawa,4OHTAM_20nM_72hr;Duke,Ishikawa,Estradiol_100nM_1hr;UW,GM12864,No | *SIAH2* | Intronic |
| rs2116684 | 3 | 150471737 | 0.92 | 0.98 | G | C | 0.23 | 0.1 | 0.33 | 0.02 | 0 | 0 | . | . | . | CCIP.LSMPTP,9_TxEnhG1;CC.TPC,11_EnhWk1;ST.MUC,1 | . | *SIAH2* | Intronic |
| rs57359526 | 3 | 150472042 | 0.92 | 0.98 | T | C | 0.23 | 0.1 | 0.33 | 0.02 | 0 | 0 | . | . | . | CC.TPC,11_EnhWk1;CCIP.LSTP,11_EnhWk1;PFK.2,9_TxEr | . | *SIAH2* | Intronic |
| rs61628582 | 3 | 150472160 | 0.9 | 0.98 | C | T | 0.2 | 0.1 | 0.33 | 0.02 | 0 | 0 | . | K562,7_Weak_Enhancer | . | CC.TPC,11_EnhWk1;CCIP.LSTP,11_EnhWk1;PFK.2,9_TxEr | UW,Jurkat,None | *SIAH2* | Intronic |
| rs2120596 | 3 | 150472390 | 0.9 | 0.98 | G | A | 0.14 | 0.1 | 0.33 | 0.02 | 0 | 0 | . | K562,7_Weak_Enhancer | HD.CD184EC,6_TssD2 | CC.TPC,11_EnhWk1;CCIP.LSTP,11_EnhWk1;PFK.2,9_TxEr | . | *SIAH2* | Intronic |
| rs60092981 | 3 | 150472999 | 0.9 | 0.98 | T | A | 0.14 | 0.1 | 0.33 | 0.02 | 0 | 0 | . | . | HD.CD184EC,6_TssD2 | CCIP.LSTP,11_EnhWk1;GAS,11_EnhWk1;R.MUC31,14_Enl | Duke,GM19238,None;Duke,Osteobl,None;Duke,iPS,None | *SIAH2* | Intronic |
| rs79220446 | 3 | 150474658 | 0.89 | 0.98 | T | C | 0.14 | 0.1 | 0.34 | 0.02 | 0 | 0 | . | GM12878,6_Weak_Enhancer;HepG2,5_Strong_Enhancer | R.MUC29,6_TssD2 | ADI.NUC,13_EnhA;BM.MSC,13_EnhA;R.MUC31,13_EnhA; | AWG,LNCaP,None;Duke,LNCaP,androgen | *SIAH2* | Intronic |
| rs60523415 | 3 | 150477002 | 0.81 | 0.94 | C | G | 0.16 | 0.1 | 0.34 | 0.02 | 0 | 0 | GM12878,2_Weak_Promoter | NHEK,5_Strong_Enhancer;HMEC,7_Weak_Enhancer | CCIP.LSMPTP,2_TssF;CCIP.LSTP,3_TssA | HRT.FE,11_EnhWk1;CCC.TREGP,11_EnhWk1;ESO,14_Enh | AWG,NHEK,None | *SIAH2* | Intronic |
| rs58418105 | 3 | 150477046 | 0.81 | 0.94 | C | A | 0.04 | 0.09 | 0.34 | 0.02 | 0 | 0 | GM12878,2_Weak_Promoter | NHEK,5_Strong_Enhancer;HMEC,7_Weak_Enhancer | CCIP.LSMPTP,2_TssF;CCIP.LSTP,3_TssA | HRT.FE,11_EnhWk1;CCC.TREGP,11_EnhWk1;ESO,14_Enh | AWG,NHEK,None | *SIAH2* | Intronic |
| rs75942495 | 3 | 150477877 | 0.81 | 0.94 | G | A | 0.16 | 0.1 | 0.34 | 0.02 | 0 | 0 | GM12878,1_Active_Promoter;Huv | HepG2,4_Strong_Enhancer;NHLF,6_Weak_Enhancer;HMEC,6_Weak_Enhancer;K562,4_Strong_Enhancer;NHEK,4_Strong | MSC.ADIPC,4_TssWk;CD19.P,2_TssF;PFK.3,2_TssF;BN.MFL,6_TssD2;CD4.MP,2_T | CCC.TMP,14_Enh;H9,11_EnhWk1;IMR90,9_TxEnhG1;BN.F | AWG,GM12878,None;Duke,H9ES,None;Duke,Hepatocytes,None;Duke,T-47D,None;UW,HVMF,None | *SIAH2* | Intronic |
| rs74950732 | 3 | 150482621 | 0.8 | 0.94 | C | T | 0.16 | 0.1 | 0.34 | 0.02 | 0 | 0 | H1,3_Poised_Promoter;GM12878,2 | HMEC,6_Weak_Enhancer;HSMM,6_Weak_Enhancer;Huvec,6_Weak_Enhancer;HepG2,4_Strong_Enhancer;NHEK,6_Weal | CD34.C,2_TssF;BN.FE0,4_TssWk;CD34.MBP1549,4_TssWk;R.MUC29,6_TssD2;CCI | CCC.TMP,14_Enh;LV,14_Enh;CCCRO.MP,13_EnhA;ST.MU | AWG,A549,None;UW,Adult_CD4_Th0,None;UW,CD34+_Mobilized,None | *SIAH2* | . |
| rs76801042 | 3 | 150482720 | 0.8 | 0.94 | A | C | 0.16 | 0.1 | 0.34 | 0.02 | 0 | 0 | GM12878,2_Weak_Promoter | HepG2,4_Strong_Enhancer;K562,4_Strong_Enhancer | CD34.C,2_TssF;BN.FE0,4_TssWk;CD34.MBP1549,4_TssWk;R.MUC29,6_TssD2;CCI | CCC.TMP,14_Enh;LV,14_Enh;CCCRO.MP,13_EnhA;ST.MU | . | *SIAH2* | . |
| rs77577446 | 3 | 150486765 | 0.8 | 0.94 | G | A | 0.11 | 0.1 | 0.34 | 0.02 | 0 | 0 | . | . | . | . | . | *SIAH2* | . |
| **rs11281251** | **3** | **156519412** | - | - | T | TTGTGAC | 0.34 | 0.69 | 0.82 | 0.58 | 0 | 0 | . | . | . | PFK.3,9_TxEnhG1;BR.H35,11_EnhWk1;BR.MYO,11_EnhWl | . | *LOC730091* | Intronic |
| rs199589043 | 3 | 156518663 | 0.85 | 0.97 | G | GGT | 0.37 | 0.7 | 0.84 | 0.6 | 0 | 0 | . | . | . | PFK.3,9_TxEnhG1 | Duke,Fibrobl,None | *LOC730091* | Intronic |
| rs1835372 | 3 | 156519027 | 0.86 | 0.97 | C | T | 0.36 | 0.7 | 0.84 | 0.61 | 0 | 0 | . | K562,7_Weak_Enhancer | . | PFK.3,9_TxEnhG1;BR.H35,11_EnhWk1 | . | *LOC730091* | Intronic |
| rs1430411 | 3 | 156525396 | 0.86 | 0.97 | T | A | 0.36 | 0.7 | 0.84 | 0.61 | 0 | 0 | . | K562,7_Weak_Enhancer;NHEK,7_Weak_Enhancer;HMEC,5_Strong_Enhancer | . | BM.MSC,14_Enh;PFM.3,11_EnhWk1;PFF.1,14_Enh;MUS.SC | . | *LOC730091* | Intronic |
| rs1430410 | 3 | 156525562 | 0.86 | 0.97 | C | A | 0.36 | 0.7 | 0.84 | 0.61 | 0 | 0 | . | K562,7_Weak_Enhancer;NHEK,7_Weak_Enhancer;HMEC,5_Strong_Enhancer | . | PFF.1,14_Enh;HUES48,12_EnhWk2;ADI.MSC,13_EnhA;PFK | . | *LOC730091* | Intronic |
| rs10936037 | 3 | 156528265 | 0.82 | 0.91 | C | T | 0.33 | 0.64 | 0.83 | 0.52 | 1 | 0 | . | NHEK,5_Strong_Enhancer;HMEC,5_Strong_Enhancer | . | ADI.MSC,14_Enh;PFF.1,14_Enh;PFK.3,14_Enh;BR.MYO,11 | . | *PA2G4P4* | Intronic |
| rs9812169 | 3 | 156528401 | 0.86 | 0.97 | G | A | 0.36 | 0.7 | 0.84 | 0.61 | 1 | 0 | . | HMEC,5_Strong_Enhancer;HepG2,6_Weak_Enhancer;Huvec,7_Weak_Enhancer;K562,6_Weak_Enhancer;NHEK,4_Strong | . | ADI.MSC,14_Enh;BR.MYO,11_EnhWk1;PFK.2,13_EnhA;GA | . | *PA2G4P4* | Intronic |
| rs7641929 | 3 | 156529394 | 0.86 | 0.97 | C | G | 0.36 | 0.7 | 0.84 | 0.61 | 1 | 1 | . | HepG2,7_Weak_Enhancer;Huvec,5_Strong_Enhancer;HMEC,5_Strong_Enhancer;K562,7_Weak_Enhancer;NHEK,4_Strong | . | BR.MYO,11_EnhWk1;H1.BMP4DT,14_Enh;DUO.MUC61,11 | AWG,A549,None;AWG,HMEC,None;Duke,AoSMC,None;Duke,Fibrobl,None;Duke,HPDE6-E6E7,None;Duke,MCF-7,Hypoxia_LacA | *PA2G4P4* | Intronic |
| rs12492804 | 3 | 156532145 | 0.86 | 0.97 | T | A | 0.36 | 0.7 | 0.84 | 0.61 | 0 | 0 | . | NHEK,5_Strong_Enhancer;GM12878,6_Weak_Enhancer;HMEC,5_Strong_Enhancer | . | BR.MYO,11_EnhWk1;BR.H35,11_EnhWk1;PFK.2,13_EnhA;l | AWG,HeLa-S3,None | *LOC730091* | Intronic |
| rs13086516 | 3 | 156532550 | 0.85 | 0.95 | C | T | 0.35 | 0.68 | 0.83 | 0.55 | 0 | 0 | . | NHEK,5_Strong_Enhancer;GM12878,6_Weak_Enhancer;HMEC,5_Strong_Enhancer | . | BR.MYO,11_EnhWk1;PFK.2,13_EnhA;ESO,14_Enh;PFF.1,1 | . | *LOC730091* | Intronic |
| rs10936038 | 3 | 156532666 | 0.86 | 0.97 | C | T | 0.35 | 0.68 | 0.84 | 0.55 | 0 | 0 | . | NHEK,5_Strong_Enhancer;GM12878,6_Weak_Enhancer;HMEC,5_Strong_Enhancer | . | ESO,14_Enh;PFF.1,14_Enh;MUS.SC,14_Enh;PFK.3,13_EnhA | UW,Th2,None | *LOC730091* | Intronic |
| rs11711674 | 3 | 156532953 | 0.84 | 0.95 | C | T | 0.35 | 0.67 | 0.83 | 0.56 | 0 | 0 | . | NHEK,5_Strong_Enhancer;GM12878,6_Weak_Enhancer;HMEC,7_Weak_Enhancer | . | ESO,14_Enh;PFF.1,14_Enh;PFK.2,14_Enh;LIV.A,14_Enh;IMF | . | *LOC730091* | Intronic |
| rs4680305 | 3 | 156533403 | 0.85 | 0.96 | G | C | 0.31 | 0.67 | 0.83 | 0.56 | 1 | 1 | GM12878,1_Active_Promoter | NHLF,7_Weak_Enhancer;HMEC,5_Strong_Enhancer;NHEK,4_Strong_Enhancer;Huvec,5_Strong_Enhancer | PANC.I,4_TssWk;PFF.1,3_TssA;DUO.MUC61,6_TssD2;PFK.3,3_TssA;R.MUC29,4_T | ESO,14_Enh;IMR90,14_Enh;CCCRA.NP,11_EnhWk1;BN.FE | AWG,A549,None;AWG,GM12878,None;AWG,NHEK,None;UW,HMVEC-dLy-Ad,None;UW,HPdLF,None | *LOC730091* | Intronic |
| rs59717073 | 3 | 156534245 | 0.85 | 0.96 | A | G | 0.36 | 0.7 | 0.83 | 0.61 | 0 | 0 | GM12878,1_Active_Promoter;NHL | Huvec,4_Strong_Enhancer;HMEC,4_Strong_Enhancer | PANC.I,4_TssWk;IPS.15,1_TssP;PFK.2,2_TssF;MUS.SC,2_TssF;ADI.MSC,3_TssA;IP | CD34.P,12_EnhWk2;CCCRO.MP,14_Enh;CCC.TMP,14_Enh | AWG,H1-hESC,None;Duke,GM18507,None;Duke,GM19238,None;Duke,Huh-7,None;Duke,MCF-7,Hypoxia_LacAcid;Duke,Stellate,N | *LOC730091* | Intronic |
| rs11717133 | 3 | 156534581 | 0.85 | 0.96 | T | C | 0.36 | 0.7 | 0.83 | 0.61 | 0 | 0 | GM12878,1_Active_Promoter;NHL | Huvec,4_Strong_Enhancer;HMEC,4_Strong_Enhancer | PANC.I,4_TssWk;PFK.2,2_TssF;MUS.SC,2_TssF;ADI.MSC,3_TssA;IPS.18,1_TssP;IP | CCCRO.MP,14_Enh;CCC.TMP,14_Enh;CD8.MP,14_Enh;HR | AWG,HUVEC,None;Duke,GM12892,None;Duke,GM19238,None;Duke,GM19239,None;Duke,GM19240,None;Duke,HSMM_emb,No | *LOC730091* | Intronic |
| rs1560415 | 3 | 156537358 | 0.85 | 0.96 | T | C | 0.35 | 0.67 | 0.83 | 0.55 | 1 | 0 | . | Huvec,4_Strong_Enhancer;NHLF,5_Strong_Enhancer;K562,7_Weak_Enhancer;HMEC,5_Strong_Enhancer;NHEK,5_Strong | . | PFK.3,13_EnhA;ADI.MSC,13_EnhA;ST.MUC,14_Enh;CD34 | . | *LOC730091* | . |
| rs1560416 | 3 | 156537419 | 0.83 | 0.96 | A | G | 0.35 | 0.68 | 0.84 | 0.55 | 1 | 0 | . | Huvec,4_Strong_Enhancer;NHLF,5_Strong_Enhancer;K562,7_Weak_Enhancer;HMEC,5_Strong_Enhancer;NHEK,5_Strong | . | PFK.3,13_EnhA;ST.MUC,14_Enh;CD34.C,11_EnhWk1;PFF. | . | *LOC730091* | . |
| rs63423161 | 3 | 156538670 | 0.85 | 0.96 | A | G | 0.36 | 0.7 | 0.83 | 0.61 | 0 | 0 | . | HSMM,5_Strong_Enhancer;Huvec,5_Strong_Enhancer;GM12878,5_Strong_Enhancer;HMEC,7_Weak_Enhancer;NHEK,7_W | . | PFF.2,14_Enh;MUS.SC,13_EnhA;CHON.BMMSC,14_Enh;PF | AWG,HUVEC,None;Duke,Chorion,None;Duke,GM19238,None;Duke,Hepatocytes,None;Duke,LNCaP,androgen;Duke,Medullo,None;I | *LOC730091* | . |
| rs9837271 | 3 | 156538738 | 0.85 | 0.96 | C | T | 0.35 | 0.68 | 0.83 | 0.55 | 0 | 0 | . | HSMM,5_Strong_Enhancer;Huvec,5_Strong_Enhancer;GM12878,5_Strong_Enhancer;HMEC,7_Weak_Enhancer | . | PFF.2,14_Enh;MUS.SC,13_EnhA;CHON.BMMSC,14_Enh;PF | AWG,A549,None;AWG,HUVEC,None;AWG,Th1,None;Duke,LNCaP,androgen;Duke,Myometr,None;UW,Adult_CD4_Th0,None;UW,( | *LOC730091* | . |
| rs9842198 | 3 | 156539204 | 0.85 | 0.96 | G | C | 0.36 | 0.7 | 0.83 | 0.6 | 0 | 0 | . | GM12878,7_Weak_Enhancer | . | PFF.2,14_Enh;MUS.SC,14_Enh | Duke,8988T,None | *LOC730091* | . |
| rs13097325 | 3 | 156539545 | 0.85 | 0.96 | G | T | 0.35 | 0.68 | 0.83 | 0.55 | 0 | 0 | . | . | . | PFF.2,14_Enh;MUS.SC,14_Enh | . | *LEKR1* | . |
| **rs11944638** | **4** | **48227719** | - | - | T | C | 0.14 | 0.12 | 0.24 | 0.05 | 0 | 0 | . | H1,7_Weak_Enhancer | . | CD15.P,13_EnhA;IPS.DF19,14_Enh;HUES48,12_EnhWk2;H1 | . | *TEC* | Intronic |
| rs6829390 | 4 | 48198986 | 0.85 | 0.95 | G | A | 0.45 | 0.16 | 0.26 | 0.06 | 1 | 0 | GM12878,1_Active_Promoter | K562,5_Strong_Enhancer | . | CD34.MBP1536,11_EnhWk1;PFF.1,11_EnhWk1;CD34.C,9 | AWG,GM12878,None;Duke,CLL,None;Duke,GM12891,None;Duke,GM18507,None;Duke,GM19238,None;Duke,GM19239,None;Duk | *TEC* | Intronic |
| rs73814690 | 4 | 48203463 | 0.92 | 0.96 | A | G | 0.12 | 0.12 | 0.24 | 0.06 | 0 | 0 | . | GM12878,5_Strong_Enhancer;K562,5_Strong_Enhancer | . | CD34.C,9_TxEnhG1;CD34.MBP1480,11_EnhWk1;CD34.MI | . | *TEC* | Intronic |
| rs73138446 | 4 | 48204780 | 0.92 | 0.96 | T | C | 0.12 | 0.12 | 0.24 | 0.05 | 0 | 0 | . | K562,7_Weak_Enhancer;GM12878,4_Strong_Enhancer | . | CD34.MBP1480,11_EnhWk1;CD34.MBP1562,13_EnhA;CD | Duke,Osteobl,None | *TEC* | Intronic |
| rs11942525 | 4 | 48205942 | 0.92 | 0.96 | C | T | 0.12 | 0.12 | 0.24 | 0.05 | 0 | 0 | . | GM12878,4_Strong_Enhancer;K562,4_Strong_Enhancer | . | CD34.MBP1480,11_EnhWk1;CD34.MBP1562,13_EnhA;CD | AWG,HSMMtube,None;AWG,K562,None;UW,CD34+_Mobilized,None;UW,CMK,None | *TEC* | Intronic |
| rs73138447 | 4 | 48206396 | 0.92 | 0.96 | C | T | 0.12 | 0.12 | 0.24 | 0.05 | 0 | 0 | . | GM12878,4_Strong_Enhancer;K562,4_Strong_Enhancer | . | CD34.MBP1562,13_EnhA;CD34.C,13_EnhA;CD19.P,11_Enl | . | *TEC* | Intronic |
| rs73138450 | 4 | 48206765 | 0.92 | 0.96 | C | T | 0.12 | 0.12 | 0.24 | 0.05 | 0 | 0 | . | GM12878,4_Strong_Enhancer;K562,4_Strong_Enhancer | . | CD34.MBP1562,13_EnhA;CD34.C,13_EnhA;HRT.FE,11_En | . | *TEC* | Intronic |
| rs66944352 | 4 | 48207199 | 0.92 | 0.96 | C | T | 0.12 | 0.14 | 0.24 | 0.09 | 0 | 0 | . | GM12878,4_Strong_Enhancer;K562,5_Strong_Enhancer | . | CD34.MBP1562,13_EnhA;CD34.C,13_EnhA;CD34.MBP153 | UW,GM12865,None | *TEC* | Intronic |
| rs2136504 | 4 | 48211923 | 0.92 | 0.96 | A | G | 0.12 | 0.12 | 0.24 | 0.05 | 0 | 0 | . | GM12878,5_Strong_Enhancer | . | CD34.MBP1480,11_EnhWk1;CD34.MBP1508,11_EnhWk1;C | . | *TEC* | Intronic |
| rs1159075 | 4 | 48215854 | 0.92 | 0.96 | C | T | 0.12 | 0.12 | 0.24 | 0.05 | 0 | 0 | . | GM12878,5_Strong_Enhancer | . | CD34.MBP1480,11_EnhWk1;CD34.MBP1562,13_EnhA;CD | AWG,HSMMtube,None | *TEC* | Intronic |
| rs145795515 | 4 | 48217094 | 0.92 | 0.96 | C | T | 0.12 | 0.12 | 0.24 | 0.05 | 0 | 0 | . | . | . | CD34.C,13_EnhA;CD34.MBP1562,14_Enh | . | *TEC* | Intronic |
| rs73138478 | 4 | 48220377 | 0.92 | 0.96 | T | C | 0.12 | 0.12 | 0.24 | 0.05 | 0 | 0 | . | . | . | . | . | *TEC* | Intronic |
| rs11722540 | 4 | 48221182 | 0.92 | 0.96 | C | T | 0.12 | 0.12 | 0.24 | 0.05 | 0 | 0 | . | K562,7_Weak_Enhancer | . | . | . | *TEC* | Intronic |
| rs36067666 | 4 | 48224710 | 0.93 | 0.96 | GC | G | 0.12 | 0.12 | 0.24 | 0.05 | 0 | 0 | . | K562,7_Weak_Enhancer | . | BR.H35,11_EnhWk1;CD15.P,14_Enh | UW,HRCEpiC,None;UW,HRE,None;UW,RPTEC,None | *TEC* | Intronic |
| rs34585972 | 4 | 48224717 | 0.93 | 0.96 | C | T | 0.12 | 0.12 | 0.24 | 0.05 | 0 | 0 | . | K562,7_Weak_Enhancer | . | BR.H35,11_EnhWk1;CD15.P,14_Enh | UW,HRCEpiC,None;UW,HRE,None;UW,RPTEC,None | *TEC* | Intronic |
| **rs11947923** | **4** | **53911337** | - | - | C | T | 0.06 | 0.36 | 0.29 | 0.4 | 0 | 0 | . | . | . | BN.FE1,12_EnhWk2 | . | *SCFD2* | Intronic |
| rs5858201 | 4 | 53884062 | 0.85 | 0.93 | CG | C | 0.03 | 0.36 | 0.28 | 0.36 | 0 | 0 | . | . | . | KID.FE,11_EnhWk1 | . | *SCFD2* | Intronic |
| rs12506630 | 4 | 53912593 | 1 | 1 | G | C | 0.04 | 0.36 | 0.29 | 0.4 | 0 | 0 | . | . | . | . | . | *SCFD2* | Intronic |
| rs17082328 | 4 | 53912794 | 1 | 1 | T | C | 0.04 | 0.37 | 0.29 | 0.4 | 0 | 0 | . | . | . | . | . | *SCFD2* | Intronic |
| **rs6555134** | **5** | **2776483** | - | - | T | C | 0.52 | 0.49 | 0.74 | 0.42 | 0 | 0 | . | . | . | . | . | *C5orf38* | . |
| rs4866493 | 5 | 2776719 | 1 | 1 | G | C | 0.52 | 0.49 | 0.74 | 0.42 | 0 | 0 | . | . | . | . | UW,Jurkat,None | *C5orf38* | . |
| rs4866496 | 5 | 2777029 | 1 | 1 | G | A | 0.52 | 0.49 | 0.74 | 0.42 | 0 | 0 | . | . | . | . | . | *C5orf38* | . |
| rs7734695 | 5 | 2777159 | 1 | 1 | C | T | 0.52 | 0.49 | 0.74 | 0.42 | 0 | 0 | . | . | . | . | Duke,Fibrobl,None | *C5orf38* | . |
| rs1497465 | 5 | 2777249 | 1 | 1 | C | T | 0.5 | 0.49 | 0.74 | 0.42 | 0 | 0 | . | . | . | H1.BMP4DT,11_EnhWk1 | . | *C5orf38* | . |
| rs7709328 | 5 | 2777311 | 0.96 | 1 | G | A | 0.51 | 0.49 | 0.74 | 0.42 | 0 | 0 | . | . | . | H1.BMP4DT,11_EnhWk1 | . | *C5orf38* | . |
| rs6869002 | 5 | 2777536 | 0.84 | -0.99 | G | A | 0.33 | 0.25 | 0.23 | 0.25 | 0 | 0 | . | . | . | . | . | *C5orf38* | . |
| **rs7765429** | **6** | **21904169** | - | - | T | C | 0.68 | 0.38 | 0.1 | 0.51 | 0 | 0 | . | . | . | HD.CD184EC,11_EnhWk1 | . | *FLJ22536* | Intronic |

| | | | | | | | | | | | | | | | | | | | |
|---|---|---|---|---|---|---|---|---|---|---|---|---|---|---|---|---|---|---|---|
| rs9466206 | 6 | 21901658 | 0.96 | 1 | A | C | 0.68 | 0.38 | 0.1 | 0.52 | 0 | 0 | . | . | . | H1.BMP4DM,14_Enh;CHON.BMMSC,11_EnhWk1 | . | *FLJ22536* | Intronic |
| rs9460684 | 6 | 21902053 | 0.94 | 1 | C | G | 0.68 | 0.4 | 0.09 | 0.54 | 0 | 0 | . | . | . | H1.BMP4DM,14_Enh | . | *FLJ22536* | Intronic |
| rs7760611 | 6 | 21903533 | 1 | 1 | T | C | 0.68 | 0.38 | 0.1 | 0.51 | 0 | 0 | . | . | . | HUES64,11 EnhWk1;IPS.20,11 EnhWk1;HD.CD184EC,13 I | . | *FLJ22536* | Intronic |
| rs9460685 | 6 | 21904671 | 1 | 1 | A | G | 0.48 | 0.35 | 0.1 | 0.51 | 0 | 0 | . | . | . | HD.CD184EC,11_EnhWk1 | . | *FLJ22536* | Intronic |
| rs9460686 | 6 | 21904934 | 1 | 1 | A | C | 0.48 | 0.35 | 0.1 | 0.51 | 0 | 0 | . | . | . | HD.CD184EC,11_EnhWk1 | . | *FLJ22536* | Intronic |
| rs9368364 | 6 | 21905643 | 1 | 1 | T | A | 0.39 | 0.33 | 0.1 | 0.51 | 0 | 0 | . | . | . | HD.CD184EC,9_TxEnhG1 | . | *FLJ22536* | Intronic |
| **rs7768862** | **6** | **85088846** | - | - | T | A | 0.34 | 0.36 | 0.26 | 0.51 | 0 | 0 | . | GM12878,6_Weak_Enhancer | . | . | . | *KIAA1009* | . |
| rs7775532 | 6 | 85059005 | 0.88 | -0.98 | A | G | 0.63 | 0.67 | 0.76 | 0.51 | 0 | 0 | . | . | . | . | . | *KIAA1009* | . |
| **rs6940159** | **6** | **170332621** | - | - | T | C | 0.59 | 0.44 | 0.18 | 0.6 | 0 | 0 | . | . | . | CD15.P,12_EnhWk2;SPL,14_Enh | . | *C6orf208* | . |
| **rs144145984** | **8** | **23644003** | - | - | CT | C | 0.53 | 0.46 | 0.53 | 0.42 | 0 | 0 | . | H1,5_Strong_Enhancer | IPS.20,4_TssWk | IPS.DF19,14 Enh;IPS.DF6,14 Enh;HUES64,13 EnhA;HUES | Duke,H9ES,None;UW,H7-hESC,None | *STC1* | . |
| rs453974 | 8 | 23629944 | 0.81 | 0.98 | C | T | 0.57 | 0.72 | 0.58 | 0.82 | 0 | 0 | . | . | . | . | . | *NKX2-6* | . |
| rs218869 | 8 | 23636281 | 0.82 | 0.98 | T | A | 0.59 | 0.72 | 0.58 | 0.82 | 0 | 0 | . | . | . | . | . | *STC1* | . |
| **rs2849506** | **8** | **101329134** | - | - | G | C | 0.61 | 0.34 | 0.46 | 0.36 | 0 | 0 | . | . | IPS.15,1_TssP | IPS.DF19,11_EnhWk1 | . | *RNF19A* | . |
| rs1660338 | 8 | 101242757 | 0.81 | -0.95 | C | G | 0.47 | 0.67 | 0.51 | 0.63 | 0 | 0 | . | . | . | R.MUC29,11_EnhWk1;R.MUC31,13_EnhA | . | *SPAG1* | Intronic |
| rs1788189 | 8 | 101249762 | 0.82 | -0.95 | G | T | 0.72 | 0.7 | 0.51 | 0.63 | 0 | 0 | . | . | . | ST.MUC,11 EnhWk1;R.MUC29,11 EnhWk1;R.MUC31,13 I | . | *SPAG1* | Intronic |
| rs1660353 | 8 | 101250907 | 0.82 | -0.95 | C | T | 0.38 | 0.65 | 0.51 | 0.63 | 0 | 0 | . | NHLF,6_Weak_Enhancer;K562,5_Strong_Enhancer;HSMM,5_Strong_Enhancer | . | ST.MUC,11 EnhWk1;R.MUC31,13 EnhA;HRT.FE,13 EnhA | AWG,A549,None;AWG,HUVEC,None;AWG,LNCaP,None;AWG,MCF-7,None;Duke,LNCaP,androgen;Duke,MCF-7,Hypoxia LacAci | *SPAG1* | Intronic |
| rs34826603 | 8 | 101250993 | 0.82 | -0.95 | CTTA | C | 0.39 | 0.65 | 0.51 | 0.63 | 0 | 0 | . | NHLF,6_Weak_Enhancer;K562,5_Strong_Enhancer;HSMM,5_Strong_Enhancer | . | ST.MUC,11 EnhWk1;R.MUC31,13 EnhA;HRT.FE,13 EnhA | . | *SPAG1* | Intronic |
| rs1692006 | 8 | 101252462 | 0.82 | -0.95 | C | T | 0.74 | 0.7 | 0.51 | 0.63 | 0 | 0 | . | . | . | H1.DMSC,14_Enh;IPS.18,11_EnhWk1;HRT.FE,11_EnhWk1 | Duke,FibroP,None;Duke,Melano,None | *SPAG1* | Intronic |
| rs6511 | 8 | 101252680 | 0.82 | -0.95 | T | C | 0.72 | 0.72 | 0.51 | 0.63 | 0 | 0 | . | . | . | IPS.18,11 EnhWk1;HRT.FE,11 EnhWk1;H1.DMSC,9 TxEnh | . | *SPAG1* | NSM |
| rs924057 | 8 | 101266157 | 0.82 | -0.95 | A | G | 0.38 | 0.66 | 0.51 | 0.63 | 0 | 0 | . | . | . | . | . | *RNF19A* | . |
| rs1692007 | 8 | 101268343 | 0.82 | -0.95 | T | C | 0.38 | 0.66 | 0.51 | 0.63 | 0 | 0 | . | . | . | . | . | *RNF19A* | . |
| rs1616833 | 8 | 101271821 | 0.82 | -0.95 | C | T | 0.38 | 0.66 | 0.51 | 0.63 | 0 | 0 | . | Huvec,4 Strong Enhancer;HSMM,4 Strong Enhancer;GM12878,4 Strong Enhancer;NHLF,5 Strong Enhancer;HMEC,5 S | . | CD34.C,13 EnhA;CD34.MBP1536,13 EnhA;CCC.TREGP,9 | UW,HA-h,None;UW,HRGEC,None;UW,HVMF,None;UW,NHLF,None | *RNF19A* | Intronic |
| rs2861348 | 8 | 101285074 | 0.83 | 0.95 | A | G | 0.62 | 0.34 | 0.48 | 0.37 | 0 | 0 | . | . | . | CD4.MP,11 EnhWk1;H1.DMSC,9 TxEnhG1;LV,9 TxEnhG1 | . | *RNF19A* | Intronic |
| rs35751827 | 8 | 101289836 | 0.83 | 0.95 | A | AT | 0.62 | 0.34 | 0.48 | 0.37 | 0 | 0 | . | . | . | ADI.NUC,14 Enh;HRT.FE,11 EnhWk1;CCIP.LSMPTP,11 E | . | *RNF19A* | Intronic |
| rs1788196 | 8 | 101290088 | 0.81 | 0.93 | C | T | 0.62 | 0.34 | 0.48 | 0.36 | 0 | 0 | . | Huvec,6_Weak_Enhancer | . | ADI.NUC,14 Enh;CCIP.LSMPTP,11 EnhWk1;PFK.3,14 Enh | . | *RNF19A* | Intronic |
| rs1788197 | 8 | 101290928 | 0.83 | 0.95 | T | C | 0.62 | 0.34 | 0.48 | 0.37 | 0 | 0 | . | . | . | CHON.BMMSC,11 EnhWk1;MUS.SC,11 EnhWk1;LIV.A,11 | . | *RNF19A* | Intronic |
| rs2439458 | 8 | 101297984 | 0.83 | 0.95 | T | C | 0.62 | 0.34 | 0.48 | 0.37 | 0 | 0 | . | . | . | CCCRO.MP,11 EnhWk1;CCIP.LSMPTP,9 TxEnhG1;CCC.T | AWG,HeLa-S3,None | *RNF19A* | Intronic |
| rs1660328 | 8 | 101303450 | 0.83 | 0.95 | A | G | 0.62 | 0.34 | 0.48 | 0.36 | 0 | 0 | . | . | . | CD4.MP,11 EnhWk1;PFM.2,11 EnhWk1;PFM.3,11 EnhWk1 | . | *RNF19A* | Intronic |
| rs1660332 | 8 | 101309490 | 0.83 | 0.95 | C | T | 0.62 | 0.34 | 0.48 | 0.36 | 0 | 0 | GM12878,2_Weak_Promoter | . | . | CCIP.LSMPTP,9 TxEnhG1;HRT.FE,11 EnhWk1;LIV.A,14 E | . | *RNF19A* | Intronic |
| rs2439460 | 8 | 101312665 | 0.84 | 0.96 | C | T | 0.62 | 0.34 | 0.48 | 0.36 | 0 | 0 | . | GM12878,4_Strong_Enhancer | . | CCIP.LSMPTP,9 TxEnhG1;CD4.MP,11 EnhWk1;CCCRO.M | . | *RNF19A* | Intronic |
| rs1660348 | 8 | 101315248 | 0.85 | 0.96 | A | G | 0.62 | 0.34 | 0.48 | 0.36 | 1 | 0 | GM12878,2_Weak_Promoter | HepG2,4_Strong_Enhancer;H1,7_Weak_Enhancer | CCC.TMP,4_TssWk | CCIP.LSMPTP,10 TxEnhG2;SK.MUS,9 TxEnhG1;BN.AC,9 | . | *RNF19A* | Intronic |
| rs1788167 | 8 | 101319996 | 0.84 | 0.96 | C | T | 0.62 | 0.34 | 0.49 | 0.36 | 0 | 0 | GM12878,1 Active Promoter;Hep | HSMM,6_Weak_Enhancer;H1,7_Weak_Enhancer | CCIP.LSTP,3 TssA;BN.AG,4 TssWk;BN.CC,4 TssWk;HUES6,4 TssWk;BR.MYO,6 | SK.MUS63,14 Enh;HRT.FE,13 EnhA;CD34.MBP1480,11 E | AWG,GM12878,None;UW,CD20+,None;UW,GM06990,None;UW,GM12864,None;UW,GM12865,None;UW,HL-60,None;UW,RPTEC | *RNF19A* | Intronic |
| rs1788168 | 8 | 101324960 | 0.85 | 0.96 | G | T | 0.62 | 0.34 | 0.48 | 0.37 | 0 | 0 | . | . | CCIP.LSTP,3_TssA;CCIP.LSMPTP,3_TssA | CCC.TMP,14 Enh;ST.SMUS28,14 Enh;CCCRO.MP,14 Enh | . | *RNF19A* | . |
| rs1962901 | 8 | 101326428 | 0.87 | 0.99 | A | G | 0.62 | 0.34 | 0.49 | 0.37 | 0 | 0 | . | GM12878,5_Strong_Enhancer;HepG2,7_Weak_Enhancer;K562,7_Weak_Enhancer | . | HRT.FE,14 Enh;R.MUC31,11 EnhWk1;CCCRA.NP,14 Enh | AWG,HSMM,None;Duke,Hepatocytes,None | *RNF19A* | . |
| rs7846076 | 8 | 101327629 | 0.87 | 0.99 | A | G | 0.41 | 0.31 | 0.49 | 0.36 | 0 | 0 | . | GM12878,5_Strong_Enhancer;HepG2,7_Weak_Enhancer | . | CD34.MBP1562,13 EnhA;CD34.P,11 EnhWk1;CD34.C,11 | Duke,8988T,None;Duke,GM12892,None;Duke,GM19240,None;Duke,Hepatocytes,None | *RNF19A* | . |
| rs7830342 | 8 | 101327738 | 0.87 | 0.99 | T | A | 0.62 | 0.34 | 0.49 | 0.37 | 0 | 0 | . | GM12878,5_Strong_Enhancer;HepG2,7_Weak_Enhancer | . | CD34.MBP1562,13 EnhA;CD34.P,11 EnhWk1;CD34.C,11 | Duke,GM19240,None;Duke,Hepatocytes,None;Duke,MCF-7,Hypoxia LacAcid;Duke,Medullo,None;Duke,Myometr,None;Duke,PanIsl | *RNF19A* | . |
| rs1371867 | 8 | 101330209 | 0.86 | 0.99 | A | C | 0.76 | 0.4 | 0.49 | 0.41 | 0 | 0 | . | . | . | LNG.FE,13_EnhA | AWG,LNCaP,None;Duke,Fibrobl,None | *RNF19A* | . |
| rs1788173 | 8 | 101330939 | 0.87 | 0.99 | C | T | 0.62 | 0.34 | 0.49 | 0.37 | 1 | 0 | . | . | . | LNG.FE,13 EnhA;HD.CD184EC,11 EnhWk1;ST.MUC,11 E | . | *RNF19A* | . |
| rs962452 | 8 | 101331369 | 0.87 | 0.99 | G | A | 0.62 | 0.34 | 0.49 | 0.36 | 0 | 0 | . | . | . | LNG.FE,11_EnhWk1;NCC.GED2,12_EnhWk2 | Duke,Fibrobl,None;Duke,pHTE,None | *RNF19A* | . |
| rs2033921 | 8 | 101331814 | 0.83 | 0.94 | A | G | 0.62 | 0.33 | 0.48 | 0.36 | 0 | 0 | . | . | . | . | . | *RNF19A* | . |
| rs1788175 | 8 | 101332089 | 0.85 | 0.96 | A | G | 0.62 | 0.33 | 0.48 | 0.36 | 0 | 0 | . | . | . | . | . | *RNF19A* | . |
| rs1788180 | 8 | 101333368 | 0.87 | 0.99 | A | G | 0.62 | 0.34 | 0.49 | 0.37 | 0 | 0 | . | . | . | CD3.P,14_Enh | UW,Jurkat,None | *RNF19A* | . |
| rs1788181 | 8 | 101334068 | 0.87 | 0.99 | C | T | 0.62 | 0.34 | 0.49 | 0.37 | 0 | 0 | . | . | . | CCCRA.NP,12_EnhWk2;CD3.P,13_EnhA | . | *RNF19A* | . |
| rs1788182 | 8 | 101334620 | 0.87 | 0.99 | C | T | 0.62 | 0.34 | 0.49 | 0.37 | 0 | 0 | . | GM12878,7_Weak_Enhancer | . | CCIP.LSMPTP,12_EnhWk2;CD3.P,14_Enh | . | *RNF19A* | . |
| rs76164815 | 8 | 101334918 | 0.87 | 0.99 | C | A | 0.62 | 0.34 | 0.49 | 0.37 | 0 | 0 | . | GM12878,7_Weak_Enhancer | . | CCIP.LSMPTP,12_EnhWk2 | AWG,GM12878,None | *RNF19A* | . |
| rs1788184 | 8 | 101335106 | 0.87 | 0.99 | A | G | 0.62 | 0.34 | 0.49 | 0.37 | 0 | 0 | . | GM12878,7_Weak_Enhancer | . | CCIP.LSMPTP,12_EnhWk2;KID.FE,14_Enh | . | *RNF19A* | . |
| rs1788186 | 8 | 101335686 | 0.87 | 0.99 | T | C | 0.62 | 0.34 | 0.49 | 0.37 | 0 | 0 | . | GM12878,5_Strong_Enhancer;Huvec,5_Strong_Enhancer | . | KID.FE,14 Enh;ADI.NUC,11 EnhWk1;LNG.FE,11 EnhWk1 | AWG,GM12878,None;Duke,Melano,None | *RNF19A* | . |
| rs1788187 | 8 | 101336099 | 0.87 | 0.99 | A | G | 0.62 | 0.34 | 0.49 | 0.37 | 1 | 1 | . | GM12878,5_Strong_Enhancer;Huvec,5_Strong_Enhancer | . | KID.FE,14 Enh;LNG.FE,11 EnhWk1;MSC.ADIPC,13 EnhA | Duke,Urothelia,UT189 | *RNF19A* | . |
| **rs142360995** | **8** | **118205719** | - | - | G | A | 0.36 | 0.19 | 0.04 | 0.2 | 0 | 0 | . | . | . | . | . | *SLC30A8* | . |
| **rs10820600** | **9** | **106856692** | - | - | T | C | 0.66 | 0.39 | 0.18 | 0.54 | 0 | 0 | GM12878,1 Active Promoter;K56 | . | BN.FE0,5 TssD1;HD.CD56MESC,3 TssA;HUES6,3 TssA;H9,3 TssA;BN.FE1,3 Tss | SK.MUS62,14_Enh | AWG,A549,None;AWG,Th1,None;Duke,Urothelia,UT189;UW,AG04449,None;UW,AG10803,None;UW,H7-hESC,None;UW,HConF,N | *SMC2* | 5'-UTR |
| rs149606008 | 9 | 106853118 | 0.8 | 0.94 | GAACT | G | 0.54 | 0.41 | 0.17 | 0.57 | 0 | 0 | . | NHLF,6_Weak_Enhancer;GM12878,7_Weak_Enhancer | CHON.BMMSC,2 TssF;PANC.I,6 TssD2;ST.SMUS28,2 TssF;COL.SMUS,6 TssD2;I | KID.FE,12 EnhWk2;ESO,14 Enh;R.MUC31,12 EnhWk2;BM | . | *SMC2* | . |
| rs3818625 | 9 | 106856452 | 0.92 | 0.98 | G | A | 0.71 | 0.43 | 0.19 | 0.58 | 0 | 0 | GM12878,1 Active Promoter;K56 | . | BN.FE0,5 TssD1;HD.CD56MESC,3 TssA;HUES6,3 TssA;H9,3 TssA;BN.FE1,3 Tss | SK.MUS62,14_Enh | AWG,A549,None;AWG,HMEC,None;AWG,HeLa-S3,None;Duke,Hepatocytes,None;UW,AG09309,None;UW,BJ,None;UW,CD20+,Nc | *SMC2* | . |
| rs10820599 | 9 | 106856691 | 0.98 | 1 | T | G | 0.68 | 0.4 | 0.18 | 0.55 | 0 | 0 | GM12878,1 Active Promoter;K56 | . | BN.FE0,5 TssD1;HD.CD56MESC,3 TssA;HUES6,3 TssA;H9,3 TssA;BN.FE1,3 Tss | SK.MUS62,14_Enh | AWG,A549,None;AWG,Th1,None;Duke,Urothelia,UT189;UW,AG04449,None;UW,AG10803,None;UW,H7-hESC,None;UW,HConF,N | *SMC2* | 5'-UTR |
| rs4742903 | 9 | 106856793 | 0.9 | 0.95 | G | C | 0.78 | 0.43 | 0.18 | 0.58 | 0 | 1 | GM12878,1 Active Promoter;K56 | . | BN.FE0,5 TssD1;HD.CD56MESC,3 TssA;HUES6,3 TssA;H9,3 TssA;BN.FE1,3 Tss | SK.MUS62,14_Enh | AWG,GM12878,None;AWG,H1-hESC,None;AWG,HeLa-S3,None;AWG,HepG2,None;AWG,K562,None;AWG,NHEK,None;AWG,Th | *SMC2* | Intronic |
| rs4743687 | 9 | 106856910 | 0.92 | 0.98 | C | T | 0.75 | 0.43 | 0.19 | 0.58 | 0 | 0 | GM12878,1 Active Promoter;K56 | . | HD.CD56MESC,3 TssA;HUES6,3 TssA;BN.FE1,3 TssA;ST.MUC,3 TssA;ADI.MSC | SK.MUS62,14_Enh | NA,HTR8svn,None;UW,Adult_CD4_Th0,None;UW,NT2-D1,None | *SMC2* | 5'-UTR; intronic |
| rs4742905 | 9 | 106856972 | 0.92 | 0.98 | G | C | 0.71 | 0.43 | 0.19 | 0.58 | 0 | 0 | GM12878,1 Active Promoter;K56 | . | HD.CD56MESC,3 TssA;HUES6,3 TssA;BN.FE1,3 TssA;ST.MUC,3 TssA;ADI.MSC | SK.MUS62,14_Enh | Duke,H9ES,None;Duke,Melano,None;Duke,T-47D,None;UW,Jurkat,None | *SMC2* | 5'-UTR; intronic |
| rs4742906 | 9 | 106857078 | 0.9 | 0.98 | A | G | 0.71 | 0.43 | 0.19 | 0.58 | 0 | 0 | GM12878,1 Active Promoter;K56 | . | HD.CD56MESC,3 TssA;HUES6,3 TssA;ADI.MSC,3 TssA;LV,3 TssA;BR.H35,3 Ts | SK.MUS62,14_Enh;SPL,14_Enh | Duke,FibroP,None;Duke,Fibrobl,None;Duke,Ishikawa,4OHTAM 20nM 72hr;Duke,Ishikawa,Estradiol 100nM 1hr;Duke,Melano,None | *SMC2* | 5'-UTR; intronic |
| rs3739736 | 9 | 106857908 | 0.92 | 0.98 | G | A | 0.71 | 0.42 | 0.19 | 0.58 | 0 | 0 | GM12878,1 Active Promoter;K56 | H1,6_Weak_Enhancer;HSMM,6_Weak_Enhancer | CD34.MBP1562,2 TssF;LNG.FE,2 TssF;CD34.MBP1549,4 TssWk;PFK.2,2 TssF;R.I | CD4.MP,13 EnhA;CD3.P,13 EnhA;CD34.MBP1536,14 Enh | Duke,T-47D,None | *SMC2* | Intronic |
| rs3739737 | 9 | 106858192 | 0.92 | 0.98 | A | G | 0.71 | 0.43 | 0.19 | 0.58 | 0 | 0 | GM12878,2 Weak Promoter;K562 | HSMM,6 Weak Enhancer;H1,7 Weak Enhancer;HepG2,6 Weak Enhancer;NHEK,7 Weak Enhancer;Huvec,6 Weak En | CHON.BMMSC,6 TssD2;HUES6,4 TssWk;HUES64,4 TssWk;BN.GM2,4 TssWk;DU | BR.MYO,14 Enh;ES.WA7,14 Enh;SK.MUS63,11 EnhWk1;C | . | *SMC2* | Intronic |
| rs10761010 | 9 | 106858788 | 0.92 | 0.98 | G | A | 0.71 | 0.43 | 0.19 | 0.58 | 0 | 0 | GM12878,2_Weak_Promoter | HepG2,6_Weak_Enhancer;HMEC,6_Weak_Enhancer;NHLF,6_Weak_Enhancer;K562,6_Weak_Enhancer | CHON.BMMSC,6_TssD2;BN.GM2,4_TssWk;KID.FE,4_TssWk;IPS.18,4_TssWk | HRT.FE,11 EnhWk1;LNG.FE,14 Enh;CD34.C,11 EnhWk1;C | AWG,HMEC,None | *SMC2* | Intronic |
| rs7048372 | 9 | 106859738 | 0.8 | 0.94 | T | C | 0.58 | 0.42 | 0.17 | 0.57 | 0 | 0 | . | GM12878,6_Weak_Enhancer | . | . | . | *SMC2* | Intronic |
| rs2274724 | 9 | 106860568 | 0.9 | 0.96 | C | T | 0.79 | 0.43 | 0.19 | 0.58 | 0 | 0 | . | GM12878,6_Weak_Enhancer | . | . | . | *SMC2* | Intronic |
| rs10820601 | 9 | 106861281 | 0.87 | 0.96 | G | A | 0.76 | 0.4 | 0.17 | 0.56 | 0 | 0 | . | . | . | . | . | *SMC2* | Intronic |
| rs7875007 | 9 | 106865691 | 0.87 | 0.97 | A | T | 0.74 | 0.4 | 0.17 | 0.53 | 0 | 0 | . | . | . | . | . | *SMC2* | Intronic |
| rs7859034 | 9 | 106865692 | 0.87 | 0.97 | G | T | 0.74 | 0.4 | 0.17 | 0.53 | 0 | 0 | . | . | . | . | . | *SMC2* | Intronic |
| rs2122577 | 9 | 106866703 | 0.9 | 0.96 | C | T | 0.79 | 0.43 | 0.19 | 0.58 | 0 | 0 | . | . | . | . | . | *SMC2* | Intronic |
| rs139217198 | 9 | 106872864 | 0.86 | 0.96 | A | ATGT | 0.71 | 0.44 | 0.19 | 0.58 | 0 | 0 | . | . | . | . | . | *SMC2* | Intronic |
| rs7854630 | 9 | 106875277 | 0.9 | 0.96 | G | T | 0.71 | 0.43 | 0.19 | 0.58 | 0 | 0 | . | . | . | . | . | *SMC2* | Intronic |
| rs10820603 | 9 | 106877939 | 0.9 | 0.96 | G | A | 0.79 | 0.43 | 0.19 | 0.58 | 0 | 0 | . | . | . | . | . | *SMC2* | Intronic |
| rs35687655 | 9 | 106878839 | 0.89 | 0.96 | TATCA | T | 0.79 | 0.44 | 0.19 | 0.57 | 0 | 0 | . | . | . | . | . | *SMC2* | Intronic |
| rs7350148 | 9 | 106883085 | 0.9 | 0.96 | T | G | 0.7 | 0.43 | 0.19 | 0.58 | 0 | 0 | . | . | . | . | . | *SMC2* | Intronic |
| rs2417487 | 9 | 106887581 | 0.9 | 0.96 | A | G | 0.73 | 0.43 | 0.19 | 0.57 | 0 | 0 | . | . | . | . | . | *SMC2* | Intronic |
| rs1026181 | 9 | 106893334 | 0.9 | 0.96 | A | G | 0.72 | 0.44 | 0.19 | 0.58 | 0 | 0 | . | . | . | . | . | *SMC2* | Intronic |
| rs7872034 | 9 | 106896809 | 0.9 | 0.96 | G | A | 0.79 | 0.44 | 0.19 | 0.58 | 1 | 1 | . | . | . | . | . | *SMC2* | SYN |
| rs10122512 | 9 | 106898410 | 0.9 | 0.96 | C | T | 0.79 | 0.43 | 0.19 | 0.58 | 0 | 0 | . | . | . | . | . | *SMC2* | Intronic |
| rs34651335 | 9 | 106899951 | 0.89 | 0.96 | TA | T | 0.79 | 0.43 | 0.19 | 0.58 | 0 | 0 | . | . | . | . | . | *SMC2* | Intronic |
| rs7023085 | 9 | 106904265 | 0.9 | 0.96 | C | T | 0.79 | 0.43 | 0.19 | 0.58 | 0 | 0 | . | . | . | . | . | *SMC2* | . |
| rs10991119 | 9 | 106905277 | 0.89 | 0.96 | T | C | 0.79 | 0.43 | 0.19 | 0.57 | 0 | 0 | . | . | H1.BMP4DT,6_TssD2 | . | . | *SMC2* | . |
| rs200238725 | 9 | 106906953 | 0.83 | 0.93 | T | TTG | 0.67 | 0.43 | 0.18 | 0.56 | 0 | 0 | . | . | . | . | . | *SMC2* | . |
| rs111648194 | 9 | 106906954 | 0.82 | 0.91 | T | TG | 0.68 | 0.41 | 0.18 | 0.55 | 0 | 0 | . | . | . | . | . | *SMC2* | . |
| rs200052337 | 9 | 106906962 | 0.83 | 0.93 | TG | T | 0.69 | 0.42 | 0.18 | 0.57 | 0 | 0 | . | . | . | . | . | *SMC2* | . |
| rs10820609 | 9 | 106907332 | 0.9 | 0.96 | G | A | 0.79 | 0.43 | 0.19 | 0.58 | 0 | 0 | . | . | . | . | . | *SMC2* | . |
| rs10991120 | 9 | 106909051 | 0.88 | 0.95 | C | T | 0.79 | 0.43 | 0.19 | 0.58 | 0 | 0 | . | . | . | . | . | *SMC2* | . |
| rs4743689 | 9 | 106909300 | 0.88 | 0.95 | G | A | 0.72 | 0.43 | 0.19 | 0.58 | 0 | 0 | . | . | . | . | . | *SMC2* | . |
| rs4743690 | 9 | 106909577 | 0.88 | 0.95 | T | C | 0.71 | 0.43 | 0.19 | 0.58 | 0 | 0 | . | . | . | KID.FE,12_EnhWk2 | . | *SMC2* | . |
| rs10217250 | 9 | 106911633 | 0.84 | 0.94 | A | T | 0.7 | 0.41 | 0.17 | 0.52 | 0 | 0 | . | GM12878,6_Weak_Enhancer;NHEK,7_Weak_Enhancer;NHLF,6_Weak_Enhancer | . | . | . | *SMC2* | . |
| rs10217420 | 9 | 106911946 | 0.83 | 0.92 | G | A | 0.51 | 0.4 | 0.18 | 0.51 | 0 | 0 | . | . | . | . | . | *SMC2* | . |
| rs10739885 | 9 | 106912892 | 0.88 | 0.95 | G | A | 0.79 | 0.43 | 0.19 | 0.58 | 0 | 0 | . | . | . | . | . | *SMC2* | . |
| rs9942894 | 9 | 106914905 | 0.88 | 0.95 | A | G | 0.8 | 0.43 | 0.19 | 0.58 | 0 | 0 | . | . | . | . | . | *SMC2* | . |
| **rs2901157** | **10** | **119262365** | - | - | A | G | 0.02 | 0.07 | 0.26 | 0.13 | 0 | 0 | . | . | . | H1.DNP,11 EnhWk1;BN.CC,12 EnhWk2;BN.FE2,12 EnhW | . | *EMX2OS* | Intronic |
| **rs10838267** | **11** | **44368892** | - | - | G | A | 0.11 | 0.35 | 0.32 | 0.51 | 0 | 0 | . | K562,6_Weak_Enhancer | . | BN.FE2,14 Enh;IPS.18,12 EnhWk2;H1.DNP,11 EnhWk1;BN | . | *ALX4* | . |
| rs7125755 | 11 | 44367897 | 0.99 | 1 | C | G | 0.29 | 0.36 | 0.32 | 0.53 | 0 | 0 | . | . | . | BN.FE2,14_Enh;IPS.DF19,11_EnhWk1;BR.MYO,14_Enh | AWG,K562,None | *ALX4* | . |
| rs4755816 | 11 | 44368416 | 0.98 | 1 | G | T | 0.31 | 0.36 | 0.33 | 0.53 | 0 | 0 | . | K562,7_Weak_Enhancer | . | BN.FE2,14 Enh;ES.WA7,15 EnhP;IPS.18,12 EnhWk2;H1.DI | . | *ALX4* | . |
| rs3853291 | 11 | 44369477 | 0.99 | 1 | G | A | 0.29 | 0.36 | 0.32 | 0.51 | 0 | 0 | . | . | . | BN.FE2,14_Enh;H1.DNP,11_EnhWk1;NCC.COR2,15_EnhP | . | *ALX4* | . |
| rs4755817 | 11 | 44372916 | 0.88 | 0.95 | G | A | 0.1 | 0.35 | 0.31 | 0.51 | 0 | 0 | . | GM12878,4_Strong_Enhancer | CD19.P,6_TssD2 | ESO,12_EnhWk2 | Duke,GM19240,None | *ALX4* | . |
| **rs78588049** | **12** | **69180907** | - | - | ATTTT | A | 0.05 | 0.17 | 0.14 | 0.16 | 0 | 1 | . | HepG2,4 Strong Enhancer;Huvec,5 Strong Enhancer;HMEC,5 Strong Enhancer;GM12878,7 Weak Enhancer;NHEK,5 S | . | CD15.P,13 EnhA;PFK.3,11 EnhWk1;PFF.1,11 EnhWk1;ADI | AWG,HepG2,None;AWG,K562,None;AWG,LNCaP,None;AWG,MCF-7,None;Duke,8988T,None;Duke,AoSMC,None;Duke,HeLa-S3,I | *SLC35E3* | . |
| **rs855596** | **12** | **103045519** | - | - | T | C | 1 | 0.99 | 0.93 | 0.95 | 0 | 0 | . | . | . | . | . | *LOC283432* | . |
| rs703564 | 12 | 103020623 | 0.84 | -0.94 | A | C | 0 | 0.01 | 0.06 | 0.04 | 0 | 0 | . | . | . | . | . | *IGF1* | . |
| rs703567 | 12 | 103021142 | 0.81 | -0.92 | A | G | 0 | 0.01 | 0.07 | 0.04 | 0 | 0 | . | . | . | . | . | *IGF1* | . |
| rs697195 | 12 | 103023753 | 0.81 | -0.92 | A | G | 0 | 0.01 | 0.07 | 0.04 | 0 | 0 | . | K562,7_Weak_Enhancer;HSMM,5_Strong_Enhancer;NHLF,6_Weak_Enhancer | . | MSC.ADIPC,13 EnhA;ADI.MSC,13 EnhA;BM.MSC,13 Enh | . | *IGF1* | . |
| rs703576 | 12 | 103025828 | 0.81 | -0.92 | C | G | 0 | 0.01 | 0.07 | 0.04 | 0 | 0 | . | HSMM,5_Strong_Enhancer | . | ADI.MSC,13_EnhA;BM.MSC,13_EnhA | . | *IGF1* | . |
| rs703579 | 12 | 103030938 | 0.81 | -0.92 | A | C | 0 | 0.01 | 0.07 | 0.04 | 0 | 0 | . | . | . | . | . | *IGF1* | . |
| rs703581 | 12 | 103032097 | 0.87 | -0.94 | T | C | 0 | 0.01 | 0.07 | 0.04 | 0 | 0 | . | . | . | . | . | *IGF1* | . |
| rs1070209 | 12 | 103033217 | 0.87 | -0.94 | A | G | 0.02 | 0.02 | 0.07 | 0.04 | 0 | 0 | . | . | . | . | . | *IGF1* | . |
| rs703584 | 12 | 103034820 | 0.87 | -0.94 | C | A | 0 | 0.01 | 0.07 | 0.04 | 0 | 0 | . | . | . | . | . | *IGF1* | . |
| rs703585 | 12 | 103035129 | 0.87 | -0.94 | C | T | 0 | 0.01 | 0.07 | 0.04 | 0 | 0 | . | . | . | . | . | *IGF1* | . |
| rs703587 | 12 | 103036245 | 0.87 | -0.94 | A | T | 0 | 0.01 | 0.07 | 0.04 | 0 | 0 | . | . | . | ST.MUC,11_EnhWk1;LIV.A,11_EnhWk1 | AWG,HSMM,None;UW,AG09309,None;UW,HAEpiC,None;UW,HFF-Myc,None;UW,HIPEpiC,None;UW,HNPCEpiC,None;UW,HPdL | *IGF1* | . |
| rs703589 | 12 | 103038955 | 0.97 | -1 | T | C | 0 | 0.01 | 0.07 | 0.04 | 0 | 0 | . | . | . | ST.MUC,11_EnhWk1 | . | *LOC283432* | . |
| rs2643673 | 12 | 103042298 | 0.9 | 1 | T | C | 0.99 | 0.99 | 0.92 | 0.96 | 0 | 0 | . | . | . | . | . | *LOC283432* | . |
| rs833732 | 12 | 103043313 | 0.97 | 1 | A | G | 1 | 0.99 | 0.93 | 0.96 | 0 | 0 | . | . | . | . | . | *LOC283432* | . |
| rs833733 | 12 | 103044411 | 0.97 | 1 | C | T | 1 | 0.99 | 0.93 | 0.96 | 0 | 0 | . | . | . | . | . | *LOC283432* | . |
| rs833734 | 12 | 103044493 | 0.97 | 1 | A | G | 1 | 0.99 | 0.93 | 0.96 | 0 | 0 | . | . | . | . | . | *LOC283432* | . |
| rs1495389 | 12 | 103045157 | 0.97 | 1 | C | T | 1 | 0.99 | 0.93 | 0.96 | 0 | 0 | . | . | . | . | . | *LOC283432* | . |
| rs833736 | 12 | 103046430 | 1 | 1 | C | G | 1 | 0.99 | 0.93 | 0.96 | 0 | 0 | . | . | . | . | . | *LOC283432* | . |
| rs833737 | 12 | 103046442 | 1 | 1 | G | A | 1 | 0.99 | 0.93 | 0.96 | 0 | 0 | . | . | . | . | . | *LOC283432* | . |
| rs833738 | 12 | 103047220 | 1 | 1 | G | A | 1 | 0.99 | 0.93 | 0.96 | 0 | 0 | . | GM12878,7_Weak_Enhancer | . | . | Duke,Fibrobl,None | *LOC283432* | . |
| rs833740 | 12 | 103047582 | 0.95 | 0.97 | T | C | 1 | 0.99 | 0.93 | 0.96 | 0 | 0 | . | . | . | . | . | *LOC283432* | . |
| rs833742 | 12 | 103051885 | 0.89 | 0.94 | A | C | 1 | 0.99 | 0.93 | 0.96 | 0 | 0 | . | . | . | . | . | *LOC283432* | . |
| rs1102946 | 12 | 103053121 | 0.89 | 0.94 | C | T | 1 | 0.99 | 0.93 | 0.96 | 0 | 0 | . | . | . | . | UW,AG10803,None | *LOC283432* | . |
| rs2132249 | 12 | 103056076 | 0.89 | 0.94 | G | A | 0.92 | 0.98 | 0.93 | 0.96 | 0 | 0 | . | . | . | . | . | *LOC283432* | . |
| rs1845680 | 12 | 103057592 | 0.89 | 0.94 | T | A | 1 | 0.99 | 0.93 | 0.96 | 0 | 0 | . | . | . | . | . | *LOC283432* | . |
| rs833707 | 12 | 103062685 | 0.89 | 0.94 | A | T | 1 | 0.99 | 0.93 | 0.96 | 0 | 0 | . | . | . | BM.MSC,12_EnhWk2 | . | *LOC283432* | . |
| rs833708 | 12 | 103064743 | 0.87 | 0.94 | C | T | 1 | 0.99 | 0.93 | 0.96 | 0 | 0 | . | . | . | . | . | *LOC283432* | . |
| rs833710 | 12 | 103065094 | 0.87 | 0.94 | G | A | 1 | 0.99 | 0.93 | 0.96 | 0 | 0 | . | . | . | . | . | *LOC283432* | . |
| rs833711 | 12 | 103065246 | 0.87 | 0.94 | A | G | 1 | 0.99 | 0.93 | 0.96 | 0 | 0 | . | . | . | . | . | *LOC283432* | . |
| rs833712 | 12 | 103066119 | 0.87 | 0.94 | A | G | 1 | 0.99 | 0.93 | 0.96 | 0 | 0 | . | HSMM,7_Weak_Enhancer | . | . | Duke,PanIsletD,None;UW,AG09309,None;UW,HFF-Myc,None;UW,SK-N-MC,None | *LOC283432* | . |
| rs833715 | 12 | 103068400 | 0.8 | 0.94 | A | G | 0.88 | 0.85 | 0.92 | 0.85 | 0 | 0 | . | . | . | . | . | *LOC283432* | . |
| **rs9316500** | **13** | **51094114** | - | - | T | G | 0.27 | 0.27 | 0.66 | 0.29 | 0 | 0 | . | . | . | MSC.ADIPC,11 EnhWk1;PFM.1,11 EnhWk1;PFM.2,14 Enh | AWG,LNCaP,None;UW,HCPEpiC,None;UW,HVMF,None | *DLEU7* | . |
| rs7991128 | 13 | 51067234 | 0.9 | 0.96 | T | G | 0.2 | 0.22 | 0.66 | 0.23 | 1 | 1 | . | . | . | . | . | *DLEU7* | . |
| rs9568434 | 13 | 51081962 | 0.89 | 0.95 | G | A | 0.21 | 0.22 | 0.66 | 0.23 | 0 | 0 | . | . | . | . | . | *DLEU7* | . |
| rs142988505 | 13 | 51082804 | 0.89 | 0.96 | G | A | 0.03 | 0.19 | 0.65 | 0.23 | 0 | 0 | . | . | . | . | UW,Jurkat,None | *DLEU7* | . |
| rs9562984 | 13 | 51082920 | 0.88 | 0.96 | C | T | 0.21 | 0.21 | 0.65 | 0.23 | 0 | 0 | . | . | . | . | UW,Jurkat,None | *DLEU7* | . |
| rs9562985 | 13 | 51085041 | 0.92 | 0.97 | G | A | 0.21 | 0.22 | 0.66 | 0.23 | 0 | 0 | . | HepG2,7_Weak_Enhancer | . | ADI.MSC,12 EnhWk2;PFM.2,12 EnhWk2;CD34.C,13 EnhA | . | *DLEU7* | . |
| rs2286657 | 13 | 51087007 | 0.91 | 0.96 | T | A | 0.21 | 0.22 | 0.66 | 0.23 | 0 | 0 | HepG2,1 Active Promoter;Huvec,: | K562,4_Strong_Enhancer;HMEC,6_Weak_Enhancer;HSMM,4_Strong_Enhancer;NHLF,4_Strong_Enhancer | CD34.MBP1562,2 TssF;DUO.MUC61,6 TssD2;CD15.P,6 TssD2;DUO.SMUS,3 Tss | SPL,14 Enh;CD34.MBP1536,13 EnhA;PFM.3,13 EnhA;GAS | Duke,GM19239,None;UW,HA-sp,None;UW,HRGEC,None;UW,RPTEC,None | *DLEU7* | . |
| rs9568440 | 13 | 51089146 | 0.95 | 0.98 | A | T | 0.21 | 0.22 | 0.66 | 0.23 | 0 | 0 | . | . | . | NCC.COR2,11 EnhWk1;SK.MUS62,11 EnhWk1;CD34.P,14 | . | *DLEU7* | . |
| rs9562986 | 13 | 51091081 | 0.95 | 0.98 | C | T | 0.21 | 0.22 | 0.66 | 0.23 | 0 | 0 | . | . | . | PFM.2,14 Enh;NCC.COR2,11 EnhWk1;BN.FE2,12 EnhWk2 | . | *DLEU7* | . |
| rs9568441 | 13 | 51093778 | 0.98 | 1 | C | A | 0.21 | 0.22 | 0.66 | 0.23 | 0 | 0 | . | . | . | MSC.ADIPC,11 EnhWk1;PFM.1,11 EnhWk1;CD4.MP,11 E | . | *DLEU7* | . |
| rs963740 | 13 | 51096095 | 0.98 | 1 | A | T | 0.27 | 0.27 | 0.66 | 0.29 | 1 | 1 | . | NHLF,6_Weak_Enhancer;HepG2,6_Weak_Enhancer;HSMM,7_Weak_Enhancer | MSC.ADIPC,6_TssD2;SK.MUS,6_TssD2 | COL.SMUS,11 EnhWk1;R.MUC31,11 EnhWk1;KID.FE,11 | AWG,HSMM,None;AWG,HepG2,None;AWG,LNCaP,None;Duke,AoSMC,None;Duke,FibroP,None;Duke,Fibrobl,None;Duke,Hepatoc | *DLEU7* | . |
| rs9568442 | 13 | 51097400 | 0.94 | 1 | G | A | 0.2 | 0.23 | 0.65 | 0.23 | 0 | 0 | . | NHLF,7_Weak_Enhancer;HSMM,7_Weak_Enhancer | MSC.ADIPC,6_TssD2 | COL.SMUS,11 EnhWk1;KID.FE,11 EnhWk1;ADI.NUC,11 | AWG,LNCaP,None;UW,BE2_C,None | *DLEU7* | . |
| rs279075 | 13 | 51100714 | 0.9 | 0.98 | A | C | 0.29 | 0.21 | 0.64 | 0.22 | 0 | 0 | . | NHLF,7_Weak_Enhancer | . | NCC.COR2,11 EnhWk1;HRT.FE,13 EnhA;CD3.P,11 EnhW | . | *DLEU7* | . |
| rs9634835 | 13 | 51101698 | 0.88 | 0.98 | G | A | 0.21 | 0.18 | 0.64 | 0.2 | 0 | 0 | . | NHLF,7_Weak_Enhancer;HMEC,7_Weak_Enhancer;HSMM,6_Weak_Enhancer;NHEK,7_Weak_Enhancer | ST.SMUS28,6_TssD2;SK.MUS,6_TssD2 | HRT.FE,13 EnhA;CD3.P,11 EnhWk1;CD34.MBP1562,13 E | UW,HA-sp,None | *DLEU7* | . |
| rs7994628 | 13 | 51104838 | 0.88 | 0.98 | A | G | 0.21 | 0.18 | 0.64 | 0.2 | 0 | 0 | . | . | . | CD3.P,11 EnhWk1;NCC.COR2,13 EnhA;IMR90,14 Enh;PFI | . | *DLEU7* | . |
| rs6561582 | 13 | 51115607 | 0.84 | 0.98 | G | A | 0.2 | 0.18 | 0.63 | 0.2 | 0 | 0 | . | . | . | . | . | *DLEU7* | . |
| rs4603449 | 13 | 51120008 | 0.83 | 0.98 | T | G | 0.2 | 0.17 | 0.63 | 0.2 | 0 | 0 | . | . | . | . | . | *DLEU7* | . |

| | | | | | | | | | | | | | | | | | | | |
|---|---|---|---|---|---|---|---|---|---|---|---|---|---|---|---|---|---|---|---|
| **rs75004998** | **14** | **77517786** | - | - | G | A | 0.08 | 0.27 | 0.49 | 0.34 | 0 | 0 | HepG2,2_Weak_Promoter | HSMM,5_Strong_Enhancer;NHLF,5_Strong_Enhancer;H1,7_Weak_Enhancer;HMEC,6_Weak_Enhancer | SK.MUS63,6 TssD2;PFF.1,6 TssD2;IPS.DF19,6 TssD2;BR.MYO,6 TssD2;ST.SMUS | PFM.3,11 EnhWk1;HRT.FE,13 EnhA;BN.GM2,11 EnhWk1; | AWG,HMEC,None;AWG,HSMMtube,None;AWG,HepG2,None;Duke,FibroP,None;Duke,Hepatocytes,None;Duke,Ishikawa,4OHTAM | *IRF2BPL* | Intronic |
| rs10873301 | 14 | 77507205 | 0.83 | 0.94 | G | T | 0.07 | 0.26 | 0.48 | 0.3 | 0 | 1 | H1,2 Weak Promoter;K562,1 Acti | HSMM,4 Strong Enhancer;HMEC,4 Strong Enhancer;NHEK,4 Strong Enhancer;Huvec,4 Strong Enhancer;NHLF,4 Stro | CD34.MBP1562,2 TssF;R.SMUS,6 TssD2;BM.MSC,2 TssF;CD15.P,2 TssF;ES.WA7 | CD34.MBP1536,13 EnhA;GAS,14 Enh;SK.MUS62,13 EnhA | AWG,HUVEC,None;AWG,HeLa-S3,None;AWG,MCF-7,None;Duke,Chorion,None;Duke,FibroP,None;Duke,GM19239,None;Duke,HP | *IRF2BPL* | . |
| rs35749957 | 14 | 77508276 | 0.83 | 0.93 | CA | C | 0.31 | 0.28 | 0.48 | 0.31 | 0 | 0 | . | HSMM,4 Strong Enhancer;Huvec,4 Strong Enhancer;NHLF,4 Strong Enhancer;HMEC,5 Strong Enhancer;NHEK,5 Stro | ADI.MSC,2 TssF;CD34.MBP1562,2 TssF;BN.FE2,2 TssF;ST.SMUS28,2 TssF;DUO. | CD34.MBP1536,13 EnhA;SK.MUS62,13 EnhA;BR.H35,13 | UW,Jurkat,None | *IRF2BPL* | Intronic |
| rs11159247 | 14 | 77509872 | 0.87 | 0.96 | G | A | 0.08 | 0.26 | 0.48 | 0.3 | 1 | 1 | . | HSMM,4 Strong Enhancer;NHLF,4 Strong Enhancer;H1,7 Weak Enhancer;NHEK,4 Strong Enhancer;Huvec,5 Strong E | ADI.MSC,2 TssF;SK.MUS,6 TssD2;CD34.MBP1562,2 TssF;PFM.2,2 TssF;BN.AC,2 | CD34.MBP1536,13 EnhA;SK.MUS62,13 EnhA;BN.AG,13 | AWG,A549,None;AWG,HMEC,None;AWG,HSMM,None;AWG,HeLa-S3,None;AWG,K562,None;AWG,NHEK,None;AWG,Th1,None | *IRF2BPL* | Intronic |
| rs55715661 | 14 | 77513173 | 0.97 | 1 | C | T | 0.08 | 0.27 | 0.5 | 0.34 | 0 | 0 | . | Huvec,7 Weak Enhancer;HMEC,5 Strong Enhancer;GM12878,7 Weak Enhancer;K562,7 Weak Enhancer;NHLF,7 Wea | . | LV,13 EnhA;PFK.3,13 EnhA;R.MUC29,13 EnhA;DUO.MU | . | *IRF2BPL* | Intronic |
| rs17105227 | 14 | 77513800 | 0.97 | 1 | G | A | 0.08 | 0.27 | 0.5 | 0.34 | 0 | 0 | . | HepG2,7_Weak_Enhancer | . | CD34.MBP1480,11 EnhWk1;CD34.MBP1562,11 EnhWk1;C | UW,Monocytes-CD14+_RO01746,None | *IRF2BPL* | Intronic |
| rs2363514 | 14 | 77516513 | 0.95 | 1 | C | G | 0.19 | 0.41 | 0.51 | 0.4 | 0 | 0 | . | GM12878,7_Weak_Enhancer | . | HRT.FE,14_Enh;PFM.2,11_EnhWk1 | . | *IRF2BPL* | Intronic |
| rs11622493 | 14 | 77517045 | 0.96 | 1 | A | G | 0.08 | 0.41 | 0.5 | 0.41 | 0 | 1 | . | . | . | LIV.A,11 EnhWk1;NCC.GED2,11 EnhWk1;PFM.2,13 EnhA | Duke,FibroP,None | *IRF2BPL* | Intronic |
| rs11622562 | 14 | 77517148 | 0.97 | 0.99 | C | T | 0.08 | 0.27 | 0.49 | 0.33 | 0 | 0 | . | . | . | LIV.A,11 EnhWk1;NCC.GED2,11 EnhWk1;PFM.2,13 EnhA | . | *IRF2BPL* | Intronic |
| rs205810 | 14 | 77517894 | 0.99 | 1 | G | A | 0.35 | 0.29 | 0.49 | 0.34 | 0 | 0 | . | HepG2,6 Weak Enhancer;K562,7 Weak Enhancer;HSMM,7 Weak Enhancer;HMEC,7 Weak Enhancer;NHLF,7 Weak | PFF.1,6_TssD2;PFF.2,6_TssD2;BN.ITL,6_TssD2;ST.SMUS28,4_TssWk | HRT.FE,13 EnhA;BN.GM2,11 EnhWk1;LNG.FE,13 EnhA;E | . | *IRF2BPL* | Intronic |
| rs4021419 | 14 | 77519693 | 0.93 | -0.99 | T | C | 0.53 | 0.56 | 0.5 | 0.59 | 0 | 0 | . | H1,7_Weak_Enhancer | PFF.2,6_TssD2 | BN.GM2,11 EnhWk1;CD34.C,11 EnhWk1;BR.H35,11 EnhV | AWG,A549,None;AWG,GM12878,None;AWG,H1-hESC,None;AWG,HMEC,None;AWG,HSMM,None;AWG,HSMMtube,None;AWG | *IRF2BPL* | Intronic |
| rs4021409 | 14 | 77519874 | 0.93 | -0.99 | T | G | 0.57 | 0.57 | 0.5 | 0.59 | 0 | 0 | . | H1,6_Weak_Enhancer | PFF.2,4_TssWk;CCCRA.NP,6_TssD2 | BN.GM2,11 EnhWk1;CD34.C,11 EnhWk1;H1.DMSC,11 Er | UW,PANC-1,None | *IRF2BPL* | Intronic |
| rs12431767 | 14 | 77519917 | 0.93 | 0.99 | T | A | 0.08 | 0.41 | 0.5 | 0.4 | 0 | 0 | . | H1,6_Weak_Enhancer | PFF.2,4_TssWk;CCCRA.NP,6_TssD2 | BN.GM2,11 EnhWk1;CD34.C,11 EnhWk1;H1.DMSC,11 Er | . | *IRF2BPL* | Intronic |
| **rs8027365** | **15** | **75808740** | - | - | A | C | 0.61 | 0.28 | 0.38 | 0.27 | 0 | 0 | . | . | . | MSC.ADIPC,11_EnhWk1 | . | *PTPN9* | Intronic |
| rs4886707 | 15 | 75755467 | 0.84 | 0.92 | C | T | 0.55 | 0.28 | 0.38 | 0.27 | 0 | 0 | . | H1,6_Weak_Enhancer | . | H9,14 Enh;IPS.DF19,14 Enh;HUES48,11 EnhWk1;ES.I3,11 | . | *PTPN9* | . |
| rs4545784 | 15 | 75755651 | 0.85 | 0.93 | A | G | 0.56 | 0.28 | 0.38 | 0.27 | 0 | 0 | . | . | . | IPS.DF19,14 Enh;HUES48,11 EnhWk1;HUES64,11 EnhWk1 | . | *PTPN9* | . |
| rs11634863 | 15 | 75766672 | 0.85 | 0.93 | C | A | 0.56 | 0.27 | 0.38 | 0.26 | 0 | 0 | . | . | . | HD.CD184EC,9_TxEnhG1 | . | *PTPN9* | Intronic |
| rs10584118 | 15 | 75784091 | 0.86 | 0.95 | TCAGA | T | 0.53 | 0.27 | 0.37 | 0.26 | 0 | 0 | . | . | . | . | . | *PTPN9* | Intronic |
| rs7495610 | 15 | 75784441 | 0.91 | 0.96 | T | C | 0.6 | 0.28 | 0.38 | 0.26 | 0 | 0 | . | . | . | . | . | *PTPN9* | Intronic |
| rs4299117 | 15 | 75789444 | 0.92 | 0.96 | C | T | 0.59 | 0.27 | 0.38 | 0.26 | 0 | 0 | . | . | . | . | . | *PTPN9* | Intronic |
| rs11637586 | 15 | 75791112 | 0.92 | 0.96 | G | C | 0.59 | 0.27 | 0.38 | 0.26 | 0 | 0 | . | . | . | . | . | *PTPN9* | Intronic |
| rs12916964 | 15 | 75792297 | 0.92 | 0.96 | T | A | 0.53 | 0.27 | 0.38 | 0.26 | 0 | 0 | . | . | . | . | . | *PTPN9* | Intronic |
| rs4381565 | 15 | 75802154 | 0.94 | 0.98 | A | G | 0.59 | 0.26 | 0.38 | 0.26 | 0 | 0 | . | . | . | . | . | *PTPN9* | Intronic |
| rs12708519 | 15 | 75803200 | 0.97 | 0.99 | T | C | 0.61 | 0.26 | 0.38 | 0.26 | 0 | 0 | . | . | . | . | . | *PTPN9* | Intronic |
| rs11639183 | 15 | 75807692 | 0.98 | 0.99 | T | C | 0.61 | 0.26 | 0.38 | 0.26 | 0 | 0 | . | . | . | . | . | *PTPN9* | Intronic |
| rs8027802 | 15 | 75808731 | 0.99 | 1 | G | A | 0.59 | 0.27 | 0.38 | 0.27 | 0 | 0 | . | . | . | MSC.ADIPC,11_EnhWk1 | . | *PTPN9* | Intronic |
| rs8027546 | 15 | 75808791 | 0.97 | 1 | C | T | 0.6 | 0.27 | 0.38 | 0.26 | 0 | 0 | . | Huvec,7_Weak_Enhancer | . | MSC.ADIPC,11_EnhWk1 | . | *PTPN9* | Intronic |
| rs8029112 | 15 | 75808972 | 0.99 | 1 | T | C | 0.61 | 0.26 | 0.38 | 0.26 | 0 | 0 | . | Huvec,7_Weak_Enhancer | . | MSC.ADIPC,11 EnhWk1;PFF.2,11 EnhWk1;IMR90,9 TxEn | AWG,A549,None;AWG,HMEC,None;AWG,HSMM,None;AWG,HSMMtube,None;AWG,HUVEC,None;AWG,HeLa-S3,None;AWG,Ll | *PTPN9* | Intronic |
| rs4322627 | 15 | 75810659 | 0.98 | 0.99 | C | T | 0.61 | 0.26 | 0.38 | 0.26 | 0 | 0 | . | . | . | PFF.1,9 TxEnhG1;PFF.2,9 TxEnhG1;H1.DMSC,9 TxEnhG1 | . | *PTPN9* | Intronic |
| rs199993688 | 15 | 75811158 | 0.95 | 0.99 | A | AG | 0.57 | 0.27 | 0.37 | 0.26 | 0 | 0 | . | . | . | . | . | *PTPN9* | Intronic |
| rs7180574 | 15 | 75811599 | 0.98 | 0.99 | A | G | 0.61 | 0.26 | 0.38 | 0.26 | 0 | 0 | . | . | . | . | . | *PTPN9* | Intronic |
| rs28610581 | 15 | 75812474 | 0.98 | 0.99 | A | G | 0.58 | 0.26 | 0.38 | 0.26 | 0 | 0 | . | . | . | . | . | *PTPN9* | Intronic |
| rs4886712 | 15 | 75812888 | 0.93 | 0.97 | C | T | 0.61 | 0.26 | 0.39 | 0.27 | 0 | 0 | . | . | . | . | . | *PTPN9* | Intronic |
| rs8030802 | 15 | 75813153 | 0.98 | 0.99 | A | G | 0.61 | 0.26 | 0.38 | 0.26 | 0 | 0 | . | . | . | H1.DMSC,9_TxEnhG1 | . | *PTPN9* | Intronic |
| rs6495182 | 15 | 75814388 | 0.98 | 0.99 | C | T | 0.55 | 0.26 | 0.38 | 0.26 | 0 | 0 | . | . | . | H1.DMSC,9_TxEnhG1 | . | *PTPN9* | Intronic |
| rs8023268 | 15 | 75814672 | 0.98 | 0.99 | A | T | 0.61 | 0.26 | 0.38 | 0.26 | 0 | 0 | . | . | . | H1.DMSC,9_TxEnhG1 | . | *PTPN9* | Intronic |
| rs8023815 | 15 | 75814704 | 0.98 | 0.99 | G | A | 0.61 | 0.26 | 0.38 | 0.26 | 0 | 0 | . | . | . | H1.DMSC,9_TxEnhG1 | . | *PTPN9* | Intronic |
| rs11636031 | 15 | 75815758 | 0.98 | 0.99 | T | C | 0.61 | 0.26 | 0.38 | 0.26 | 0 | 0 | . | . | . | H1.DMSC,9_TxEnhG1;CHON.BMMSC,9_TxEnhG1 | . | *PTPN9* | Intronic |
| rs7176938 | 15 | 75817115 | 0.93 | 0.97 | T | C | 0.61 | 0.26 | 0.38 | 0.26 | 0 | 0 | . | . | . | . | . | *PTPN9* | Intronic |
| rs8036758 | 15 | 75817562 | 0.93 | 0.97 | G | A | 0.59 | 0.26 | 0.38 | 0.26 | 0 | 0 | . | . | . | . | . | *PTPN9* | Intronic |
| rs8024244 | 15 | 75822230 | 0.93 | 0.97 | G | A | 0.55 | 0.26 | 0.38 | 0.26 | 0 | 0 | . | . | . | LIV.A,14_Enh | . | *PTPN9* | Intronic |
| rs4886715 | 15 | 75831449 | 0.91 | 0.96 | C | T | 0.61 | 0.26 | 0.38 | 0.26 | 0 | 0 | . | . | . | . | . | *PTPN9* | Intronic |
| rs60972074 | 15 | 75833786 | 0.88 | 0.95 | C | T | 0.6 | 0.26 | 0.37 | 0.26 | 0 | 0 | . | . | . | ST.MUC,14_Enh | . | *PTPN9* | Intronic |
| rs7163907 | 15 | 75845097 | 0.88 | 0.95 | T | C | 0.59 | 0.26 | 0.38 | 0.26 | 0 | 0 | . | K562,7_Weak_Enhancer | DUO.MUC61,6_TssD2 | MSC.ADIPC,11 EnhWk1;GAS,11 EnhWk1;LIV.A,11 EnhW | AWG,K562,None;Duke,LNCaP,androgen;Duke,Osteobl,None | *PTPN9* | Intronic |
| rs28783769 | 15 | 75849009 | 0.88 | 0.95 | C | A | 0.6 | 0.26 | 0.38 | 0.26 | 0 | 0 | . | . | . | . | . | *PTPN9* | Intronic |
| rs4393535 | 15 | 75851293 | 0.8 | 0.92 | C | T | 0.56 | 0.25 | 0.37 | 0.26 | 0 | 0 | . | . | . | . | . | *PTPN9* | Intronic |
| rs4886716 | 15 | 75856022 | 0.87 | 0.94 | G | C | 0.57 | 0.26 | 0.38 | 0.26 | 0 | 0 | . | . | . | . | . | *PTPN9* | Intronic |
| rs7184046 | 15 | 75866150 | 0.84 | 0.93 | G | C | 0.61 | 0.26 | 0.38 | 0.26 | 0 | 0 | GM12878,2_Weak_Promoter | H1,6_Weak_Enhancer;HMEC,7_Weak_Enhancer;K562,6_Weak_Enhancer | IPS.20,6 TssD2;IPS.18,6 TssD2;HUES64,6 TssD2;IPS.15,6 TssD2;HUES48,6 TssD2 | CD15.P,11 EnhWk1;MUS.SC,11 EnhWk1;ADI.MSC,11 Enh | AWG,Th1,None | *PTPN9* | Intronic |
| **rs76535198** | **16** | **71892498** | - | - | A | C | 0.1 | 0.14 | 0.31 | 0.13 | 0 | 0 | . | . | . | ADI.NUC,11 EnhWk1;SK.MUS62,14 Enh;SK.MUS63,14 Er | . | *ATXN1L* | Intronic |
| rs113070273 | 16 | 71873883 | 0.87 | 1 | C | T | 0.1 | 0.14 | 0.28 | 0.13 | 0 | 0 | . | . | . | . | . | *ATXN1L* | . |
| rs112121746 | 16 | 71874983 | 0.85 | 1 | G | A | 0.1 | 0.14 | 0.28 | 0.13 | 0 | 0 | . | GM12878,7_Weak_Enhancer | . | . | . | *ATXN1L* | . |
| rs113584314 | 16 | 71882860 | 0.89 | 1 | G | A | 0.1 | 0.14 | 0.28 | 0.14 | 0 | 0 | . | . | . | SK.MUS62,9 TxEnhG1;SK.MUS63,9 TxEnhG1;IPS.DF19,9 | . | *ATXN1L* | Intronic |
| rs16973477 | 16 | 71887381 | 0.87 | 1 | A | G | 0.1 | 0.16 | 0.28 | 0.16 | 1 | 1 | . | GM12878,4_Strong_Enhancer | . | CCIP.LSMPTP,9 TxEnhG1;CC.TPC,9 TxEnhG1;ADI.NUC, | AWG,MCF-7,None;UW,Adult_CD4_Th0,None | *ATXN1L* | 3'-UTR; intronic |
| rs200761004 | 16 | 71891983 | 0.93 | 1 | T | TG | 0.09 | 0.13 | 0.29 | 0.12 | 0 | 0 | . | . | . | CC.TPC,9 TxEnhG1;ADI.NUC,11 EnhWk1;SK.MUS62,14 | . | *ATXN1L* | Intronic |
| rs142336745 | 16 | 71905997 | 0.92 | 0.97 | G | A | 0.1 | 0.14 | 0.31 | 0.14 | 0 | 0 | . | GM12878,7_Weak_Enhancer | . | . | . | *ZNF821* | Intronic |
| rs3794691 | 16 | 71908441 | 0.97 | 1 | T | C | 0.1 | 0.14 | 0.3 | 0.14 | 0 | 0 | . | . | . | . | . | *ZNF821* | Intronic |
| rs112452788 | 16 | 71911198 | 0.98 | 1 | C | T | 0.1 | 0.13 | 0.3 | 0.14 | 0 | 0 | . | . | . | . | . | *ZNF821* | Intronic |
| rs112012852 | 16 | 71911785 | 0.96 | 1 | T | A | 0.1 | 0.14 | 0.3 | 0.14 | 0 | 0 | . | . | . | . | . | *ZNF821* | Intronic |
| rs149288672 | 16 | 71916281 | 0.82 | 1 | CAG | C | 0.1 | 0.09 | 0.27 | 0.13 | 0 | 0 | GM12878,2_Weak_Promoter | K562,7_Weak_Enhancer;Huvec,6_Weak_Enhancer | R.MUC29,4 TssWk;CCC.TREGP,4 TssWk;R.SMUS,4 TssWk;PANC.I,4 TssWk;BN. | CD34.MBP1480,11 EnhWk1;PFM.2,11 EnhWk1;CD8.NP,11 | . | *ZNF821* | Intronic |
| rs142428477 | 16 | 71936253 | 0.81 | 0.91 | A | G | 0.11 | 0.14 | 0.31 | 0.14 | 0 | 0 | . | GM12878,6_Weak_Enhancer | . | CD4.NP,11 EnhWk1;CD8.NP,11 EnhWk1;CD4.MP,11 Enh | . | *KIAA0174* | Intronic |
| **rs12481286** | **20** | **52287610** | - | - | G | T | 0.05 | 0.22 | 0.35 | 0.23 | 0 | 0 | . | . | . | R.MUC29,11_EnhWk1 | . | *ZNF217* | . |
| rs6063975 | 20 | 52278275 | 0.92 | 0.98 | C | G | 0.05 | 0.22 | 0.35 | 0.23 | 0 | 0 | . | GM12878,4_Strong_Enhancer;HepG2,4_Strong_Enhancer;NHLF,6_Weak_Enhancer | CD8.MP,6_TssD2 | SPL,14 Enh;GAS,14 Enh;HUES48,11 EnhWk1;ESO,14 Enh; | AWG,MCF-7,None;Duke,8988T,None;Duke,AoSMC,None;Duke,Fibrobl,None;Duke,GM12892,None;Duke,MCF-7,Hypoxia LacAcid | *ZNF217* | . |
| rs12625046 | 20 | 52282887 | 0.82 | 0.96 | T | C | 0.24 | 0.28 | 0.32 | 0.32 | 0 | 0 | . | . | . | R.MUC29,11_EnhWk1 | AWG,MCF-7,None | *ZNF217* | . |
| rs57942030 | 20 | 52283527 | 0.84 | 1 | T | A | 0.25 | 0.27 | 0.31 | 0.31 | 0 | 0 | . | . | . | . | . | *ZNF217* | . |
| rs113200595 | 20 | 52285202 | 0.89 | 0.97 | T | G | 0.05 | 0.23 | 0.34 | 0.27 | 0 | 0 | . | . | . | . | . | *ZNF217* | . |
| rs112582910 | 20 | 52285250 | 0.99 | 1 | A | G | 0.05 | 0.21 | 0.34 | 0.23 | 0 | 0 | . | . | . | . | . | *ZNF217* | . |
| rs12624860 | 20 | 52285882 | 1 | 1 | C | G | 0.05 | 0.21 | 0.35 | 0.23 | 0 | 0 | . | HepG2,7_Weak_Enhancer | . | R.MUC31,11 EnhWk1;DUO.MUC61,14 Enh;DUO.SMUS,1 | AWG,HMEC,None | *ZNF217* | . |
| rs200998131 | 20 | 52287492 | 0.92 | 0.98 | A | AG | 0.08 | 0.2 | 0.34 | 0.22 | 0 | 0 | . | . | . | . | . | *ZNF217* | . |
| rs6022654 | 20 | 52289243 | 0.98 | 0.99 | C | A | 0.36 | 0.32 | 0.35 | 0.34 | 0 | 0 | . | Huvec,7_Weak_Enhancer;HSMM,5_Strong_Enhancer | MUS.SC,3_TssA | ADI.MSC,11 EnhWk1;CHON.BMMSC,11 EnhWk1;PFF.1,1 | AWG,HSMM,None;AWG,HUVEC,None;AWG,HeLa-S3,None;AWG,MCF-7,None;Duke,Gliobla,None;Duke,HPDE6-E6E7,None;Duke | *ZNF217* | . |
| rs6022655 | 20 | 52289354 | 0.98 | 0.99 | T | C | 0.36 | 0.32 | 0.35 | 0.34 | 0 | 0 | . | Huvec,7_Weak_Enhancer;HSMM,5_Strong_Enhancer | MUS.SC,3_TssA | ADI.MSC,11 EnhWk1;CHON.BMMSC,11 EnhWk1;PFF.1,1 | AWG,A549,None;AWG,HSMM,None;AWG,HSMMtube,None;Duke,Fibrobl,None;UW,HA-sp,None;UW,HAc,None;UW,HFF-Myc,No | *ZNF217* | . |
| rs6022656 | 20 | 52289363 | 0.98 | 0.99 | T | C | 0.36 | 0.32 | 0.35 | 0.34 | 0 | 0 | . | Huvec,7_Weak_Enhancer;HSMM,5_Strong_Enhancer | MUS.SC,3_TssA | ADI.MSC,11 EnhWk1;CHON.BMMSC,11 EnhWk1;PFF.1,1 | AWG,A549,None;AWG,HSMM,None;AWG,HSMMtube,None;Duke,Fibrobl,None;UW,AG09309,None;UW,HA-sp,None;UW,HAEpiC | *ZNF217* | . |
| rs6068614 | 20 | 52289702 | 0.85 | 0.99 | C | A | 0.03 | 0.2 | 0.31 | 0.2 | 0 | 0 | . | HSMM,7_Weak_Enhancer | MUS.SC,2_TssF | PFF.1,11 EnhWk1;PFM.2,11 EnhWk1;BM.MSC,11 EnhWk1 | . | *ZNF217* | . |
| rs6013752 | 20 | 52292517 | 0.98 | 0.99 | T | C | 0.26 | 0.3 | 0.34 | 0.34 | 0 | 0 | . | . | . | . | . | *ZNF217* | . |
| rs13036868 | 20 | 52294195 | 0.81 | 0.97 | A | T | 0.03 | 0.19 | 0.31 | 0.2 | 0 | 0 | . | HepG2,4 Strong Enhancer;HSMM,7 Weak Enhancer;H1,7 Weak Enhancer;Huvec,7 Weak Enhancer;K562,4 Strong En | . | BM.MSC,11 EnhWk1;PANC,12 EnhWk2;CD34.P,11 EnhW | AWG,HUVEC,None;Duke,AoSMC,None;Duke,Myometr,None;Duke,PanIsletD,None;UW,AG04449,None;UW,AG04450,None;UW,AC | *ZNF217* | . |
| rs13039563 | 20 | 52296849 | 0.93 | 0.99 | G | A | 0.04 | 0.22 | 0.33 | 0.23 | 0 | 0 | . | HepG2,4_Strong_Enhancer;H1,7_Weak_Enhancer;Huvec,7_Weak_Enhancer;NHLF,7_Weak_Enhancer | HD.CD184EC,4_TssWk | MSC.ADIPC,11 EnhWk1;HUES48,11 EnhWk1;CC.TPC,11 | AWG,MCF-7,None;Duke,HeLa-S3,IFNa4h;Duke,ProgFib,None | *ZNF217* | . |
| **rs35418111** | **21** | **47856670** | - | - | G | A | 0.04 | 0.11 | 0.19 | 0.07 | 0 | 0 | . | . | . | BN.AC,9 TxEnhG1;CD3.P,9 TxEnhG1;BN.SN,9 TxEnhG1; | . | *PCNT* | Intronic |
| rs7275926 | 21 | 47840234 | 0.81 | 0.93 | C | T | 0.1 | 0.14 | 0.2 | 0.13 | 0 | 0 | . | . | . | SPL,9_TxEnhG1 | Duke,Melano,None;Duke,Osteobl,None | *PCNT* | Intronic |
| rs13053115 | 21 | 47841597 | 0.81 | 0.93 | G | A | 0.07 | 0.14 | 0.2 | 0.13 | 0 | 0 | . | . | . | SPL,9_TxEnhG1;IPS.DF19,9_TxEnhG1 | Duke,PanIslets,None | *PCNT* | Intronic |
| rs8134832 | 21 | 47845242 | 0.81 | 0.94 | A | G | 0.55 | 0.24 | 0.2 | 0.23 | 0 | 0 | . | H1,7_Weak_Enhancer | CD8.MP,2_TssF | CD3.P,13 EnhA;H1.DNP,14 Enh;CD4.NP,13 EnhA;CCCRA | AWG,LNCaP,None;AWG,Th1,None | *PCNT* | Intronic |
| rs71326328 | 21 | 47848908 | 0.83 | 0.94 | G | A | 0.08 | 0.14 | 0.2 | 0.13 | 0 | 0 | . | . | . | BN.AC,9_TxEnhG1 | Duke,MCF-7,Hypoxia_LacAcid;Duke,Medullo,None | *PCNT* | Intronic |
| rs57385578 | 21 | 47850178 | 0.93 | 0.99 | C | T | 0.01 | 0.11 | 0.18 | 0.07 | 0 | 0 | . | . | . | BN.AC,9_TxEnhG1 | AWG,Th1,None;Duke,Hepatocytes,None;Duke,Medullo,None;Duke,Myometr,None | *PCNT* | Intronic |
| rs9983522 | 21 | 47851796 | 0.84 | 0.94 | G | A | 0.07 | 0.14 | 0.2 | 0.13 | 0 | 0 | . | . | . | BN.AC,9_TxEnhG1;CD3.P,9_TxEnhG1;CD8.NP,14_Enh | AWG,H1-hESC,None;AWG,HMEC,None;AWG,LNCaP,None;Duke,Hepatocytes,None;Duke,iPS,None;Duke,pHTE,None | *PCNT* | SYN |
| rs9712582 | 21 | 47853022 | 0.84 | 0.94 | C | T | 0.1 | 0.14 | 0.2 | 0.13 | 0 | 0 | . | . | . | CD3.P,9_TxEnhG1 | . | *PCNT* | Intronic |
| rs8126917 | 21 | 47855137 | 0.82 | 0.94 | T | G | 0.07 | 0.14 | 0.2 | 0.13 | 0 | 0 | . | . | . | . | . | *PCNT* | Intronic |
| rs17371795 | 21 | 47855876 | 0.83 | 0.94 | A | G | 0.07 | 0.14 | 0.2 | 0.13 | 0 | 0 | . | . | . | CD8.NP,11 EnhWk1;BN.AC,9 TxEnhG1;CD3.P,9 TxEnhG1 | UW,Th2,None | *PCNT* | SYN |
| rs7276409 | 21 | 47857440 | 0.84 | -0.95 | C | T | 0.9 | 0.75 | 0.8 | 0.81 | 0 | 0 | . | . | . | BN.AC,9 TxEnhG1;CD3.P,9 TxEnhG1;CD8.NP,13 EnhA;IP | . | *PCNT* | Intronic |
| rs9981192 | 21 | 47858774 | 0.83 | -0.95 | T | C | 0.91 | 0.75 | 0.8 | 0.8 | 0 | 0 | . | . | . | . | . | *PCNT* | Intronic |
| rs10854482 | 21 | 47862188 | 0.81 | -0.93 | T | C | 0.89 | 0.75 | 0.8 | 0.81 | 0 | 0 | . | . | . | BN.CC,9_TxEnhG1;ES.WA7,14_Enh | . | *PCNT* | Intronic |
| rs2839262 | 21 | 47862624 | 0.83 | -0.94 | A | G | 0.91 | 0.76 | 0.8 | 0.81 | 0 | 0 | . | . | . | ES.WA7,14 Enh;BN.AG,9 TxEnhG1;BN.ITL,9 TxEnhG1;CI | Duke,Chorion,None | *PCNT* | Intronic |
| rs1010111 | 21 | 47871585 | 0.8 | -0.94 | G | A | 0.73 | 0.7 | 0.8 | 0.73 | 0 | 0 | . | . | . | BN.HM150,14 Enh;SK.MUS62,11 EnhWk1;SK.MUS,13 Enl | Duke,Fibrobl,None;UW,AG04450,None | *PCNT* | . |
| rs7280077 | 21 | 47872606 | 0.82 | -0.94 | G | A | 0.91 | 0.76 | 0.8 | 0.81 | 0 | 0 | . | . | . | GAS,11 EnhWk1;BN.AG,14 Enh;BN.AC,14 Enh;BN.ITL,11 | AWG,HMEC,None;Duke,Urothelia,UT189 | *DIP2A* | . |
| rs8130538 | 21 | 47876915 | 0.82 | -0.94 | T | C | 0.82 | 0.75 | 0.8 | 0.81 | 0 | 0 | GM12878,2 Weak Promoter;HepC | HMEC,5_Strong_Enhancer;Huvec,7_Weak_Enhancer;NHEK,5_Strong_Enhancer;NHLF,6_Weak_Enhancer | SK.MUS,2_TssF;HRT.FE,2_TssF;SK.MUS62,2_TssF | GAS,11 EnhWk1;PFF.2,14 Enh;BR.MYO,11 EnhWk1;BN.A | AWG,NHEK,None;AWG,Th1,None;UW,PrEC,None | *DIP2A* | . |
| rs2078203 | 21 | 47881309 | 0.81 | -0.94 | A | G | 0.74 | 0.7 | 0.8 | 0.74 | 1 | 0 | . | HepG2,6_Weak_Enhancer;GM12878,6_Weak_Enhancer | . | CCCRA.NP,14 Enh;CD8.NP,11 EnhWk1;CCIP.LSTP,11 En | Duke,Osteobl,None | *DIP2A* | Intronic |
| rs1981330 | 21 | 47881635 | 0.81 | -0.94 | A | G | 0.88 | 0.72 | 0.8 | 0.74 | 0 | 0 | . | . | . | CCCRA.NP,14 Enh;CD8.NP,11 EnhWk1;CCIP.LSTP,11 En | . | *DIP2A* | Intronic |
| **rs34331122** | **22** | **19762428** | - | - | C | CTT | 0.71 | 0.61 | 0.59 | 0.42 | 0 | 0 | . | K562,4_Strong_Enhancer | . | CD8.MP,14_Enh;IPS.DF19,11_EnhWk1;CD4.MP,14_Enh | . | *TBX1* | Intronic |
| rs200354114 | 22 | 19764608 | 0.96 | 0.99 | T | TGTAA | 0.32 | 0.51 | 0.58 | 0.34 | 0 | 0 | . | K562,7_Weak_Enhancer | . | . | . | *TBX1* | Intronic |
