# Supplementary material for: Identification of novel breast cancer susceptibility loci in meta-analyses conducted among Asian and European descendants
Source: Nat Commun. 2020 Mar 5;11:1217. doi: 10.1038/s41467-020-15046-w (PMC7057957; doi:10.1038/s41467-020-15046-w)
Supplement: Supplementary file 7 — Supplementary Data 4 [file 41467_2020_15046_MOESM7_ESM.pdf]

Supplementary Data 4. Regulatory functional annotation of SNPs in high LD (r^2>0.8) with newly-associated SNPs in European populations using Haploreg

SNP	Chr	BP	LD r^2	D'	ref	alt	AFR	AMR	ASN	EUR	GERP cons	SiPhy cons
**rs72906468**	**1**	**17772093**	-	-	A	T	0.21	0.26	0.36	0.2	0	0
rs34548121	1	17725627	0.84	0.93	G	A	0.15	0.25	0.36	0.2	0	0
rs34167614	1	17728072	0.84	0.93	G	A	0.12	0.26	0.36	0.2	0	0
rs717612	1	17729612	0.84	0.93	C	G	0.14	0.26	0.36	0.2	0	0
rs12093070	1	17742024	0.84	0.93	T	C	0.15	0.25	0.37	0.2	0	0
rs6586541	1	17753749	0.94	0.98	C	T	0.18	0.27	0.37	0.2	0	0
rs7549366	1	17757937	0.91	0.96	C	T	0.2	0.27	0.37	0.2	0	0
rs2883272	1	17773159	0.96	1	G	A	0.2	0.23	0.05	0.19	0	0
rs6692736	1	17773914	0.85	0.99	T	C	0.2	0.22	0.05	0.18	0	0
rs35016926	1	17774479	0.85	0.99	C	A	0.21	0.22	0.05	0.18	0	0
**rs3790585**	**1**	**46023356**	-	-	A	T	0.21	0.22	0.31	0.15	0	0
rs514595	1	46036960	0.87	-0.95	T	C	0.7	0.77	0.7	0.85	0	0
rs518216	1	46037357	0.85	-0.95	C	T	0.7	0.77	0.65	0.85	0	0
rs659437	1	46037394	0.85	-0.95	T	C	0.69	0.77	0.65	0.85	0	0
rs499600	1	46039077	0.87	-0.94	T	G	0.75	0.78	0.7	0.85	0	0
rs35506410	1	46039202	0.82	-0.94	GC	G	0.69	0.77	0.65	0.85	0	0
**rs2758598**	**1**	**1.56E+08**	-	-	G	A	0.18	0.28	0.15	0.35	0	0
rs6427304	1	156156789	0.86	0.94	A	G	0.71	0.32	0.15	0.36	0	0
rs11587860	1	156156951	0.85	0.93	G	C	0.67	0.32	0.15	0.36	0	0
rs2758615	1	156158367	0.84	0.92	T	C	0.7	0.32	0.15	0.35	0	0
rs2842864	1	156159217	0.84	0.92	G	A	0.67	0.32	0.15	0.35	0	0
rs2842865	1	156159532	0.84	0.92	G	A	0.64	0.32	0.15	0.35	0	0
rs2758616	1	156160358	0.81	0.9	T	C	0.67	0.32	0.15	0.35	0	0
rs2842869	1	156160696	0.84	0.92	A	G	0.65	0.31	0.15	0.35	0	0
rs2758618	1	156160892	0.84	0.92	T	C	0.64	0.31	0.15	0.35	0	0
rs2758619	1	156162559	0.86	0.92	A	G	0.67	0.32	0.15	0.35	0	0
rs2540183	1	156164885	0.89	0.96	C	G	0.18	0.28	0.15	0.35	0	0
rs3001790	1	156165290	0.86	0.93	T	G	0.67	0.32	0.15	0.35	0	0
rs2984615	1	156165317	0.86	0.93	G	C	0.67	0.32	0.15	0.35	1	1
rs2842882	1	156165342	0.86	0.93	T	G	0.67	0.32	0.15	0.35	1	1
rs2842857	1	156168736	0.87	0.94	T	C	0.68	0.32	0.15	0.36	0	0
rs2072499	1	156169610	0.92	0.98	A	G	0.67	0.33	0.15	0.36	0	0
rs2253677	1	156171296	0.88	0.94	C	G	0.67	0.32	0.15	0.35	0	0
rs2253809	1	156172125	0.92	0.98	T	C	0.67	0.32	0.15	0.36	0	0
rs2266514	1	156173685	0.88	0.94	T	C	0.67	0.32	0.15	0.35	0	0
rs2736605	1	156174475	0.88	0.94	T	C	0.64	0.32	0.15	0.35	0	0
rs200096331	1	156174831	0.83	0.92	GA	G	0.64	0.31	0.14	0.35	0	0
rs2241108	1	156181007	0.92	0.98	C	G	0.67	0.33	0.16	0.36	0	0
rs2853641	1	156181770	0.92	0.98	A	G	0.67	0.32	0.16	0.36	0	0
rs2241107	1	156182710	0.92	0.98	T	C	0.67	0.32	0.16	0.36	0	0
rs1060604	1	156184268	0.96	0.98	T	C	0.19	0.29	0.16	0.35	0	0
rs1137703	1	156184458	0.91	0.97	T	C	0.67	0.32	0.16	0.36	0	0
rs2244144	1	156184598	0.96	0.98	G	A	0.19	0.29	0.16	0.35	0	0
rs2540175	1	156184747	0.91	0.97	T	C	0.61	0.32	0.15	0.36	0	0
rs2853643	1	156184831	0.96	0.98	G	A	0.19	0.29	0.16	0.35	0	0
rs7534434	1	156186615	0.84	0.93	A	G	0.63	0.31	0.16	0.35	0	0
rs2475757	1	156186634	0.82	0.97	C	G	0.42	0.26	0.15	0.32	0	0
rs2540173	1	156188214	0.96	1	A	G	0.67	0.32	0.16	0.36	0	0
rs2853646	1	156189626	0.95	0.99	C	T	0.19	0.28	0.15	0.35	0	0
rs7529564	1	156189793	0.88	0.94	T	C	0.62	0.31	0.16	0.35	0	0
rs6427307	1	156190083	0.94	1	A	G	0.68	0.33	0.16	0.37	0	0
rs2247476	1	156190280	0.8	0.99	C	A	0.08	0.2	0.03	0.31	0	0
rs887953	1	156191149	0.98	1	T	C	0.46	0.3	0.16	0.36	0	0
rs909269	1	156192028	0.8	0.99	C	T	0.01	0.2	0.03	0.31	0	0
rs2247937	1	156194363	0.9	0.99	C	T	0.16	0.28	0.15	0.33	0	0
rs2758600	1	156195205	0.99	0.99	C	T	0.18	0.28	0.15	0.35	0	0
rs2248074	1	156195888	0.97	0.99	C	T	0.39	0.3	0.16	0.35	0	0
rs2248080	1	156195971	0.93	0.99	T	A	0.17	0.27	0.15	0.34	0	0
rs2736613	1	156196016	0.97	0.98	T	C	0.42	0.3	0.16	0.35	0	0
rs2984613	1	156197380	0.87	0.96	C	T	0.22	0.3	0.32	0.37	0	0
rs3001789	1	156197614	0.87	0.96	T	G	0.42	0.32	0.33	0.37	0	0
rs2758603	1	156198994	0.87	0.96	T	C	0.42	0.32	0.33	0.37	0	0
rs143621173	1	156200354	0.87	0.95	T	TGA	0.42	0.32	0.33	0.36	0	0
rs2758605	1	156200445	0.87	0.96	G	C	0.42	0.32	0.33	0.37	0	0
rs2842870	1	156200671	0.88	0.97	T	C	0.53	0.34	0.33	0.37	0	0
rs1052053	1	156202173	0.84	0.96	A	G	0.71	0.36	0.33	0.38	1	1
rs2736609	1	156202640	0.88	0.97	C	T	0.25	0.32	0.34	0.37	0	0
rs2758607	1	156202759	0.89	0.97	G	A	0.25	0.32	0.33	0.37	0	0
rs2251636	1	156202809	0.85	0.95	G	C	0.48	0.33	0.33	0.37	0	0
rs2758608	1	156202860	0.89	0.97	A	T	0.25	0.32	0.33	0.37	0	0
rs2758609	1	156202868	0.89	0.97	G	A	0.25	0.32	0.33	0.37	0	0
rs2251847	1	156204047	0.87	0.96	G	A	0.46	0.32	0.33	0.37	0	0
rs2842873	1	156204653	0.85	0.96	C	T	0.7	0.35	0.33	0.37	0	0
rs2736611	1	156205383	0.8	0.91	A	C	0.51	0.31	0.33	0.35	0	0
**rs6756513**	**2**	**70172587**	-	-	G	A	0.32	0.18	0.28	0.29	0	0
**rs73006998**	**3**	**1.5E+08**	-	-	G	A	0.32	0.11	0.32	0.02	0	0
rs6789306	3	150461224	0.81	1	A	G	0.36	0.12	0.36	0.02	0	0
rs7615125	3	150461900	1	1	C	T	0.32	0.11	0.32	0.02	0	0
rs7615292	3	150461933	1	1	G	A	0.32	0.11	0.32	0.02	0	0
rs73006999	3	150465010	0.81	1	G	A	0.36	0.12	0.36	0.02	1	0
rs73008903	3	150465053	0.81	1	C	T	0.36	0.12	0.36	0.02	0	0
rs73008905	3	150465435	0.81	1	T	C	0.35	0.12	0.36	0.02	0	0
rs73008907	3	150465456	0.81	1	G	A	0.36	0.11	0.36	0.02	0	0
rs55899470	3	150465739	0.81	1	G	A	0.35	0.12	0.36	0.02	0	0
rs57824095	3	150465887	0.81	1	G	A	0.35	0.12	0.36	0.02	0	0
rs1879420	3	150466564	0.92	1	T	C	0.1	0.09	0.32	0.02	0	0
rs1991781	3	150468433	0.81	1	G	A	0.32	0.12	0.36	0.02	0	0
rs2116684	3	150471737	0.85	0.92	G	C	0.23	0.1	0.33	0.02	0	0
rs57359526	3	150472042	0.85	0.92	T	C	0.23	0.1	0.33	0.02	0	0
rs61628582	3	150472160	0.85	0.92	C	T	0.2	0.1	0.33	0.02	0	0
rs2166061	3	150472262	0.81	1	G	C	0.49	0.13	0.41	0.02	0	0
rs2120596	3	150472390	0.92	1	G	A	0.14	0.1	0.33	0.02	0	0
rs60092981	3	150472999	0.92	1	T	A	0.14	0.1	0.33	0.02	0	0
rs57965761	3	150473071	0.85	0.92	G	A	0.24	0.1	0.37	0.02	0	0
rs60538652	3	150473606	0.92	1	A	T	0.18	0.1	0.37	0.02	0	0
rs60523415	3	150477002	0.85	0.92	C	G	0.16	0.1	0.34	0.02	0	0
rs75942495	3	150477877	0.85	0.92	G	A	0.16	0.1	0.34	0.02	0	0
rs1915365	3	150479070	0.85	0.92	C	G	0.2	0.1	0.38	0.02	0	0
rs1838337	3	150479816	0.85	0.92	G	T	0.2	0.1	0.38	0.02	0	0
rs1444197	3	150480057	0.85	0.92	A	T	0.2	0.1	0.38	0.02	0	0
rs74950732	3	150482621	0.85	0.92	C	T	0.16	0.1	0.34	0.02	0	0
rs76801042	3	150482720	0.85	0.92	A	C	0.16	0.1	0.34	0.02	0	0
rs79914414	3	150483746	0.85	0.92	A	G	0.16	0.1	0.35	0.02	0	0
rs74549747	3	150484689	0.85	0.92	A	G	0.16	0.1	0.34	0.02	0	0
rs16862842	3	150484844	0.85	0.92	T	C	0.16	0.1	0.34	0.02	0	0
rs79709238	3	150488477	0.85	0.92	A	C	0.16	0.1	0.34	0.02	0	0
rs41372346	3	150489116	0.85	0.92	C	G	0.16	0.1	0.35	0.02	0	0
rs34901265	3	150489317	0.85	0.92	G	C	0.21	0.11	0.35	0.02	0	0
rs77431731	3	150489911	0.85	0.92	T	G	0.16	0.1	0.35	0.02	0	0
rs59741644	3	150496188	0.85	0.92	C	A	0.13	0.1	0.36	0.02	0	0
rs73010967	3	150498056	0.85	0.92	T	C	0.24	0.12	0.37	0.02	0	0
rs6803830	3	150510579	0.85	0.92	A	G	0.29	0.12	0.41	0.02	0	0
rs78552868	3	150511031	0.85	0.92	G	A	0.1	0.1	0.37	0.02	0	0
rs77437769	3	150513864	0.85	0.92	C	T	0.37	0.12	0.4	0.02	0	0
rs78943763	3	150513865	0.85	0.92	A	G	0.36	0.12	0.4	0.02	0	0
rs149408779	3	150514531	0.85	0.92	CCTT	C	0.1	0.09	0.37	0.02	0	0
rs201959055	3	150514705	0.85	0.92	T	TA	0.23	0.1	0.32	0.02	0	0
rs113840938	3	150514718	0.85	0.92	T	C	0.4	0.13	0.39	0.02	0	0
rs76936100	3	150518718	0.85	0.92	A	G	0.15	0.1	0.32	0.02	0	0
rs58403524	3	150520960	0.85	0.92	G	C	0.15	0.1	0.31	0.02	0	0
**rs11281251**	**3**	**1.57E+08**	-	-	T	TTGTGAC	0.34	0.69	0.82	0.58	0	0
rs199589043	3	156518663	0.9	0.99	G	GGT	0.37	0.7	0.84	0.6	0	0
rs1835372	3	156519027	0.91	1	C	T	0.36	0.7	0.84	0.61	0	0
rs1430411	3	156525396	0.91	1	T	A	0.36	0.7	0.84	0.61	0	0
rs1430410	3	156525562	0.91	1	C	A	0.36	0.7	0.84	0.61	0	0
rs9812169	3	156528401	0.91	1	G	A	0.36	0.7	0.84	0.61	1	0
rs7641929	3	156529394	0.91	1	C	G	0.36	0.7	0.84	0.61	1	1
rs12492804	3	156532145	0.91	1	T	A	0.36	0.7	0.84	0.61	0	0
rs12497539	3	156533937	0.9	0.97	A	C	0.36	0.7	0.82	0.59	0	0
rs59717073	3	156534245	0.91	1	A	G	0.36	0.7	0.83	0.61	0	0
rs11717133	3	156534581	0.91	1	T	C	0.36	0.7	0.83	0.61	0	0
rs63423161	3	156538670	0.91	1	A	G	0.36	0.7	0.83	0.61	0	0
rs9842198	3	156539204	0.9	0.99	G	C	0.36	0.7	0.83	0.6	0	0
rs9858861	3	156542637	0.89	0.99	G	C	0.36	0.7	0.84	0.61	0	0
rs13072896	3	156542807	0.87	0.96	G	C	0.36	0.69	0.84	0.6	0	0

SNP	Promoter (ENCODE)	Enhancer (ENCODE)	Promoter (Roadmap)
**rs72906468**	.	.	.
rs34548121	.	.	.
rs34167614	.	H1,7_Weak_Enhancer;K562,7_Weak_Enhancer	.
rs717612	.	NHEK,6_Weak_Enhancer;HMEC,6_Weak_Enhancer	.
rs12093070	.	.	.
rs6586541	.	.	.
rs7549366	.	.	CD34.MBP1508,4_TssWk
rs2883272	.	.	.
rs6692736	.	HSMM,6_Weak_Enhancer	.
rs35016926	.	HSMM,7_Weak_Enhancer	ADI.MSC,6_TssD2
**rs3790585**	.	HMEC,5 Strong Enhancer;NHEK,5 Strong Enhancer;GM12878,4 Strong Enhancer;Huvec,7 Weak Enhanc	.
rs514595	.	.	.
rs518216	.	.	.
rs659437	.	.	.
rs499600	.	H1,6_Weak_Enhancer;K562,4_Strong_Enhancer	.
rs35506410	.	H1,6_Weak_Enhancer;K562,4_Strong_Enhancer	.
**rs2758598**	.	.	.
rs6427304	.	.	.
rs11587860	.	.	.
rs2758615	.	.	.
rs2842864	.	HepG2,7_Weak_Enhancer	.
rs2842865	.	HepG2,7_Weak_Enhancer	.
rs2758616	.	HepG2,7_Weak_Enhancer	.
rs2842869	.	HepG2,7_Weak_Enhancer	.
rs2758618	.	HepG2,7_Weak_Enhancer	.
rs2758619	.	K562,4_Strong_Enhancer;GM12878,7_Weak_Enhancer;Huvec,7_Weak_Enhancer	PFM.2,4_TssWk
rs2540183	GM12878,2 Weak Promoter;K562,2 Weak Promoter;HSMM,2 Weak Promoter;I	NHLF,4_Strong_Enhancer;HepG2,6_Weak_Enhancer;NHEK,6_Weak_Enhancer	DUO.MUC61,2 TssF;SK.MUS62,6 TssD2;SK.MUS63,2 TssF;SK.MUS,2 TssF;CD15.P,2 TssF;BN.FE0,2 TssF;CD34.MBP1480,(
rs3001790	HSMM,2_Weak_Promoter;GM12878,2_Weak_Promoter;Huvec,2_Weak_Promoter	HepG2,7_Weak_Enhancer;HMEC,7_Weak_Enhancer	HD.CD184EC,4 TssWk;H1.BMP4DM,4 TssWk;IPS.18,4 TssWk;LNG.FE,4 TssWk;MSC.ADIPC,4 TssWk;LIV.A,6 TssD2;PFM
rs2984615	HSMM,2_Weak_Promoter;GM12878,2_Weak_Promoter;Huvec,2_Weak_Promoter	HepG2,7_Weak_Enhancer;HMEC,7_Weak_Enhancer	HD.CD184EC,4 TssWk;H1.BMP4DM,4 TssWk;IPS.18,4 TssWk;LNG.FE,4 TssWk;MSC.ADIPC,4 TssWk;LIV.A,6 TssD2;PFM
rs2842882	HSMM,2_Weak_Promoter;GM12878,2_Weak_Promoter;Huvec,2_Weak_Promoter	HepG2,7_Weak_Enhancer;HMEC,7_Weak_Enhancer	HD.CD184EC,4 TssWk;H1.BMP4DM,4 TssWk;IPS.18,4 TssWk;LNG.FE,4 TssWk;MSC.ADIPC,4 TssWk;LIV.A,6 TssD2;PFM
rs2842857	.	GM12878,4_Strong_Enhancer;HepG2,5_Strong_Enhancer	.
rs2072499	.	GM12878,4_Strong_Enhancer	.
rs2253677	GM12878,2_Weak_Promoter	.	CD15.P,2_TssF
rs2253809	GM12878,2_Weak_Promoter	.	CD15.P,2_TssF
rs2266514	.	Huvec,7_Weak_Enhancer	.
rs2736605	.	.	.
rs200096331	.	.	.
rs2241108	.	.	.
rs2853641	.	.	.
rs2241107	NHLF,1 Active Promoter;HSMM,1 Active Promoter;HepG2,1 Active Promoter;I	.	COL.MUC32,4 TssWk;BR.MYO,4 TssWk;PANC.I,4 TssWk;H9,3 TssA;BN.HM150,3 TssA;BN.MFL,3 TssA;CCC.TMP,3 TssA
rs1060604	.	GM12878,4 Strong Enhancer;K562,4 Strong Enhancer;H1,6 Weak Enhancer;HepG2,4 Strong Enhancer;H	CD34.MBP1562,2 TssF;SK.MUS62,6 TssD2;CHON.BMMSC,6 TssD2;LIV.A,2 TssF;PFM.2,2 TssF;R.MUC29,6 TssD2;CCIP.LS
rs1137703	.	K562,4 Strong Enhancer;HMEC,5 Strong Enhancer;NHEK,5 Strong Enhancer;GM12878,5 Strong Enhanc	CD34.MBP1562,6_TssD2;PFF.2,4_TssWk;LIV.A,6_TssD2;SK.MUS,2_TssF
rs2244144	.	HMEC,5_Strong_Enhancer;NHEK,5_Strong_Enhancer;K562,7_Weak_Enhancer	CD34.MBP1562,6_TssD2;PFF.2,4_TssWk;LIV.A,6_TssD2;SK.MUS,2_TssF
rs2540175	.	HMEC,5_Strong_Enhancer;NHEK,5_Strong_Enhancer;K562,7_Weak_Enhancer	.
rs2853643	.	NHEK,5_Strong_Enhancer;K562,7_Weak_Enhancer;HMEC,7_Weak_Enhancer	.
rs7534434	.	.	HRT.FE,2_TssF;LIV.A,1_TssP
rs2475757	.	.	HRT.FE,2_TssF;LIV.A,1_TssP
rs2540173	.	HMEC,5 Strong Enhancer;NHEK,4 Strong Enhancer;GM12878,5 Strong Enhancer;Huvec,5 Strong Enhan	CD15.P,2_TssF;CD34.MBP1562,2_TssF;CD34.MBP1480,6_TssD2
rs2853646	.	GM12878,7_Weak_Enhancer	.
rs7529564	.	.	.
rs6427307	.	.	.
rs2247476	.	.	.
rs887953	.	.	.
rs909269	.	.	.
rs2247937	.	.	.
rs2758600	.	GM12878,7_Weak_Enhancer	IMR90,4_TssWk
rs2248074	.	GM12878,4_Strong_Enhancer;HSMM,7_Weak_Enhancer	.
rs2248080	.	GM12878,4_Strong_Enhancer;HSMM,7_Weak_Enhancer	.
rs2736613	.	HSMM,7_Weak_Enhancer;GM12878,5_Strong_Enhancer	.
rs2984613	.	GM12878,7_Weak_Enhancer	.
rs3001789	.	GM12878,7_Weak_Enhancer	.
rs2758603	.	.	.
rs143621173	.	K562,5_Strong_Enhancer	.
rs2758605	.	K562,7_Weak_Enhancer	.
rs2842870	.	.	.
rs1052053	.	K562,7_Weak_Enhancer	.
rs2736609	.	K562,7_Weak_Enhancer;HSMM,7_Weak_Enhancer;NHLF,7_Weak_Enhancer	.
rs2758607	.	K562,7_Weak_Enhancer;HSMM,7_Weak_Enhancer;NHLF,7_Weak_Enhancer	.
rs2251636	.	K562,7_Weak_Enhancer;HSMM,7_Weak_Enhancer;NHLF,7_Weak_Enhancer;Huvec,7_Weak_Enhancer	.
rs2758608	.	K562,7_Weak_Enhancer;HSMM,7_Weak_Enhancer;NHLF,7_Weak_Enhancer;Huvec,7_Weak_Enhancer	.
rs2758609	.	K562,7_Weak_Enhancer;HSMM,7_Weak_Enhancer;NHLF,7_Weak_Enhancer;Huvec,7_Weak_Enhancer	.
rs2251847	.	NHLF,5_Strong_Enhancer;Huvec,7_Weak_Enhancer	.
rs2842873	.	.	.
rs2736611	.	K562,7_Weak_Enhancer	.
**rs6756513**	.	HMEC,5_Strong_Enhancer;NHEK,4_Strong_Enhancer;K562,5_Strong_Enhancer;Huvec,5_Strong_Enhancer	CD34.MBP1508,2_TssF;CD15.P,2_TssF;CD34.MBP1562,2_TssF
**rs73006998**	.	.	.
rs6789306	.	GM12878,5 Strong Enhancer;NHEK,4 Strong Enhancer;HMEC,4 Strong Enhancer;HSMM,7 Weak Enhan	PFK.2,2_TssF;PFK.3,2_TssF
rs7615125	.	GM12878,5 Strong Enhancer;NHEK,4 Strong Enhancer;HMEC,5 Strong Enhancer;HSMM,6 Weak Enhan	.
rs7615292	.	GM12878,5 Strong Enhancer;NHEK,4 Strong Enhancer;HSMM,6 Weak Enhancer;NHLF,7 Weak Enhanc	.
rs73006999	.	.	.
rs73008903	.	.	.
rs73008905	.	.	.
rs73008907	.	.	.
rs55899470	.	.	.
rs57824095	.	.	.
rs1879420	.	HSMM,7_Weak_Enhancer	.
rs1991781	.	NHEK,7_Weak_Enhancer	.
rs2116684	.	.	.
rs57359526	.	.	.
rs61628582	.	K562,7_Weak_Enhancer	.
rs2166061	.	K562,7_Weak_Enhancer	HD.CD184EC,6_TssD2
rs2120596	.	K562,7_Weak_Enhancer	HD.CD184EC,6_TssD2
rs60092981	.	.	HD.CD184EC,6_TssD2
rs57965761	.	.	.
rs60538652	.	.	.
rs60523415	GM12878,2_Weak_Promoter	NHEK,5_Strong_Enhancer;HMEC,7_Weak_Enhancer	CCIP.LSMPTP,2_TssF;CCIP.LSTP,3_TssA
rs75942495	GM12878,1_Active_Promoter;Huvec,2_Weak_Promoter	HepG2,4 Strong Enhancer;NHLF,6 Weak Enhancer;HMEC,6 Weak Enhancer;K562,4 Strong Enhancer;NI	MSC.ADIPC,4 TssWk;CD19.P,2 TssF;PFK.3,2 TssF;BN.MFL,6 TssD2;CD4.MP,2 TssF;R.SMUS,4 TssWk;PFK.2,2 TssF;CD3
rs1915365	GM12878,1 Active Promoter;Huvec,2 Weak Promoter;NHLF,2 Weak Promoter;I	H1,6_Weak_Enhancer	CD19.P,2 TssF;R.MUC31,2 TssF;BN.CC,6 TssD2;CC.TPC,2 TssF;CD34.MBP1562,2 TssF;CD34.P,2 TssF;MUS.SC,2 TssF;DU
rs1838337	GM12878,1 Active Promoter;HepG2,1 Active Promoter;HMEC,1 Active Promot	.	CD19.P,2 TssF;CD34.MBP1562,2 TssF;CD34.P,2 TssF;SK.MUS62,6 TssD2;CCIP.LSMPTP,3 TssA;SK.MUS,6 TssD2;CCC.TM
rs1444197	GM12878,1 Active Promoter;HepG2,1 Active Promoter;HMEC,1 Active Promot	.	CD19.P,2 TssF;CD34.P,2 TssF;SK.MUS62,6 TssD2;CCIP.LSMPTP,3 TssA;CCC.TMP,3 TssA;CCIP.LSTP,3 TssA;CD8.MP,3
rs74950732	H1,3_Poised_Promoter;GM12878,2_Weak_Promoter	HMEC,6 Weak Enhancer;HSMM,6 Weak Enhancer;Huvec,6 Weak Enhancer;HepG2,4 Strong Enhancer;N	CD34.C,2 TssF;BN.FE0,4 TssWk;CD34.MBP1549,4 TssWk;R.MUC29,6 TssD2;CCIP.LSMPTP,2 TssF;DUO.MUC61,6 TssD2;(
rs76801042	GM12878,2_Weak_Promoter	HepG2,4_Strong_Enhancer;K562,4_Strong_Enhancer	CD34.C,2 TssF;BN.FE0,4 TssWk;CD34.MBP1549,4 TssWk;R.MUC29,6 TssD2;CCIP.LSMPTP,2 TssF;DUO.MUC61,6 TssD2;(
rs79914414	.	HepG2,4 Strong Enhancer;GM12878,4 Strong Enhancer;K562,6 Weak Enhancer;NHLF,6 Weak Enhancer	CCIP.LSMPTP,2_TssF
rs74549747	.	HMEC,7 Weak Enhancer;NHEK,7 Weak Enhancer;GM12878,6 Weak Enhancer;HepG2,5 Strong Enhance	.
rs16862842	.	HMEC,7 Weak Enhancer;NHEK,7 Weak Enhancer;HepG2,5 Strong Enhancer;GM12878,7 Weak Enhance	.
rs79709238	.	HepG2,5_Strong_Enhancer	.
rs41372346	.	HepG2,5_Strong_Enhancer	.
rs34901265	.	HepG2,5_Strong_Enhancer	.
rs77431731	.	HepG2,7_Weak_Enhancer	.
rs59741644	.	.	.
rs73010967	.	.	.
rs6803830	.	.	.
rs78552868	.	.	.
rs77437769	.	.	.
rs78943763	.	.	.
rs149408779	.	.	.
rs201959055	.	.	.
rs113840938	.	.	.
rs76936100	.	.	.
rs58403524	.	.	.
**rs11281251**	.	.	.
rs199589043	.	.	.
rs1835372	.	K562,7_Weak_Enhancer	.
rs1430411	.	K562,7_Weak_Enhancer;NHEK,7_Weak_Enhancer;HMEC,5_Strong_Enhancer	.
rs1430410	.	K562,7_Weak_Enhancer;NHEK,7_Weak_Enhancer;HMEC,5_Strong_Enhancer	.
rs9812169	.	HMEC,5 Strong Enhancer;HepG2,6 Weak Enhancer;Huvec,7 Weak Enhancer;K562,6 Weak Enhancer;NH	.
rs7641929	.	HepG2,7 Weak Enhancer;Huvec,5 Strong Enhancer;HMEC,5 Strong Enhancer;K562,7 Weak Enhancer;NI	.
rs12492804	.	NHEK,5_Strong_Enhancer;GM12878,6_Weak_Enhancer;HMEC,5_Strong_Enhancer	.
rs12497539	GM12878,1 Active Promoter;NHLF,1 Active Promoter;H1,3 Poised Promoter;NI	HSMM,6_Weak_Enhancer;Huvec,4_Strong_Enhancer;HMEC,4_Strong_Enhancer	PANC.I,4 TssWk;BR.MYO,4 TssWk;IPS.15,1 TssP;PFK.2,2 TssF;MUS.SC,2 TssF;ADI.MSC,3 TssA;IPS.18,1 TssP;IPS.20,1
rs59717073	GM12878,1 Active Promoter;NHLF,1 Active Promoter;H1,3 Poised Promoter;NI	Huvec,4_Strong_Enhancer;HMEC,4_Strong_Enhancer	PANC.I,4 TssWk;IPS.15,1 TssP;PFK.2,2 TssF;MUS.SC,2 TssF;ADI.MSC,3 TssA;IPS.18,1 TssP;IPS.20,1 TssP;HUES48,1 Tss
rs11717133	GM12878,1 Active Promoter;NHLF,1 Active Promoter;H1,3 Poised Promoter;NI	Huvec,4_Strong_Enhancer;HMEC,4_Strong_Enhancer	PANC.I,4 TssWk;PFK.2,2 TssF;MUS.SC,2 TssF;ADI.MSC,3 TssA;IPS.18,1 TssP;IPS.20,1 TssP;HUES48,1 TssP;LIV.A,3 Tss
rs63423161	.	HSMM,5 Strong Enhancer;Huvec,5 Strong Enhancer;GM12878,5 Strong Enhancer;HMEC,7 Weak Enhan	.
rs9842198	.	GM12878,7_Weak_Enhancer	.
rs9858861	.	NHEK,5_Strong_Enhancer;HMEC,7_Weak_Enhancer;K562,6_Weak_Enhancer;NHLF,7_Weak_Enhancer	.
rs13072896	.	HMEC,7_Weak_Enhancer;K562,6_Weak_Enhancer;NHLF,7_Weak_Enhancer;NHEK,7_Weak_Enhancer	.

SNP	Enhancer (Roadmap)	Gene	dbSNP functional annotation
**rs72906468**	.	*RCC2*	.
rs34548121	CD15.P,11_EnhWk1	*PADI6*	Intronic
rs34167614	HUES6,12_EnhWk2;H1.DNP,11_EnhWk1;IPS.DF19,11_EnhWk1;PFK.2,14_Enh	*PADI6*	3'-UTR
rs717612	PFK.2,11 EnhWk1;PFK.3,12 EnhWk2;BR.MYO,11 EnhWk1;BR.H35,13 EnhA;PFM.2,14 E	*PADI6*	.
rs12093070	.	*RCC2*	Intronic
rs6586541	PFM.2,9 TxEnhG1;IPS.DF19,9 TxEnhG1;HUES6,9 TxEnhG1;R.MUC31,9 TxEnhG1;GAS,9	*RCC2*	Intronic
rs7549366	H1.BMP4DM,14 Enh;CD34.MBP1480,11 EnhWk1;CD34.P,14 Enh;HUES6,14 Enh;CD34.M	*RCC2*	Intronic
rs2883272	HD.CD184EC,12_EnhWk2;HUES6,11_EnhWk1	*RCC2*	.
rs6692736	ADI.MSC,11_EnhWk1;SK.MUS62,14_Enh;ADI.NUC,12_EnhWk2	*RCC2*	.
rs35016926	SK.MUS62,14_Enh;ADI.NUC,12_EnhWk2;MSC.ADIPC,12_EnhWk2	*RCC2*	.
**rs3790585**	CD34.MBP1480,11 EnhWk1;ESO,14 Enh;PFK.3,11 EnhWk1;CD34.MBP1508,11 EnhWk1;(	*AKR1A1*	Intronic
rs514595	SK.MUS,14_Enh	*AKR1A1*	.
rs518216	.	*AKR1A1*	.
rs659437	.	*AKR1A1*	.
rs499600	HUES48,11 EnhWk1;HUES6,11 EnhWk1;IPS.15,11 EnhWk1;ES.I3,13 EnhA;H1,13 EnhA;I	*AKR1A1*	.
rs35506410	HUES48,11 EnhWk1;HUES6,11 EnhWk1;IPS.15,11 EnhWk1;H1,13 EnhA;H1.BMP4DM,11	*AKR1A1*	.
**rs2758598**	SK.MUS,9 TxEnhG1;H1.BMP4DM,14 Enh;BN.AG,14 Enh;BN.CC,14 Enh;BN.HM150,14 E	*PMF1*	Intronic
rs6427304	.	*SLC25A44*	.
rs11587860	.	*SLC25A44*	.
rs2758615	.	*SLC25A44*	.
rs2842864	.	*SLC25A44*	.
rs2842865	.	*SLC25A44*	.
rs2758616	.	*SLC25A44*	.
rs2842869	.	*SLC25A44*	.
rs2758618	.	*SLC25A44*	.
rs2758619	CD19.P,14 Enh;CD34.C,11 EnhWk1;CD34.MBP1562,14 Enh;CD34.MBP1508,14 Enh;CD15	*SLC25A44*	.
rs2540183	SPL,11 EnhWk1;H1.DMSC,11 EnhWk1;CCCRA.NP,11 EnhWk1;CCCRO.MP,11 EnhWk1;	*SLC25A44*	Intronic
rs3001790	H1.DMSC,11 EnhWk1;CCCRA.NP,11 EnhWk1;CCCRO.MP,11 EnhWk1;LV,14 Enh;PANC	*SLC25A44*	Intronic
rs2984615	H1.DMSC,11 EnhWk1;CCCRA.NP,11 EnhWk1;CCCRO.MP,11 EnhWk1;LV,14 Enh;PANC	*SLC25A44*	Intronic
rs2842882	H1.DMSC,11 EnhWk1;CCCRA.NP,11 EnhWk1;CCCRO.MP,11 EnhWk1;LV,14 Enh;PANC	*SLC25A44*	Intronic
rs2842857	HD.CD184EC,9 TxEnhG1;CD15.P,9 TxEnhG1;CD4.NP,11 EnhWk1;CC.TPC,11 EnhWk1;S	*SLC25A44*	Intronic
rs2072499	CD15.P,9 TxEnhG1;CD4.NP,11 EnhWk1;BN.CC,9 TxEnhG1;PFM.2,9 TxEnhG1;BN.MFL,9	*SLC25A44*	Intronic
rs2253677	CD4.NP,11 EnhWk1;LIV.A,9 TxEnhG1;SK.MUS,9 TxEnhG1;CC.TPC,9 TxEnhG1;ST.SMU	*SLC25A44*	Intronic
rs2253809	LIV.A,9 TxEnhG1;CC.TPC,9 TxEnhG1;BN.GM2,9 TxEnhG1;PFF.2,9 TxEnhG1;CD34.P,9	*SLC25A44*	Intronic
rs2266514	COL.SMUS,11 EnhWk1;ADI.NUC,11 EnhWk1;CD15.P,13 EnhA;ADI.MSC,14 Enh;MSC.A	*SLC25A44*	Intronic
rs2736605	ADI.NUC,13 EnhA;MSC.ADIPC,13 EnhA;BN.HM150,9 TxEnhG1;ADI.MSC,13 EnhA;BN.I	*SLC25A44*	Intronic
rs200096331	MSC.ADIPC,13_EnhA;CD15.P,9_TxEnhG1;ADI.NUC,14_Enh	*SLC25A44*	Intronic
rs2241108	IPS.DF19,14_Enh;PFF.2,9_TxEnhG1	*SLC25A44*	3'-UTR
rs2853641	IPS.DF19,14 Enh;PFF.2,10 TxEnhG2;PFM.1,9 TxEnhG1;BN.FE2,9 TxEnhG1;BN.AC,9 Tx	*SLC25A44*	3'-UTR
rs2241107	SPL,14_Enh;H1.DMSC,14_Enh;ESO,14_Enh;H1.BMP4DT,14_Enh;GAS,14_Enh	*PMF1*	.
rs1060604	H1.BMP4DM,14 Enh;BN.SN,13 EnhA;CD4.NP,11 EnhWk1;CD8.NP,11 EnhWk1;H1.DMSC	*PMF1*	Intronic
rs1137703	CD4.NP,11 EnhWk1;CD8.NP,11 EnhWk1;BR.H35,12 EnhWk2;ST.SMUS28,10 TxEnhG2;C	*PMF1*	Intronic
rs2244144	CD4.NP,11 EnhWk1;CD8.NP,11 EnhWk1;BR.H35,12 EnhWk2;ST.SMUS28,10 TxEnhG2;C	*PMF1*	Intronic
rs2540175	CD8.NP,11 EnhWk1;PANC,11 EnhWk1;GAS,11 EnhWk1;BN.GM2,14 Enh;CD4.MP,11 Er	*PMF1*	Intronic
rs2853643	CD8.NP,11 EnhWk1;PANC,11 EnhWk1;GAS,11 EnhWk1;CD4.MP,11 EnhWk1;CD8.MP,1	*PMF1*	Intronic
rs7534434	CD3.P,10 TxEnhG2;H1.DNP,10 TxEnhG2;IPS.DF19,10 TxEnhG2;CD34.MBP1508,10 TxEr	*PMF1*	Intronic
rs2475757	CD3.P,10 TxEnhG2;H1.DNP,10 TxEnhG2;IPS.DF19,10 TxEnhG2;CD34.MBP1508,10 TxEr	*PMF1*	Intronic
rs2540173	SPL,14 Enh;CCIP.LSMPTP,13 EnhA;CC.TPC,11 EnhWk1;CD8.MP,11 EnhWk1;ESO,14 E	*PMF1*	Intronic
rs2853646	SPL,14 Enh;CD34.MBP1480,11 EnhWk1;CD34.MBP1562,13 EnhA;CD34.MBP1549,11 Enh	*PMF1*	Intronic
rs7529564	SPL,14 Enh;CD34.MBP1480,11 EnhWk1;CD34.MBP1562,13 EnhA;CD34.MBP1549,11 Enh	*PMF1*	Intronic
rs6427307	CD34.MBP1562,14_Enh;CD34.MBP1536,9_TxEnhG1	*PMF1*	Intronic
rs2247476	CD34.MBP1562,14_Enh;CD34.MBP1536,9_TxEnhG1	*PMF1*	Intronic
rs887953	CD34.MBP1536,9 TxEnhG1;CD34.MBP1562,9 TxEnhG1;CC.TPC,9 TxEnhG1;BN.MFL,9 T	*PMF1*	Intronic
rs909269	CD34.MBP1562,9 TxEnhG1;CCIP.LSMPTP,9 TxEnhG1;SK.MUS,9 TxEnhG1;CCIP.LSTP,1	*PMF1*	Intronic
rs2247937	SK.MUS,9 TxEnhG1;H1.BMP4DM,14 Enh;BN.AG,14 Enh;BN.CC,14 Enh;BN.HM150,14 E	*PMF1*	Intronic
rs2758600	H1.BMP4DM,14 Enh;BN.AG,14 Enh;BN.CC,14 Enh;BN.SN,14 Enh;BN.MFL,14 Enh;BN.I	*PMF1*	Intronic
rs2248074	BN.MFL,14 Enh;LIV.A,11 EnhWk1;CCC.TREGP,14 Enh;CD34.MBP1480,11 EnhWk1;CD3	*PMF1*	Intronic
rs2248080	BN.MFL,14 Enh;LIV.A,11 EnhWk1;CCC.TREGP,14 Enh;CD34.MBP1480,11 EnhWk1;CD3	*PMF1*	Intronic
rs2736613	BN.MFL,14 Enh;LIV.A,11 EnhWk1;CCC.TREGP,14 Enh;CD34.MBP1480,11 EnhWk1;BN.	*PMF1*	Intronic
rs2984613	DUO.MUC61,14_Enh;BN.CC,14_Enh	*PMF1*	Intronic
rs3001789	DUO.MUC61,14_Enh;BN.CC,14_Enh	*PMF1*	Intronic
rs2758603	DUO.MUC61,9_TxEnhG1	*PMF1*	Intronic
rs143621173	PFF.2,9 TxEnhG1;ADI.MSC,9 TxEnhG1;SK.MUS,9 TxEnhG1;BN.CC,14 Enh;R.SMUS,11	*PMF1*	Intronic
rs2758605	ADI.MSC,9 TxEnhG1;SK.MUS,9 TxEnhG1;BN.CC,14 Enh;R.SMUS,11 EnhWk1;COL.SMU	*PMF1*	Intronic
rs2842870	BN.CC,14_Enh;COL.SMUS,14_Enh	*PMF1*	Intronic
rs1052053	CD34.MBP1562,11 EnhWk1;PFF.2,9 TxEnhG1;ES.I3,11 EnhWk1;PFM.2,9 TxEnhG1;HUES	*PMF1*	Nonsynonymous; intronic
rs2736609	CD34.MBP1562,11 EnhWk1;HD.CD56MESC,11 EnhWk1;PFK.3,11 EnhWk1;MUS.SC,11 I	*PMF1*	Intronic
rs2758607	CD34.MBP1562,11 EnhWk1;HD.CD56MESC,11 EnhWk1;PFK.3,11 EnhWk1;MUS.SC,11 I	*PMF1*	Intronic
rs2251636	HD.CD56MESC,11 EnhWk1;PFK.3,11 EnhWk1;MUS.SC,11 EnhWk1;BM.MSC,11 EnhWk	*PMF1*	Intronic
rs2758608	HD.CD56MESC,11 EnhWk1;PFK.3,11 EnhWk1;MUS.SC,11 EnhWk1;BM.MSC,11 EnhWk	*PMF1*	Intronic
rs2758609	HD.CD56MESC,11 EnhWk1;PFK.3,11 EnhWk1;MUS.SC,11 EnhWk1;BM.MSC,11 EnhWk	*PMF1*	Intronic
rs2251847	HD.CD56MESC,9_TxEnhG1;IMR90,9_TxEnhG1;MUS.SC,13_EnhA;PFF.2,9_TxEnhG1	*PMF1*	Intronic
rs2842873	.	*PMF1*	Intronic
rs2736611	.	*PMF1*	Intronic
**rs6756513**	CD34.MBP1480,11 EnhWk1;CD34.P,13 EnhA;CD34.MBP1536,13 EnhA;CD34.MBP1549,1	*MXD1*	.
**rs73006998**	CD4.MP,11 EnhWk1;GAS,11 EnhWk1;LIV.A,13 EnhA;ESO,14 Enh;CCIP.LSTP,11 EnhW	*SIAH2*	Intronic
rs6789306	LIV.A,13 EnhA;CD4.MP,11 EnhWk1;CCIP.LSTP,14 Enh;LV,14 Enh;GAS,11 EnhWk1;PA	*SIAH2*	Intronic
rs7615125	CD4.MP,11 EnhWk1;GAS,11 EnhWk1;PFF.1,11 EnhWk1;BM.MSC,13 EnhA;HD.CD56ME	*SIAH2*	Intronic
rs7615292	CD4.MP,11 EnhWk1;GAS,11 EnhWk1;PFF.1,11 EnhWk1;BM.MSC,13 EnhA;HD.CD56ME	*SIAH2*	Intronic
rs73006999	GAS,11 EnhWk1;LIV.A,13 EnhA;ESO,14 Enh;CCIP.LSTP,11 EnhWk1;R.MUC29,11 EnhV	*SIAH2*	Intronic
rs73008903	GAS,11 EnhWk1;LIV.A,13 EnhA;ESO,14 Enh;CCIP.LSTP,11 EnhWk1;R.MUC29,11 EnhV	*SIAH2*	Intronic
rs73008905	GAS,11 EnhWk1;LIV.A,13 EnhA;CCIP.LSTP,11 EnhWk1;R.MUC29,11 EnhWk1;ST.MUC	*SIAH2*	Intronic
rs73008907	GAS,11 EnhWk1;LIV.A,13 EnhA;CCIP.LSTP,11 EnhWk1;R.MUC29,11 EnhWk1;ST.MUC	*SIAH2*	Intronic
rs55899470	GAS,11 EnhWk1;LIV.A,13 EnhA;CCIP.LSTP,11 EnhWk1;R.MUC29,11 EnhWk1;ST.MUC	*SIAH2*	Intronic
rs57824095	GAS,11 EnhWk1;LIV.A,13 EnhA;CCIP.LSTP,11 EnhWk1;R.MUC29,11 EnhWk1;ST.MUC	*SIAH2*	Intronic
rs1879420	GAS,11 EnhWk1;LIV.A,13 EnhA;CCIP.LSTP,11 EnhWk1;R.MUC29,11 EnhWk1;ST.MUC	*SIAH2*	Intronic
rs1991781	LIV.A,13 EnhA;R.MUC29,11 EnhWk1;ST.MUC,11 EnhWk1;PFK.3,9 TxEnhG1;CD4.MP,1	*SIAH2*	Intronic
rs2116684	CCIP.LSMPTP,9 TxEnhG1;CC.TPC,11 EnhWk1;ST.MUC,11 EnhWk1;CCIP.LSTP,11 Enh	*SIAH2*	Intronic
rs57359526	CC.TPC,11 EnhWk1;CCIP.LSTP,11 EnhWk1;PFK.2,9 TxEnhG1;ESO,14 Enh;GAS,11 Enh	*SIAH2*	Intronic
rs61628582	CC.TPC,11 EnhWk1;CCIP.LSTP,11 EnhWk1;PFK.2,9 TxEnhG1;ESO,14 Enh;GAS,11 Enh	*SIAH2*	Intronic
rs2166061	CC.TPC,11 EnhWk1;CCIP.LSTP,11 EnhWk1;PFK.2,9 TxEnhG1;GAS,11 EnhWk1;LIV.A,1	*SIAH2*	Intronic
rs2120596	CC.TPC,11 EnhWk1;CCIP.LSTP,11 EnhWk1;PFK.2,9 TxEnhG1;GAS,11 EnhWk1;LIV.A,1	*SIAH2*	Intronic
rs60092981	CCIP.LSTP,11 EnhWk1;GAS,11 EnhWk1;R.MUC31,14 Enh;PANC,11 EnhWk1;CD4.MP,1	*SIAH2*	Intronic
rs57965761	R.MUC31,14 Enh;R.MUC29,11 EnhWk1;ADI.NUC,14 Enh;LIV.A,13 EnhA;CCIP.LSMPTP	*SIAH2*	Intronic
rs60538652	R.MUC31,14 Enh;BM.MSC,14 Enh;CD15.P,11 EnhWk1;IMR90,14 Enh;HRT.FE,14 Enh;LI	*SIAH2*	Intronic
rs60523415	HRT.FE,11 EnhWk1;CCC.TREGP,11 EnhWk1;ESO,14 Enh;CD8.MP,11 EnhWk1;PFK.2,14	*SIAH2*	Intronic
rs75942495	CCC.TMP,14 Enh;H9,11 EnhWk1;IMR90,9 TxEnhG1;BN.FE2,11 EnhWk1;H1.BMP4DM,11	*SIAH2*	Intronic
rs1915365	SK.MUS63,13 EnhA;SPL,14 Enh;LV,14 Enh;ESO,14 Enh;H1,12 EnhWk2;HD.CD56MESC,	*SIAH2*	Intronic
rs1838337	.	*SIAH2*	Intronic
rs1444197	.	*SIAH2*	Intronic
rs74950732	CCC.TMP,14 Enh;LV,14 Enh;CCCRO.MP,13 EnhA;ST.MUC,14 Enh;HRT.FE,13 EnhA;PF	*SIAH2*	.
rs76801042	CCC.TMP,14 Enh;LV,14 Enh;CCCRO.MP,13 EnhA;ST.MUC,14 Enh;HRT.FE,13 EnhA;PF	*SIAH2*	.
rs79914414	ST.MUC,14 Enh;ES.I3,11 EnhWk1;CD4.MP,12 EnhWk2;MSC.ADIPC,11 EnhWk1;CD34.C	*SIAH2*	.
rs74549747	HUES64,12 EnhWk2;IPS.15,12 EnhWk2;IMR90,11 EnhWk1;CD34.C,12 EnhWk2;CHON.B	*SIAH2*	.
rs16862842	HUES64,12 EnhWk2;IPS.15,12 EnhWk2;CCIP.LSMPTP,13 EnhA;CCIP.LSTP,13 EnhA;R.I	*SIAH2*	.
rs79709238	LIV.A,11 EnhWk1;HRT.FE,11 EnhWk1;PFF.1,11 EnhWk1;H1.DMSC,11 EnhWk1;MUS.S(	*SIAH2*	.
rs41372346	PFF.1,11 EnhWk1;H1.DMSC,11 EnhWk1;MUS.SC,11 EnhWk1;MSC.ADIPC,11 EnhWk1;I	*SIAH2*	.
rs34901265	MSC.ADIPC,11 EnhWk1;PFF.2,11 EnhWk1;ADI.MSC,11 EnhWk1;ADI.NUC,12 EnhWk2;	*SIAH2*	.
rs77431731	LIV.A,14_Enh	*SIAH2*	.
rs59741644	.	*SIAH2*	.
rs73010967	.	*SIAH2*	.
rs6803830	HRT.FE,12_EnhWk2	*SIAH2*	.
rs78552868	.	*SIAH2*	.
rs77437769	NCC.COR2,11_EnhWk1	*SIAH2*	.
rs78943763	NCC.COR2,11_EnhWk1	*SIAH2*	.
rs149408779	.	*SIAH2*	.
rs201959055	.	*SIAH2*	.
rs113840938	.	*SIAH2*	.
rs76936100	HUES6,14_Enh;H1.DNP,14_Enh	*SIAH2*	.
rs58403524	HUES6,14_Enh	*SIAH2*	.
**rs11281251**	PFK.3,9_TxEnhG1;BR.H35,11_EnhWk1;BR.MYO,11_EnhWk1	*LOC730091*	Intronic
rs199589043	PFK.3,9_TxEnhG1	*LOC730091*	Intronic
rs1835372	PFK.3,9_TxEnhG1;BR.H35,11_EnhWk1	*LOC730091*	Intronic
rs1430411	BM.MSC,14 Enh;PFM.3,11 EnhWk1;PFF.1,14 Enh;MUS.SC,14 Enh;HUES48,12 EnhWk2;	*LOC730091*	Intronic
rs1430410	PFF.1,14 Enh;HUES48,12 EnhWk2;ADI.MSC,13 EnhA;PFK.3,9 TxEnhG1;CHON.BMMSC	*LOC730091*	Intronic
rs9812169	ADI.MSC,14 Enh;BR.MYO,11 EnhWk1;PFK.2,13 EnhA;GAS,11 EnhWk1;H1.DMSC,11 E	*PA2G4P4*	Intronic
rs7641929	BR.MYO,11 EnhWk1;H1.BMP4DT,14 Enh;DUO.MUC61,11 EnhWk1;PFF.1,13 EnhA;R.MU	*PA2G4P4*	Intronic
rs12492804	BR.MYO,11 EnhWk1;BR.H35,11 EnhWk1;PFK.2,13 EnhA;LIV.A,14 Enh;ESO,14 Enh;PFI	*LOC730091*	Intronic
rs12497539	BN.FE0,15 EnhP;HD.CD56MESC,14 Enh;CCIP.LSTP,12 EnhWk2;CD3.P,14 Enh;CD4.NP,	*LOC730091*	Intronic
rs59717073	CD34.P,12 EnhWk2;CCCRO.MP,14 Enh;CCC.TMP,14 Enh;CD8.MP,14 Enh;BN.FE0,12 E	*LOC730091*	Intronic
rs11717133	CCCRO.MP,14 Enh;CCC.TMP,14 Enh;CD8.MP,14 Enh;HRT.FE,14 Enh;CD34.MBP1480,1	*LOC730091*	Intronic
rs63423161	PFF.2,14 Enh;MUS.SC,13 EnhA;CHON.BMMSC,14 Enh;PFK.3,14 Enh;PFF.1,11 EnhWk1	*LOC730091*	.
rs9842198	PFF.2,14_Enh;MUS.SC,14_Enh	*LOC730091*	.
rs9858861	PFF.2,14 Enh;MUS.SC,14 Enh;R.MUC31,11 EnhWk1;ESO,14 Enh;ST.MUC,11 EnhWk1;B	*LEKR1*	.
rs13072896	PFF.2,14 Enh;MUS.SC,14 Enh;ESO,14 Enh;BM.MSC,14 Enh;CD15.P,14 Enh;CHON.BMN	*LEKR1*	.

rs6776157	3	156543482	0.83	0.99	T	A	0.36	0.72	0.84	0.62	0	0	H1,3_Poised_Promoter	HMEC,5_Strong_Enhancer;K562,4_Strong_Enhancer;HSMM,5_Strong_Enhancer;HepG2,6_Weak_Enhancer;	PFK.3,2_TssF;CD34.MBP1562,2_TssF;CD3.P,2_TssF;BN.FE0,4_TssWk;HRT.FE,2_TssF;KID.FE,6_TssD2;LNG.FE,5_TssD1;IMR	ST.MUC,14_Enh;LIV.A,14_Enh;H1.BMP4DT,11_EnhWk1;H1.DMSC,11_EnhWk1;CD34.MB	*LEKR1*	.
**rs11944638**	**4**	**48227719**	-	-	T	C	0.14	0.12	0.24	0.05	0	0	.	H1,7_Weak_Enhancer	.	CD15.P,13_EnhA;IPS.DF19,14_Enh;HUES48,12_EnhWk2;H1.DMSC,11_EnhWk1;H9,12_Enh	*TEC*	Intronic
rs6829390	4	48198986	0.88	0.97	G	A	0.45	0.16	0.26	0.06	1	0	GM12878,1_Active_Promoter	K562,5_Strong_Enhancer	.	CD34.MBP1536,11_EnhWk1;PFF.1,11_EnhWk1;CD34.C,9_TxEnhG1;CD34.P,14_Enh;PFF.2	*TEC*	Intronic
rs73814690	4	48203463	0.86	0.95	A	G	0.12	0.12	0.24	0.06	0	0	.	GM12878,5_Strong_Enhancer;K562,5_Strong_Enhancer	.	CD34.C,9_TxEnhG1;CD34.MBP1480,11_EnhWk1;CD34.MBP1562,13_EnhA;CD15.P,11_Enh	*TEC*	Intronic
rs73138446	4	48204780	0.9	0.95	T	C	0.12	0.12	0.24	0.05	0	0	.	K562,7_Weak_Enhancer;GM12878,4_Strong_Enhancer	.	CD34.MBP1480,11_EnhWk1;CD34.MBP1562,13_EnhA;CD34.MBP1536,14_Enh;CD34.MBP1	*TEC*	Intronic
rs11942525	4	48205942	0.9	0.95	C	T	0.12	0.12	0.24	0.05	0	0	.	GM12878,4_Strong_Enhancer;K562,4_Strong_Enhancer	.	CD34.MBP1480,11_EnhWk1;CD34.MBP1562,13_EnhA;CD34.P,13_EnhA;CD34.C,13_EnhA;	*TEC*	Intronic
rs73138447	4	48206396	0.9	0.95	C	T	0.12	0.12	0.24	0.05	0	0	.	GM12878,4_Strong_Enhancer;K562,4_Strong_Enhancer	.	CD34.MBP1562,13_EnhA;CD34.C,13_EnhA;CD19.P,11_EnhWk1;CD4.MP,14_Enh;HRT.FE,	*TEC*	Intronic
rs73138450	4	48206765	0.9	0.95	C	T	0.12	0.12	0.24	0.05	0	0	.	GM12878,4_Strong_Enhancer;K562,4_Strong_Enhancer	.	CD34.MBP1562,13_EnhA;CD34.C,13_EnhA;HRT.FE,11_EnhWk1;CD34.MBP1536,13_EnhA	*TEC*	Intronic
rs2136504	4	48211923	0.9	0.95	A	G	0.12	0.12	0.24	0.05	0	0	.	GM12878,5_Strong_Enhancer	.	CD34.MBP1480,11_EnhWk1;CD34.MBP1508,11_EnhWk1;CD34.MBP1536,13_EnhA;CD34.M	*TEC*	Intronic
rs1159075	4	48215854	0.9	0.95	C	T	0.12	0.12	0.24	0.05	0	0	.	GM12878,5_Strong_Enhancer	.	CD34.MBP1480,11_EnhWk1;CD34.MBP1562,13_EnhA;CD34.MBP1549,14_Enh;CD15.P,14	*TEC*	Intronic
rs145795515	4	48217094	0.9	0.95	C	T	0.12	0.12	0.24	0.05	0	0	.	.	.	CD34.C,13_EnhA;CD34.MBP1562,14_Enh	*TEC*	Intronic
rs73138478	4	48220377	0.95	0.97	T	C	0.12	0.12	0.24	0.05	0	0	.	.	.	.	*TEC*	Intronic
rs11722540	4	48221182	0.95	0.97	C	T	0.12	0.12	0.24	0.05	0	0	.	K562,7_Weak_Enhancer	.	.	*TEC*	Intronic
rs36067666	4	48224710	0.95	0.97	GC	G	0.12	0.12	0.24	0.05	0	0	.	K562,7_Weak_Enhancer	.	BR.H35,11_EnhWk1;CD15.P,14_Enh	*TEC*	Intronic
rs34585972	4	48224717	0.95	0.97	C	T	0.12	0.12	0.24	0.05	0	0	.	K562,7_Weak_Enhancer	.	BR.H35,11_EnhWk1;CD15.P,14_Enh	*TEC*	Intronic
**rs11947923**	**4**	**53911337**	-	-	C	T	0.06	0.36	0.29	0.4	0	0	.	.	.	BN.FE1,12_EnhWk2	*SCFD2*	Intronic
rs5858201	4	53884062	0.82	0.98	CG	C	0.03	0.36	0.28	0.36	0	0	.	.	.	KID.FE,11_EnhWk1	*SCFD2*	Intronic
rs12506630	4	53912593	1	1	G	C	0.04	0.36	0.29	0.4	0	0	.	.	.	.	*SCFD2*	Intronic
rs17082328	4	53912794	1	1	T	C	0.04	0.37	0.29	0.4	0	0	.	.	.	.	*SCFD2*	Intronic
**rs6555134**	**5**	**2776483**	-	-	T	C	0.52	0.49	0.74	0.42	0	0	.	.	.	.	*C5orf38*	.
rs2935617	5	2764948	0.8	-0.99	G	C	0.62	0.62	0.68	0.63	0	0	.	.	.	.	*C5orf38*	.
rs6555129	5	2765442	0.8	-1	G	A	0.88	0.65	0.68	0.63	0	0	.	.	.	.	*C5orf38*	.
rs6883371	5	2767357	0.8	-1	C	T	0.76	0.64	0.68	0.63	0	0	.	H1,7_Weak_Enhancer	.	HUES6,12_EnhWk2;IPS.18,12_EnhWk2;HUES64,12_EnhWk2;HUES48,12_EnhWk2	*C5orf38*	.
rs2935622	5	2767527	0.8	-1	A	C	0.88	0.65	0.68	0.63	0	0	.	H1,7_Weak_Enhancer	.	HUES6,12_EnhWk2;IPS.18,12_EnhWk2;HUES64,12_EnhWk2;HUES48,12_EnhWk2	*C5orf38*	.
rs2962597	5	2767835	0.88	-1	T	C	0.68	0.61	0.68	0.61	0	0	.	.	.	HUES48,12_EnhWk2	*C5orf38*	.
rs2962588	5	2768502	0.8	-1	C	G	0.88	0.65	0.68	0.63	0	0	.	.	.	.	*C5orf38*	.
rs2935600	5	2769264	0.8	-1	T	C	0.88	0.65	0.68	0.63	0	0	.	.	.	HUES48,12_EnhWk2	*C5orf38*	.
rs76899139	5	2775515	0.81	0.96	C	T	0.14	0.36	0.32	0.39	0	0	.	.	.	.	*C5orf38*	.
rs4866492	5	2776562	0.85	1	C	T	0.13	0.35	0.33	0.38	0	0	.	.	.	.	*C5orf38*	.
rs4866493	5	2776719	1	1	G	C	0.52	0.49	0.74	0.42	0	0	.	.	.	.	*C5orf38*	.
rs4866496	5	2777029	1	1	G	A	0.52	0.49	0.74	0.42	0	0	.	.	.	.	*C5orf38*	.
rs7734695	5	2777159	1	1	C	T	0.52	0.49	0.74	0.42	0	0	.	.	.	.	*C5orf38*	.
rs1497465	5	2777249	0.99	1	C	T	0.5	0.49	0.74	0.42	0	0	.	.	.	H1.BMP4DT,11_EnhWk1	*C5orf38*	.
rs7709328	5	2777311	1	1	G	A	0.51	0.49	0.74	0.42	0	0	.	.	.	H1.BMP4DT,11_EnhWk1	*C5orf38*	.
rs11960374	5	2777869	0.84	0.99	T	C	0.13	0.35	0.33	0.38	0	0	.	.	.	.	*C5orf38*	.
rs2068829	5	2778782	0.89	0.99	C	T	0.4	0.44	0.86	0.39	0	0	.	.	.	.	*C5orf38*	.
**rs7765429**	**6**	**21904169**	-	-	T	C	0.68	0.38	0.1	0.51	0	0	.	.	.	HD.CD184EC,11_EnhWk1	*FLJ22536*	Intronic
rs9466206	6	21901658	0.97	1	A	C	0.68	0.38	0.1	0.52	0	0	.	.	.	H1.BMP4DM,14_Enh;CHON.BMMSC,11_EnhWk1	*FLJ22536*	Intronic
rs9460684	6	21902053	0.87	1	C	G	0.68	0.4	0.09	0.54	0	0	.	.	.	H1.BMP4DM,14_Enh	*FLJ22536*	Intronic
rs7760611	6	21903533	0.96	0.99	T	C	0.68	0.38	0.1	0.51	0	0	.	.	.	HUES64,11_EnhWk1;IPS.20,11_EnhWk1;HD.CD184EC,13_EnhA;LNG.FE,11_EnhWk1	*FLJ22536*	Intronic
rs9460685	6	21904671	0.94	0.97	A	G	0.48	0.35	0.1	0.51	0	0	.	.	.	HD.CD184EC,11_EnhWk1	*FLJ22536*	Intronic
rs9460686	6	21904934	0.94	0.97	A	C	0.48	0.35	0.1	0.51	0	0	.	.	.	HD.CD184EC,11_EnhWk1	*FLJ22536*	Intronic
rs9368364	6	21905643	0.93	0.97	T	A	0.39	0.33	0.1	0.51	0	0	.	.	.	HD.CD184EC,9_TxEnhG1	*FLJ22536*	Intronic
**rs7768862**	**6**	**85088846**	-	-	T	A	0.34	0.36	0.26	0.51	0	0	.	GM12878,6_Weak_Enhancer	.	.	*KIAA1009*	.
**rs6940159**	**6**	**1.7E+08**	-	-	T	C	0.59	0.44	0.18	0.6	0	0	.	.	.	CD15.P,12_EnhWk2;SPL,14_Enh	*C6orf208*	.
**rs144145984**	**8**	**23644003**	-	-	CT	C	0.53	0.46	0.53	0.42	0	0	.	H1,5_Strong_Enhancer	IPS.20,4_TssWk	IPS.DF19,14_Enh;IPS.DF6,14_Enh;HUES64,13_EnhA;HUES48,13_EnhA;H1.BMP4DM,12_E	*STC1*	.
**rs2849506**	**8**	**1.01E+08**	-	-	G	C	0.61	0.34	0.46	0.36	0	0	.	.	IPS.15,1_TssP	IPS.DF19,11_EnhWk1	*RNF19A*	.
rs2453624	8	101230523	0.81	-0.92	A	G	0.85	0.72	0.58	0.63	0	0	.	GM12878,5_Strong_Enhancer	.	CD34.MBP1480,11_EnhWk1;CD8.MP,11_EnhWk1;CCIP.LSMPTP,13_EnhA;CD34.C,11_Enh	*SPAG1*	Intronic
rs2083499	8	101230534	0.81	-0.92	T	A	0.85	0.72	0.58	0.63	0	0	.	GM12878,5_Strong_Enhancer	.	CD34.MBP1480,11_EnhWk1;CD8.MP,11_EnhWk1;CCIP.LSMPTP,13_EnhA;CD34.C,11_Enh	*SPAG1*	Intronic
rs2919471	8	101232701	0.82	-0.92	T	C	0.85	0.72	0.58	0.63	0	0	.	.	.	CD34.MBP1508,11_EnhWk1;ST.MUC,11_EnhWk1;R.MUC31,11_EnhWk1;LNG.FE,11_EnhW	*SPAG1*	Intronic
rs3020182	8	101233008	0.82	-0.92	T	C	0.75	0.71	0.58	0.63	0	0	.	.	.	R.MUC31,11_EnhWk1	*SPAG1*	Intronic
rs2919469	8	101233896	0.82	-0.92	T	C	0.84	0.72	0.58	0.63	0	0	.	.	.	.	*SPAG1*	Intronic
rs2047249	8	101234126	0.82	-0.92	A	G	0.87	0.72	0.58	0.63	0	0	.	.	.	IPS.DF6,12_EnhWk2;ES.WA7,14_Enh	*SPAG1*	Intronic
rs2860518	8	101238120	0.82	-0.92	G	T	0.47	0.68	0.57	0.63	0	0	.	.	.	CD15.P,11_EnhWk1;CD19.P,14_Enh	*SPAG1*	Intronic
rs1660338	8	101242757	0.82	-0.92	C	G	0.47	0.67	0.51	0.63	0	0	.	.	.	R.MUC29,11_EnhWk1;R.MUC31,13_EnhA	*SPAG1*	Intronic
rs1613453	8	101244215	0.82	-0.92	G	T	0.82	0.72	0.58	0.63	0	0	.	.	.	ST.MUC,11_EnhWk1;R.MUC29,14_Enh;R.MUC31,11_EnhWk1	*SPAG1*	Intronic
rs1692004	8	101247320	0.82	-0.92	T	C	0.83	0.72	0.58	0.63	0	0	.	.	.	R.MUC31,9_TxEnhG1;ST.MUC,11_EnhWk1;R.MUC29,9_TxEnhG1	*SPAG1*	Intronic
rs1788189	8	101249762	0.83	-0.92	G	T	0.72	0.7	0.51	0.63	0	0	.	.	.	ST.MUC,11_EnhWk1;R.MUC29,11_EnhWk1;R.MUC31,13_EnhA;H1.DMSC,14_Enh;GAS,11	*SPAG1*	Intronic
rs1660353	8	101250907	0.83	-0.92	C	T	0.38	0.65	0.51	0.63	0	0	.	NHLF,6_Weak_Enhancer;K562,5_Strong_Enhancer;HSMM,5_Strong_Enhancer	.	ST.MUC,11_EnhWk1;R.MUC31,13_EnhA;HRT.FE,13_EnhA;IMR90,11_EnhWk1;PFF.1,11_E	*SPAG1*	Intronic
rs34826603	8	101250993	0.82	-0.92	CTTA	C	0.39	0.65	0.51	0.63	0	0	.	NHLF,6_Weak_Enhancer;K562,5_Strong_Enhancer;HSMM,5_Strong_Enhancer	.	ST.MUC,11_EnhWk1;R.MUC31,13_EnhA;HRT.FE,13_EnhA;IMR90,11_EnhWk1;PFF.1,11_E	*SPAG1*	Intronic
rs1692006	8	101252462	0.83	-0.93	C	T	0.74	0.7	0.51	0.63	0	0	.	.	.	H1.DMSC,14_Enh;IPS.18,11_EnhWk1;HRT.FE,11_EnhWk1	*SPAG1*	Intronic
rs6511	8	101252680	0.83	-0.92	T	C	0.72	0.72	0.51	0.63	0	0	.	.	.	IPS.18,11_EnhWk1;HRT.FE,11_EnhWk1;H1.DMSC,9_TxEnhG1;HD.CD184EC,11_EnhWk1	*SPAG1*	Nonsynonymous
rs1630928	8	101257870	0.84	-0.93	A	G	0.74	0.7	0.58	0.63	0	0	.	.	.	.	*SPAG1*	.
rs1618528	8	101259134	0.85	-0.93	T	C	0.83	0.72	0.58	0.63	0	0	.	.	.	CD15.P,11_EnhWk1;CD34.P,11_EnhWk1	*SPAG1*	.
rs1788191	8	101260844	0.86	-0.93	C	T	0.83	0.72	0.58	0.64	0	0	.	.	.	CD15.P,11_EnhWk1	*SPAG1*	.
rs924057	8	101266157	0.86	-0.94	A	G	0.38	0.66	0.51	0.63	0	0	.	.	.	.	*RNF19A*	.
rs1692007	8	101268343	0.86	-0.94	T	C	0.38	0.66	0.51	0.63	0	0	.	.	.	.	*RNF19A*	.
rs1616833	8	101271821	0.86	-0.94	C	T	0.38	0.66	0.51	0.63	0	0	.	Huvec,4_Strong_Enhancer;HSMM,4_Strong_Enhancer;GM12878,4_Strong_Enhancer;NHLF,5_Strong_Enhar	.	CD34.C,13_EnhA;CD34.MBP1536,13_EnhA;CCC.TREGP,9_TxEnhG1;BR.H35,9_TxEnhG1;(	*RNF19A*	Intronic
rs1660351	8	101275034	0.85	0.93	C	T	0.17	0.28	0.42	0.37	0	0	.	HMEC,5_Strong_Enhancer;NHEK,5_Strong_Enhancer;K562,7_Weak_Enhancer	.	CD34.MBP1480,11_EnhWk1;CCIP.LSMPTP,9_TxEnhG1;BR.H35,9_TxEnhG1;CD4.MP,9_Tx	*RNF19A*	Intronic
rs1788194	8	101277916	0.86	0.93	T	C	0.17	0.28	0.42	0.36	0	0	.	.	.	CCIP.LSMPTP,9_TxEnhG1;CD15.P,13_EnhA	*RNF19A*	Intronic
rs1660323	8	101278724	0.86	0.93	T	A	0.16	0.28	0.42	0.36	0	0	.	.	.	CD15.P,13_EnhA;LNG.FE,9_TxEnhG1	*RNF19A*	Intronic
rs1660324	8	101281271	0.86	0.93	C	T	0.16	0.28	0.42	0.36	0	0	.	.	.	.	*RNF19A*	Intronic
rs2861348	8	101285074	0.86	0.94	A	G	0.62	0.34	0.48	0.37	0	0	.	.	.	CD4.MP,11_EnhWk1;H1.DMSC,9_TxEnhG1;LV,9_TxEnhG1;PANC,11_EnhWk1	*RNF19A*	Intronic
rs1020171	8	101288610	0.86	0.93	T	C	0.17	0.28	0.42	0.36	0	0	.	HMEC,5_Strong_Enhancer;HSMM,6_Weak_Enhancer;Huvec,6_Weak_Enhancer;NHLF,6_Weak_Enhancer;N	.	ADI.NUC,14_Enh;DUO.SMUS,11_EnhWk1;H1.DMSC,14_Enh;HRT.FE,11_EnhWk1;BR.H35	*RNF19A*	Intronic
rs35751827	8	101289836	0.86	0.94	A	AT	0.62	0.34	0.48	0.37	0	0	.	.	.	ADI.NUC,14_Enh;HRT.FE,11_EnhWk1;CCIP.LSMPTP,11_EnhWk1;PFK.3,14_Enh;CD4.MP	*RNF19A*	Intronic
rs1788196	8	101290088	0.87	0.94	C	T	0.62	0.34	0.48	0.36	0	0	.	Huvec,6_Weak_Enhancer	.	ADI.NUC,14_Enh;CCIP.LSMPTP,11_EnhWk1;PFK.3,14_Enh;BN.AC,11_EnhWk1;MSC.ADI	*RNF19A*	Intronic
rs1660326	8	101290447	0.86	0.93	G	A	0.17	0.28	0.42	0.36	0	0	.	HSMM,7_Weak_Enhancer	MSC.ADIPC,6_TssD2	PFK.3,14_Enh;BR.H35,11_EnhWk1;CHON.BMMSC,11_EnhWk1;MUS.SC,11_EnhWk1;SK.M	*RNF19A*	Intronic
rs1788197	8	101290928	0.86	0.94	T	C	0.62	0.34	0.48	0.37	0	0	.	.	.	CHON.BMMSC,11_EnhWk1;MUS.SC,11_EnhWk1;LIV.A,11_EnhWk1;BM.MSC,11_EnhWk	*RNF19A*	Intronic
rs6989997	8	101292031	0.84	0.93	A	C	0.16	0.27	0.42	0.37	0	0	.	.	.	LV,11_EnhWk1;PANC,11_EnhWk1;CD4.MP,11_EnhWk1;CCIP.LSMPTP,11_EnhWk1;COL.	*RNF19A*	Intronic
rs1660327	8	101292206	0.85	0.93	A	C	0.16	0.27	0.42	0.37	0	0	.	.	.	CD4.MP,11_EnhWk1;CCIP.LSMPTP,9_TxEnhG1	*RNF19A*	Intronic
rs1788199	8	101292854	0.86	0.93	A	G	0.17	0.27	0.42	0.36	0	0	.	.	.	CD4.MP,11_EnhWk1;CCIP.LSMPTP,9_TxEnhG1;ADI.NUC,14_Enh;MSC.ADIPC,9_TxEnh	*RNF19A*	Intronic
rs959022	8	101295992	0.87	0.94	T	A	0.16	0.27	0.42	0.36	0	0	.	.	.	CD4.MP,11_EnhWk1;COL.SMUS,11_EnhWk1;CCCRO.MP,11_EnhWk1;CCIP.LSMPTP,9_T	*RNF19A*	Intronic
rs2439458	8	101297984	0.87	0.94	T	C	0.62	0.34	0.48	0.37	0	0	.	.	.	CCCRO.MP,11_EnhWk1;CCIP.LSMPTP,9_TxEnhG1;CCC.TREGP,14_Enh;HD.CD184EC,9_	*RNF19A*	Intronic
rs1788202	8	101302556	0.87	0.94	C	T	0.17	0.27	0.43	0.36	0	0	.	NHLF,7_Weak_Enhancer	.	CCIP.LSMPTP,9_TxEnhG1;CD4.MP,11_EnhWk1;PFM.2,11_EnhWk1;CHON.BMMSC,14_E	*RNF19A*	Intronic
rs1660328	8	101303450	0.88	0.94	A	G	0.62	0.34	0.48	0.36	0	0	.	.	.	CD4.MP,11_EnhWk1;PFM.2,11_EnhWk1;PFM.3,11_EnhWk1;LV,11_EnhWk1;MSC.ADIPC,	*RNF19A*	Intronic
rs1788203	8	101303721	0.87	0.94	T	C	0.16	0.27	0.42	0.36	0	0	.	.	.	CD4.MP,11_EnhWk1;MSC.ADIPC,9_TxEnhG1;HRT.FE,11_EnhWk1;R.SMUS,11_EnhWk1;(	*RNF19A*	Intronic
rs1788204	8	101303936	0.87	0.94	C	T	0.16	0.27	0.42	0.36	0	0	.	.	.	CD4.MP,11_EnhWk1;MSC.ADIPC,9_TxEnhG1;HRT.FE,11_EnhWk1;R.SMUS,11_EnhWk1;(	*RNF19A*	Intronic
rs1660329	8	101304048	0.87	0.94	G	T	0.16	0.27	0.42	0.36	0	0	.	.	.	CD4.MP,11_EnhWk1;HRT.FE,11_EnhWk1;R.SMUS,11_EnhWk1;COL.SMUS,11_EnhWk1;B	*RNF19A*	Intronic
rs1660330	8	101305015	0.87	0.94	T	A	0.16	0.27	0.42	0.36	0	0	.	.	.	CD4.MP,11_EnhWk1;COL.SMUS,11_EnhWk1;SK.MUS62,11_EnhWk1;CCIP.LSMPTP,9_Tx	*RNF19A*	Intronic
rs1788205	8	101306546	0.87	0.94	G	C	0.16	0.27	0.42	0.36	0	0	.	GM12878,5_Strong_Enhancer	.	CD4.MP,11_EnhWk1;CCIP.LSMPTP,9_TxEnhG1;CCIP.LSTP,11_EnhWk1;HRT.FE,14_Enh;	*RNF19A*	Intronic
rs1788206	8	101306966	0.87	0.94	A	G	0.16	0.27	0.42	0.36	0	0	.	GM12878,7_Weak_Enhancer	.	CD4.MP,11_EnhWk1;CCIP.LSMPTP,9_TxEnhG1;CCIP.LSTP,11_EnhWk1;HRT.FE,14_Enh;	*RNF19A*	Intronic
rs1660332	8	101309490	0.88	0.94	C	T	0.62	0.34	0.48	0.36	0	0	GM12878,2_Weak_Promoter	.	.	CCIP.LSMPTP,9_TxEnhG1;HRT.FE,11_EnhWk1;LIV.A,14_Enh;CD4.MP,11_EnhWk1;KID.	*RNF19A*	Intronic
rs1660333	8	101309989	0.87	0.94	T	C	0.16	0.27	0.42	0.36	0	0	.	GM12878,6_Weak_Enhancer	.	CCIP.LSMPTP,9_TxEnhG1;HRT.FE,11_EnhWk1;CD4.MP,11_EnhWk1;PFM.2,11_EnhWk1;	*RNF19A*	Intronic
rs1660346	8	101312497	0.87	0.94	A	G	0.16	0.27	0.42	0.36	0	0	.	.	.	CCIP.LSMPTP,9_TxEnhG1;CD4.MP,11_EnhWk1;CCCRO.MP,11_EnhWk1;CCIP.LSTP,9_T	*RNF19A*	Intronic
rs1788208	8	101312515	0.87	0.94	T	C	0.16	0.27	0.42	0.36	0	0	.	.	.	CCIP.LSMPTP,9_TxEnhG1;CD4.MP,11_EnhWk1;CCCRO.MP,11_EnhWk1;CCIP.LSTP,9_T	*RNF19A*	Intronic
rs2439460	8	101312665	0.88	0.94	C	T	0.62	0.34	0.48	0.36	0	0	.	GM12878,4_Strong_Enhancer	.	CCIP.LSMPTP,9_TxEnhG1;CD4.MP,11_EnhWk1;CCCRO.MP,11_EnhWk1;CCIP.LSTP,9_T	*RNF19A*	Intronic
rs1660347	8	101314980	0.87	0.94	G	A	0.16	0.27	0.42	0.36	0	0	.	GM12878,4_Strong_Enhancer;HepG2,4_Strong_Enhancer	.	CD4.MP,11_EnhWk1;CD8.MP,11_EnhWk1;CCIP.LSMPTP,10_TxEnhG2;SK.MUS,9_TxEnh	*RNF19A*	Intronic
rs1660348	8	101315248	0.88	0.94	A	G	0.62	0.34	0.48	0.36	1	0	GM12878,2_Weak_Promoter	HepG2,4_Strong_Enhancer;H1,7_Weak_Enhancer	CCC.TMP,4_TssWk	CCIP.LSMPTP,10_TxEnhG2;SK.MUS,9_TxEnhG1;BN.AC,9_TxEnhG1;CD3.P,9_TxEnhG1;	*RNF19A*	Intronic
rs1660349	8	101319326	0.85	0.93	G	A	0.16	0.27	0.42	0.36	0	0	GM12878,1_Active_Promoter;HepG2,1_Active_Promoter;NHEK,2_Weak_Promote	HMEC,6_Weak_Enhancer;NHLF,6_Weak_Enhancer;HSMM,6_Weak_Enhancer	CCIP.LSTP,3_TssA;PANC.I,6_TssD2;KID.FE,4_TssWk;BM.MSC,6_TssD2;CD4.NP,6_TssD2;COL.SMUS,6_TssD2;BN.HM150,4	BR.H35,11_EnhWk1;SK.MUS63,14_Enh;R.SMUS,11_EnhWk1;BN.FE0,14_Enh;HRT.FE,13_	*RNF19A*	Intronic
rs1788167	8	101319996	0.88	0.94	C	T	0.62	0.34	0.49	0.36	0	0	GM12878,1_Active_Promoter;HepG2,1_Active_Promoter;K562,2_Weak_Promoter;	HSMM,6_Weak_Enhancer;H1,7_Weak_Enhancer	CCIP.LSTP,3_TssA;BN.AG,4_TssWk;BN.CC,4_TssWk;HUES6,4_TssWk;BR.MYO,6_TssD2;ADI.NUC,2_TssF;CHON.BMMSC,6	SK.MUS63,14_Enh;HRT.FE,13_EnhA;CD34.MBP1480,11_EnhWk1;CD34.P,11_EnhWk1;CD	*RNF19A*	Intronic
rs1660345	8	101322762	0.87	0.94	C	A	0.16	0.27	0.42	0.36	0	0	GM12878,1_Active_Promoter;HepG2,1_Active_Promoter;NHEK,1_Active_Promot	HMEC,4_Strong_Enhancer;Huvec,4_Strong_Enhancer	CCIP.LSTP,3_TssA;CCIP.LSMPTP,3_TssA;PFK.2,3_TssA;HD.CD56MESC,3_TssA;MSC.ADIPC,3_TssA;R.MUC29,3_TssA;BR.I	SK.MUS62,14_Enh	*RNF19A*	.
rs1660344	8	101324659	0.88	0.94	A	G	0.16	0.27	0.42	0.36	0	0	.	.	CCIP.LSTP,3_TssA;CCIP.LSMPTP,3_TssA	CCC.TMP,14_Enh;BN.ITL,14_Enh;ST.SMUS28,14_Enh;CCCRO.MP,14_Enh;HRT.FE,14_En	*RNF19A*	.
rs1788168	8	101324960	0.88	0.95	G	T	0.62	0.34	0.48	0.37	0	0	.	.	CCIP.LSTP,3_TssA;CCIP.LSMPTP,3_TssA	CCC.TMP,14_Enh;ST.SMUS28,14_Enh;CCCRO.MP,14_Enh;HRT.FE,14_Enh;R.MUC31,11_	*RNF19A*	.
rs1788169	8	101326314	0.88	0.94	C	T	0.16	0.27	0.43	0.36	0	0	.	GM12878,5_Strong_Enhancer;HepG2,7_Weak_Enhancer;K562,7_Weak_Enhancer	CCIP.LSTP,2_TssF	HRT.FE,14_Enh;R.MUC31,11_EnhWk1;COL.MUC32,11_EnhWk1;CCCRA.NP,14_Enh;CD3.	*RNF19A*	.
rs1962901	8	101326428	0.88	0.95	A	G	0.62	0.34	0.49	0.37	0	0	.	GM12878,5_Strong_Enhancer;HepG2,7_Weak_Enhancer;K562,7_Weak_Enhancer	.	HRT.FE,14_Enh;R.MUC31,11_EnhWk1;CCCRA.NP,14_Enh;CD3.P,11_EnhWk1;CD34.MBP	*RNF19A*	.
rs1125706	8	101326739	0.88	0.94	T	A	0.16	0.27	0.43	0.36	0	0	.	K562,7_Weak_Enhancer;GM12878,4_Strong_Enhancer;HepG2,6_Weak_Enhancer	.	R.MUC31,11_EnhWk1;CCCRA.NP,14_Enh;CD3.P,11_EnhWk1;CD34.MBP1562,13_EnhA;C	*RNF19A*	.
rs28595052	8	101327553	0.88	0.94	G	A	0.16	0.27	0.43	0.36	0	0	.	GM12878,5_Strong_Enhancer;HepG2,7_Weak_Enhancer	.	CD3.P,11_EnhWk1;CD34.MBP1562,13_EnhA;CD34.P,11_EnhWk1;CD34.C,11_EnhWk1;CD	*RNF19A*	.
rs7846076	8	101327629	0.87	0.94	A	G	0.41	0.31	0.49	0.36	0	0	.	GM12878,5_Strong_Enhancer;HepG2,7_Weak_Enhancer	.	CD34.MBP1562,13_EnhA;CD34.P,11_EnhWk1;CD34.C,11_EnhWk1;CD34.MBP1480,12_Enh	*RNF19A*	.
rs7830342	8	101327738	0.88	0.95	T	A	0.62	0.34	0.49	0.37	0	0	.	GM12878,5_Strong_Enhancer;HepG2,7_Weak_Enhancer	.	CD34.MBP1562,13_EnhA;CD34.P,11_EnhWk1;CD34.C,11_EnhWk1;CD34.MBP1480,12_Enh	*RNF19A*	.
rs1371868	8	101330267	0.88	0.94	C	T	0.17	0.27	0.43	0.36	0	0	.	.	.	LNG.FE,13_EnhA	*RNF19A*	.
rs1660311	8	101330579	0.87	0.94	C	T	0.17	0.27	0.43	0.37	0	0	.	.	.	LNG.FE,13_EnhA	*RNF19A*	.
rs1660312	8	101330634	0.88	0.94	C	G	0.16	0.27	0.43	0.36	0	0	.	.	.	LNG.FE,13_EnhA	*RNF19A*	.
rs1788173	8	101330939	0.88	0.95	C	T	0.62	0.34	0.49	0.37	1	0	.	.	.	LNG.FE,13_EnhA;HD.CD184EC,11_EnhWk1;ST.MUC,11_EnhWk1	*RNF19A*	.
rs1788174	8	101330957	0.88	0.94	G	C	0.16	0.27	0.43	0.36	1	0	.	.	.	LNG.FE,13_EnhA;HD.CD184EC,11_EnhWk1;ST.MUC,11_EnhWk1	*RNF19A*	.
rs962451	8	101331010	0.87	0.94	C	T	0.16	0.27	0.43	0.36	0	0	.	.	.	LNG.FE,13_EnhA;HD.CD184EC,11_EnhWk1;ST.MUC,11_EnhWk1;KID.FE,11_EnhWk1;IPS	*RNF19A*	.
rs962452	8	101331369	0.86	0.93	G	A	0.62	0.34	0.49	0.36	0	0	.	.	.	LNG.FE,11_EnhWk1;NCC.GED2,12_EnhWk2	*RNF19A*	.
rs201467304	8	101331538	0.86	0.93	TTC	T	0.16	0.27	0.43	0.36	0	0	.	.	.	.	*RNF19A*	.
rs11308441	8	101331539	0.86	0.93	TC	T	0.16	0.27	0.43	0.36	0	0	.	.	.	.	*RNF19A*	.
rs2033921	8	101331814	0.87	0.94	A	G	0.62	0.33	0.48	0.36	0	0	.	.	.	.	*RNF19A*	.
rs2033922	8	101331865	0.86	0.93	C	G	0.16	0.27	0.41	0.36	0	0	.	.	.	.	*RNF19A*	.
rs1788175	8	101332089	0.86	0.93	A	G	0.62	0.33	0.48	0.36	0	0	.	.	.	.	*RNF19A*	.
rs1788176	8	101332112	0.86	0.93	A	G	0.16	0.27	0.42	0.36	0	0	.	.	.	.	*RNF19A*	.
rs1788177	8	101332311	0.84	0.92	G	A	0.16	0.27	0.42	0.36	0	0	.	.	.	.	*RNF19A*	.
rs1788178	8	101332518	0.87	0.94	A	C	0.16	0.27	0.43	0.36	0	0	.	.	.	.	*RNF19A*	.
rs1660313	8	101332712	0.87	0.94	G	A	0.16	0.27	0.43	0.36	0	0	.	.	.	CD3.P,14_Enh	*RNF19A*	.
rs1660314	8	101332953	0.86	0.93	C	T	0.16	0.27	0.42	0.36	0	0	.	.	.	CD3.P,14_Enh	*RNF19A*	.
rs200348503	8	101332954	0.81	0.92	ACG	A	0.13	0.27	0.4	0.35	0	0	.	.	.	CD3.P,14_Enh	*RNF19A*	.
rs1660315	8	101332956	0.86	0.93	G	A	0.16	0.27	0.42	0.36	0	0	.	.	.	CD3.P,14_Enh	*RNF19A*	.
rs1660316	8	101333049	0.87	0.94	T	C	0.16	0.27	0.43	0.36	0	0	.	.	.	CD3.P,14_Enh	*RNF19A*	.
rs1660317	8	101333252	0.87	0.94	G	T	0.16	0.27	0.43	0.36	0	0	.	.	.	CD3.P,14_Enh	*RNF19A*	.
rs1788179	8	101333345	0.87	0.94	A	G	0.16	0.27	0.43	0.36	0	0	.	.	.	CD3.P,14_Enh	*RNF19A*	.
rs1788180	8	101333368	0.87	0.94	A	G	0.62	0.34	0.49	0.37	0	0	.	.	.	CD3.P,14_Enh	*RNF19A*	.
rs1788181	8	101334068	0.87	0.94	C	T	0.62	0.34	0.49	0.37	0	0	.	.	.	CCCRA.NP,12_EnhWk2;CD3.P,13_EnhA	*RNF19A*	.
rs1788182	8	101334620	0.87	0.94	C	T	0.62	0.34	0.49	0.37	0	0	.	GM12878,7_Weak_Enhancer	.	CCIP.LSMPTP,12_EnhWk2;CD3.P,14_Enh	*RNF19A*	.
rs76164815	8	101334918	0.87	0.94	C	A	0.62	0.34	0.49	0.37	0	0	.	GM12878,7_Weak_Enhancer	.	CCIP.LSMPTP,12_EnhWk2	*RNF19A*	.
rs1788184	8	101335106	0.87	0.94	A	G	0.62	0.34	0.49	0.37	0	0	.	GM12878,7_Weak_Enhancer	.	CCIP.LSMPTP,12_EnhWk2;KID.FE,14_Enh	*RNF19A*	.
rs1788185	8	101335529	0.87	0.94	T	C	0.16	0.28	0.43	0.36	1	0	.	GM12878,7_Weak_Enhancer;Huvec,7_Weak_Enhancer	.	KID.FE,14_Enh;ADI.NUC,11_EnhWk1;MSC.ADIPC,14_Enh	*RNF19A*	.
rs1788186	8	101335686	0.87	0.94	T	C	0.62	0.34	0.49	0.37	0	0	.	GM12878,5_Strong_Enhancer;Huvec,5_Strong_Enhancer	.	KID.FE,14_Enh;ADI.NUC,11_EnhWk1;LNG.FE,11_EnhWk1;MSC.ADIPC,13_EnhA	*RNF19A*	.
rs1788187	8	101336099	0.87	0.94	A	G	0.62	0.34	0.49	0.37	1	1	.	GM12878,5_Strong_Enhancer;Huvec,5_Strong_Enhancer	.	KID.FE,14_Enh;LNG.FE,11_EnhWk1;MSC.ADIPC,13_EnhA;ADI.NUC,13_EnhA;ADI.MSC,	*RNF19A*	.

rs2439456	8	101336874	0.87	0.94	C	T	0.62	0.34	0.43	0.36	0	0	.	GM12878,7_Weak_Enhancer;Huvec,5_Strong_Enhancer	.	MSC.ADIPC,13_EnhA;ADI.NUC,14_Enh	*RNF19A*	.
rs1788209	8	101337056	0.84	0.91	C	T	0.59	0.33	0.4	0.36	0	0	.	GM12878,7_Weak_Enhancer;Huvec,5_Strong_Enhancer	.	ADI.NUC,14_Enh;R.MUC31,11_EnhWk1;MSC.ADIPC,14_Enh	*RNF19A*	.
rs1660318	8	101337279	0.85	0.94	T	C	0.59	0.33	0.42	0.37	0	0	.	GM12878,7_Weak_Enhancer;Huvec,5_Strong_Enhancer	.	ADI.NUC,14_Enh;MSC.ADIPC,14_Enh	*RNF19A*	.
rs1788210	8	101337312	0.86	0.94	T	A	0.17	0.28	0.42	0.37	0	0	.	GM12878,7_Weak_Enhancer;Huvec,5_Strong_Enhancer	.	ADI.NUC,14_Enh;MSC.ADIPC,14_Enh	*RNF19A*	.
rs1660319	8	101337530	0.86	0.94	A	G	0.62	0.33	0.42	0.37	0	0	.	GM12878,7_Weak_Enhancer;Huvec,7_Weak_Enhancer	.	ADI.NUC,14_Enh;MSC.ADIPC,13_EnhA	*RNF19A*	.
rs1619759	8	101337560	0.86	0.94	T	C	0.16	0.28	0.42	0.37	0	0	.	GM12878,7_Weak_Enhancer;Huvec,7_Weak_Enhancer	.	ADI.NUC,14_Enh;MSC.ADIPC,13_EnhA	*RNF19A*	.
rs1660320	8	101337579	0.86	0.94	A	C	0.62	0.33	0.42	0.37	0	0	.	GM12878,7_Weak_Enhancer;Huvec,7_Weak_Enhancer	.	ADI.NUC,14_Enh;MSC.ADIPC,13_EnhA	*RNF19A*	.
rs1612773	8	101337818	0.86	0.94	G	C	0.62	0.34	0.42	0.37	0	0	.	GM12878,7_Weak_Enhancer;Huvec,7_Weak_Enhancer	.	MSC.ADIPC,13 EnhA;LV,11 EnhWk1;ADI.NUC,13 EnhA;GAS,12 EnhWk2;CCIP.LSTP,1	*RNF19A*	.
**rs142360995**	**8**	**1.18E+08**	**-**	**-**	G	A	0.36	0.19	0.04	0.2	0	0	.	.	.	.	*SLC30A8*	.
rs16889471	8	118190620	0.86	0.98	G	A	0.52	0.22	0.05	0.22	0	0	.	.	.	.	*SLC30A8*	.
rs6469681	8	118203434	0.87	0.99	A	G	0.42	0.22	0.05	0.22	0	0	K562,2_Weak_Promoter	.	HUES6,4_TssWk	.	*SLC30A8*	.
rs189573274	8	118205677	0.88	0.98	G	A	0.41	0.22	0.05	0.21	0	0	.	.	.	.	*SLC30A8*	.
rs10101645	8	118216395	0.8	0.95	T	G	0.29	0.21	0.05	0.22	0	0	.	.	.	PANC.I,11_EnhWk1	*SLC30A8*	.
rs10105491	8	118217271	0.8	0.95	T	C	0.37	0.21	0.05	0.22	0	0	.	.	.	PANC.I,11_EnhWk1	*SLC30A8*	.
**rs10820600**	**9**	**1.07E+08**	**-**	**-**	T	C	0.66	0.39	0.18	0.54	0	0	GM12878,1 Active Promoter;K562,1 Active Promoter;NHEK,1 Active Promoter	.	BN.FE0,5 TssD1;HD.CD56MESC,3 TssA;HUES6,3 TssA;H9,3 TssA;BN.FE1,3 TssA;ST.MUC,3 TssA;ADI.MSC,3 TssA;LV,3	SK.MUS62,14_Enh	*SMC2*	5'-UTR
rs3818625	9	106856452	0.85	0.99	G	A	0.71	0.43	0.19	0.58	0	0	GM12878,1 Active Promoter;K562,1 Active Promoter;NHEK,1 Active Promoter	.	BN.FE0,5 TssD1;HD.CD56MESC,3 TssA;HUES6,3 TssA;H9,3 TssA;BN.FE1,3 TssA;ST.MUC,3 TssA;ADI.MSC,3 TssA;LV,3	SK.MUS62,14_Enh	*SMC2*	.
rs3818626	9	106856633	0.81	0.95	C	T	0.49	0.42	0.17	0.57	0	0	GM12878,1 Active Promoter;K562,1 Active Promoter;NHEK,1 Active Promoter	.	BN.FE0,5 TssD1;HD.CD56MESC,3 TssA;HUES6,3 TssA;H9,3 TssA;BN.FE1,3 TssA;ST.MUC,3 TssA;ADI.MSC,3 TssA;LV,3	SK.MUS62,14_Enh	*SMC2*	5'-UTR
rs10820599	9	106856691	0.95	0.99	T	G	0.68	0.4	0.18	0.55	0	0	GM12878,1 Active Promoter;K562,1 Active Promoter;NHEK,1 Active Promoter	.	BN.FE0,5 TssD1;HD.CD56MESC,3 TssA;HUES6,3 TssA;H9,3 TssA;BN.FE1,3 TssA;ST.MUC,3 TssA;ADI.MSC,3 TssA;LV,3	SK.MUS62,14_Enh	*SMC2*	5'-UTR
rs4742903	9	106856793	0.85	0.99	G	C	0.78	0.43	0.18	0.58	0	1	GM12878,1 Active Promoter;K562,1 Active Promoter;NHEK,1 Active Promoter	.	BN.FE0,5 TssD1;HD.CD56MESC,3 TssA;HUES6,3 TssA;H9,3 TssA;BN.FE1,3 TssA;ST.MUC,3 TssA;ADI.MSC,3 TssA;LV,3	SK.MUS62,14_Enh	*SMC2*	Intronic
rs4743687	9	106856910	0.85	0.99	C	T	0.75	0.43	0.19	0.58	0	0	GM12878,1 Active Promoter;K562,1 Active Promoter;NHEK,1 Active Promoter	.	HD.CD56MESC,3 TssA;HUES6,3 TssA;BN.FE1,3 TssA;ST.MUC,3 TssA;ADI.MSC,3 TssA;LV,3 TssA;BR.H35,3 TssA;IMR90.	SK.MUS62,14_Enh	*SMC2*	5'-UTR; intronic
rs4742904	9	106856952	0.81	0.95	C	T	0.49	0.42	0.17	0.57	0	0	GM12878,1 Active Promoter;K562,1 Active Promoter;NHEK,1 Active Promoter	.	HD.CD56MESC,3 TssA;HUES6,3 TssA;BN.FE1,3 TssA;ST.MUC,3 TssA;ADI.MSC,3 TssA;LV,3 TssA;BR.H35,3 TssA;IMR90.	SK.MUS62,14_Enh	*SMC2*	5'-UTR; intronic
rs4742905	9	106856972	0.85	0.99	G	C	0.71	0.43	0.19	0.58	0	0	GM12878,1 Active Promoter;K562,1 Active Promoter;NHEK,1 Active Promoter	.	HD.CD56MESC,3 TssA;HUES6,3 TssA;BN.FE1,3 TssA;ST.MUC,3 TssA;ADI.MSC,3 TssA;LV,3 TssA;BR.H35,3 TssA;IMR90.	SK.MUS62,14_Enh	*SMC2*	5'-UTR; intronic
rs4742906	9	106857078	0.85	0.99	A	G	0.71	0.43	0.19	0.58	0	0	GM12878,1 Active Promoter;K562,1 Active Promoter;NHEK,1 Active Promoter	.	HD.CD56MESC,3 TssA;HUES6,3 TssA;ADI.MSC,3 TssA;LV,3 TssA;BR.H35,3 TssA;IMR90,3 TssA;PFK.2,3 TssA;BN.FE2,3	SK.MUS62,14_Enh;SPL,14_Enh	*SMC2*	5'-UTR; intronic
rs3739736	9	106857908	0.84	0.99	G	A	0.71	0.42	0.19	0.58	0	0	GM12878,1 Active Promoter;K562,1 Active Promoter;HMEC,1 Active Promoter	H1,6_Weak_Enhancer;HSMM,6_Weak_Enhancer	CD34.MBP1562,2 TssF;LNG.FE,2 TssF;CD34.MBP1549,4 TssWk;PFK.2,2 TssF;R.MUC29,4 TssWk;BM.MSC,6 TssD2;PANC.	CD4.MP,13 EnhA;CD3.P,13 EnhA;CD34.MBP1536,14 Enh;CD4.NP,11 EnhWk1;BR.MYO,	*SMC2*	Intronic
rs3739737	9	106858192	0.83	0.99	A	G	0.71	0.43	0.19	0.58	0	0	GM12878,2_Weak_Promoter;K562,2_Weak_Promoter;NHLF,2_Weak_Promoter	HSMM,6 Weak Enhancer;H1,7 Weak Enhancer;HepG2,6 Weak Enhancer;NHEK,7 Weak Enhancer;Huvec	CHON.BMMSC,6 TssD2;HUES6,4 TssWk;HUES64,4 TssWk;BN.GM2,4 TssWk;DUO.MUC61,4 TssWk;HD.CD184EC,4 TssWl	BR.MYO,14 Enh;ES.WA7,14 Enh;SK.MUS63,11 EnhWk1;CD34.MBP1480,11 EnhWk1;BR	*SMC2*	Intronic
rs10761010	9	106858788	0.84	0.99	G	A	0.71	0.43	0.19	0.58	0	0	GM12878,2_Weak_Promoter	HepG2,6_Weak_Enhancer;HMEC,6_Weak_Enhancer;NHLF,6_Weak_Enhancer;K562,6_Weak_Enhancer	CHON.BMMSC,6_TssD2;BN.GM2,4_TssWk;KID.FE,4_TssWk;IPS.18,4_TssWk	HRT.FE,11 EnhWk1;LNG.FE,14 Enh;CD34.C,11 EnhWk1;CD4.MP,11 EnhWk1;PFM.3,11	*SMC2*	Intronic
rs11330299	9	106859701	0.8	0.94	TA	T	0.58	0.42	0.17	0.57	0	0	.	GM12878,6_Weak_Enhancer	.	.	*SMC2*	Intronic
rs7048372	9	106859738	0.8	0.94	T	C	0.58	0.42	0.17	0.57	0	0	.	GM12878,6_Weak_Enhancer	.	.	*SMC2*	Intronic
rs2274724	9	106860568	0.82	0.98	C	T	0.79	0.43	0.19	0.58	0	0	.	GM12878,6_Weak_Enhancer	.	.	*SMC2*	Intronic
rs10820601	9	106861281	0.86	0.96	G	A	0.76	0.4	0.17	0.56	0	0	.	.	.	.	*SMC2*	Intronic
rs10820602	9	106861282	0.82	0.91	C	T	0.54	0.39	0.15	0.55	0	0	.	.	.	.	*SMC2*	Intronic
rs2122577	9	106866703	0.82	0.98	C	T	0.79	0.43	0.19	0.58	0	0	.	.	.	.	*SMC2*	Intronic
rs139217198	9	106872864	0.81	0.96	A	ATGT	0.71	0.44	0.19	0.58	0	0	.	.	.	.	*SMC2*	Intronic
rs7854630	9	106875277	0.8	0.97	G	T	0.71	0.43	0.19	0.58	0	0	.	.	.	.	*SMC2*	Intronic
rs10820603	9	106877939	0.8	0.96	G	A	0.79	0.43	0.19	0.58	0	0	.	.	.	.	*SMC2*	Intronic
**rs2901157**	**10**	**1.19E+08**	**-**	**-**	A	G	0.02	0.07	0.26	0.13	0	0	.	.	.	H1.DNP,11 EnhWk1;BN.CC,12 EnhWk2;BN.FE2,12 EnhWk2;BN.FE0,15 EnhP;PFF.1,15 I	*EMX2OS*	Intronic
rs12770891	10	119256834	0.8	0.91	G	T	0.34	0.2	0.26	0.14	0	0	.	HepG2,7_Weak_Enhancer	.	COL.SMUS,12_EnhWk2	*EMX2OS*	Intronic
**rs10838267**	**11**	**44368892**	**-**	**-**	G	A	0.11	0.35	0.32	0.51	0	0	.	K562,6_Weak_Enhancer	.	BN.FE2,14 Enh;IPS.18,12 EnhWk2;H1.DNP,11 EnhWk1;BN.FE0,12 EnhWk2;NCC.COR2,	*ALX4*	.
rs4755814	11	44366518	0.85	0.99	T	C	0.7	0.4	0.39	0.55	0	0	.	.	.	BN.FE2,14_Enh;BN.FE1,12_EnhWk2;BN.FE0,12_EnhWk2	*ALX4*	.
rs3913042	11	44367045	0.86	0.99	A	G	0.72	0.41	0.38	0.54	0	0	.	.	.	BN.FE2,14_Enh;BR.MYO,11_EnhWk1	*ALX4*	.
rs7125755	11	44367897	0.93	1	C	G	0.29	0.36	0.32	0.53	0	0	.	.	.	BN.FE2,14_Enh;IPS.DF19,11_EnhWk1;BR.MYO,14_Enh	*ALX4*	.
rs10838266	11	44368032	0.85	0.98	A	C	0.7	0.4	0.38	0.54	0	0	.	K562,7_Weak_Enhancer	.	BN.FE2,14_Enh;IPS.DF19,11_EnhWk1;BR.MYO,14_Enh	*ALX4*	.
rs4755815	11	44368381	0.87	1	A	G	0.71	0.4	0.39	0.54	0	0	.	K562,7_Weak_Enhancer	.	BN.FE2,14_Enh;BR.MYO,11_EnhWk1	*ALX4*	.
rs4755816	11	44368416	0.93	1	G	T	0.31	0.36	0.33	0.53	0	0	.	K562,7_Weak_Enhancer	.	BN.FE2,14_Enh;ES.WA7,15_EnhP;IPS.18,12_EnhWk2;H1.DNP,11_EnhWk1	*ALX4*	.
rs6485512	11	44369196	0.92	1	A	G	0.7	0.4	0.38	0.53	0	0	.	K562,7_Weak_Enhancer	.	BN.FE2,14_Enh;H1.DNP,11_EnhWk1;BN.FE0,12_EnhWk2;NCC.COR2,15_EnhP	*ALX4*	.
rs3853291	11	44369477	0.99	1	G	A	0.29	0.36	0.32	0.51	0	0	.	.	.	BN.FE2,14_Enh;H1.DNP,11_EnhWk1;NCC.COR2,15_EnhP	*ALX4*	.
rs2863089	11	44370034	0.92	1	C	T	0.69	0.39	0.38	0.53	0	0	.	.	.	BN.FE2,14_Enh;BR.MYO,14_Enh	*ALX4*	.
rs6485513	11	44370140	0.92	1	C	T	0.62	0.38	0.38	0.53	0	0	.	.	.	BN.FE2,14_Enh;BR.MYO,14_Enh	*ALX4*	.
rs10742705	11	44371717	0.9	0.99	A	G	0.68	0.41	0.38	0.53	0	0	.	.	.	.	*ALX4*	.
rs4755817	11	44372916	0.96	0.98	G	A	0.1	0.35	0.31	0.51	0	0	.	GM12878,4_Strong_Enhancer	CD19.P,6_TssD2	ESO,12_EnhWk2	*ALX4*	.
**rs78588049**	**12**	**69180907**	**-**	**-**	ATTTT	A	0.05	0.17	0.14	0.16	0	1	.	HepG2,4 Strong Enhancer;Huvec,5 Strong Enhancer;HMEC,5 Strong Enhancer;GM12878,7 Weak Enhan	.	CD15.P,13 EnhA;PFK.3,11 EnhWk1;PFF.1,11 EnhWk1;ADI.NUC,12 EnhWk2;MUS.SC,11	*SLC35E3*	.
**rs855596**	**12**	**1.03E+08**	**-**	**-**	T	C	1	0.99	0.93	0.95	0	0	.	.	.	.	*LOC283432*	.
rs78693353	12	102965986	0.88	-1	C	A	0	0.03	0.15	0.04	1	0	.	.	.	.	*IGF1*	.
rs61937477	12	102972154	0.88	-1	C	A	0	0.03	0.13	0.04	0	0	.	.	.	.	*IGF1*	.
rs61937479	12	102977074	0.88	-1	G	A	0	0.03	0.13	0.04	0	0	.	.	.	ST.MUC,12_EnhWk2	*IGF1*	.
rs11611928	12	102979757	0.85	-0.97	A	G	0	0.03	0.13	0.04	0	0	.	.	.	.	*IGF1*	.
rs61937482	12	102980979	0.88	-1	T	C	0	0.03	0.13	0.04	0	0	.	.	.	.	*IGF1*	.
rs1840959	12	102982041	0.88	-1	G	A	0	0.03	0.13	0.04	0	0	.	.	.	.	*IGF1*	.
rs61941038	12	102989316	0.85	-0.97	A	T	0	0.03	0.13	0.04	0	0	.	K562,7_Weak_Enhancer	.	IPS.DF19,12_EnhWk2	*IGF1*	.
rs61941039	12	102989606	0.85	-0.97	T	C	0	0.03	0.13	0.04	0	0	.	.	.	IPS.DF19,12_EnhWk2;PFF.1,15_EnhP	*IGF1*	.
rs61941040	12	102989710	0.85	-0.97	A	C	0	0.03	0.13	0.04	0	0	.	.	.	IPS.DF19,12_EnhWk2;PFF.1,15_EnhP	*IGF1*	.
rs17032884	12	102995192	0.85	-0.97	A	G	0	0.03	0.13	0.04	0	0	.	.	.	CHON.BMMSC,12_EnhWk2	*IGF1*	.
rs17032899	12	102997164	0.85	-0.97	T	C	0	0.03	0.13	0.04	0	0	.	.	.	.	*IGF1*	.
rs1096914	12	103001065	0.85	-0.97	T	G	0.002	0.03	0.13	0.04	0	0	.	.	.	.	*IGF1*	.
rs833469	12	103002657	0.85	-0.97	T	C	0.09	0.04	0.13	0.04	0	0	.	.	.	.	*IGF1*	.
rs833472	12	103005100	0.88	-1	C	T	0	0.03	0.13	0.04	0	0	.	H1,7_Weak_Enhancer	.	HUES6,12 EnhWk2;ES.I3,11 EnhWk1;CHON.BMMSC,12 EnhWk2;HUES64,12 EnhWk2;H	*IGF1*	.
rs833463	12	103007185	0.85	-0.97	A	G	0	0.03	0.13	0.04	0	0	.	.	.	H1,14_Enh;IPS.18,12_EnhWk2;HUES48,11_EnhWk1;HUES64,12_EnhWk2	*IGF1*	.
rs1070208	12	103008745	0.85	-0.97	A	G	0	0.03	0.13	0.04	0	0	.	.	.	.	*IGF1*	.
rs703551	12	103009144	0.85	-0.97	G	A	0	0.03	0.13	0.04	0	0	.	.	.	.	*IGF1*	.
rs703553	12	103010139	0.85	-0.97	C	A	0	0.03	0.13	0.04	0	0	.	.	.	.	*IGF1*	.
rs703555	12	103011455	0.85	-0.97	T	A	0	0.03	0.13	0.04	0	0	.	.	.	.	*IGF1*	.
rs703556	12	103011894	0.88	-1	A	G	0	0.03	0.13	0.04	0	0	.	.	.	.	*IGF1*	.
rs703557	12	103012049	0.88	-1	A	G	0	0.03	0.13	0.04	0	0	.	.	.	.	*IGF1*	.
rs703560	12	103014414	0.88	-1	A	G	0	0.03	0.13	0.04	0	0	.	.	.	.	*IGF1*	.
rs703563	12	103020031	0.88	-1	A	G	0	0.04	0.13	0.04	0	0	.	.	.	.	*IGF1*	.
rs703564	12	103020623	0.85	-0.97	A	C	0	0.01	0.06	0.04	0	0	.	.	.	.	*IGF1*	.
rs703567	12	103021142	0.88	-1	A	G	0	0.01	0.07	0.04	0	0	.	.	.	.	*IGF1*	.
rs703568	12	103021298	0.85	-1	A	T	0	0.01	0.06	0.04	0	0	.	.	.	.	*IGF1*	.
rs697195	12	103023753	0.88	-1	A	G	0	0.01	0.07	0.04	0	0	.	K562,7_Weak_Enhancer;HSMM,5_Strong_Enhancer;NHLF,6_Weak_Enhancer	.	MSC.ADIPC,13 EnhA;ADI.MSC,13 EnhA;BM.MSC,13 EnhA;MUS.SC,12 EnhWk2;CHON	*IGF1*	.
rs703576	12	103025828	0.88	-1	C	G	0	0.01	0.07	0.04	0	0	.	HSMM,5_Strong_Enhancer	.	ADI.MSC,13_EnhA;BM.MSC,13_EnhA	*IGF1*	.
rs833728	12	103029988	0.88	-1	A	C	0	0.01	0.07	0.04	0	0	.	.	.	.	*IGF1*	.
rs703579	12	103030938	0.88	-1	A	C	0	0.01	0.07	0.04	0	0	.	.	.	.	*IGF1*	.
rs703581	12	103032097	0.88	-1	T	C	0	0.01	0.07	0.04	0	0	.	.	.	.	*IGF1*	.
rs1070209	12	103033217	0.91	-1	A	G	0.02	0.02	0.07	0.04	0	0	.	.	.	.	*IGF1*	.
rs703584	12	103034820	0.88	-1	C	A	0	0.01	0.07	0.04	0	0	.	.	.	.	*IGF1*	.
rs703585	12	103035129	0.88	-1	C	T	0	0.01	0.07	0.04	0	0	.	.	.	.	*IGF1*	.
rs703587	12	103036245	0.88	-1	A	T	0	0.01	0.07	0.04	0	0	.	.	.	ST.MUC,11_EnhWk1;LIV.A,11_EnhWk1	*IGF1*	.
rs703589	12	103038955	0.88	-1	T	C	0	0.01	0.07	0.04	0	0	.	.	.	ST.MUC,11_EnhWk1	*LOC283432*	.
rs2643673	12	103042298	0.88	1	T	C	0.99	0.99	0.92	0.96	0	0	.	.	.	.	*LOC283432*	.
rs833732	12	103043313	0.88	1	A	G	1	0.99	0.93	0.96	0	0	.	.	.	.	*LOC283432*	.
rs833733	12	103044411	0.91	1	C	T	1	0.99	0.93	0.96	0	0	.	.	.	.	*LOC283432*	.
rs833734	12	103044493	0.91	1	A	G	1	0.99	0.93	0.96	0	0	.	.	.	.	*LOC283432*	.
rs1495389	12	103045157	0.91	1	C	T	1	0.99	0.93	0.96	0	0	.	.	.	.	*LOC283432*	.
rs833736	12	103046430	0.88	1	C	G	1	0.99	0.93	0.96	0	0	.	.	.	.	*LOC283432*	.
rs833737	12	103046442	0.88	1	G	A	1	0.99	0.93	0.96	0	0	.	.	.	.	*LOC283432*	.
rs833738	12	103047220	0.88	1	G	A	1	0.99	0.93	0.96	0	0	.	GM12878,7_Weak_Enhancer	.	.	*LOC283432*	.
rs833740	12	103047582	0.88	1	T	C	1	0.99	0.93	0.96	0	0	.	.	.	.	*LOC283432*	.
rs833742	12	103051885	0.91	1	A	C	1	0.99	0.93	0.96	0	0	.	.	.	.	*LOC283432*	.
rs1102946	12	103053121	0.88	1	C	T	1	0.99	0.93	0.96	0	0	.	.	.	.	*LOC283432*	.
rs2132249	12	103056076	0.88	1	G	A	0.92	0.98	0.93	0.96	0	0	.	.	.	.	*LOC283432*	.
rs1845680	12	103057592	0.88	1	T	A	1	0.99	0.93	0.96	0	0	.	.	.	.	*LOC283432*	.
rs833707	12	103062685	0.88	1	A	T	1	0.99	0.93	0.96	0	0	.	.	.	BM.MSC,12_EnhWk2	*LOC283432*	.
rs200397453	12	103064636	0.85	0.94	A	AT	0.99	0.98	0.92	0.96	0	0	.	.	.	.	*LOC283432*	.
rs833708	12	103064743	0.88	1	C	T	1	0.99	0.93	0.96	0	0	.	.	.	.	*LOC283432*	.
rs833710	12	103065094	0.88	1	G	A	1	0.99	0.93	0.96	0	0	.	.	.	.	*LOC283432*	.
rs833711	12	103065246	0.88	1	A	G	1	0.99	0.93	0.96	0	0	.	.	.	.	*LOC283432*	.
rs833712	12	103066119	0.88	1	A	G	1	0.99	0.93	0.96	0	0	.	HSMM,7_Weak_Enhancer	.	.	*LOC283432*	.
rs833722	12	103073597	0.88	1	G	C	0.98	0.98	0.92	0.96	0	0	.	.	.	.	*LOC283432*	.
rs2643694	12	103079901	0.88	1	G	A	1	0.99	0.92	0.96	1	0	.	.	.	ADI.MSC,12_EnhWk2	*LOC283432*	.
rs2647874	12	103080067	0.88	1	T	C	1	0.99	0.92	0.96	0	0	.	.	.	ADI.MSC,12_EnhWk2;MSC.ADIPC,12_EnhWk2;ST.MUC,12_EnhWk2	*LOC283432*	.
rs1495390	12	103081707	0.88	1	G	A	1	0.99	0.92	0.96	0	0	.	.	.	ST.MUC,12_EnhWk2;BM.MSC,12_EnhWk2	*LOC283432*	.
rs2643693	12	103082161	0.88	1	G	A	0.98	0.98	0.92	0.96	0	0	.	.	.	.	*LOC283432*	.
**rs9316500**	**13**	**51094114**	**-**	**-**	T	G	0.27	0.27	0.66	0.29	0	0	.	.	.	MSC.ADIPC,11 EnhWk1;PFM.1,11 EnhWk1;PFM.2,14 Enh;BN.CC,14 Enh;LNG.FE,11 Er	*DLEU7*	.
rs9562987	13	51095908	0.88	1	G	A	0.46	0.37	0.77	0.31	0	0	.	NHLF,6_Weak_Enhancer;HepG2,6_Weak_Enhancer;HSMM,6_Weak_Enhancer	MSC.ADIPC,6_TssD2;LNG.FE,6_TssD2	BN.SN,11 EnhWk1;COL.SMUS,11 EnhWk1;R.MUC31,11 EnhWk1;KID.FE,11 EnhWk1;BI	*DLEU7*	.
rs963740	13	51096095	1	1	A	T	0.27	0.27	0.66	0.29	1	1	.	NHLF,6_Weak_Enhancer;HepG2,6_Weak_Enhancer;HSMM,7_Weak_Enhancer	MSC.ADIPC,6_TssD2;SK.MUS,6_TssD2	COL.SMUS,11 EnhWk1;R.MUC31,11 EnhWk1;KID.FE,11 EnhWk1;BN.ITL,11 EnhWk1;S	*DLEU7*	.
**rs75004998**	**14**	**77517786**	**-**	**-**	G	A	0.08	0.27	0.49	0.34	0	0	HepG2,2_Weak_Promoter	HSMM,5_Strong_Enhancer;NHLF,5_Strong_Enhancer;H1,7_Weak_Enhancer;HMEC,6_Weak_Enhancer	SK.MUS63,6 TssD2;PFF.1,6 TssD2;IPS.DF19,6 TssD2;BR.MYO,6 TssD2;ST.SMUS28,6 TssD2;COL.SMUS,6 TssD2;SK.MUS	PFM.3,11 EnhWk1;HRT.FE,13 EnhA;BN.GM2,11 EnhWk1;LNG.FE,13 EnhA;BN.AG,11 E	*IRF2BPL*	Intronic
rs10873301	14	77507205	0.82	0.98	G	T	0.07	0.26	0.48	0.3	0	1	H1,2_Weak_Promoter;K562,1_Active_Promoter	HSMM,4 Strong Enhancer;HMEC,4 Strong Enhancer;NHEK,4 Strong Enhancer;Huvec,4 Strong Enhance	CD34.MBP1562,2 TssF;R.SMUS,6 TssD2;BM.MSC,2 TssF;CD15.P,2 TssF;ES.WA7,4 TssWk;SK.MUS,6 TssD2;MUS.SC,2 Ts	CD34.MBP1536,13 EnhA;GAS,14 Enh;SK.MUS62,13 EnhA;BR.H35,13 EnhA;SPL,14 Enh	*IRF2BPL*	.
rs11159247	14	77509872	0.82	0.98	G	A	0.08	0.26	0.48	0.3	1	1	.	HSMM,4 Strong Enhancer;NHLF,4 Strong Enhancer;H1,7 Weak Enhancer;NHEK,4 Strong Enhancer;Huv	ADI.MSC,2 TssF;SK.MUS,6 TssD2;CD34.MBP1562,2 TssF;PFM.2,2 TssF;BN.AC,2 TssF;BN.CC,2 TssF;BN.ITL,2 TssF;CD15	CD34.MBP1536,13 EnhA;SK.MUS62,13 EnhA;BN.AG,13 EnhA;PFM.3,13 EnhA;ADI.NUC	*IRF2BPL*	Intronic
rs55715661	14	77513173	0.95	0.98	C	T	0.08	0.27	0.5	0.34	0	0	.	Huvec,7 Weak Enhancer;HMEC,5 Strong Enhancer;GM12878,7 Weak Enhancer;K562,7 Weak Enhancer;	.	LV,13 EnhA;PFK.3,13 EnhA;R.MUC29,13 EnhA;DUO.MUC61,13 EnhA;LNG.FE,13 EnhA	*IRF2BPL*	Intronic
rs17105227	14	77513800	0.95	0.98	G	A	0.08	0.27	0.5	0.34	0	0	.	HepG2,7_Weak_Enhancer	.	CD34.MBP1480,11 EnhWk1;CD34.MBP1562,11 EnhWk1;CD15.P,11 EnhWk1;CD34.MBP1	*IRF2BPL*	Intronic
rs11622562	14	77517148	0.96	1	C	T	0.08	0.27	0.49	0.33	0	0	.	.	.	LIV.A,11 EnhWk1;NCC.GED2,11 EnhWk1;PFM.2,13 EnhA;BN.AC,14 Enh;PFF.1,11 Enh	*IRF2BPL*	Intronic
rs205810	14	77517894	0.98	1	G	A	0.35	0.29	0.49	0.34	0	0	.	HepG2,6 Weak Enhancer;K562,7 Weak Enhancer;HSMM,7 Weak Enhancer;HMEC,7 Weak Enhancer;NH	PFF.1,6_TssD2;PFF.2,6_TssD2;BN.ITL,6_TssD2;ST.SMUS28,4_TssWk	HRT.FE,13 EnhA;BN.GM2,11 EnhWk1;LNG.FE,13 EnhA;BN.AG,11 EnhWk1;CHON.BMN	*IRF2BPL*	Intronic
rs8022640	14	77524587	0.82	0.94	G	A	0.23	0.24	0.45	0.32	1	1	.	.	.	NCC.GED2,11_EnhWk1;BN.GM2,11_EnhWk1;BN.FE0,11_EnhWk1	*IRF2BPL*	Intronic
rs17105272	14	77529783	0.81	0.94	C	T	0.23	0.24	0.45	0.32	0	0	.	GM12878,7_Weak_Enhancer	.	IPS.DF19,11 EnhWk1;BN.FE0,11 EnhWk1;BN.ITL,14 Enh;BN.FE2,11 EnhWk1;BN.AG,14	*IRF2BPL*	Intronic
**rs8027365**	**15**	**75808740**	**-**	**-**	A	C	0.61	0.28	0.38	0.27	0	0	.	.	.	MSC.ADIPC,11_EnhWk1	*PTPN9*	Intronic
rs4886707	15	75755467	0.82	0.91	C	T	0.55	0.28	0.38	0.27	0	0	.	H1,6_Weak_Enhancer	.	H9,14 Enh;IPS.DF19,14 Enh;HUES48,11 EnhWk1;ES.I3,11 EnhWk1;HUES64,11 EnhWk1;	*PTPN9*	.
rs4545784	15	75755651	0.82	0.91	A	G	0.56	0.28	0.38	0.27	0	0	.	.	.	IPS.DF19,14_Enh;HUES48,11_EnhWk1;HUES64,11_EnhWk1;H1,14_Enh;HUES6,14_Enh	*PTPN9*	.
rs4886708	15	75762608	0.83	0.93	C	T	0.61	0.29	0.5	0.27	0	0	.	.	.	.	*PTPN9*	Intronic
rs8034317	15	75764343	0.82	0.92	G	A	0.61	0.29	0.5	0.27	0	0	.	.	.	.	*PTPN9*	Intronic
rs11634863	15	75766672	0.84	0.94	C	A	0.56	0.27	0.38	0.26	0	0	.	.	.	HD.CD184EC,9_TxEnhG1	*PTPN9*	Intronic
rs28432989	15	75768297	0.8	0.89	T	C	0.61	0.31	0.5	0.27	0	0	.	.	.	.	*PTPN9*	Intronic
rs28737338	15	75768679	0.83	0.93	C	T	0.61	0.29	0.49	0.27	0	0	.	.	.	.	*PTPN9*	Intronic
rs4886446	15	75770521	0.83	0.93	G	T	0.61	0.29	0.49	0.27	0	0	.	.	.	.	*PTPN9*	Intronic
rs111534199	15	75778310	0.8	0.93	G	A	0.6	0.27	0.34	0.26	0	0	.	.	.	.	*PTPN9*	Intronic
rs12708518	15	75783051	0.85	0.93	T	C	0.61	0.28	0.49	0.27	0	0	.	.	.	.	*PTPN9*	Intronic
rs10584118	15	75784091	0.85	0.96	TCAGA	T	0.53	0.27	0.37	0.26	0	0	.	.	.	.	*PTPN9*	Intronic
rs7495610	15	75784441	0.88	0.96	T	C	0.6	0.28	0.38	0.26	0	0	.	.	.	.	*PTPN9*	Intronic
rs4299117	15	75789444	0.88	0.96	C	T	0.59	0.27	0.38	0.26	0	0	.	.	.	.	*PTPN9*	Intronic
rs11637586	15	75791112	0.88	0.96	G	C	0.59	0.27	0.38	0.26	0	0	.	.	.	.	*PTPN9*	Intronic

rs12916964	15	75792297	0.88	0.96	T	A	0.53	0.27	0.38	0.26	0	0	.	.	.	.	*PTPN9*	Intronic
rs11638974	15	75799306	0.83	0.94	G	A	0.54	0.26	0.49	0.26	0	0	.	.	.	KID.FE,9_TxEnhG1	*PTPN9*	Intronic
rs4381565	15	75802154	0.81	0.94	A	G	0.59	0.26	0.38	0.26	0	0	.	.	.	.	*PTPN9*	Intronic
rs12708519	15	75803200	0.9	0.97	T	C	0.61	0.26	0.38	0.26	0	0	.	.	.	.	*PTPN9*	Intronic
rs11639183	15	75807692	0.85	0.96	T	C	0.61	0.26	0.38	0.26	0	0	.	.	.	.	*PTPN9*	Intronic
rs8027802	15	75808731	0.97	0.99	G	A	0.59	0.27	0.38	0.27	0	0	.	.	.	MSC.ADIPC,11_EnhWk1	*PTPN9*	Intronic
rs8027546	15	75808791	0.85	0.96	C	T	0.6	0.27	0.38	0.26	0	0	.	Huvec,7_Weak_Enhancer	.	MSC.ADIPC,11_EnhWk1	*PTPN9*	Intronic
rs8029112	15	75808972	0.9	0.97	T	C	0.61	0.26	0.38	0.26	0	0	.	Huvec,7_Weak_Enhancer	.	MSC.ADIPC,11_EnhWk1;PFF.2,11_EnhWk1;IMR90,9_TxEnhG1;PFF.1,11_EnhWk1	*PTPN9*	Intronic
rs4322627	15	75810659	0.9	0.97	C	T	0.61	0.26	0.38	0.26	0	0	.	.	.	PFF.1,9_TxEnhG1;PFF.2,9_TxEnhG1;H1.DMSC,9_TxEnhG1;PFM.1,9_TxEnhG1	*PTPN9*	Intronic
rs199993688	15	75811158	0.85	0.95	A	AG	0.57	0.27	0.37	0.26	0	0	.	.	.	.	*PTPN9*	Intronic
rs7180574	15	75811599	0.9	0.97	A	G	0.61	0.26	0.38	0.26	0	0	.	.	.	.	*PTPN9*	Intronic
rs28610581	15	75812474	0.9	0.97	A	G	0.58	0.26	0.38	0.26	0	0	.	.	.	.	*PTPN9*	Intronic
rs147396934	15	75812817	0.86	0.97	AGATAGATG	A	0.49	0.24	0.34	0.25	0	0	.	.	.	.	*PTPN9*	Intronic
rs4886712	15	75812888	0.88	0.96	C	T	0.61	0.26	0.39	0.27	0	0	.	.	.	.	*PTPN9*	Intronic
rs8030802	15	75813153	0.9	0.97	A	G	0.61	0.26	0.38	0.26	0	0	.	.	.	H1.DMSC,9_TxEnhG1	*PTPN9*	Intronic
rs6495182	15	75814388	0.9	0.97	C	T	0.55	0.26	0.38	0.26	0	0	.	.	.	H1.DMSC,9_TxEnhG1	*PTPN9*	Intronic
rs8023268	15	75814672	0.9	0.97	A	T	0.61	0.26	0.38	0.26	0	0	.	.	.	H1.DMSC,9_TxEnhG1	*PTPN9*	Intronic
rs8023815	15	75814704	0.9	0.97	G	A	0.61	0.26	0.38	0.26	0	0	.	.	.	H1.DMSC,9_TxEnhG1	*PTPN9*	Intronic
rs11636031	15	75815758	0.9	0.97	T	C	0.61	0.26	0.38	0.26	0	0	.	.	.	H1.DMSC,9_TxEnhG1;CHON.BMMSC,9_TxEnhG1	*PTPN9*	Intronic
rs7176938	15	75817115	0.88	0.97	T	C	0.61	0.26	0.38	0.26	0	0	.	.	.	.	*PTPN9*	Intronic
rs8036758	15	75817562	0.9	0.97	G	A	0.59	0.26	0.38	0.26	0	0	.	.	.	.	*PTPN9*	Intronic
rs8024244	15	75822230	0.9	0.97	G	A	0.55	0.26	0.38	0.26	0	0	.	.	.	LIV.A,14_Enh	*PTPN9*	Intronic
rs11636199	15	75825337	0.82	0.92	A	G	0.62	0.27	0.5	0.27	1	0	.	HSMM,5_Strong_Enhancer;Huvec,4_Strong_Enhancer;HMEC,5_Strong_Enhancer;NHEK,5_Strong_Enhance	MUS.SC,6_TssD2;PFF.1,2_TssF	LIV.A,13_EnhA;BM.MSC,11_EnhWk1;R.SMUS,11_EnhWk1;SK.MUS,11_EnhWk1;BN.SN,1	*PTPN9*	Intronic
rs12909863	15	75825822	0.82	0.92	G	C	0.6	0.27	0.49	0.27	0	0	.	HMEC,5_Strong_Enhancer;NHEK,5_Strong_Enhancer	.	LIV.A,13_EnhA;R.SMUS,11_EnhWk1;SK.MUS,11_EnhWk1;BN.SN,14_Enh;BR.MYO,11_Er	*PTPN9*	Intronic
rs12708520	15	75830539	0.82	0.92	C	T	0.61	0.27	0.5	0.27	0	0	.	.	.	.	*PTPN9*	Intronic
rs4886715	15	75831449	0.84	0.94	C	T	0.61	0.26	0.38	0.26	0	0	.	.	.	.	*PTPN9*	Intronic
rs60972074	15	75833786	0.8	0.93	C	T	0.6	0.26	0.37	0.26	0	0	.	.	.	ST.MUC,14_Enh	*PTPN9*	Intronic
rs62027181	15	75839952	0.8	0.91	A	G	0.61	0.27	0.49	0.27	0	0	.	GM12878,7_Weak_Enhancer	.	BN.MFL,11_EnhWk1	*PTPN9*	Intronic
rs7163907	15	75845097	0.81	0.93	T	C	0.59	0.26	0.38	0.26	0	0	.	K562,7_Weak_Enhancer	DUO.MUC61,6_TssD2	MSC.ADIPC,11_EnhWk1;GAS,11_EnhWk1;LIV.A,11_EnhWk1;CD15.P,11_EnhWk1	*PTPN9*	Intronic
rs4886716	15	75856022	0.8	0.92	G	C	0.57	0.26	0.38	0.26	0	0	.	.	.	.	*PTPN9*	Intronic
**rs76535198**	**16**	**71892498**	-	-	A	C	0.1	0.14	0.31	0.13	0	0	.	.	.	ADI.NUC,11_EnhWk1;SK.MUS62,14_Enh;SK.MUS63,14_Enh;H1.DMSC,13_EnhA;LV,13_E	*ATXN1L*	Intronic
rs142561957	16	71862092	0.85	0.93	G	GGAAC	0.1	0.13	0.27	0.13	0	0	.	.	.	PFM.2,12_EnhWk2	*ATXN1L*	.
rs199811113	16	71862096	0.8	0.94	C	CGA,CGAAC	0.09	0.12	0.24	0.12	0	0	.	.	.	PFM.2,12_EnhWk2	*ATXN1L*	.
rs112791888	16	71862662	0.88	0.97	C	G	0.1	0.14	0.28	0.14	0	0	.	.	.	.	*ATXN1L*	.
rs113070273	16	71873883	0.92	0.97	C	T	0.1	0.14	0.28	0.13	0	0	.	.	.	.	*ATXN1L*	.
rs112121746	16	71874983	0.95	0.99	G	A	0.1	0.14	0.28	0.13	0	0	.	GM12878,7_Weak_Enhancer	.	.	*ATXN1L*	.
rs113584314	16	71882860	0.95	1	G	A	0.1	0.14	0.28	0.14	0	0	.	.	.	SK.MUS62,9_TxEnhG1;SK.MUS63,9_TxEnhG1;IPS.DF19,9_TxEnhG1	*ATXN1L*	Intronic
rs200761004	16	71891983	0.91	0.99	T	TG	0.09	0.13	0.29	0.12	0	0	.	.	.	CC.TPC,9_TxEnhG1;ADI.NUC,11_EnhWk1;SK.MUS62,14_Enh;SK.MUS63,11_EnhWk1	*ATXN1L*	Intronic
rs142336745	16	71905997	0.92	0.99	G	A	0.1	0.14	0.31	0.14	0	0	.	GM12878,7_Weak_Enhancer	.	.	*ZNF821*	Intronic
rs3794691	16	71908441	0.92	0.99	T	C	0.1	0.14	0.3	0.14	0	0	.	.	.	.	*ZNF821*	Intronic
rs112452788	16	71911198	0.88	0.97	C	T	0.1	0.13	0.3	0.14	0	0	.	.	.	.	*ZNF821*	Intronic
rs112012852	16	71911785	0.9	0.99	T	A	0.1	0.14	0.3	0.14	0	0	.	.	.	.	*ZNF821*	Intronic
rs149288672	16	71916281	0.89	0.95	CAG	C	0.1	0.09	0.27	0.13	0	0	GM12878,2_Weak_Promoter	K562,7_Weak_Enhancer;Huvec,6_Weak_Enhancer	R.MUC29,4_TssWk;CCC.TREGP,4_TssWk;R.SMUS,4_TssWk;PANC.I,4_TssWk;BN.FE2,3_TssA;BN.FE1,3_TssA;CD3.P,2_TssF	CD34.MBP1480,11_EnhWk1;PFM.2,11_EnhWk1;CD8.NP,11_EnhWk1;CCC.TMP,14_Enh;LI	*ZNF821*	Intronic
rs142428477	16	71936253	0.89	0.98	A	G	0.11	0.14	0.31	0.14	0	0	.	GM12878,6_Weak_Enhancer	.	CD4.NP,11_EnhWk1;CD8.NP,11_EnhWk1;CD4.MP,11_EnhWk1;SK.MUS63,14_Enh;CCC.T	*KIAA0174*	Intronic
rs61523290	16	71943556	0.88	0.98	A	G	0.1	0.14	0.32	0.14	0	0	.	.	.	CD4.NP,11_EnhWk1;CCIP.LSTP,11_EnhWk1;CD4.MP,11_EnhWk1;CD8.NP,11_EnhWk1;C	*KIAA0174*	Intronic
rs57807129	16	71948043	0.84	0.95	T	C	0.1	0.14	0.3	0.14	0	0	.	Huvec,7_Weak_Enhancer	.	ADI.NUC,14_Enh;CHON.BMMSC,11_EnhWk1;CCIP.LSMPTP,11_EnhWk1;LIV.A,11_EnhV	*KIAA0174*	Intronic
rs1049794	16	71950450	0.83	0.94	C	T	0.1	0.14	0.31	0.14	0	1	.	.	.	BN.AG,11_EnhWk1;SK.MUS63,13_EnhA;BN.HM150,11_EnhWk1;CCIP.LSTP,11_EnhWk1;	*KIAA0174*	SYN
rs72801786	16	71955085	0.84	0.95	C	T	0.1	0.14	0.31	0.14	0	0	.	.	.	PANC.I,9_TxEnhG1;SK.MUS62,9_TxEnhG1	*KIAA0174*	Intronic
rs72801789	16	71959748	0.82	0.94	C	T	0.1	0.14	0.32	0.14	0	0	.	.	.	ADI.NUC,9_TxEnhG1;H1.DMSC,9_TxEnhG1;SK.MUS,9_TxEnhG1;PFM.3,9_TxEnhG1	*KIAA0174*	Intronic
rs74559371	16	71964361	0.82	0.94	CAT	C	0.1	0.14	0.31	0.14	0	0	.	.	.	BR.H35,9_TxEnhG1;ADI.MSC,9_TxEnhG1;HD.CD56MESC,9_TxEnhG1;BN.AC,9_TxEnhG1	*PKD1L3*	Intronic
rs149587742	16	71964975	0.81	0.94	GTTTA	G	0.1	0.14	0.31	0.14	0	0	.	.	.	HD.CD56MESC,9_TxEnhG1;COL.SMUS,9_TxEnhG1;DUO.SMUS,14_Enh;PANC.I,11_EnhV	*PKD1L3*	Intronic
rs16973500	16	71965196	0.82	0.94	C	T	0.1	0.14	0.31	0.14	0	0	.	.	.	DUO.SMUS,14_Enh;R.MUC31,11_EnhWk1	*PKD1L3*	Intronic
rs72801797	16	71975300	0.83	0.94	A	G	0.1	0.14	0.31	0.14	0	0	.	.	.	.	*PKD1L3*	Intronic
**rs12481286**	**20**	**52287610**	-	-	G	T	0.05	0.22	0.35	0.23	0	0	.	.	.	R.MUC29,11_EnhWk1	*ZNF217*	.
rs6063975	20	52278275	0.96	0.98	C	G	0.05	0.22	0.35	0.23	0	0	.	GM12878,4_Strong_Enhancer;HepG2,4_Strong_Enhancer;NHLF,6_Weak_Enhancer	CD8.MP,6_TssD2	SPL,14_Enh;GAS,14_Enh;HUES48,11_EnhWk1;ESO,14_Enh;CD34.MBP1536,11_EnhWk1;H	*ZNF217*	.
rs112582910	20	52285250	0.99	1	A	G	0.05	0.21	0.34	0.23	0	0	.	.	.	.	*ZNF217*	.
rs12624860	20	52285882	0.99	1	C	G	0.05	0.21	0.35	0.23	0	0	.	HepG2,7_Weak_Enhancer	.	R.MUC31,11_EnhWk1;DUO.MUC61,14_Enh;DUO.SMUS,14_Enh;ST.MUC,11_EnhWk1	*ZNF217*	.
rs200998131	20	52287492	0.93	0.98	A	AG	0.08	0.2	0.34	0.22	0	0	.	.	.	.	*ZNF217*	.
rs6068614	20	52289702	0.8	0.99	C	A	0.03	0.2	0.31	0.2	0	0	.	HSMM,7_Weak_Enhancer	MUS.SC,2_TssF	PFF.1,11_EnhWk1;PFM.2,11_EnhWk1;BM.MSC,11_EnhWk1;H1.DMSC,11_EnhWk1;IMR90	*ZNF217*	.
rs13039563	20	52296849	0.94	0.97	G	A	0.04	0.22	0.33	0.23	0	0	.	HepG2,4_Strong_Enhancer;H1,7_Weak_Enhancer;Huvec,7_Weak_Enhancer;NHLF,7_Weak_Enhancer	HD.CD184EC,4_TssWk	MSC.ADIPC,11_EnhWk1;HUES48,11_EnhWk1;CC.TPC,11_EnhWk1;HD.CD56MESC,12_Er	*ZNF217*	.
rs6068620	20	52298091	0.82	0.96	A	G	0.03	0.2	0.32	0.21	0	0	.	.	.	CD34.P,14_Enh;CD15.P,11_EnhWk1	*ZNF217*	.
**rs35418111**	**21**	**47856670**	-	-	G	A	0.04	0.11	0.19	0.07	0	0	.	.	.	BN.AC,9_TxEnhG1;CD3.P,9_TxEnhG1;BN.SN,9_TxEnhG1;PFF.2,9_TxEnhG1;CD8.NP,11_E	*PCNT*	Intronic
rs57385578	21	47850178	0.88	0.96	C	T	0.01	0.11	0.18	0.07	0	0	.	.	.	BN.AC,9_TxEnhG1	*PCNT*	Intronic
**rs34331122**	**22**	**19762428**	-	-	C	CTT	0.71	0.61	0.59	0.42	0	0	.	K562,4_Strong_Enhancer	.	CD8.MP,14_Enh;IPS.DF19,11_EnhWk1;CD4.MP,14_Enh	*TBX1*	Intronic
rs4819521	22	19765322	0.87	0.97	C	T	0.7	0.61	0.61	0.4	0	0	.	K562,7_Weak_Enhancer	.	.	*TBX1*	Intronic
rs5746826	22	19767051	0.85	0.95	G	T	0.7	0.6	0.63	0.4	1	0	.	HSMM,7_Weak_Enhancer	.	IMR90,12_EnhWk2;ADI.MSC,12_EnhWk2;BM.MSC,14_Enh;SK.MUS,14_Enh;PFF.1,12_Enl	*TBX1*	3'-UTR; intronic
