# Supplementary material for: Identification of novel breast cancer susceptibility loci in meta-analyses conducted among Asian and European descendants
Source: Nat Commun. 2020 Mar 5;11:1217. doi: 10.1038/s41467-020-15046-w (PMC7057957; doi:10.1038/s41467-020-15046-w)
Supplement: Supplementary file 8 — Supplementary Data 5 [file 41467_2020_15046_MOESM8_ESM.pdf]

Supplementary Data 5. Replication of breast cancer susceptibility SNPs previously reported

Locus	SNP	Chr	Position	Test/other	OR $_{Asian}$	95% CI $_{Asian}$	P $_{Asian}$	FPRP$_{Asian}$ [a]	OR $_{Asian+EA}$	95% CI $_{Asian+EA}$	P $_{Asian+EA}$	FPRP$_{Asian+EA}$ [b]	OR $_{literature}$	Gene
SNPs initially reported in Asians														
1p22.3	rs12118297	1	87779217	T/G	0.95	(0.93-0.98)	8.1×10^{-4}	0.843	0.97	(0.96-0.99)	7.0×10^{-5}	0.316	0.91	*LMO4*
1q32.1	rs4951011	1	203766331	G/A	1.05	(1.03-1.08)	2.3×10^{-4}	0.604	1.04	(1.03-1.06)	1.8×10^{-8}	1.1E-04	1.06	*ZC3H11A*
5q14.3	rs10474352	5	90732225	C/T	1.07	(1.04-1.10)	3.9×10^{-7}	2.6E-03	1.06	(1.05-1.08)	1.1×10^{-16}	6.9E-13	1.09	*ARRDC3*
6q25.1	rs9485372	6	149608874	G/A	1.08	(1.05-1.11)	2.4×10^{-8}	1.6E-04	1.05	(1.03-1.06)	7.6×10^{-12}	5.2E-08	1.11	*TAB2*
6q25.1	rs2046210	6	151948366	A/G	1.23	(1.20-1.27)	8.0×10^{-53}	2.7E-49	1.11	(1.10-1.13)	3.2×10^{-74}	1.1E-70	1.29	*ESR1*
10q21.2	rs10822013	10	64251977	T/C	1.07	(1.05-1.10)	8.8×10^{-8}	5.9E-04	1.06	(1.05-1.07)	2.9×10^{-27}	9.7E-24	1.10	*ZNF365*
11q24.3	rs7107217	11	129473690	C/A	1.06	(1.04-1.09)	6.2×10^{-6}	0.040	1.05	(1.04-1.06)	3.9×10^{-17}	1.3E-13	1.08	*BARX2*
15q26.1	rs2290203	15	91512067	G/A	1.10	(1.07-1.13)	2.6×10^{-13}	1.8E-09	1.06	(1.05-1.08)	2.4×10^{-19}	8.1E-16	1.08	*PRC1*
16q12.1	rs4784227	16	52599188	T/C	1.22	(1.19-1.26)	1.7×10^{-40}	5.6E-37	1.24	(1.22-1.25)	2.1×10^{-240}	6.8E-237	1.25	*TOX3*
21q22.12	rs16992204	21	36111201	C/T	1.13	(1.08-1.17)	7.8×10^{-10}	5.2E-06	1.13	(1.08-1.17)	7.8×10^{-10}	5.2E-06	1.13	*LINC00160*
22q13.1	rs12628403	22	39358037	C/A	1.08	(1.05-1.12)	1.1×10^{-6}	7.4E-03	1.09	(1.07-1.11)	3.1×10^{-17}	1.0E-13	1.17	*APOBEC3A, APOBEC3B*
SNPs initially reported in Euopreans														
1p36.22	rs616488	1	10566215	A/G	1.06	(1.03-1.09)	5.8×10^{-5}	0.280	1.06	(1.05-1.07)	1.6×10-23	5.4E-20	1.06	*PEX14*
1p36.13	rs2992756	1	18807339	T/C	1.04	(1.01-1.08)	0.015	0.990	1.05	(1.04-1.06)	5.2×10-17	1.7E-13	1.05	*KLHDC7A*
1p34.2	rs4233486	1	41380440	T/C	1.02	(0.99-1.05)	0.309	1.000	1.04	(1.02-1.05)	1.6×10-08	1.0E-04	1.04	
1p34.1	rs1707302	1	46600917	A/G	0.96	(0.93-0.98)	0.002	0.938	0.96	(0.95-0.97)	3.4×10-10	2.2E-06	0.96	*PIK3R3, LOC101929626*
1p32.3	rs140850326	1	50846032	CAAAGGGCAAGATC TCCTTTTT/C	1.00	(0.96-1.04)	0.964	1.000	1.03	(1.02-1.04)	2.5×10-07	1.7E-03	1.03	
1p22.3	rs17426269	1	88156923	A/G	1.03	(0.81-1.32)	0.798	1.000	1.05	(1.03-1.07)	1.5×10-08	9.8E-05	1.05	
1p13.2	rs11552449	1	114448389	T/C	1.01	(0.98-1.04)	0.440	1.000	1.04	(1.03-1.06)	1.2×10-09	7.7E-06	1.06	*DCLRE1B*
1p12	rs7529522	1	118230221	T/C	0.98	(0.96-1.01)	0.208	0.999	0.96	(0.95-0.97)	5.1×10-10	3.3E-06	0.95	
1p11.2	rs11249433	1	121280613	A/G	0.89	(0.82-0.97)	0.005	0.973	0.91	(0.89-0.92)	7.8×10-54	2.6E-50	0.91	*EMBP1*
1q21.1	rs12405132	1	145644984	T/C	1.00	(0.96-1.04)	0.983	1.000	0.96	(0.95-0.98)	5.2×10-09	3.5E-05	0.96	*RNF115*
1q21.2	rs12048493	1	149927034	A/C	0.97	(0.94-1.00)	0.062	0.998	0.95	(0.94-0.97)	3.6×10-14	2.3E-10	0.95	*OTUD7B*
1q22	rs4971059	1	155148781	A/G	1.01	(0.98-1.04)	0.651	1.000	1.04	(1.03-1.05)	2.2×10-10	1.5E-06	1.04	*TRIM46*
1q32.1	rs6678914	1	202187176	A/G	0.98	(0.94-1.01)	0.135	0.999	0.99	(0.98-1.00)	1.3×10-01	0.999	0.99	*LGR6*
1q32.1	rs4245739	1	204518842	A/C	1.00	(0.94-1.06)	0.927	1.000	0.97	(0.96-0.99)	1.7×10-04	0.526	0.97	*MDM4*
1q41	rs11117758	1	217220574	A/G	0.98	(0.93-1.04)	0.581	1.000	0.96	(0.94-0.97)	4.4×10-09	3.0E-05	0.96	*ESRRG*
2p25.1	rs113577745	2	10135681	C/G	1.18	(0.81-1.71)	0.388	1.000	0.94	(0.92-0.96)	5.0×10-10	3.4E-06	0.94	*GRHL1*
2p24.1	rs12710696	2	19320803	T/C	1.05	(1.02-1.08)	0.001	0.878	1.04	(1.03-1.05)	6.3×10-11	4.4E-07	1.04	
2p23.3	rs200648189	2	24739694	CT/C	1.03	(0.97-1.09)	0.348	1.000	1.02	(1.00-1.04)	2.3×10-02	0.994	1.02	*NCOA1*
2p23.3	rs6725517	2	25129473	A/G	1.01	(0.98-1.04)	0.477	1.000	1.04	(1.03-1.05)	4.5×10-11	3.0E-07	1.05	*ADCY3*
2p23.2	rs4577244	2	29120733	T/C	1.00	(0.97-1.03)	0.929	1.000	1.00	(0.98-1.01)	4.9×10-01	1.000	0.99	*WDR43*
2q13	rs71801447	2	111925731	CTTATGTT/C	0.98	(0.94-1.03)	0.467	1.000	0.94	(0.92-0.96)	2.3×10-07	1.5E-03	0.93	*BCL2L11*
2q14.1	rs4849887	2	121245122	T/C	0.92	(0.89-0.95)	4.8×10^{-7}	0.003	0.91	(0.90-0.93)	2.1×10-25	6.9E-22	0.91	
2q31.1	rs2016394	2	172972971	A/G	0.97	(0.94-1.01)	0.102	0.999	0.96	(0.95-0.97)	2.6×10-12	1.7E-08	0.96	*DLX2-AS1*
2q31.1	rs1550623	2	174212894	A/G	1.04	(0.92-1.17)	0.522	1.000	1.05	(1.04-1.07)	5.6×10-10	3.7E-06	1.05	*CDCA7*
2q33.1	rs1830298	2	202181247	T/C	0.91	(0.89-0.94)	6.7×10^{-10}	4.5E-06	0.94	(0.93-0.95)	6.1×10-24	2.0E-20	0.95	*CASP8/ ALS2CR12*
2q35	rs4442975	2	217920769	T/G	0.94	(0.90-0.97)	0.001	0.885	0.88	(0.87-0.89)	2.3×10-97	7.5E-94	0.88	*IGFBP5*
2q35	rs34005590	2	217963060	A/C	1.72	(0.88-3.37)	0.114	0.999	0.82	(0.79-0.84)	5.3×10-41	1.8E-37	0.81	*IGFBP5*
2q35	rs16857609	2	218296508	T/C	1.07	(1.05-1.10)	1.2×10^{-7}	8.3E-04	1.07	(1.06-1.09)	2.2×10-31	7.2E-28	1.08	*DIRC3*
2q36.3	rs12479355	2	227226952	A/G	1.01	(0.97-1.06)	0.503	1.000	1.04	(1.03-1.05)	3.8×10-08	2.6E-04	1.04	
3p26.1	rs6762644	3	4742276	A/G	0.96	(0.92-1.01)	0.126	0.999	0.95	(0.94-0.96)	1.0×10-18	3.5E-15	0.95	*EGOT/ITPR1*
3p24.1	rs4973768	3	27416013	T/C	1.08	(1.05-1.12)	6.0×10^{-7}	4.0E-03	1.10	(1.09-1.11)	4.9×10-62	1.6E-58	1.10	*SLC4A7*
3p.24.1	rs12493607	3	30682939	C/G	1.05	(1.02-1.08)	7.3×10^{-4}	0.829	1.05	(1.04-1.06)	2.6×10-16	1.8E-12	1.05	*TGFBR2*
3p21.31	rs6796502	3	46866866	A/G	0.95	(0.92-0.99)	0.009	0.984	0.93	(0.91-0.95)	7.1×10-16	4.8E-12	0.92	
3p14.1	rs1053338	3	63967900	A/G	0.99	(0.95-1.02)	0.454	1.000	0.95	(0.94-0.97)	6.7×10-10	4.4E-06	0.94	*ATNX7*
3p13	rs6805189	3	71532113	T/C	1.02	(0.89-1.17)	0.738	1.000	1.03	(1.02-1.05)	4.4×10-08	3.0E-04	1.03	*FOXP1*

3p12.1	rs9833888	3	99723580	T/G	1.03	(0.99-1.07)	0.136	0.999	1.04	(1.03-1.06)	3.1×10-10	2.1E-06	1.05	*CMSS1, FILIP1L*
3q23	rs34207738	3	141112859	CTT/C	0.97	(0.92-1.03)	0.332	1.000	0.95	(0.94-0.96)	2.1×10-15	1.3E-11	0.95	*ZBTB38*
3q26.31	rs58058861	3	172285237	A/G	1.04	(1.01-1.07)	0.006	0.976	1.05	(1.03-1.06)	3.4×10-12	2.2E-08	1.05	
4p14	rs6815814	4	38816338	A/C	0.99	(0.96-1.02)	0.510	1.000	0.96	(0.95-0.97)	1.3×10-11	9.3E-08	0.95	
4q21.23	4:84370124	4	84370124	TA/TAA	0.97	(0.94-1.00)	0.061	0.998	0.96	(0.95-0.97)	3.7×10-10	2.4E-06	0.96	*HELQ*
4q22.1	rs10022462	4	89243818	T/C	1.12	(1.02-1.23)	0.020	0.992	1.04	(1.03-1.05)	3.0×10-10	1.9E-06	1.04	*LOC105369192*
4q24	rs9790517	4	106084778	T/C	1.01	(0.98-1.03)	0.636	1.000	1.04	(1.03-1.05)	1.5×10-09	9.9E-06	1.05	*TET2*
4q28.1	rs77528541	4	126843504	T/G	0.78	(0.58-1.05)	0.108	0.999	0.94	(0.93-0.96)	7.2×10-10	4.8E-06	0.94	
4q34.1	rs6828523	4	175846426	A/C	0.94	(0.92-0.97)	1.9×10^{-4}	0.555	0.91	(0.90-0.93)	4.7×10-27	1.6E-23	0.90	*ADAM29*
5p15.33	rs116095464	5	345109	T/C	1.06	(0.84-1.33)	0.648	1.000	0.92	(0.90-0.95)	5.8×10-09	3.9E-05	0.92	*AHRR*
5p15.33	rs10069690	5	1279790	T/C	1.08	(1.04-1.11)	3.5×10^{-5}	0.187	1.06	(1.05-1.08)	2.0×10-20	6.5E-17	1.06	*TERT*
5p15.33	rs3215401	5	1296255	A/AG	1.05	(1.02-1.09)	8.5×10^{-4}	0.849	1.07	(1.05-1.08)	3.0×10-23	1.0E-19	1.07	*TERT*
5p15.1	rs13162653	5	16187528	T/G	1.02	(0.99-1.06)	0.227	0.999	0.97	(0.96-0.99)	1.1×10-05	0.068	0.97	
5p13.3	rs2012709	5	32567732	T/C	1.00	(0.96-1.04)	0.975	1.000	1.03	(1.02-1.05)	5.5×10-08	3.6E-04	1.04	
5p12	rs10941679	5	44706498	A/G	0.91	(0.89-0.94)	4.1×10^{-11}	2.7E-07	0.89	(0.88-0.90)	1.3×10-80	4.5E-77	0.88	*FGF10, MRPS30*
5q11.1	rs35951924	5	50195093	A/AT	0.99	(0.95-1.04)	0.774	1.000	1.05	(1.03-1.06)	2.5×10-10	1.7E-06	1.05	
5q11.2	rs62355902	5	56053723	A/T	0.95	(0.93-0.98)	0.001	0.872	0.87	(0.86-0.88)	2.4×10-86	7.9E-83	0.84	*MAP3K1*
5q11.2	rs10472076	5	58184061	T/C	1.00	(0.97-1.03)	0.941	1.000	0.97	(0.96-0.98)	1.7×10-07	1.1E-03	0.96	*RAB3C*
5q11.2	rs1353747	5	58337481	T/G	1.09	(0.79-1.51)	0.609	1.000	1.06	(1.04-1.09)	3.3×10-09	2.2E-05	1.06	*PDE4D*
5q14.2	rs7707921	5	81538046	A/T	1.08	(0.93-1.25)	0.314	1.000	1.05	(1.04-1.07)	1.3×10-12	9.1E-09	1.05	*ATG10*
5q22.1	rs6882649	5	111217786	T/G	1.04	(1.00-1.08)	0.049	0.997	1.04	(1.03-1.05)	5.7×10-10	3.7E-06	1.04	*NREP*
5q31.1	rs6596100	5	132407058	T/C	0.99	(0.95-1.03)	0.672	1.000	0.96	(0.95-0.97)	2.1×10-08	1.4E-04	0.96	*HSPA4*
5q33.3	rs1432679	5	158244083	T/C	0.93	(0.90-0.95)	1.3×10^{-7}	8.7E-04	0.93	(0.92-0.94)	5.1×10-37	1.7E-33	0.93	*EBF1*
5q35.1	rs4562056	5	169591487	T/G	1.02	(0.99-1.05)	0.140	0.999	1.04	(1.03-1.05)	4.6×10-10	3.0E-06	1.04	
6p25.3	rs11242675	6	1318878	T/C	0.99	(0.97-1.02)	0.530	1.000	1.02	(1.01-1.03)	1.2×10-03	0.894	1.03	*FOXQ1*
6p24.3	rs9348512	6	10456706	A/C	1.03	(0.99-1.07)	0.105	0.999	1.01	(0.99-1.02)	4.2×10-01	1.000	1.00	*TFAP2A*
6p23	rs204247	6	13722523	A/G	0.97	(0.94-0.99)	0.010	0.985	0.96	(0.95-0.97)	3.0×10-14	1.9E-10	0.96	*RANBP9*
6p22.3	rs3819405	6	16399557	T/C	1.01	(0.98-1.04)	0.704	1.000	0.97	(0.96-0.98)	5.6×10-07	3.7E-03	0.96	*ATXN1*
6p22.3	rs2223621	6	20621238	T/C	1.00	(0.97-1.02)	0.809	1.000	1.03	(1.02-1.05)	2.0×10-08	1.3E-04	1.04	*CDKAL1*
6p22.1	rs9257408	6	28926220	C/G	1.01	(0.98-1.03)	0.613	1.000	1.03	(1.02-1.04)	3.0×10-07	1.9E-03	1.03	
6q14.1	rs12207986	6	81094287	A/G	1.03	(1.00-1.07)	0.046	0.997	1.04	(1.03-1.05)	2.0×10-10	1.3E-06	1.04	
6q14.1	rs17529111	6	82128386	T/C	0.97	(0.94-1.00)	0.073	0.998	0.96	(0.95-0.97)	3.4×10-10	2.2E-06	0.96	
6q23.1	rs6569648	6	130349119	T/C	1.03	(0.97-1.09)	0.317	1.000	1.05	(1.04-1.07)	1.9×10-12	1.2E-08	1.05	*L3MBTL3*
6q25.1	rs2747652	6	152437016	T/C	0.96	(0.94-0.99)	0.003	0.951	0.94	(0.93-0.95)	5.9×10-28	2.0E-24	0.94	*ESR1*
7p15.3	rs7971	7	21940960	A/G	1.00	(0.97-1.03)	0.997	1.000	1.03	(1.02-1.04)	1.6×10-07	1.1E-03	1.04	*DNAH11, CDCA7L*
7p15.1	rs17156577	7	28356889	T/C	1.02	(0.95-1.10)	0.633	1.000	0.95	(0.93-0.97)	2.3×10-08	1.5E-04	0.94	*CREB5*
7q21.2	rs6964587	7	91630620	T/G	1.05	(1.01-1.08)	0.009	0.984	1.04	(1.03-1.05)	2.7×10-12	1.8E-08	1.04	*AKAP9*
7q21.3	rs17268829	7	94113799	T/C	0.95	(0.92-0.98)	0.003	0.956	0.95	(0.94-0.96)	4.1×10-15	2.7E-11	0.95	
7q22.1	rs71559437	7	101552440	A/G	0.94	(0.88-1.01)	0.087	0.998	0.93	(0.92-0.95)	9.7×10-13	6.3E-09	0.93	*CUX1*
7q32.3	rs4593472	7	130667121	T/C	0.95	(0.92-0.98)	0.004	0.960	0.96	(0.94-0.97)	2.4×10-13	1.6E-09	0.96	*FLJ43663*
7q34	rs11977670	7	139942304	A/G	1.05	(1.02-1.08)	2.1×10^{-4}	0.590	1.05	(1.04-1.07)	1.1×10-19	3.8E-16	1.05	
7q35	rs720475	7	144074929	A/G	0.99	(0.92-1.07)	0.765	1.000	0.95	(0.94-0.97)	1.9×10-11	1.3E-07	0.95	*NOBOX, ARHGEF6*
8p23.3	rs66823261	8	170692	T/C	0.95	(0.92-0.99)	0.010	0.986	0.96	(0.94-0.97)	1.1×10-08	7.4E-05	0.96	*RPL23AP53*
8p12	rs9693444	8	29509616	A/C	1.07	(1.04-1.10)	6.1×10^{-6}	0.039	1.07	(1.05-1.08)	8.1×10-26	2.7E-22	1.06	
8p11.23	rs13365225	8	36858483	A/G	1.06	(1.03-1.09)	1.6×10^{-4}	0.518	1.07	(1.06-1.09)	1.4×10-23	4.7E-20	1.08	
8q21.11	rs6472903	8	76230301	T/G	1.14	(1.07-1.22)	8.8×10^{-5}	0.370	1.08	(1.07-1.10)	1.1×10-23	3.7E-20	1.08	
8q21.11	rs2943559	8	76417937	A/G	1.01	(0.97-1.06)	0.574	1.000	0.91	(0.90-0.93)	6.9×10-19	2.3E-15	0.89	*HNF4G*
8q22.3	rs514192	8	102478959	A/T	1.05	(1.02-1.08)	3.2×10^{-4}	0.681	1.04	(1.03-1.05)	1.2×10-11	8.1E-08	1.04	
8q23.1	rs12546444	8	106358620	A/T	1.06	(1.01-1.12)	0.016	0.991	1.07	(1.05-1.09)	3.9×10-12	2.6E-08	1.07	*ZFPM3*
8q23.3	rs13267382	8	117209548	A/G	1.03	(1.00-1.06)	0.028	0.995	1.04	(1.03-1.05)	2.0×10-12	1.3E-08	1.04	*LINC00536*
8q24.13	rs58847541	8	124610166	A/G	1.03	(1.00-1.07)	0.076	0.998	1.06	(1.04-1.07)	4.6×10-13	3.0E-09	1.06	
8q24.13	rs17350191	8	124757661	T/C	1.05	(1.01-1.08)	0.013	0.988	1.04	(1.03-1.05)	1.1×10-10	7.6E-07	1.04	*ANXA13*
8q24.21	rs13281615	8	128355618	A/G	0.96	(0.94-0.99)	0.002	0.934	0.91	(0.90-0.92)	2.3×10-55	7.7E-52	0.90	
8q24.21	rs11780156	8	129194641	T/C	0.98	(0.95-1.01)	0.226	0.999	1.05	(1.03-1.06)	1.5×10-09	9.8E-06	1.06	*MYC*

9p21.3	rs1011970	9	22062134	T/G	1.03	(0.99-1.08)	0.145	0.999	1.06	(1.05-1.08)	6.4×10-16	4.1E-12	1.07	*CDKN2A,CDKN2B*
9q31.2	rs10759243	9	110306115	A/C	1.05	(1.02-1.08)	2.9×10^{-4}	0.658	1.06	(1.05-1.07)	3.4×10-21	1.1E-17	1.06	
9q31.2	rs10816625	9	110837073	A/G	0.91	(0.89-0.94)	1.6×10^{-10}	1.1E-06	0.90	(0.89-0.92)	7.7×10-27	2.6E-23	0.90	
9q31.2	rs13294895	9	110837176	T/C	1.03	(0.94-1.13)	0.562	1.000	1.07	(1.05-1.09)	7.1×10-17	2.4E-13	1.07	
9q31.2	rs676256	9	110895353	T/C	1.05	(0.98-1.12)	0.194	0.999	1.10	(1.09-1.12)	2.8×10-53	9.5E-50	1.10	
9q33.1	rs1895062	9	119313486	A/G	1.01	(0.98-1.04)	0.476	1.000	1.04	(1.03-1.06)	1.1×10-13	7.4E-10	1.05	*ASTN2*
9q33.3	rs10760444	9	129396434	A/G	0.93	(0.91-0.96)	1.0×10^{-7}	6.7E-04	0.96	(0.95-0.97)	8.3×10-14	5.3E-10	0.96	*LMX1B*
9q34.2	rs8176636	9	136151579	T/TGGTGCAGGCGC AGGAAAAAATTGTG GCAATTCCTCA	1.04	(1.01-1.08)	0.014	0.989	1.05	(1.03-1.06)	4.9×10-10	3.2E-06	1.05	*ABO*
10p15.1	rs2380205	10	5886734	T/C	0.97	(0.93-1.01)	0.135	0.999	0.98	(0.97-0.99)	5.3×10-05	2.6E-01	0.98	*ANKRD16*
10p14	rs67958007	10	9088113	T/TG	1.01	(0.96-1.08)	0.638	1.000	1.06	(1.04-1.08)	4.5×10-10	3.1E-06	1.06	
10p12.31	rs7072776	10	22032942	A/G	1.01	(0.95-1.08)	0.677	1.000	1.06	(1.05-1.08)	2.4×10-19	8.1E-16	1.06	*DNAJC1*
10p12.31	rs11814448	10	22315843	A/C	0.90	(0.77-1.05)	0.179	0.999	0.84	(0.80-0.87)	4.0×10-18	1.3E-14	0.83	*DNAJC1*
10q21.2	rs10995201	10	64299890	A/G	1.08	(0.99-1.18)	0.100	0.998	1.14	(1.12-1.16)	5.1×10-52	1.7E-48	1.14	*ZNF365*
10q22.3	rs704010	10	80841148	T/C	1.05	(1.03-1.08)	2.0×10^{-4}	0.575	1.08	(1.06-1.09)	3.4×10-38	1.1E-34	1.08	*ZMZ1*
10q23.33	rs140936696	10	95292187	CAA/C	1.02	(0.99-1.05)	0.192	0.999	1.04	(1.03-1.06)	5.4×10-08	3.6E-04	1.05	
10q25.2	rs7904519	10	114773927	A/G	0.98	(0.91-1.05)	0.529	1.000	0.96	(0.94-0.97)	1.8×10-13	1.2E-09	0.96	*TCFL2*
10q26.12	rs11199914	10	123093901	T/C	0.97	(0.94-0.99)	0.019	0.992	0.96	(0.95-0.97)	4.2×10-13	2.9E-09	0.96	
10q26.13	rs2981578	10	123340311	T/C	0.83	(0.80-0.85)	6.5×10^{-46}	2.2E-42	0.81	(0.81-0.82)	2.3×10-291	7.6E-288	0.81	*FGFR2*
10q26.13	rs45631563	10	123349324	A/T	0.95	(0.77-1.17)	0.617	1.000	1.21	(1.18-1.25)	1.1×10-35	3.7E-32	1.22	*FGFR2*
11p15	rs6597981	11	803017	A/G	0.98	(0.95-1.01)	0.253	0.999	0.96	(0.95-0.97)	2.5×10-12	1.8E-08	0.96	*PIDD1*
11p15.5	rs3817198	11	1909006	T/C	0.94	(0.91-0.98)	0.002	0.922	0.94	(0.93-0.95)	5.5×10-21	1.8E-17	0.94	*LSP1*
11q13.1	rs3903072	11	65583066	T/G	0.98	(0.95-1.02)	0.351	1.000	0.96	(0.95-0.97)	5.7×10-12	3.8E-08	0.96	
11q13.3	rs75915166	11	69379161	A/C	1.26	(1.00-1.60)	0.054	0.997	1.30	(1.27-1.33)	1.3×10-96	4.3E-93	1.30	*CCND1*
11q22.3	rs11374964	11	108345515	G/GA	1.02	(0.99-1.04)	0.277	0.999	1.00	(0.99-1.01)	6.5×10-01	1.0E+00	1.00	*KDELC2*
11q24.3	rs11820646	11	129461171	T/C	0.97	(0.95-1.00)	0.025	0.994	0.96	(0.95-0.97)	3.3×10-15	2.2E-11	0.95	
12p13.1	rs12422552	12	14413931	C/G	1.08	(1.05-1.11)	6.0×10^{-7}	4.0E-03	1.06	(1.05-1.07)	2.4×10-20	7.9E-17	1.06	
12p11.22	rs7297051	12	28174817	T/C	0.89	(0.86-0.91)	3.9×10^{-14}	2.6E-10	0.89	(0.87-0.90)	5.5×10-72	1.8E-68	0.89	
12q21.31	rs202049448	12	85009437	T/C	0.99	(0.96-1.03)	0.711	1.000	1.03	(1.02-1.05)	8.5×10-07	5.6E-03	1.04	
12q22	rs17356907	12	96027759	A/G	1.05	(1.02-1.08)	0.002	0.935	1.09	(1.07-1.10)	1.3×10-40	4.3E-37	1.09	*NTN4*
12q24.21	rs1292011	12	115836522	A/G	1.10	(1.07-1.13)	1.4×10^{-10}	9.4E-07	1.09	(1.08-1.10)	1.0×10-47	3.4E-44	1.09	*TBX3*
12q24.31	rs206966	12	120832146	T/C	1.02	(0.96-1.09)	0.496	1.000	1.05	(1.03-1.07)	5.0×10-08	3.4E-04	1.05	
13q22.1	rs6562760	13	73957681	A/G	0.98	(0.81-1.20)	0.881	1.000	0.96	(0.94-0.97)	1.3×10-09	8.9E-06	0.96	
14q13.3	rs2236007	14	37132769	A/G	0.93	(0.91-0.96)	6.4×10^{-6}	0.041	0.93	(0.92-0.94)	1.1×10-25	3.6E-22	0.93	*PAX9*
14q24.1	rs2588809	14	68660428	T/C	1.08	(1.00-1.17)	0.058	0.997	1.07	(1.05-1.08)	1.3×10-14	8.6E-11	1.06	*RAD51B*
14q24.1	rs999737	14	69034682	T/C	1.12	(0.86-1.46)	0.403	1.000	0.91	(0.90-0.92)	1.2×10-38	4.0E-35	0.91	*RAD51B*
14q32.11	rs941764	14	91841069	A/G	0.96	(0.93-1.00)	0.050	0.997	0.96	(0.94-0.97)	1.7×10-13	1.2E-09	0.95	*CCDC88C*
14q32.12	rs11627032	14	93104072	T/C	1.02	(0.99-1.05)	0.307	1.000	1.04	(1.03-1.06)	2.0×10-10	1.3E-06	1.05	*RIN3*
14q32.33	rs10623258	14	105212261	CTT/C	1.01	(0.98-1.04)	0.565	1.000	1.03	(1.02-1.04)	7.9×10-08	5.4E-04	1.04	*ADSSL1*
16p13.3	rs11076805	16	4106788	A/C	0.99	(0.94-1.04)	0.630	1.000	0.97	(0.96-0.98)	3.2×10-05	0.177	0.97	*ADCY9*
16q12.2	rs17817449	16	53813367	T/G	1.07	(1.03-1.11)	6.2×10^{-4}	0.805	1.06	(1.05-1.07)	5.4×10-24	1.8E-20	1.06	*FTO*
16q12.2	rs11075995	16	53855291	A/T	1.05	(1.02-1.08)	9.2×10^{-4}	0.861	1.04	(1.03-1.06)	3.2×10-11	2.2E-07	1.04	*FTO*
16q12.2	rs28539243	16	54682064	A/G	1.03	(1.01-1.06)	0.018	0.992	1.05	(1.04-1.06)	1.1×10-15	7.2E-12	1.05	
16q13	rs2432539	16	56420987	A/G	1.05	(1.02-1.08)	0.001	0.880	1.04	(1.03-1.05)	2.7×10-10	1.7E-06	1.04	*AMFR*
16q23.2	rs13329835	16	80650805	A/G	0.93	(0.88-0.99)	0.017	0.991	0.92	(0.91-0.94)	2.9×10-28	9.6E-25	0.92	*CDYL2*
16q24.2	rs4496150	16	87085237	A/C	0.96	(0.94-0.99)	0.007	0.980	0.96	(0.95-0.97)	1.9×10-10	1.3E-06	0.96	
17q11.2	rs146699004	17	29230520	G/GGT	0.96	(0.91-1.01)	0.095	0.998	0.96	(0.95-0.97)	5.6×10-10	3.8E-06	0.96	*ATAD5*
17q22	rs2787486	17	53209774	A/C	1.08	(1.05-1.12)	4.7×10^{-7}	3.2E-03	1.08	(1.07-1.09)	2.1×10-34	7.0E-31	1.08	

17q25.3	rs745570	17	77781725	A/G	1.03	(1.00-1.06)	0.026	0.994	1.04	(1.03-1.05)	3.3×10-11	2.1E-07	1.04	
18q11.2	rs527616	18	24337424	C/G	0.97	(0.94-1.00)	0.022	0.993	0.95	(0.94-0.96)	7.7×10-16	5.5E-12	0.95	
18q11.2	rs1436904	18	24570667	T/G	1.03	(1.01-1.06)	0.015	0.990	1.05	(1.04-1.06)	7.8×10-16	5.2E-12	1.05	*CHST9*
18q12.1	rs36194942	18	25401204	A/AT	1.01	(0.98-1.04)	0.468	1.000	1.03	(1.02-1.04)	2.7×10-06	0.017	1.03	*CDH2*
18q12.1	rs117618124	18	29977689	T/C	0.93	(0.69-1.24)	0.604	1.000	1.11	(1.08-1.15)	1.1×10-11	7.3E-08	1.11	*GAREM1*
18q12.3	rs6507583	18	42399590	A/G	1.06	(1.02-1.10)	0.006	0.974	1.08	(1.06-1.11)	9.7×10-14	6.6E-10	1.09	*SETBP1*
19p13.2	rs322144	19	11423703	C/G	1.01	(0.98-1.05)	0.488	1.000	1.02	(1.01-1.04)	2.1×10-04	5.8E-01	1.03	*TSPAN16*
19p13.12	rs2594714	19	13954571	A/G	0.96	(0.93-0.99)	0.009	0.984	0.96	(0.95-0.97)	3.8×10-10	2.5E-06	0.96	
19p13.11	rs4808801	19	18571141	A/G	1.06	(1.03-1.09)	1.5×10^{-4}	0.495	1.07	(1.06-1.08)	2.3×10-31	7.7E-28	1.07	*ELL*
19p13.11	rs2965183	19	19545696	A/G	1.02	(0.99-1.05)	0.180	0.999	1.04	(1.03-1.05)	9.8×10-12	6.7E-08	1.05	*GATAD2A, MIR640*
19q12	rs113701136	19	30277729	T/C	1.02	(0.99-1.06)	0.178	0.999	1.02	(1.01-1.03)	5.6×10-04	0.785	1.02	*CCNE1*
19q13.31	rs3760982	19	44286513	A/G	0.99	(0.96-1.03)	0.746	1.000	1.05	(1.03-1.06)	1.6×10-14	1.1E-10	1.05	*KCCN4, LYPD5*
19q13.22	rs71338792	19	46183031	A/AT	0.96	(0.92-0.99)	0.022	0.993	0.95	(0.94-0.97)	2.7×10-10	1.8E-06	0.95	*GIPR*
20q11.22	rs2284378	20	32588095	T/C	0.99	(0.95-1.02)	0.392	1.000	1.01	(1.00-1.02)	8.8×10-02	0.998	1.01	*RALY*
20q13.13	rs6122906	20	48945911	A/G	0.97	(0.94-1.00)	0.029	0.995	0.95	(0.94-0.97)	3.7×10-11	2.4E-07	0.95	
21q21.1	rs2823093	21	16520832	A/G	0.94	(0.87-1.00)	0.056	0.997	0.94	(0.92-0.95)	1.7×10-21	5.6E-18	0.94	*NRIP1*
22q13.1	rs738321	22	38568833	C/G	1.03	(1.00-1.06)	0.087	0.998	1.05	(1.03-1.06)	7.2×10-14	4.8E-10	1.05	*PLA2G6*
22q13.1	rs6001930	22	40876234	T/C	0.95	(0.92-0.97)	2.0×10^{-4}	0.580	0.90	(0.89-0.92)	5.4×10-34	1.8E-30	0.89	*MKL1*
22q13.31	rs28512361	22	46283297	A/G	1.04	(0.95-1.14)	0.351	1.000	1.06	(1.04-1.08)	1.4×10-08	9.4E-05	1.06	

[a]OR literature: The ORs for risk variants identified in Asian populations were extracted from Zheng W et al. Nat Gen 2009, Cai Q et al. Hum Mol Genet. 2011, Long J et al. PLoS Genet. 2012, Cai Q et al. Nat Genet. 2014, and Han MR et al. Hum Mol Genet. 2016

; those idenfied in European populations were extracted from Michailidou K et al. Nature. 2017

[b]FPRP: The false positive report probability
