# Supplementary material for: Identification of novel breast cancer susceptibility loci in meta-analyses conducted among Asian and European descendants
Source: Nat Commun. 2020 Mar 5;11:1217. doi: 10.1038/s41467-020-15046-w (PMC7057957; doi:10.1038/s41467-020-15046-w)
Supplement: Supplementary file 9 — Supplementary Data 6 [file 41467_2020_15046_MOESM9_ESM.pdf]

Supplementary Data 6. Independent secondary associations found within previously known loci: A conditional analysis

SNP	Adjusted SNP	Loci	Chr	BP	EA	OA	EAF $_{Asian}$	OR (95%CI) $_{Asian}$	P $_{Asian}$	P $_{Asian+EA}$	Direction	Note
rs490037	rs616488	1p36.22	1	10485235	A	G	0.1599	0.92 (0.89-0.96)	5.43E-05	5.35E-04	--++--+---	Secondary signals
rs490960	rs616488	1p36.22	1	10485342	A	G	0.1613	0.92 (0.89-0.96)	3.20E-05	3.60E-04	--++--+---	Secondary signals
rs515423	rs616488	1p36.22	1	10485669	A	G	0.1599	0.92 (0.88-0.95)	1.75E-05	1.61E-04	--++--+---	Secondary signals
rs661256	rs616488	1p36.22	1	10490539	C	G	0.1606	0.92 (0.88-0.95)	1.97E-05	2.98E-04	--++------	Secondary signals
rs661272	rs616488	1p36.22	1	10490550	C	G	0.1612	0.92 (0.88-0.95)	1.80E-05	2.79E-04	--++------	Secondary signals
rs484416	rs616488	1p36.22	1	10491209	A	G	0.1625	0.92 (0.88-0.96)	2.12E-05	2.08E-04	--++--+---	Secondary signals
rs2847336	rs616488	1p36.22	1	10495110	T	C	0.1633	0.92 (0.89-0.96)	2.54E-05	2.31E-04	--++------	Secondary signals
rs676995	rs616488	1p36.22	1	10498453	T	C	0.1636	0.92 (0.89-0.96)	2.09E-05	1.67E-04	--++------	Secondary signals
rs589400	rs616488	1p36.22	1	10561282	G	A	0.8237	1.09 (1.04-1.13)	3.31E-05	4.54E-03	-+--++-+++	Secondary signals
rs2480784	rs616488	1p36.22	1	10570721	A	G	0.1517	0.91 (0.87-0.95)	6.65E-06	2.86E-07	----------	Secondary signals
rs1411402	rs616488	1p36.22	1	10579545	T	G	0.1812	0.91 (0.88-0.94)	9.19E-07	3.10E-03	----------	Better signals
rs2483677	rs616488	1p36.22	1	10580891	T	C	0.1812	0.91 (0.88-0.95)	1.19E-06	3.02E-03	----------	Better signals
rs2506885	rs616488	1p36.22	1	10581051	T	A	0.757	1.09 (1.05-1.13)	5.92E-06	2.06E-05	+-++++++++	Secondary signals
rs2056417	rs616488	1p36.22	1	10581658	A	G	0.1812	0.91 (0.87-0.94)	6.27E-07	6.02E-03	----------	Better signals
rs2480782	rs616488	1p36.22	1	10593296	T	G	0.152	0.91 (0.88-0.95)	8.67E-06	6.30E-07	----------	Secondary signals
rs2026792	rs616488	1p36.22	1	10594790	G	A	0.8182	1.10 (1.06-1.14)	1.78E-06	3.52E-04	++++++++++	Better signals
rs2506889	rs616488	1p36.22	1	10596022	T	C	0.1816	0.91 (0.88-0.95)	1.27E-06	5.95E-04	----------	Better signals
rs12375	rs616488	1p36.22	1	10596341	T	C	0.1818	0.91 (0.88-0.95)	1.39E-06	6.67E-04	----------	Better signals
rs2480777	rs616488	1p36.22	1	10597551	T	C	0.1818	0.91 (0.88-0.95)	1.57E-06	7.49E-04	----------	Better signals
rs2506892	rs616488	1p36.22	1	10598216	A	G	0.1815	0.91 (0.88-0.95)	1.46E-06	6.70E-04	----------	Better signals
rs2150038	rs616488	1p36.22	1	10609973	T	G	0.1814	0.91 (0.88-0.95)	1.34E-06	8.33E-04	+---------	Better signals
rs9430149	rs616488	1p36.22	1	10612820	C	G	0.1812	0.91 (0.88-0.94)	8.94E-07	4.96E-04	+---------	Better signals
rs67488812	rs616488	1p36.22	1	10614804	G	A	0.8186	1.10 (1.06-1.14)	1.08E-06	5.48E-04	++++++++++	Better signals
rs17035305	rs616488	1p36.22	1	10616505	A	C	0.1815	0.91 (0.88-0.95)	2.30E-06	6.09E-04	----------	Better signals
rs12028449	rs616488	1p36.22	1	10617034	A	G	0.1811	0.91 (0.88-0.95)	1.24E-06	6.66E-04	+---------	Better signals
rs11121587	rs616488	1p36.22	1	10618109	G	A	0.8184	1.10 (1.06-1.14)	1.01E-06	4.92E-04	-+++++++++	Better signals
rs4846235	rs616488	1p36.22	1	10632235	T	C	0.1812	0.91 (0.88-0.94)	9.15E-07	6.87E-04	+---------	Better signals
rs7567444	rs71801447	2q13	2	111863438	T	C	0.5116	1.07 (1.03-1.1)	9.06E-05	2.79E-01	+-++++???-	Better signals
rs2015454	rs71801447	2q13	2	111872148	A	G	0.5132	1.07 (1.04-1.11)	4.18E-05	2.54E-01	+-++++???-	Better signals
rs4848393	rs71801447	2q13	2	111877297	T	C	0.513	1.07 (1.03-1.1)	6.84E-05	2.21E-01	+-++++???-	Better signals
rs2241845	rs71801447	2q13	2	111879100	A	G	0.5133	1.07 (1.04-1.1)	5.29E-05	2.38E-01	+-++++???-	Better signals
rs6758181	rs71801447	2q13	2	111886914	C	T	0.4873	0.94 (0.91-0.97)	9.61E-05	2.16E-01	-+----???+	Better signals
rs4849417	rs71801447	2q13	2	111887703	T	A	0.4867	0.94 (0.91-0.97)	7.85E-05	2.61E-01	-+----???+	Better signals
rs6746608	rs71801447	2q13	2	111892984	A	G	0.5137	1.07 (1.03-1.1)	9.32E-05	2.34E-01	+-++++???-	Better signals
rs6750599	rs71801447	2q13	2	111893869	A	T	0.5138	1.07 (1.03-1.1)	6.14E-05	2.02E-01	+-++++???-	Better signals
rs13396983	rs71801447	2q13	2	111900598	A	G	0.5136	1.07 (1.03-1.1)	9.51E-05	2.01E-01	+-++++???-	Better signals
rs1980045	rs71801447	2q13	2	111904541	A	G	0.5127	1.07 (1.03-1.1)	5.88E-05	5.88E-05	+-++++????	Better signals
rs36018702	rs71801447	2q13	2	111928373	A	C	0.5136	1.07 (1.03-1.1)	9.93E-05	2.07E-01	+-++++???-	Better signals
rs78296102	rs4849887	2q14.1	2	121206308	T	C	0.9592	0.8 (0.74-0.87)	4.02E-07	4.02E-07	---------?	Better signals
rs10624353	rs34207738	3q23	3	141074870	ACT	A	0.6594	0.94 (0.91-0.97)	8.42E-05	1.44E-02	--------?-	Better signals
rs1863868	rs34207738	3q23	3	141087623	C	T	0.6546	0.94 (0.91-0.96)	1.62E-05	9.10E-03	--------?-	Better signals
rs7624084	rs34207738	3q23	3	141093285	C	T	0.6457	0.93 (0.9-0.96)	5.00E-06	1.35E-01	--------?-	Better signals
rs6440003	rs34207738	3q23	3	141094209	A	G	0.3479	1.07 (1.04-1.11)	5.01E-06	4.43E-01	++++++++?+	Better signals
rs4683605	rs34207738	3q23	3	141094769	A	C	0.3348	1.06 (1.03-1.1)	8.67E-05	6.65E-01	++++++++?-	Better signals
rs6764769	rs34207738	3q23	3	141100280	G	A	0.6522	0.93 (0.9-0.96)	6.54E-06	3.18E-01	--------?-	Better signals
rs6763931	rs34207738	3q23	3	141102833	A	G	0.3471	1.07 (1.04-1.1)	7.47E-06	4.45E-01	++++++++?+	Better signals

rs724016	rs34207738	3q23	3	141105570	G	A	0.6582	0.93 (0.91-0.96)	1.02E-05	1.02E-05	--------??	Better signals
rs7632381	rs34207738	3q23	3	141106063	C	T	0.6546	0.94 (0.91-0.97)	2.58E-05	2.58E-05	--------??	Better signals
rs4683606	rs34207738	3q23	3	141110074	G	A	0.6548	0.94 (0.91-0.96)	1.86E-05	1.86E-05	--------??	Better signals
rs34466132	rs34207738	3q23	3	141117969	CT	C	0.3258	1.07 (1.04-1.1)	2.68E-05	3.16E-01	++++++++?+	Better signals
rs13068733	rs34207738	3q23	3	141118028	G	A	0.6545	0.94 (0.91-0.97)	2.19E-05	2.19E-05	--------??	Better signals
rs2871960	rs34207738	3q23	3	141121814	C	A	0.6542	0.94 (0.91-0.97)	3.46E-05	3.46E-05	--------??	Better signals
rs1344674	rs34207738	3q23	3	141125186	G	A	0.6538	0.94 (0.91-0.97)	2.66E-05	3.78E-01	--------?-	Better signals
rs1344672	rs34207738	3q23	3	141125705	G	C	0.6543	0.94 (0.91-0.97)	2.59E-05	4.01E-01	--------?-	Better signals
rs6763927	rs34207738	3q23	3	141140366	T	A	0.6534	0.94 (0.91-0.97)	3.88E-05	2.88E-01	--------?-	Better signals
rs6440006	rs34207738	3q23	3	141142691	A	G	0.3488	1.07 (1.04-1.1)	2.72E-05	6.69E-02	++++++++?+	Better signals
rs7650602	rs34207738	3q23	3	141147414	C	T	0.6539	0.94 (0.91-0.97)	2.60E-05	6.05E-02	--------?-	Better signals
rs7625643	rs34207738	3q23	3	141150026	G	A	0.6536	0.94 (0.91-0.96)	1.71E-05	7.66E-02	--------?-	Better signals
rs6440008	rs34207738	3q23	3	141154542	C	T	0.6956	0.93 (0.9-0.96)	7.57E-06	2.38E-01	--------?-	Better signals
rs9878347	rs34207738	3q23	3	141169982	C	A	0.6531	0.93 (0.9-0.96)	5.00E-07	2.10E-04	--------?-	Better signals
rs9879503	rs34207738	3q23	3	141170661	G	A	0.654	0.93 (0.9-0.96)	4.98E-07	1.84E-04	--------?-	Better signals
rs1863867	rs34207738	3q23	3	141175620	C	A	0.6589	0.93 (0.91-0.96)	2.79E-06	2.25E-04	--------?-	Better signals
rs2312193	rs34207738	3q23	3	141188399	C	T	0.7319	0.92 (0.9-0.95)	7.50E-07	3.84E-03	--------?-	Better signals
rs3806646	rs34207738	3q23	3	141204844	C	G	0.314	1.08 (1.05-1.11)	2.49E-07	5.30E-05	++++++++?+	Better signals
rs3821712	rs34207738	3q23	3	141205185	T	C	0.3388	1.08 (1.05-1.11)	4.10E-07	5.36E-05	++++++++?+	Better signals
rs11917587	rs34207738	3q23	3	141207575	A	G	0.3392	1.08 (1.04-1.11)	9.51E-07	3.14E-04	++++++++?+	Better signals
rs7643837	rs34207738	3q23	3	141209867	C	T	0.6605	0.93 (0.9-0.96)	7.11E-07	5.67E-05	--------?-	Better signals
rs9813177	rs34207738	3q23	3	141212518	G	A	0.6635	0.93 (0.9-0.96)	8.57E-07	6.44E-05	--------?-	Better signals
rs6795168	rs34207738	3q23	3	141213581	A	T	0.3364	1.08 (1.04-1.11)	9.02E-07	7.18E-05	++++++++?+	Better signals
rs6808837	rs34207738	3q23	3	141217954	C	T	0.6618	0.93 (0.9-0.96)	6.21E-07	4.43E-05	--------?-	Better signals
rs6763498	rs34207738	3q23	3	141218568	A	T	0.3357	1.08 (1.04-1.11)	8.77E-07	6.17E-05	++++++++?+	Better signals
rs6767158	rs34207738	3q23	3	141228874	T	C	0.3356	1.08 (1.04-1.11)	1.01E-06	5.83E-05	++++++++?+	Better signals
rs13069717	rs34207738	3q23	3	141234523	C	T	0.6616	0.93 (0.9-0.96)	8.39E-07	4.83E-05	--------?-	Better signals
rs35711802	rs34207738	3q23	3	141236524	C	G	0.3386	1.08 (1.05-1.11)	7.63E-07	4.95E-05	++++++++?+	Better signals
rs6785874	rs34207738	3q23	3	141244816	A	G	0.3344	1.08 (1.04-1.11)	8.92E-07	6.12E-05	++++++++?+	Better signals
rs6800122	rs34207738	3q23	3	141249398	T	C	0.3366	1.08 (1.04-1.11)	8.32E-07	5.05E-05	++++++++?+	Better signals
rs9835593	rs34207738	3q23	3	141263990	A	G	0.338	1.08 (1.05-1.11)	6.80E-07	5.16E-05	++++++++?+	Better signals
rs6776003	rs34207738	3q23	3	141266493	A	G	0.3385	1.08 (1.05-1.11)	8.41E-07	2.85E-04	++++++++?+	Better signals
rs11711375	rs34207738	3q23	3	141287539	G	A	0.6612	0.93 (0.9-0.96)	4.87E-07	5.50E-05	--------?-	Better signals
rs1493209	rs34207738	3q23	3	141292618	A	G	0.3358	1.08 (1.05-1.11)	6.08E-07	6.55E-05	++++++++?+	Better signals
rs3821710	rs34207738	3q23	3	141301451	A	G	0.3354	1.08 (1.05-1.11)	4.59E-07	4.81E-05	++++++++?+	Better signals
rs6440013	rs34207738	3q23	3	141304571	G	A	0.6621	0.93 (0.9-0.96)	5.58E-07	5.48E-05	--------?-	Better signals
rs11916906	rs34207738	3q23	3	141314133	T	A	0.661	0.93 (0.9-0.96)	5.22E-07	6.72E-05	--------?-	Better signals
3:141320284:CAAAAAAAAA	rs34207738	3q23	3	141320284	CA	CAAAAAAAAAGAA	0.3107	1.08 (1.05-1.11)	1.87E-06	1.87E-06	+++++++???	Better signals
rs295320	rs34207738	3q23	3	141324556	G	A	0.6602	0.93 (0.9-0.95)	2.32E-07	4.78E-05	--------?-	Better signals
rs295322	rs34207738	3q23	3	141326602	C	T	0.738	0.92 (0.9-0.95)	3.69E-07	5.91E-07	--------?-	Better signals
rs295323	rs34207738	3q23	3	141327474	A	G	0.3391	1.08 (1.05-1.11)	4.21E-07	4.79E-05	++++++++?+	Better signals
rs2172471	rs34207738	3q23	3	141331222	A	G	0.3384	1.08 (1.05-1.11)	3.93E-07	4.75E-05	++++++++?+	Better signals
rs6440015	rs34207738	3q23	3	141336351	T	G	0.3381	1.08 (1.05-1.11)	4.67E-07	1.45E-04	++++++++?+	Better signals
rs2639998	rs34207738	3q23	3	141336708	T	A	0.6609	0.93 (0.9-0.96)	5.99E-07	1.63E-04	--------?-	Better signals
rs10589233	rs34207738	3q23	3	141336832	A	AAG	0.3131	1.08 (1.05-1.12)	2.58E-07	6.06E-05	++++++++?+	Better signals
rs55813385	rs34207738	3q23	3	141336835	T	A	0.6867	0.92 (0.9-0.95)	2.52E-07	1.37E-04	--------?-	Better signals
rs11746538	rs116095464	5p15.33	5	427466	A	C	0.4931	0.93 (0.9-0.96)	1.05E-05	7.38E-01	---?--?-?+	Better signals
rs349577	rs116095464	5p15.33	5	445726	G	C	0.5062	1.07 (1.04-1.11)	9.35E-06	6.63E-01	+++?++?+?-	Better signals
rs74626386	rs62355902	5q11.2	5	55990052	T	G	0.1183	1.12 (1.07-1.17)	5.56E-07	2.24E-06	+-++++?+++	Better signals
rs79299334	rs62355902	5q11.2	5	55991699	A	G	0.1343	1.13 (1.08-1.17)	3.28E-08	9.44E-09	++++++?+++	Better signals

rs77367410	rs62355902	5q11.2	5	55992290	G	A	0.8596	0.89 (0.85-0.93)	3.25E-08	5.03E-09	------?---	Better signals
rs6877066	rs62355902	5q11.2	5	55993813	C	T	0.8639	0.89 (0.85-0.93)	4.58E-08	2.18E-08	------?---	Better signals
rs16886113	rs62355902	5q11.2	5	55995035	G	T	0.8564	0.89 (0.86-0.93)	3.91E-08	6.21E-09	------?---	Better signals
rs74773564	rs62355902	5q11.2	5	55995869	T	C	0.1297	1.13 (1.08-1.18)	3.40E-08	7.97E-09	++++++?+++	Better signals
rs73756771	rs62355902	5q11.2	5	56001002	G	C	0.8852	0.85 (0.81-0.89)	8.11E-11	1.69E-12	------?-?-	Better signals
rs75433101	rs62355902	5q11.2	5	56002070	A	T	0.1148	1.17 (1.12-1.23)	9.08E-11	2.86E-13	++++++?+?+	Better signals
rs73756772	rs62355902	5q11.2	5	56002136	C	G	0.1149	1.17 (1.12-1.23)	8.91E-11	1.21E-13	++++++?+?+	Better signals
rs73756773	rs62355902	5q11.2	5	56002851	A	T	0.1149	1.17 (1.12-1.23)	8.49E-11	1.34E-13	++++++?+?+	Better signals
rs55878836	rs62355902	5q11.2	5	56004565	G	C	0.8851	0.85 (0.81-0.89)	8.79E-11	1.36E-13	------?-?-	Better signals
rs113465266	rs62355902	5q11.2	5	56004872	C	CA	0.8852	0.85 (0.81-0.9)	9.87E-11	3.90E-13	------?-?-	Better signals
rs57841600	rs62355902	5q11.2	5	56005065	G	A	0.8851	0.85 (0.81-0.9)	8.85E-11	1.36E-13	------?-?-	Better signals
rs60588964	rs62355902	5q11.2	5	56006008	C	A	0.8852	0.85 (0.81-0.9)	9.02E-11	1.37E-13	------?-?-	Better signals
rs115302578	rs62355902	5q11.2	5	56006345	A	G	0.1191	1.17 (1.12-1.23)	8.35E-11	1.18E-13	+-++++?+?+	Better signals
rs111944656	rs62355902	5q11.2	5	56008230	T	G	0.1148	1.17 (1.12-1.23)	1.12E-10	2.99E-13	++++++?+?+	Better signals
rs112191071	rs62355902	5q11.2	5	56008241	A	G	0.1148	1.17 (1.12-1.23)	1.12E-10	2.81E-13	++++++?+?+	Better signals
rs6883200	rs62355902	5q11.2	5	56010025	G	C	0.8852	0.85 (0.81-0.9)	1.48E-10	3.58E-13	------?-?-	Better signals
rs68115105	rs62355902	5q11.2	5	56011234	T	G	0.4537	1.07 (1.04-1.1)	8.46E-06	2.50E-11	+-++++?+++	Better signals
rs73756777	rs62355902	5q11.2	5	56011236	T	G	0.1395	1.15 (1.1-1.2)	3.14E-09	3.14E-09	++++++?+??	Better signals
rs113803454	rs62355902	5q11.2	5	56012282	T	TC	0.1159	1.17 (1.12-1.23)	7.43E-11	3.65E-13	++++++?+?+	Better signals
rs111872579	rs62355902	5q11.2	5	56012629	TA	T	0.8847	0.85 (0.81-0.9)	1.45E-10	6.99E-13	------?-?-	Better signals
rs73756780	rs62355902	5q11.2	5	56013798	A	T	0.1572	1.13 (1.08-1.18)	4.59E-08	1.46E-12	+-++++?+?+	Better signals
rs189669878	rs62355902	5q11.2	5	56013985	A	T	0.9618	0.78 (0.71-0.85)	6.71E-09	6.71E-09	------?-??	Better signals
rs200520587	rs62355902	5q11.2	5	56020509	GT	GTT	0.8836	0.86 (0.82-0.91)	2.43E-08	2.43E-08	------????	Better signals
rs112476198	rs62355902	5q11.2	5	56020572	C	T	0.8853	0.85 (0.81-0.89)	5.60E-11	2.68E-14	------?-?-	Better signals
rs79488097	rs62355902	5q11.2	5	56022251	C	T	0.8854	0.85 (0.81-0.89)	5.70E-11	2.71E-14	------?-?-	Better signals
rs16886164	rs62355902	5q11.2	5	56022907	A	G	0.1142	1.18 (1.12-1.24)	3.83E-11	4.29E-14	++++++?+?+	Better signals
rs76482628	rs62355902	5q11.2	5	56025682	A	G	0.1088	1.2 (1.14-1.26)	2.04E-13	1.14E-14	+-++++?+?+	Better signals
rs59957907	rs62355902	5q11.2	5	56027240	G	A	0.6106	0.94 (0.92-0.97)	3.00E-05	3.00E-05	-----+++-?	Better signals
rs138805815	rs62355902	5q11.2	5	56027591	T	C	0.1101	1.21 (1.15-1.27)	7.23E-14	7.05E-15	+-++++?+?+	Better signals
rs11285405	rs62355902	5q11.2	5	56037627	CA	C	0.6842	0.93 (0.9-0.96)	2.26E-06	2.13E-01	------?-?+	Better signals
rs77961606	rs62355902	5q11.2	5	56040643	C	T	0.8891	0.83 (0.79-0.87)	6.01E-14	1.85E-15	-+----?-?-	Better signals
rs76250845	rs62355902	5q11.2	5	56042972	T	C	0.1097	1.21 (1.15-1.27)	4.76E-14	2.23E-15	+-++++?+?+	Better signals
rs16886218	rs62355902	5q11.2	5	56044486	T	G	0.1098	1.21 (1.15-1.27)	5.51E-14	1.83E-15	+-++++?+?+	Better signals
rs78591923	rs62355902	5q11.2	5	56044507	A	G	0.1097	1.21 (1.15-1.27)	5.05E-14	3.53E-15	+-++++?+?+	Better signals
rs16886220	rs62355902	5q11.2	5	56044508	A	T	0.1098	1.21 (1.15-1.27)	5.52E-14	1.83E-15	+-++++?+?+	Better signals
rs73131777	rs62355902	5q11.2	5	56044903	T	C	0.1097	1.21 (1.15-1.27)	5.81E-14	3.35E-15	+-++++?+?+	Better signals
rs113417697	rs62355902	5q11.2	5	56045795	G	GA	0.1122	1.21 (1.15-1.27)	5.08E-14	1.16E-12	++++++?+?+	Better signals
rs73131786	rs62355902	5q11.2	5	56046256	C	A	0.8904	0.83 (0.79-0.87)	5.01E-14	2.91E-15	-+----?-?-	Better signals
rs56793118	rs62355902	5q11.2	5	56046399	C	T	0.8904	0.83 (0.79-0.87)	5.06E-14	3.45E-15	-+----?-?-	Better signals
rs79957360	rs62355902	5q11.2	5	56046461	T	C	0.1096	1.21 (1.15-1.27)	5.07E-14	2.93E-15	+-++++?+?+	Better signals
rs80146798	rs62355902	5q11.2	5	56046651	T	C	0.1096	1.21 (1.15-1.27)	5.09E-14	2.75E-15	+-++++?+?+	Better signals
rs73131789	rs62355902	5q11.2	5	56047143	A	G	0.1094	1.21 (1.15-1.27)	7.55E-14	3.59E-15	+-++++?+?+	Better signals
rs80152369	rs62355902	5q11.2	5	56047342	A	C	0.1094	1.21 (1.15-1.27)	7.83E-14	4.15E-15	+-++++?+?+	Better signals
rs35605179	rs62355902	5q11.2	5	56048008	GA	G	0.8773	0.84 (0.8-0.88)	1.71E-12	1.98E-13	-+----?-?-	Better signals
rs75499227	rs62355902	5q11.2	5	56048635	G	A	0.8903	0.83 (0.79-0.87)	5.83E-14	3.70E-15	-+----?-?-	Better signals
rs73131801	rs62355902	5q11.2	5	56051006	A	G	0.1093	1.21 (1.15-1.27)	6.47E-14	4.07E-15	++++++?+?+	Better signals
rs79160707	rs62355902	5q11.2	5	56052938	T	C	0.1095	1.21 (1.15-1.27)	5.25E-14	5.06E-15	++++++?+?+	Better signals
rs112776581	rs62355902	5q11.2	5	56054333	T	TA	0.1098	1.21 (1.15-1.27)	3.46E-14	4.75E-15	++++++?+?+	Better signals
rs76485124	rs62355902	5q11.2	5	56060400	T	C	0.0985	1.21 (1.15-1.27)	1.12E-12	6.11E-12	++++++?+?+	Better signals
rs112497245	rs62355902	5q11.2	5	56060954	A	G	0.1636	1.16 (1.12-1.21)	5.35E-14	9.89E-19	++++++?+?+	Better signals
rs78209889	rs62355902	5q11.2	5	56065572	C	T	0.8365	0.86 (0.83-0.89)	5.41E-14	4.03E-18	------?-?-	Better signals

rs16886272	rs62355902	5q11.2	5	56067434	A	G	0.1635	1.16 (1.12-1.21)	1.49E-13	1.87E-18	++++++?+?+	Better signals
rs4358485	rs62355902	5q11.2	5	56074451	G	A	0.2442	1.08 (1.04-1.11)	3.93E-06	2.04E-01	++++++++--	Better signals
rs6450406	rs62355902	5q11.2	5	56076625	A	G	0.7564	0.93 (0.9-0.96)	4.04E-06	2.01E-01	--------++	Better signals
rs10461616	rs62355902	5q11.2	5	56080347	T	C	0.7616	0.93 (0.9-0.96)	4.24E-06	2.26E-01	------+-++	Better signals
rs10055224	rs62355902	5q11.2	5	56081201	T	C	0.762	0.93 (0.9-0.96)	2.73E-06	2.39E-01	------+-++	Better signals
rs10940512	rs62355902	5q11.2	5	56082094	G	C	0.244	1.08 (1.04-1.11)	2.98E-06	2.28E-01	++++++++--	Better signals
rs10471991	rs62355902	5q11.2	5	56085632	T	C	0.7562	0.93 (0.9-0.96)	2.69E-06	2.36E-01	--------++	Better signals
rs10549600	rs62355902	5q11.2	5	56087731	GTA	G	0.2328	1.09 (1.05-1.12)	5.54E-07	2.05E-01	++++++-+?-	Better signals
rs113317823	rs62355902	5q11.2	5	56087883	T	C	0.136	1.16 (1.12-1.21)	1.04E-12	1.71E-13	++++++?+?+	Better signals
rs144983009	rs62355902	5q11.2	5	56087899	T	C	0.157	1.15 (1.1-1.2)	8.84E-12	2.69E-13	++++++?+-+	Better signals
rs9292122	rs62355902	5q11.2	5	56087910	G	A	0.2536	1.08 (1.05-1.12)	8.62E-07	1.19E-01	++++++-+--	Better signals
rs3309	rs62355902	5q11.2	5	56092779	T	A	0.2657	1.08 (1.05-1.11)	1.75E-06	2.95E-01	++++++++--	Better signals
rs6888317	rs62355902	5q11.2	5	56093307	G	A	0.273	1.08 (1.04-1.11)	2.34E-06	2.77E-01	++++++++--	Better signals
rs6870175	rs62355902	5q11.2	5	56093371	A	G	0.7269	0.93 (0.9-0.96)	2.26E-06	2.85E-01	--------++	Better signals
rs4146565	rs62355902	5q11.2	5	56093825	T	C	0.7283	0.93 (0.9-0.96)	2.06E-06	2.86E-01	--------++	Better signals
rs2408597	rs62355902	5q11.2	5	56098632	G	A	0.2571	1.08 (1.05-1.11)	1.29E-06	2.80E-01	++++++-+--	Better signals
rs6893754	rs62355902	5q11.2	5	56100006	G	A	0.2573	1.08 (1.05-1.11)	1.40E-06	2.84E-01	++++++-+--	Better signals
rs6862118	rs62355902	5q11.2	5	56106474	G	A	0.249	1.08 (1.04-1.11)	3.56E-06	2.31E-01	++++++++--	Better signals
5:56109436:TACAA:AACAA	rs62355902	5q11.2	5	56109436	AACAA	TACAA	0.1274	1.15 (1.1-1.21)	8.57E-10	8.57E-10	++++++????	Better signals
5:56109723:T:<INS:ME:ALU	rs62355902	5q11.2	5	56109723	NS:ME:AL	T	0.0911	1.16 (1.09-1.25)	1.06E-05	3.07E-08	++++?+???+	Better signals
rs7731700	rs62355902	5q11.2	5	56111927	C	T	0.2392	1.08 (1.04-1.11)	4.29E-06	2.25E-01	++++++++--	Better signals
rs2591951	rs62355902	5q11.2	5	56120770	T	A	0.4563	0.94 (0.92-0.97)	7.93E-05	4.01E-11	-+-+----+-	Better signals
rs16886364	rs62355902	5q11.2	5	56122344	G	A	0.8676	0.86 (0.83-0.9)	1.57E-12	7.42E-16	------?-+-	Better signals
rs79953845	rs62355902	5q11.2	5	56124794	C	G	0.1332	1.16 (1.11-1.21)	1.85E-12	6.08E-16	++++++?+-+	Better signals
rs111968853	rs62355902	5q11.2	5	56128939	T	C	0.1332	1.16 (1.11-1.21)	3.02E-12	9.23E-16	++++++?+-+	Better signals
rs76418981	rs62355902	5q11.2	5	56130534	G	T	0.8668	0.86 (0.83-0.9)	3.58E-12	1.07E-15	------?-+-	Better signals
rs16886397	rs62355902	5q11.2	5	56134276	G	A	0.8668	0.86 (0.83-0.9)	3.09E-12	1.38E-15	------?-+-	Better signals
rs77371588	rs62355902	5q11.2	5	56134560	G	T	0.9291	0.81 (0.77-0.86)	3.11E-12	1.13E-13	------?-?-	Better signals
rs60590641	rs62355902	5q11.2	5	56141155	G	A	0.929	0.81 (0.77-0.86)	2.38E-12	1.20E-13	------?-?-	Better signals
rs16886420	rs62355902	5q11.2	5	56142999	T	C	0.1333	1.16 (1.11-1.21)	2.36E-12	1.12E-15	++++++?+-+	Better signals
rs60200883	rs62355902	5q11.2	5	56143217	C	A	0.8667	0.86 (0.83-0.9)	2.30E-12	8.56E-16	------?-+-	Better signals
rs199942689	rs62355902	5q11.2	5	56143929	CA	C	0.2387	1.09 (1.06-1.13)	1.75E-07	1.75E-07	++++++-+??	Better signals
rs41106	rs62355902	5q11.2	5	56145057	G	T	0.2695	1.08 (1.05-1.11)	6.07E-07	3.83E-01	+++++++++-	Better signals
rs33329	rs62355902	5q11.2	5	56145068	T	C	0.7345	0.92 (0.89-0.95)	2.00E-07	2.00E-07	---------?	Better signals
rs832567	rs62355902	5q11.2	5	56152416	C	A	0.2631	1.08 (1.05-1.12)	5.56E-07	3.75E-01	+++++++++-	Better signals
rs78686102	rs62355902	5q11.2	5	56152795	A	G	0.0691	1.23 (1.16-1.3)	7.05E-12	8.31E-13	++++++?+?+	Better signals
rs1017226	rs62355902	5q11.2	5	56153392	C	T	0.8659	0.86 (0.82-0.89)	6.51E-13	3.60E-16	------?-?-	Better signals
rs150755620	rs62355902	5q11.2	5	56156683	A	AAAAAAAAG	0.1288	1.15 (1.1-1.2)	1.45E-09	2.00E-13	++++++???+	Better signals
rs832573	rs62355902	5q11.2	5	56159578	T	C	0.7593	0.92 (0.89-0.95)	3.48E-07	3.48E-01	------+-?+	Better signals
rs56822906	rs62355902	5q11.2	5	56165914	G	A	0.8639	0.86 (0.82-0.9)	1.30E-12	5.24E-16	------?-?-	Better signals
rs2229882	rs62355902	5q11.2	5	56168712	T	C	0.0695	1.22 (1.15-1.3)	1.30E-11	2.99E-13	++++++?+?+	Better signals
rs16886448	rs62355902	5q11.2	5	56170813	G	C	0.8677	0.86 (0.82-0.9)	1.54E-12	1.85E-15	------?-?-	Better signals
rs9686160	rs62355902	5q11.2	5	56172001	C	G	0.1327	1.16 (1.12-1.21)	1.43E-12	5.31E-16	++++++?+?+	Better signals
rs2548663	rs62355902	5q11.2	5	56172778	G	A	0.2375	1.09 (1.05-1.12)	3.85E-07	3.75E-01	++++++-++-	Better signals
rs9687226	rs62355902	5q11.2	5	56176446	T	C	0.1328	1.16 (1.12-1.21)	1.85E-12	2.37E-15	++++++?+?+	Better signals
rs702689	rs62355902	5q11.2	5	56177443	G	A	0.2332	1.08 (1.05-1.12)	5.39E-07	3.55E-01	+++++++++-	Better signals
rs3822625	rs62355902	5q11.2	5	56178111	G	A	0.8673	0.86 (0.82-0.9)	1.13E-12	2.66E-15	------?-?-	Better signals
rs16877026	rs62355902	5q11.2	5	56184926	C	G	0.1327	1.16 (1.12-1.21)	1.26E-12	6.07E-16	++++++?+?+	Better signals
rs76058858	rs62355902	5q11.2	5	56186439	A	G	0.1324	1.16 (1.12-1.21)	1.65E-12	8.02E-16	++++++?+?+	Better signals
rs58835146	rs62355902	5q11.2	5	56186986	C	A	0.2998	1.08 (1.05-1.12)	2.37E-06	8.27E-02	+++-++++?-	Better signals
rs376579643	rs62355902	5q11.2	5	56193454	C	CCTGT	0.8615	0.86 (0.83-0.9)	1.67E-12	1.71E-14	------?-?-	Better signals

rs77982563	rs62355902	5q11.2	5	56193667	T	C	0.1526	1.15 (1.11-1.2)	4.24E-12	1.38E-14	++++++?+?+	Better signals
rs12655019	rs62355902	5q11.2	5	56195790	G	A	0.8459	0.87 (0.84-0.9)	3.38E-12	1.28E-12	------?---	Better signals
rs252912	rs62355902	5q11.2	5	56195792	C	T	0.2648	1.08 (1.04-1.11)	1.52E-06	9.18E-01	++++++-+--	Better signals
rs12657353	rs62355902	5q11.2	5	56196276	A	G	0.1503	1.15 (1.11-1.2)	4.00E-12	5.65E-13	++++++?+++	Better signals
rs96844	rs62355902	5q11.2	5	56196604	G	A	0.2648	1.08 (1.04-1.11)	1.21E-06	9.04E-01	++++++-+--	Better signals
rs5868034	rs62355902	5q11.2	5	56198221	GA	G	0.442	0.94 (0.91-0.97)	2.10E-05	8.42E-12	-+-+------	Better signals
rs173764	rs62355902	5q11.2	5	56198920	C	T	0.446	0.94 (0.92-0.97)	2.61E-05	1.86E-11	-+-+------	Better signals
rs832539	rs62355902	5q11.2	5	56199386	T	G	0.5551	1.06 (1.03-1.09)	2.59E-05	1.54E-11	+-+-++++++	Better signals
rs76679228	rs62355902	5q11.2	5	56199511	T	C	0.1507	1.15 (1.11-1.2)	2.28E-12	2.19E-13	++++++?+++	Better signals
rs832538	rs62355902	5q11.2	5	56200016	C	T	0.4419	0.94 (0.92-0.97)	2.18E-05	1.14E-11	-+-+------	Better signals
rs702686	rs62355902	5q11.2	5	56201350	A	G	0.5545	1.06 (1.03-1.09)	2.39E-05	8.28E-12	+-+-++++++	Better signals
rs112793966	rs62355902	5q11.2	5	56204118	T	C	0.1541	1.15 (1.11-1.2)	2.50E-12	2.97E-13	++++++?+++	Better signals
rs10042998	rs62355902	5q11.2	5	56205299	G	A	0.8455	0.87 (0.84-0.9)	3.93E-12	6.55E-13	------?---	Better signals
rs252924	rs62355902	5q11.2	5	56205643	A	G	0.732	0.93 (0.9-0.96)	1.68E-06	9.23E-01	------+-++	Better signals
rs33320	rs62355902	5q11.2	5	56206529	C	T	0.2618	1.08 (1.04-1.11)	1.78E-06	9.29E-01	++++++-+--	Better signals
rs6450410	rs62355902	5q11.2	5	56206701	G	A	0.8499	0.87 (0.84-0.91)	5.55E-12	5.34E-14	------?---	Better signals
rs7731829	rs62355902	5q11.2	5	56207711	C	T	0.8457	0.87 (0.84-0.91)	5.08E-12	5.37E-14	------?---	Better signals
rs2271202	rs62355902	5q11.2	5	56208541	C	T	0.8469	0.87 (0.84-0.91)	5.46E-12	4.86E-14	------?---	Better signals
rs33317	rs62355902	5q11.2	5	56209275	G	A	0.267	1.07 (1.04-1.11)	2.23E-06	8.51E-01	++++++-+--	Better signals
rs75167082	rs62355902	5q11.2	5	56211404	C	T	0.8475	0.87 (0.84-0.9)	3.32E-12	3.63E-14	------?---	Better signals
rs1965681	rs62355902	5q11.2	5	56212186	G	A	0.8468	0.87 (0.84-0.91)	5.73E-12	4.64E-14	------?---	Better signals
rs3839259	rs62355902	5q11.2	5	56212892	C	CATG	0.8499	0.87 (0.84-0.91)	9.21E-12	8.87E-14	------?-?-	Better signals
rs1466010	rs62355902	5q11.2	5	56213156	G	A	0.8472	0.87 (0.84-0.91)	7.38E-12	5.64E-14	------?---	Better signals
rs1466007	rs62355902	5q11.2	5	56213639	C	A	0.8473	0.87 (0.84-0.91)	1.01E-11	7.16E-14	------?---	Better signals
rs35420907	rs62355902	5q11.2	5	56213941	TATC	T	0.85	0.88 (0.85-0.92)	1.04E-08	5.56E-12	------???-	Better signals
rs150002094	rs62355902	5q11.2	5	56214460	ACTT	A	0.8499	0.88 (0.85-0.92)	1.09E-08	5.91E-12	------???-	Better signals
rs12654125	rs62355902	5q11.2	5	56215753	A	G	0.1499	1.15 (1.1-1.19)	1.04E-11	1.03E-13	++++++?+++	Better signals
rs3756586	rs62355902	5q11.2	5	56217196	G	A	0.8468	0.87 (0.84-0.91)	7.58E-12	5.84E-14	------?---	Better signals
rs16886496	rs62355902	5q11.2	5	56217529	C	T	0.8472	0.87 (0.84-0.91)	1.03E-11	4.18E-14	------?---	Better signals
rs702681	rs62355902	5q11.2	5	56218029	C	T	0.2675	1.07 (1.04-1.11)	2.29E-06	8.60E-01	++++++-+--	Better signals
rs74865790	rs62355902	5q11.2	5	56219698	C	T	0.8472	0.87 (0.84-0.91)	1.04E-11	3.91E-14	------?---	Better signals
rs35795629	rs62355902	5q11.2	5	56220080	GT	G	0.846	0.88 (0.84-0.91)	4.62E-11	2.77E-15	---+--?-?-	Better signals
rs16886497	rs62355902	5q11.2	5	56220714	C	T	0.8469	0.87 (0.84-0.91)	1.08E-11	5.68E-14	------?-?-	Better signals
rs12657064	rs62355902	5q11.2	5	56220999	A	G	0.1531	1.15 (1.1-1.19)	1.23E-11	5.78E-14	++++++?+?+	Better signals
rs79354983	rs62355902	5q11.2	5	56221537	G	A	0.8469	0.87 (0.84-0.91)	1.17E-11	3.65E-14	------?-?-	Better signals
rs6882657	rs62355902	5q11.2	5	56222739	T	C	0.1532	1.15 (1.1-1.19)	1.06E-11	3.80E-14	++++++?+?+	Better signals
rs252897	rs62355902	5q11.2	5	56223000	G	A	0.4426	0.94 (0.91-0.97)	3.49E-05	1.51E-11	-+-+----+-	Better signals
rs58477254	rs62355902	5q11.2	5	56223878	AT	A	0.8468	0.87 (0.84-0.91)	1.19E-11	4.99E-14	------?-?-	Better signals
rs10684065	rs62355902	5q11.2	5	56224485	T	TTGGC	0.5386	1.07 (1.03-1.1)	6.74E-05	1.34E-11	+-+-+++??+	Better signals
rs252893	rs62355902	5q11.2	5	56224714	T	C	0.5563	1.06 (1.03-1.09)	3.19E-05	1.86E-11	+-+-++++-+	Better signals
rs61154548	rs62355902	5q11.2	5	56224720	T	C	0.0736	1.21 (1.14-1.28)	5.10E-11	9.08E-16	++++++?+?+	Better signals
rs113772507	rs62355902	5q11.2	5	56225276	T	C	0.1532	1.15 (1.1-1.19)	1.24E-11	4.04E-14	++++++?+?+	Better signals
rs173763	rs62355902	5q11.2	5	56225418	G	A	0.4594	0.94 (0.91-0.96)	1.42E-05	1.11E-11	-+-+----?-	Better signals
rs189695	rs62355902	5q11.2	5	56225769	T	C	0.5402	1.07 (1.04-1.1)	1.65E-05	1.35E-11	+-+-++++?+	Better signals
rs252889	rs62355902	5q11.2	5	56227069	C	A	0.4594	0.94 (0.91-0.96)	1.40E-05	1.21E-11	-+-+----?-	Better signals
rs142586759	rs62355902	5q11.2	5	56227379	GATAA	G	0.8498	0.88 (0.85-0.92)	1.37E-08	1.20E-12	------???-	Better signals
rs138611541	rs62355902	5q11.2	5	56228040	C	T	0.9257	0.83 (0.78-0.88)	1.02E-10	1.10E-14	------?-?-	Better signals
rs832531	rs62355902	5q11.2	5	56228401	A	G	0.5407	1.07 (1.04-1.1)	1.31E-05	1.26E-11	+-+-++++?+	Better signals
rs16886510	rs62355902	5q11.2	5	56228445	A	G	0.1543	1.14 (1.1-1.19)	2.05E-11	4.50E-14	++++++?+++	Better signals
rs78075120	rs62355902	5q11.2	5	56228467	A	G	0.1554	1.14 (1.1-1.19)	2.27E-11	4.49E-14	++++++?+++	Better signals
rs76869724	rs62355902	5q11.2	5	56228495	C	T	0.8447	0.88 (0.84-0.91)	2.30E-11	4.24E-14	------?---	Better signals

rs16886518	rs62355902	5q11.2	5	56233737	A	C	0.3257	0.92 (0.89-0.95)	5.16E-07	3.22E-04	---+--+---	Better signals
rs12514633	rs62355902	5q11.2	5	56236013	T	C	0.1669	1.13 (1.09-1.18)	6.26E-11	6.29E-14	++++++?+++	Better signals
rs16886525	rs62355902	5q11.2	5	56237665	A	C	0.1703	1.13 (1.09-1.17)	7.37E-11	6.84E-14	++++++?+++	Better signals
rs201971331	rs62355902	5q11.2	5	56237882	C	CAGTTAAGTTTA	0.9297	0.85 (0.8-0.91)	8.45E-07	6.21E-12	------???-	Better signals
rs75713680	rs62355902	5q11.2	5	56238204	A	G	0.1703	1.13 (1.09-1.17)	6.76E-11	6.55E-14	++++++?+++	Better signals
rs56154402	rs62355902	5q11.2	5	56241284	T	C	0.3325	0.92 (0.89-0.95)	4.20E-07	3.29E-04	---+--+---	Better signals
rs76797434	rs62355902	5q11.2	5	56241689	G	A	0.8294	0.88 (0.85-0.92)	8.96E-11	9.42E-14	------?---	Better signals
rs58568222	rs62355902	5q11.2	5	56243303	T	C	0.3254	0.92 (0.89-0.95)	4.06E-07	1.32E-04	---+--+---	Better signals
rs62356565	rs62355902	5q11.2	5	56243966	G	A	0.6747	1.09 (1.05-1.12)	5.01E-07	3.17E-04	+++-++-+++	Better signals
rs111932730	rs62355902	5q11.2	5	56244291	A	G	0.1706	1.13 (1.09-1.17)	1.26E-10	1.26E-13	++++++?+++	Better signals
rs55698001	rs62355902	5q11.2	5	56245140	G	A	0.668	1.09 (1.05-1.13)	4.98E-07	4.12E-04	+++-++-+++	Better signals
rs74455703	rs62355902	5q11.2	5	56245269	G	C	0.8292	0.88 (0.85-0.92)	1.41E-10	1.44E-13	------?---	Better signals
rs34804097	rs62355902	5q11.2	5	56246276	AAAC	A	0.5095	1.07 (1.03-1.1)	7.95E-05	3.55E-04	+-+-+++??-	Better signals
rs62356567	rs62355902	5q11.2	5	56249269	G	A	0.6675	1.09 (1.05-1.13)	7.13E-07	2.50E-04	+++-++-+++	Better signals
rs79565352	rs62355902	5q11.2	5	56251753	A	G	0.1214	1.14 (1.08-1.2)	3.67E-07	2.53E-14	++++++?+++	Better signals
rs74571895	rs62355902	5q11.2	5	56254772	G	A	0.8777	0.88 (0.83-0.92)	3.92E-07	1.16E-14	------?---	Better signals
rs78743305	rs62355902	5q11.2	5	56256217	A	G	0.122	1.14 (1.08-1.2)	5.65E-07	2.36E-14	++++++?+++	Better signals
rs7726354	rs62355902	5q11.2	5	56256483	T	C	0.1224	1.14 (1.08-1.2)	5.71E-07	3.48E-14	++++++?+++	Better signals
rs16886556	rs62355902	5q11.2	5	56260339	G	T	0.6714	1.07 (1.03-1.1)	8.33E-05	2.94E-04	++++++?+-+	Better signals
rs62356590	rs62355902	5q11.2	5	56261029	G	A	0.6921	1.07 (1.04-1.11)	3.36E-05	4.80E-04	++++++?+-+	Better signals
rs79760198	rs62355902	5q11.2	5	56262639	C	T	0.936	0.86 (0.81-0.91)	3.69E-07	1.06E-12	------?-?-	Better signals
rs79041328	rs62355902	5q11.2	5	56264375	G	A	0.9361	0.86 (0.81-0.91)	4.46E-07	1.22E-12	------?-?-	Better signals
rs6893174	rs62355902	5q11.2	5	56265025	T	C	0.0642	1.16 (1.1-1.24)	5.71E-07	7.67E-13	++++++?+?+	Better signals
rs113325879	rs62355902	5q11.2	5	56266142	A	G	0.0639	1.17 (1.1-1.24)	5.32E-07	1.41E-12	++++++?+?+	Better signals
rs142258027	rs62355902	5q11.2	5	56266278	A	G	0.0639	1.17 (1.1-1.24)	5.33E-07	1.41E-12	++++++?+?+	Better signals
rs113173541	rs62355902	5q11.2	5	56266689	A	C	0.0639	1.17 (1.1-1.24)	5.33E-07	1.65E-12	++++++?+?+	Better signals
rs78925509	rs62355902	5q11.2	5	56267141	G	A	0.9361	0.86 (0.81-0.91)	5.20E-07	1.47E-12	------?-?-	Better signals
rs113567198	rs62355902	5q11.2	5	56267155	A	AT	0.0642	1.17 (1.1-1.24)	3.47E-07	1.13E-13	++++++?+?+	Better signals
rs73122135	rs62355902	5q11.2	5	56267308	A	G	0.0643	1.17 (1.1-1.24)	4.42E-07	2.52E-13	++++++?+?+	Better signals
rs80089016	rs62355902	5q11.2	5	56267444	A	G	0.0639	1.16 (1.1-1.24)	5.75E-07	1.40E-12	++++++?+?+	Better signals
rs73122138	rs62355902	5q11.2	5	56267949	A	G	0.064	1.17 (1.1-1.24)	4.21E-07	2.57E-13	++++++?+?+	Better signals
rs80310238	rs62355902	5q11.2	5	56267984	A	C	0.0639	1.17 (1.1-1.24)	5.12E-07	1.63E-12	++++++?+?+	Better signals
rs77706078	rs62355902	5q11.2	5	56268686	A	G	0.0639	1.17 (1.1-1.24)	4.93E-07	1.16E-12	++++++?+?+	Better signals
rs144063116	rs62355902	5q11.2	5	56268884	TGGGAGC	C	0.0641	1.14 (1.07-1.22)	4.14E-05	1.51E-11	++++++???+	Better signals
rs2408651	rs62355902	5q11.2	5	56268991	C	T	0.9222	0.85 (0.8-0.9)	2.44E-09	1.03E-14	------?-?-	Better signals
rs79459889	rs62355902	5q11.2	5	56269286	A	G	0.0775	1.18 (1.11-1.24)	3.67E-09	8.56E-14	++++++?+-+	Better signals
rs111773762	rs62355902	5q11.2	5	56269336	C	T	0.9225	0.85 (0.81-0.9)	3.47E-09	7.87E-14	------?-+-	Better signals
rs80097053	rs62355902	5q11.2	5	56269492	T	C	0.0775	1.18 (1.12-1.24)	2.88E-09	6.77E-14	++++++?+-+	Better signals
rs59536253	rs62355902	5q11.2	5	56269512	G	A	0.9222	0.85 (0.8-0.89)	1.94E-09	1.04E-14	------?-?-	Better signals
rs112032073	rs62355902	5q11.2	5	56270717	T	G	0.0775	1.18 (1.12-1.24)	2.99E-09	4.82E-14	++++++?+++	Better signals
rs62356591	rs62355902	5q11.2	5	56271724	T	C	0.3075	0.93 (0.9-0.96)	4.00E-05	2.65E-04	------?-+-	Better signals
rs72759746	rs62355902	5q11.2	5	56275927	T	C	0.3005	0.93 (0.9-0.97)	6.20E-05	3.86E-04	------?---	Better signals
rs66585063	rs9397437,rs2747652	6q25.1	6	151952645	T	G	0.03	1.2 (1.11-1.3)	9.93E-06	4.99E-17	++++++++++	Secondary signals
rs9784821	rs9397437,rs2747652	6q25.1	6	151953309	C	T	0.9701	0.83 (0.77-0.9)	9.79E-06	6.34E-17	----------	Secondary signals
rs11753383	rs9397437,rs2747652	6q25.1	6	151954659	A	G	0.0304	1.2 (1.1-1.3)	1.45E-05	5.03E-17	+-++++++++	Secondary signals
rs11759768	rs9397437,rs2747652	6q25.1	6	151955481	G	C	0.9706	0.82 (0.76-0.9)	4.86E-06	1.40E-15	-+--------	Secondary signals
rs73001924	rs9397437,rs2747652	6q25.1	6	151955995	T	A	0.9701	0.83 (0.77-0.9)	7.65E-06	1.34E-15	----------	Secondary signals
rs12203668	rs9397437,rs2747652	6q25.1	6	151956625	G	A	0.97	0.83 (0.76-0.9)	6.36E-06	1.57E-15	----------	Secondary signals
rs11155806	rs9397437,rs2747652	6q25.1	6	151956890	A	C	0.0297	1.21 (1.11-1.31)	6.89E-06	1.62E-15	++++++++++	Secondary signals
rs66688403	rs9397437,rs2747652	6q25.1	6	151958153	C	A	0.9709	0.83 (0.77-0.91)	1.60E-05	3.92E-13	-+--------	Secondary signals
rs10872677	rs9397437,rs2747652	6q25.1	6	151959249	T	C	0.0297	1.21 (1.11-1.31)	7.12E-06	1.64E-15	++++++++++	Secondary signals

rs12197785	rs9397437,rs2747652	6q25.1	6	151960605	G	C	0.9702	0.83 (0.76-0.9)	6.49E-06	1.44E-15	----------	Secondary signals
rs67413479	rs9397437,rs2747652	6q25.1	6	151960929	A	G	0.0304	1.21 (1.11-1.31)	5.13E-06	1.19E-15	++++++++++	Secondary signals
rs3020313	rs9397437,rs2747652	6q25.1	6	151961900	A	G	0.0299	1.21 (1.11-1.31)	6.42E-06	1.15E-15	++++++++++	Secondary signals
rs115713943	rs9397437,rs2747652	6q25.1	6	151967935	G	A	0.9693	0.83 (0.77-0.9)	1.33E-05	3.99E-14	----------	Secondary signals
rs3020302	rs9397437,rs2747652	6q25.1	6	151969326	G	A	0.9695	0.83 (0.77-0.91)	1.40E-05	1.64E-14	----------	Secondary signals
rs11155807	rs9397437,rs2747652	6q25.1	6	151969362	A	T	0.0305	1.2 (1.11-1.3)	1.36E-05	1.47E-14	++++++++++	Secondary signals
rs3020331	rs9397437,rs2747652	6q25.1	6	152008780	T	C	0.1357	1.13 (1.08-1.17)	9.10E-10	5.14E-21	++++++++++	Secondary signals
rs3020332	rs9397437,rs2747652	6q25.1	6	152008924	T	C	0.1636	1.08 (1.04-1.12)	1.30E-05	2.82E-15	++++++++++	Secondary signals
rs2941741	rs9397437,rs2747652	6q25.1	6	152008982	A	G	0.1347	1.13 (1.08-1.17)	8.19E-10	5.22E-24	++++++++++	Secondary signals
rs2941740	rs9397437,rs2747652	6q25.1	6	152009638	G	A	0.8645	0.89 (0.86-0.92)	1.90E-09	1.39E-23	----------	Secondary signals
rs3020333	rs9397437,rs2747652	6q25.1	6	152010254	G	A	0.8167	0.93 (0.9-0.96)	2.27E-05	2.36E-15	---+------	Secondary signals
rs142786557	rs9397437,rs2747652	6q25.1	6	152010370	A	AT	0.1362	1.13 (1.08-1.17)	4.06E-09	1.21E-22	++++++++?+	Secondary signals
rs2982573	rs9397437,rs2747652	6q25.1	6	152010534	C	T	0.8648	0.89 (0.86-0.92)	1.24E-09	1.12E-23	----------	Secondary signals
rs2982572	rs9397437,rs2747652	6q25.1	6	152010561	T	C	0.1352	1.13 (1.08-1.17)	1.00E-09	7.35E-24	++++++++++	Secondary signals
rs2982571	rs9397437,rs2747652	6q25.1	6	152012739	T	A	0.8633	0.89 (0.86-0.93)	2.56E-09	2.91E-24	----------	Secondary signals
rs3020334	rs9397437,rs2747652	6q25.1	6	152012956	G	A	0.8642	0.89 (0.86-0.93)	3.39E-09	3.99E-24	----------	Secondary signals
rs2941742	rs9397437,rs2747652	6q25.1	6	152012988	G	A	0.8632	0.89 (0.86-0.93)	2.50E-09	3.35E-24	----------	Secondary signals
rs3020335	rs9397437,rs2747652	6q25.1	6	152013223	T	G	0.1365	1.12 (1.08-1.17)	1.95E-09	9.16E-25	++++++++++	Secondary signals
rs2982570	rs9397437,rs2747652	6q25.1	6	152013748	T	C	0.1395	1.12 (1.08-1.17)	1.32E-09	9.24E-25	++++++++++	Secondary signals
rs3020336	rs9397437,rs2747652	6q25.1	6	152013760	T	G	0.1368	1.12 (1.08-1.17)	2.44E-09	1.09E-24	++++++++++	Secondary signals
rs2941739	rs9397437,rs2747652	6q25.1	6	152014804	C	G	0.1645	1.08 (1.04-1.12)	2.41E-05	1.55E-18	++++++++++	Secondary signals
rs141433618	rs9397437,rs2747652	6q25.1	6	152016083	A	AAGTGTGCC	0.0268	0.83 (0.76-0.91)	8.76E-05	3.79E-09	-------??-	Secondary signals
rs862346	rs9397437,rs2747652	6q25.1	6	152016369	T	A	0.8409	0.93 (0.89-0.96)	2.56E-05	1.61E-21	+---------	Secondary signals
rs851996	rs9397437,rs2747652	6q25.1	6	152016803	T	C	0.159	1.08 (1.04-1.12)	2.47E-05	6.05E-22	-+++++++++	Secondary signals
rs1293936	rs9397437,rs2747652	6q25.1	6	152017691	G	T	0.8404	0.93 (0.89-0.96)	2.80E-05	6.90E-21	+---------	Secondary signals
rs1293935	rs9397437,rs2747652	6q25.1	6	152017958	G	C	0.8219	0.93 (0.9-0.96)	3.48E-05	4.06E-21	---+------	Secondary signals
rs200496833	rs9397437,rs2747652	6q25.1	6	152018902	A	ATTCTATAGC	0.0269	0.83 (0.76-0.91)	7.70E-05	3.37E-09	-------??-	Secondary signals
rs851985	rs9397437,rs2747652	6q25.1	6	152020390	A	C	0.8675	0.89 (0.86-0.93)	4.99E-09	4.99E-09	---------?	Secondary signals
rs34905961	rs9397437,rs2747652	6q25.1	6	152021803	C	CTTT	0.8733	0.9 (0.87-0.94)	1.26E-06	3.32E-29	--------?-	Secondary signals
rs113950968	rs9397437,rs2747652	6q25.1	6	152022346	GT	G	0.8183	0.92 (0.89-0.96)	3.68E-05	2.85E-22	--------?-	Secondary signals
rs851984	rs9397437,rs2747652	6q25.1	6	152023191	A	G	0.1299	1.12 (1.08-1.17)	5.82E-09	6.82E-32	++++++++++	Secondary signals
rs851983	rs9397437,rs2747652	6q25.1	6	152024415	G	A	0.8702	0.89 (0.86-0.93)	8.23E-09	1.39E-31	----------	Secondary signals
rs851982	rs9397437,rs2747652	6q25.1	6	152024985	C	T	0.8691	0.9 (0.86-0.93)	1.74E-08	3.43E-31	----------	Secondary signals
rs74366500	rs9397437,rs2747652	6q25.1	6	152026689	A	G	0.9513	0.85 (0.8-0.9)	3.23E-07	3.23E-07	---------?	Secondary signals
rs851981	rs9397437,rs2747652	6q25.1	6	152027074	T	A	0.8715	0.89 (0.86-0.93)	1.27E-08	4.37E-22	----------	Secondary signals
rs851980	rs9397437,rs2747652	6q25.1	6	152027955	C	T	0.8708	0.9 (0.86-0.93)	2.26E-08	4.54E-22	----------	Secondary signals
rs851975	rs9397437,rs2747652	6q25.1	6	152031303	T	C	0.1289	1.12 (1.07-1.16)	1.85E-08	2.74E-21	++++++++++	Secondary signals
rs139680793	rs9397437,rs2747652	6q25.1	6	152034062	A	G	0.1289	1.12 (1.08-1.16)	1.66E-08	2.99E-21	++++++++++	Secondary signals
rs141222583	rs9397437,rs2747652	6q25.1	6	152034624	A	G	0.1292	1.12 (1.07-1.16)	1.86E-08	4.00E-21	++++++++++	Secondary signals
rs150182883	rs9397437,rs2747652	6q25.1	6	152034758	T	C	0.1265	1.12 (1.08-1.16)	1.90E-08	3.95E-21	++++++++++	Secondary signals
rs115931026	rs9397437,rs2747652	6q25.1	6	152034820	C	T	0.8732	0.89 (0.86-0.93)	1.94E-08	3.44E-21	----------	Secondary signals
rs2206948	rs9397437,rs2747652	6q25.1	6	152037557	A	T	0.128	1.12 (1.08-1.16)	1.42E-08	2.92E-21	++++++++++	Secondary signals
rs6932703	rs9397437,rs2747652	6q25.1	6	152038240	A	G	0.1295	1.12 (1.07-1.16)	2.47E-08	2.15E-21	++++++++++	Secondary signals
rs12526447	rs9397437,rs2747652	6q25.1	6	152040125	G	A	0.8699	0.9 (0.86-0.93)	2.98E-08	6.54E-21	----------	Secondary signals
rs3020339	rs9397437,rs2747652	6q25.1	6	152040615	A	G	0.1303	1.11 (1.07-1.16)	3.41E-08	2.94E-21	++++++++++	Secondary signals
rs75212262	rs9397437,rs2747652	6q25.1	6	152040877	G	T	0.943	0.86 (0.81-0.91)	4.82E-07	2.07E-08	---+------	Secondary signals
rs10484921	rs9397437,rs2747652	6q25.1	6	152042260	A	C	0.1297	1.12 (1.07-1.16)	1.71E-08	4.22E-21	++++++++++	Secondary signals
rs11755191	rs9397437,rs2747652	6q25.1	6	152044107	C	T	0.9432	0.86 (0.81-0.91)	5.36E-07	4.99E-09	---+------	Secondary signals
rs7749659	rs9397437,rs2747652	6q25.1	6	152044884	G	A	0.8695	0.9 (0.86-0.93)	4.33E-08	3.00E-21	----------	Secondary signals
rs6940919	rs9397437,rs2747652	6q25.1	6	152047476	G	T	0.8544	0.92 (0.89-0.95)	7.28E-06	1.81E-19	----------	Secondary signals
rs2982561	rs9397437,rs2747652	6q25.1	6	152052652	C	T	0.1828	1.09 (1.06-1.13)	1.74E-07	6.03E-16	++++++++++	Secondary signals

rs6904031	rs9397437,rs2747652	6q25.1	6	152055978	T	A	0.9156	0.89 (0.85-0.93)	1.23E-06	1.27E-25	----------	Secondary signals
rs2982558	rs9397437,rs2747652	6q25.1	6	152056146	A	G	0.8169	0.91 (0.88-0.94)	8.62E-08	3.36E-16	----------	Secondary signals
rs2982556	rs9397437,rs2747652	6q25.1	6	152056842	G	A	0.1829	1.1 (1.06-1.13)	1.12E-07	3.93E-16	++++++++++	Secondary signals
rs3020300	rs9397437,rs2747652	6q25.1	6	152058844	T	A	0.1781	1.1 (1.06-1.13)	2.03E-07	4.50E-16	++++++++++	Secondary signals
rs2982552	rs9397437,rs2747652	6q25.1	6	152059563	G	A	0.1794	1.09 (1.06-1.13)	2.92E-07	1.36E-15	++++++++++	Secondary signals
rs2982551	rs9397437,rs2747652	6q25.1	6	152061210	G	T	0.2019	1.09 (1.06-1.13)	1.72E-07	9.94E-16	++++++++++	Secondary signals
rs2982550	rs9397437,rs2747652	6q25.1	6	152061474	G	T	0.202	1.09 (1.06-1.13)	1.73E-07	1.42E-15	++++++++++	Secondary signals
rs3020301	rs9397437,rs2747652	6q25.1	6	152061579	T	C	0.7983	0.91 (0.88-0.95)	1.69E-07	1.40E-15	----------	Secondary signals
rs1361024	rs9397437,rs2747652	6q25.1	6	152070928	A	G	0.085	1.12 (1.07-1.17)	4.47E-06	2.98E-17	++++++++++	Secondary signals
rs9918437	rs9397437,rs2747652	6q25.1	6	152072718	T	G	0.084	1.12 (1.06-1.17)	7.19E-06	1.12E-17	++++++++++	Secondary signals
rs6935443	rs9397437,rs2747652	6q25.1	6	152114448	G	A	0.5594	1.07 (1.04-1.1)	7.99E-07	3.23E-03	++++++++++	Secondary signals
rs78880143	rs9397437,rs2747652	6q25.1	6	152117128	C	CA	0.4992	0.95 (0.92-0.97)	9.68E-05	1.53E-03	-------+?+	Secondary signals
rs2881766	rs9397437,rs2747652	6q25.1	6	152119119	G	T	0.5582	1.07 (1.04-1.1)	5.10E-07	4.99E-03	++++++++++	Secondary signals
rs9478244	rs9397437,rs2747652	6q25.1	6	152122037	G	A	0.5629	1.07 (1.04-1.1)	7.73E-07	5.13E-03	+++++++-++	Secondary signals
rs6914438	rs9397437,rs2747652	6q25.1	6	152129588	T	C	0.2847	0.93 (0.9-0.96)	1.15E-06	4.59E-08	----------	Secondary signals
rs3778609	rs9397437,rs2747652	6q25.1	6	152133187	T	C	0.2839	0.93 (0.91-0.96)	1.16E-06	4.84E-08	----------	Secondary signals
rs3778610	rs9397437,rs2747652	6q25.1	6	152133351	G	A	0.7156	1.07 (1.04-1.11)	1.03E-06	1.13E-07	++++++++++	Secondary signals
rs7771894	rs9397437,rs2747652	6q25.1	6	152145916	T	C	0.2821	0.93 (0.9-0.96)	1.04E-06	3.81E-08	----------	Secondary signals
rs9340844	rs9397437,rs2747652	6q25.1	6	152201624	A	G	0.1015	0.89 (0.85-0.93)	2.09E-07	2.09E-07	---------?	Secondary signals
rs12176058	rs9397437,rs2747652	6q25.1	6	152209151	T	C	0.1017	0.89 (0.85-0.93)	1.33E-07	1.33E-07	---------?	Secondary signals
rs12176079	rs9397437,rs2747652	6q25.1	6	152209314	A	G	0.1017	0.89 (0.85-0.93)	1.33E-07	1.33E-07	---------?	Secondary signals
rs9397452	rs9397437,rs2747652	6q25.1	6	152215626	T	C	0.1012	0.89 (0.85-0.93)	8.47E-08	8.47E-08	---------?	Secondary signals
rs9371561	rs9397437,rs2747652	6q25.1	6	152218393	A	G	0.1012	0.89 (0.85-0.93)	8.13E-08	8.13E-08	---------?	Secondary signals
rs371510396	rs9397437,rs2747652	6q25.1	6	152219519	A	AT	0.1348	0.91 (0.87-0.95)	2.68E-05	4.35E-06	--+-----?-	Secondary signals
rs186812658	rs9397437,rs2747652	6q25.1	6	152220900	A	C	0.1022	0.89 (0.85-0.93)	6.65E-08	6.65E-08	---------?	Secondary signals
rs12174347	rs9397437,rs2747652	6q25.1	6	152221531	A	C	0.1021	0.89 (0.85-0.93)	6.61E-08	6.61E-08	---------?	Secondary signals
rs9383594	rs9397437,rs2747652	6q25.1	6	152221861	C	G	0.8979	1.13 (1.08-1.18)	6.66E-08	6.66E-08	+++++++++?	Secondary signals
rs74847648	rs9397437,rs2747652	6q25.1	6	152222490	A	G	0.1021	0.89 (0.85-0.93)	6.59E-08	6.59E-08	---------?	Secondary signals
rs9340866	rs9397437,rs2747652	6q25.1	6	152224651	A	T	0.1787	0.93 (0.9-0.96)	1.49E-05	3.75E-05	----------	Secondary signals
rs35232263	rs9397437,rs2747652	6q25.1	6	152234697	G	A	0.821	1.08 (1.04-1.12)	1.21E-05	4.42E-05	++++++++++	Secondary signals
rs9322343	rs9397437,rs2747652	6q25.1	6	152237759	G	A	0.8164	1.08 (1.04-1.11)	1.15E-05	3.44E-05	++++++++++	Secondary signals
rs9322344	rs9397437,rs2747652	6q25.1	6	152237780	C	T	0.8165	1.08 (1.04-1.11)	1.24E-05	4.36E-05	++++++++++	Secondary signals
rs9397072	rs9397437,rs2747652	6q25.1	6	152239321	T	C	0.1836	0.93 (0.9-0.96)	8.37E-06	3.17E-05	----------	Secondary signals
rs5880952	rs9397437,rs2747652	6q25.1	6	152319503	C	CT	0.6747	0.94 (0.92-0.97)	8.19E-05	1.36E-07	--------?-	Secondary signals
rs722209	rs9397437,rs2747652	6q25.1	6	152323196	T	C	0.3278	1.06 (1.03-1.09)	9.79E-05	1.88E-07	++++++++++	Secondary signals
rs2207231	rs9397437,rs2747652	6q25.1	6	152329884	G	A	0.6767	0.95 (0.92-0.97)	6.58E-05	1.31E-07	----------	Secondary signals
rs71979928	rs9397437,rs2747652	6q25.1	6	152332042	C	CTCT	0.679	0.94 (0.91-0.97)	4.13E-05	2.98E-08	--------?-	Secondary signals
rs13203975	rs9397437,rs2747652	6q25.1	6	152333104	A	G	0.3233	1.06 (1.03-1.09)	6.72E-05	1.36E-07	++++++++++	Secondary signals
rs12663193	rs9397437,rs2747652	6q25.1	6	152335236	A	G	0.3227	1.06 (1.03-1.09)	7.55E-05	5.42E-08	++++++++++	Secondary signals
rs34862067	rs9397437,rs2747652	6q25.1	6	152339974	AAAG	A	0.6812	0.94 (0.91-0.97)	6.42E-05	2.53E-07	-------??-	Secondary signals
rs2207232	rs9397437,rs2747652	6q25.1	6	152340288	C	T	0.6398	0.95 (0.92-0.97)	7.26E-05	2.16E-07	--------+-	Secondary signals
rs67690147	rs9397437,rs2747652	6q25.1	6	152340494	A	G	0.3596	1.06 (1.03-1.09)	7.31E-05	1.87E-07	++++++++-+	Secondary signals
rs17082033	rs9397437,rs2747652	6q25.1	6	152340648	T	A	0.6403	0.95 (0.92-0.97)	7.81E-05	5.41E-08	--------+-	Secondary signals
rs9340985	rs9397437,rs2747652	6q25.1	6	152341587	C	T	0.6462	0.94 (0.92-0.97)	2.05E-05	1.57E-08	--------+-	Secondary signals
rs9340995	rs9397437,rs2747652	6q25.1	6	152342779	C	G	0.3587	1.06 (1.03-1.08)	9.69E-05	4.84E-08	++++++++-+	Secondary signals
rs3020418	rs9397437,rs2747652	6q25.1	6	152345162	A	G	0.4452	1.06 (1.03-1.09)	1.20E-05	5.59E-09	+-++++++++	Secondary signals
rs113533024	rs9397437,rs2747652	6q25.1	6	152346190	T	TC	0.4452	1.06 (1.04-1.09)	7.18E-06	3.41E-09	+-++++++?+	Secondary signals
rs3020425	rs9397437,rs2747652	6q25.1	6	152353512	T	C	0.4417	1.06 (1.03-1.09)	8.68E-06	4.91E-09	+-++++++++	Secondary signals
rs3020426	rs9397437,rs2747652	6q25.1	6	152353642	G	A	0.5583	0.94 (0.92-0.97)	4.98E-06	3.58E-09	-+--------	Secondary signals
rs11155828	rs9397437,rs2747652	6q25.1	6	152353765	G	A	0.5571	0.94 (0.92-0.97)	6.14E-06	4.01E-09	-+--------	Secondary signals
rs9383604	rs9397437,rs2747652	6q25.1	6	152354307	G	A	0.5579	0.94 (0.92-0.97)	7.66E-06	4.60E-09	-+--------	Secondary signals

rs9383959	rs9397437,rs2747652	6q25.1	6	152354695	T	C	0.445	1.06 (1.04-1.09)	5.22E-06	4.56E-09	+-++++++?+	Secondary signals
rs79388591	rs9397437,rs2747652	6q25.1	6	152355649	GT	G	0.67	0.94 (0.91-0.97)	2.14E-05	3.38E-06	--------?-	Secondary signals
rs926778	rs9397437,rs2747652	6q25.1	6	152355782	A	C	0.4441	1.06 (1.03-1.09)	7.55E-06	1.58E-09	+-++++++++	Secondary signals
rs926779	rs9397437,rs2747652	6q25.1	6	152355920	A	G	0.4441	1.06 (1.03-1.09)	7.55E-06	1.58E-09	+-++++++++	Secondary signals
rs2982708	rs9397437,rs2747652	6q25.1	6	152356220	C	T	0.5561	0.94 (0.92-0.97)	6.29E-06	1.80E-09	-+--------	Secondary signals
rs6941835	rs9397437,rs2747652	6q25.1	6	152356270	G	A	0.6702	0.94 (0.92-0.97)	4.97E-05	8.09E-06	----------	Secondary signals
rs2860321	rs9397437,rs2747652	6q25.1	6	152356619	T	C	0.4444	1.06 (1.03-1.09)	7.64E-06	1.07E-09	+-++++++++	Secondary signals
rs3020429	rs9397437,rs2747652	6q25.1	6	152356649	T	C	0.4444	1.06 (1.03-1.09)	8.40E-06	1.13E-09	+-++++++++	Secondary signals
rs9397472	rs9397437,rs2747652	6q25.1	6	152357047	G	A	0.6702	0.94 (0.92-0.97)	4.61E-05	7.70E-06	----------	Secondary signals
rs2982713	rs9397437,rs2747652	6q25.1	6	152359171	A	G	0.4447	1.06 (1.03-1.09)	6.44E-06	1.32E-09	+-++++++++	Secondary signals
rs2982714	rs9397437,rs2747652	6q25.1	6	152359331	A	G	0.4447	1.06 (1.03-1.09)	6.45E-06	1.32E-09	+-++++++++	Secondary signals
rs9383961	rs9397437,rs2747652	6q25.1	6	152361239	G	A	0.6701	0.95 (0.92-0.97)	5.58E-05	8.42E-06	----------	Secondary signals
rs4289664	rs9397437,rs2747652	6q25.1	6	152361383	T	C	0.33	1.06 (1.03-1.09)	5.39E-05	7.67E-06	++++++++++	Secondary signals
rs2144026	rs9397437,rs2747652	6q25.1	6	152362334	A	G	0.4447	1.06 (1.03-1.09)	6.81E-06	1.26E-09	+-++++++++	Secondary signals
rs60693153	rs9397437,rs2747652	6q25.1	6	152366829	C	G	0.3305	1.06 (1.03-1.09)	5.30E-05	7.83E-06	++++++++++	Secondary signals
rs9341004	rs9397437,rs2747652	6q25.1	6	152370248	G	C	0.6701	0.94 (0.92-0.97)	4.87E-05	6.64E-06	----------	Secondary signals
rs11285053	rs9397437,rs2747652	6q25.1	6	152379347	T	TA	0.3591	1.06 (1.03-1.09)	6.75E-05	5.12E-09	+-++++++?+	Secondary signals
rs9397482	rs9397437,rs2747652	6q25.1	6	152379539	A	G	0.3304	1.06 (1.03-1.09)	7.06E-05	7.89E-06	++++++++++	Secondary signals
rs34801860	rs9397437,rs2747652	6q25.1	6	152379816	CCTGT	C	0.3644	1.06 (1.03-1.09)	8.86E-05	6.91E-09	+-+++++??+	Secondary signals
6:152382087:C:CTTTTT	rs9397437,rs2747652	6q25.1	6	152382087	CTTTTT	C	0.3348	1.07 (1.03-1.1)	2.99E-05	2.99E-05	+++++++???	Secondary signals
rs2273207	rs9397437,rs2747652	6q25.1	6	152382325	G	A	0.6696	0.95 (0.92-0.97)	7.74E-05	8.99E-06	----------	Secondary signals
rs3798568	rs9397437,rs2747652	6q25.1	6	152383383	A	G	0.3304	1.06 (1.03-1.09)	7.86E-05	1.05E-05	++++++++++	Secondary signals
rs3778080	rs9397437,rs2747652	6q25.1	6	152385236	C	A	0.6697	0.95 (0.92-0.97)	6.10E-05	9.50E-06	----------	Secondary signals
rs3778081	rs9397437,rs2747652	6q25.1	6	152386628	G	A	0.6724	0.95 (0.92-0.97)	9.13E-05	1.72E-05	----------	Secondary signals
rs6919225	rs9397437,rs2747652	6q25.1	6	152386890	C	T	0.6464	0.95 (0.92-0.97)	4.27E-05	1.14E-08	--------+-	Secondary signals
rs3778082	rs9397437,rs2747652	6q25.1	6	152387664	A	G	0.3537	1.06 (1.03-1.09)	4.23E-05	5.47E-09	++++++++-+	Secondary signals
rs3778084	rs9397437,rs2747652	6q25.1	6	152388039	C	T	0.6464	0.95 (0.92-0.97)	3.98E-05	7.28E-09	--------+-	Secondary signals
rs3778085	rs9397437,rs2747652	6q25.1	6	152388127	G	T	0.6464	0.95 (0.92-0.97)	4.23E-05	4.91E-09	--------+-	Secondary signals
rs7762662	rs9397437,rs2747652	6q25.1	6	152388873	G	A	0.6464	0.95 (0.92-0.97)	4.18E-05	9.62E-09	--------+-	Secondary signals
rs3798572	rs9397437,rs2747652	6q25.1	6	152389170	A	G	0.3536	1.06 (1.03-1.09)	4.07E-05	4.50E-09	++++++++-+	Secondary signals
rs3798573	rs9397437,rs2747652	6q25.1	6	152389362	G	A	0.6463	0.94 (0.92-0.97)	3.82E-05	3.51E-09	--------+-	Secondary signals
rs11311524	rs9397437,rs2747652	6q25.1	6	152389665	T	TA	0.3546	1.06 (1.03-1.09)	1.51E-05	1.25E-09	++++++++?+	Secondary signals
6:152389989:C:<CN0>	rs9397437,rs2747652	6q25.1	6	152389989	<CN0>	C	0.36	1.07 (1.04-1.1)	1.66E-05	3.35E-09	+++++++??+	Secondary signals
rs373028494	rs9397437,rs2747652	6q25.1	6	152392233	C	G	0.3398	1.06 (1.03-1.09)	3.22E-05	1.63E-09	+-++++++?+	Secondary signals
rs6557198	rs9397437,rs2747652	6q25.1	6	152392439	A	G	0.3537	1.06 (1.03-1.09)	5.18E-05	1.13E-08	++++++++-+	Secondary signals
rs6928634	rs9397437,rs2747652	6q25.1	6	152392712	G	C	0.6464	0.95 (0.92-0.97)	5.27E-05	1.48E-08	--------+-	Secondary signals
rs9479190	rs9397437,rs2747652	6q25.1	6	152393112	G	A	0.6465	0.95 (0.92-0.97)	5.11E-05	1.24E-08	--------+-	Secondary signals
rs9479191	rs9397437,rs2747652	6q25.1	6	152393160	G	T	0.6465	0.95 (0.92-0.97)	5.10E-05	1.38E-08	--------+-	Secondary signals
rs9479192	rs9397437,rs2747652	6q25.1	6	152393173	T	C	0.3535	1.06 (1.03-1.09)	5.28E-05	1.03E-08	++++++++-+	Secondary signals
rs376582138	rs9397437,rs2747652	6q25.1	6	152393218	C	CGCT	0.6455	0.94 (0.91-0.97)	1.73E-05	3.73E-09	--------?-	Secondary signals
rs12055837	rs9397437,rs2747652	6q25.1	6	152393367	T	A	0.6465	0.95 (0.92-0.97)	5.20E-05	1.40E-08	--------+-	Secondary signals
rs9397483	rs9397437,rs2747652	6q25.1	6	152393650	G	A	0.6465	0.95 (0.92-0.97)	5.19E-05	1.40E-08	--------+-	Secondary signals
rs3778087	rs9397437,rs2747652	6q25.1	6	152393707	T	G	0.3535	1.06 (1.03-1.09)	5.23E-05	1.40E-08	++++++++-+	Secondary signals
rs3778088	rs9397437,rs2747652	6q25.1	6	152393721	A	G	0.3537	1.06 (1.03-1.09)	5.74E-05	1.51E-08	++++++++-+	Secondary signals
rs3778089	rs9397437,rs2747652	6q25.1	6	152393761	A	G	0.354	1.06 (1.03-1.09)	6.41E-05	1.64E-08	++++++++-+	Secondary signals
rs9397484	rs9397437,rs2747652	6q25.1	6	152394237	C	T	0.6464	0.95 (0.92-0.97)	4.82E-05	1.26E-08	--------+-	Secondary signals
rs35396879	rs9397437,rs2747652	6q25.1	6	152394332	GA	G	0.6454	0.94 (0.91-0.97)	1.70E-05	3.68E-09	--------?-	Secondary signals
rs9478274	rs9397437,rs2747652	6q25.1	6	152394525	G	A	0.6464	0.95 (0.92-0.97)	4.92E-05	1.28E-08	--------+-	Secondary signals
rs9479193	rs9397437,rs2747652	6q25.1	6	152394779	A	G	0.3536	1.06 (1.03-1.09)	5.09E-05	1.37E-08	++++++++-+	Secondary signals
rs9479194	rs9397437,rs2747652	6q25.1	6	152395139	A	G	0.3536	1.06 (1.03-1.09)	5.08E-05	1.37E-08	++++++++-+	Secondary signals
rs9478276	rs9397437,rs2747652	6q25.1	6	152395187	T	C	0.3536	1.06 (1.03-1.09)	5.08E-05	1.37E-08	++++++++-+	Secondary signals

rs9479195	rs9397437,rs2747652	6q25.1	6	152395269	C	T	0.6464	0.95 (0.92-0.97)	5.08E-05	1.37E-08	--------+-	Secondary signals
rs7776315	rs9397437,rs2747652	6q25.1	6	152395348	T	C	0.3536	1.06 (1.03-1.09)	5.08E-05	1.37E-08	++++++++-+	Secondary signals
rs7757959	rs9397437,rs2747652	6q25.1	6	152395535	G	A	0.6464	0.95 (0.92-0.97)	5.07E-05	1.37E-08	--------+-	Secondary signals
rs7766205	rs9397437,rs2747652	6q25.1	6	152395770	C	T	0.6464	0.95 (0.92-0.97)	5.07E-05	1.37E-08	--------+-	Secondary signals
rs7766550	rs9397437,rs2747652	6q25.1	6	152395965	C	T	0.6465	0.95 (0.92-0.97)	5.07E-05	1.37E-08	--------+-	Secondary signals
rs7766577	rs9397437,rs2747652	6q25.1	6	152396013	C	T	0.6464	0.95 (0.92-0.97)	5.06E-05	1.37E-08	--------+-	Secondary signals
rs7766585	rs9397437,rs2747652	6q25.1	6	152396036	T	G	0.6465	0.95 (0.92-0.97)	5.08E-05	5.08E-05	--------+?	Secondary signals
rs78028948	rs9397437,rs2747652	6q25.1	6	152396108	C	T	0.6464	0.95 (0.92-0.97)	5.06E-05	1.37E-08	--------+-	Secondary signals
rs77870622	rs9397437,rs2747652	6q25.1	6	152396141	A	T	0.3531	1.06 (1.03-1.09)	4.67E-05	1.29E-08	++++++++-+	Secondary signals
rs3798575	rs9397437,rs2747652	6q25.1	6	152396403	G	A	0.6464	0.95 (0.92-0.97)	5.04E-05	1.37E-08	--------+-	Secondary signals
rs3798576	rs9397437,rs2747652	6q25.1	6	152396586	C	T	0.6464	0.95 (0.92-0.97)	5.04E-05	1.36E-08	--------+-	Secondary signals
rs9479196	rs9397437,rs2747652	6q25.1	6	152396940	T	C	0.3537	1.06 (1.03-1.09)	4.86E-05	2.40E-08	++++++++-+	Secondary signals
rs67737839	rs9397437,rs2747652	6q25.1	6	152396945	T	C	0.3537	1.06 (1.03-1.09)	4.77E-05	2.37E-08	++++++++-+	Secondary signals
rs9478279	rs9397437,rs2747652	6q25.1	6	152396950	A	G	0.3532	1.06 (1.03-1.09)	4.54E-05	1.21E-08	++++++++-+	Secondary signals
rs9479197	rs9397437,rs2747652	6q25.1	6	152397092	C	T	0.6464	0.95 (0.92-0.97)	5.09E-05	1.38E-08	--------+-	Secondary signals
rs9479198	rs9397437,rs2747652	6q25.1	6	152397134	C	T	0.6464	0.95 (0.92-0.97)	5.11E-05	1.38E-08	--------+-	Secondary signals
rs9479199	rs9397437,rs2747652	6q25.1	6	152397278	G	A	0.6461	0.95 (0.92-0.97)	5.44E-05	1.46E-08	-+------+-	Secondary signals
rs34777288	rs9397437,rs2747652	6q25.1	6	152397463	T	C	0.354	1.06 (1.03-1.09)	5.53E-05	1.04E-08	++++++++-+	Secondary signals
rs35237247	rs9397437,rs2747652	6q25.1	6	152397584	G	A	0.6467	0.95 (0.92-0.97)	4.86E-05	1.34E-08	-+------+-	Secondary signals
rs34322424	rs9397437,rs2747652	6q25.1	6	152397602	G	A	0.6466	0.95 (0.92-0.97)	4.87E-05	1.34E-08	-+------+-	Secondary signals
rs36083386	rs9397437,rs2747652	6q25.1	6	152397912	AC	A	0.6452	0.94 (0.91-0.97)	1.80E-05	3.86E-09	-+------?-	Secondary signals
rs13211322	rs9397437,rs2747652	6q25.1	6	152398050	C	A	0.6468	0.94 (0.92-0.97)	4.18E-05	1.27E-08	-+------+-	Secondary signals
rs13211638	rs9397437,rs2747652	6q25.1	6	152398051	A	G	0.3532	1.06 (1.03-1.09)	4.19E-05	1.27E-08	+-++++++-+	Secondary signals
rs13211433	rs9397437,rs2747652	6q25.1	6	152398079	T	C	0.3545	1.06 (1.03-1.09)	4.74E-05	1.25E-08	+-++++++-+	Secondary signals
rs9478280	rs9397437,rs2747652	6q25.1	6	152398221	T	C	0.3543	1.06 (1.03-1.09)	4.69E-05	1.30E-08	+-++++++-+	Secondary signals
rs111769614	rs9397437,rs2747652	6q25.1	6	152398345	AGCTCG	A	0.6406	0.94 (0.91-0.97)	1.92E-05	8.01E-09	-+-----??-	Secondary signals
rs9478281	rs9397437,rs2747652	6q25.1	6	152398414	T	C	0.3539	1.06 (1.03-1.09)	5.51E-05	1.47E-08	+-++++++-+	Secondary signals
rs9479200	rs9397437,rs2747652	6q25.1	6	152398505	G	A	0.6461	0.95 (0.92-0.97)	5.51E-05	1.47E-08	-+------+-	Secondary signals
rs9478282	rs9397437,rs2747652	6q25.1	6	152398669	T	C	0.3519	1.06 (1.03-1.09)	4.52E-05	1.53E-08	+-++++++-+	Secondary signals
rs9479201	rs9397437,rs2747652	6q25.1	6	152398670	G	A	0.6476	0.94 (0.92-0.97)	4.55E-05	1.46E-08	-+------+-	Secondary signals
rs35807076	rs9397437,rs2747652	6q25.1	6	152398814	C	G	0.3296	1.06 (1.03-1.09)	8.13E-05	1.02E-05	++++++++++	Secondary signals
rs9478283	rs9397437,rs2747652	6q25.1	6	152398934	A	G	0.354	1.06 (1.03-1.09)	5.55E-05	1.56E-08	+-++++++-+	Secondary signals
rs9478284	rs9397437,rs2747652	6q25.1	6	152398963	C	G	0.3539	1.06 (1.03-1.09)	5.45E-05	1.45E-08	+-++++++-+	Secondary signals
rs75819951	rs9397437,rs2747652	6q25.1	6	152399345	A	G	0.3539	1.06 (1.03-1.09)	5.33E-05	1.44E-08	+-++++++-+	Secondary signals
rs9478286	rs9397437,rs2747652	6q25.1	6	152399520	A	G	0.3542	1.06 (1.03-1.09)	5.47E-05	1.66E-08	+-++++++-+	Secondary signals
rs35525869	rs9397437,rs2747652	6q25.1	6	152399523	T	C	0.3542	1.06 (1.03-1.09)	5.47E-05	1.66E-08	+-++++++-+	Secondary signals
rs35650978	rs9397437,rs2747652	6q25.1	6	152399556	A	G	0.3542	1.06 (1.03-1.09)	5.62E-05	1.70E-08	+-++++++-+	Secondary signals
rs35498177	rs9397437,rs2747652	6q25.1	6	152399690	A	G	0.3541	1.06 (1.03-1.09)	5.51E-05	1.48E-08	+-++++++-+	Secondary signals
rs35156982	rs9397437,rs2747652	6q25.1	6	152399792	G	C	0.6459	0.95 (0.92-0.97)	5.51E-05	1.48E-08	-+------+-	Secondary signals
rs9479202	rs9397437,rs2747652	6q25.1	6	152400248	G	C	0.6462	0.95 (0.92-0.97)	5.52E-05	1.48E-08	-+------+-	Secondary signals
rs9479203	rs9397437,rs2747652	6q25.1	6	152400259	A	G	0.3539	1.06 (1.03-1.09)	5.68E-05	1.52E-08	+-++++++-+	Secondary signals
rs9479204	rs9397437,rs2747652	6q25.1	6	152400529	A	G	0.3541	1.06 (1.03-1.09)	5.64E-05	1.66E-08	+-++++++-+	Secondary signals
rs9479205	rs9397437,rs2747652	6q25.1	6	152400595	G	A	0.6458	0.95 (0.92-0.97)	5.64E-05	1.66E-08	-+------+-	Secondary signals
rs9479206	rs9397437,rs2747652	6q25.1	6	152400760	T	A	0.647	0.95 (0.92-0.97)	5.03E-05	1.53E-08	-+------+-	Secondary signals
rs9479207	rs9397437,rs2747652	6q25.1	6	152400982	G	A	0.6458	0.95 (0.92-0.97)	5.67E-05	1.67E-08	-+------+-	Secondary signals
rs9479208	rs9397437,rs2747652	6q25.1	6	152401171	C	T	0.6458	0.95 (0.92-0.97)	5.56E-05	1.65E-08	-+------+-	Secondary signals
rs9479209	rs9397437,rs2747652	6q25.1	6	152401385	T	C	0.3543	1.06 (1.03-1.09)	5.69E-05	1.68E-08	+-++++++-+	Secondary signals
rs140646696	rs9397437,rs2747652	6q25.1	6	152401437	AC	A	0.6449	0.94 (0.91-0.97)	1.91E-05	4.29E-09	-+------?-	Secondary signals
rs9479210	rs9397437,rs2747652	6q25.1	6	152401651	G	A	0.6458	0.95 (0.92-0.97)	5.70E-05	1.68E-08	-+------+-	Secondary signals
rs9479213	rs9397437,rs2747652	6q25.1	6	152402201	T	C	0.3543	1.06 (1.03-1.09)	5.71E-05	1.49E-08	+-++++++-+	Secondary signals
rs9479214	rs9397437,rs2747652	6q25.1	6	152402326	C	T	0.6466	0.95 (0.92-0.97)	4.91E-05	1.51E-08	-+------+-	Secondary signals

rs9478287	rs9397437,rs2747652	6q25.1	6	152402382	A	T	0.3539	1.06 (1.03-1.09)	5.08E-05	1.55E-08	+-++++++-+	Secondary signals
rs9478288	rs9397437,rs2747652	6q25.1	6	152402457	C	G	0.3545	1.06 (1.03-1.09)	5.44E-05	1.64E-08	+-++++++-+	Secondary signals
rs9479215	rs9397437,rs2747652	6q25.1	6	152402987	T	C	0.3547	1.06 (1.03-1.09)	5.64E-05	1.87E-08	+-++++++-+	Secondary signals
rs9479216	rs9397437,rs2747652	6q25.1	6	152403128	A	C	0.3545	1.06 (1.03-1.09)	5.86E-05	1.72E-08	+-++++++-+	Secondary signals
rs12175682	rs9397437,rs2747652	6q25.1	6	152403253	T	C	0.3297	1.06 (1.03-1.09)	6.57E-05	8.97E-06	++++++++++	Secondary signals
rs9479217	rs9397437,rs2747652	6q25.1	6	152403390	T	C	0.3544	1.06 (1.03-1.09)	5.73E-05	1.70E-08	+-++++++-+	Secondary signals
rs9479218	rs9397437,rs2747652	6q25.1	6	152403418	C	T	0.6456	0.95 (0.92-0.97)	5.74E-05	1.70E-08	-+------+-	Secondary signals
rs138747530	rs9397437,rs2747652	6q25.1	6	152403644	T	C	0.3545	1.06 (1.03-1.09)	5.91E-05	1.83E-08	+-++++++-+	Secondary signals
rs144673118	rs9397437,rs2747652	6q25.1	6	152403698	C	T	0.6458	0.95 (0.92-0.97)	5.76E-05	1.70E-08	-+------+-	Secondary signals
rs141661612	rs9397437,rs2747652	6q25.1	6	152403759	G	A	0.6464	0.95 (0.92-0.97)	5.13E-05	1.57E-08	-+------+-	Secondary signals
rs138555265	rs9397437,rs2747652	6q25.1	6	152404238	G	GA	0.3554	1.06 (1.03-1.09)	1.99E-05	4.71E-09	+-++++++?+	Secondary signals
rs141867918	rs9397437,rs2747652	6q25.1	6	152404528	G	A	0.6477	0.95 (0.92-0.97)	6.44E-05	1.99E-08	-+------+-	Secondary signals
rs35084216	rs9397437,rs2747652	6q25.1	6	152404784	G	A	0.6454	0.95 (0.92-0.97)	5.89E-05	1.83E-08	-+------+-	Secondary signals
rs58461766	rs9397437,rs2747652	6q25.1	6	152404868	C	A	0.6454	0.95 (0.92-0.97)	5.88E-05	1.83E-08	-+------+-	Secondary signals
rs34075826	rs9397437,rs2747652	6q25.1	6	152404924	G	T	0.6454	0.95 (0.92-0.97)	5.89E-05	1.83E-08	-+------+-	Secondary signals
rs9397081	rs9397437,rs2747652	6q25.1	6	152405299	C	T	0.6454	0.95 (0.92-0.97)	5.91E-05	1.84E-08	-+------+-	Secondary signals
rs59223084	rs9397437,rs2747652	6q25.1	6	152405567	A	G	0.3512	1.06 (1.03-1.09)	4.84E-05	1.37E-08	+-++++++-+	Secondary signals
rs11155832	rs9397437,rs2747652	6q25.1	6	152405607	T	C	0.3519	1.06 (1.03-1.09)	6.95E-05	2.01E-08	++++++++-+	Secondary signals
rs11155833	rs9397437,rs2747652	6q25.1	6	152405608	G	A	0.6481	0.95 (0.92-0.97)	6.96E-05	2.01E-08	--------+-	Secondary signals
rs13192678	rs9397437,rs2747652	6q25.1	6	152405910	T	C	0.3547	1.06 (1.03-1.09)	5.79E-05	1.82E-08	+-++++++-+	Secondary signals
rs3822990	rs9397437,rs2747652	6q25.1	6	152405965	T	C	0.3297	1.06 (1.03-1.09)	8.61E-05	9.63E-06	++++++++++	Secondary signals
rs3842100	rs9397437,rs2747652	6q25.1	6	152406538	G	GA	0.3301	1.06 (1.03-1.09)	4.49E-05	4.34E-06	++++++++?+	Secondary signals
rs35494677	rs9397437,rs2747652	6q25.1	6	152406628	G	A	0.6703	0.95 (0.92-0.97)	8.63E-05	9.65E-06	----------	Secondary signals
rs66465244	rs9397437,rs2747652	6q25.1	6	152407061	C	A	0.6703	0.95 (0.92-0.97)	8.61E-05	8.97E-06	----------	Secondary signals
rs67933127	rs9397437,rs2747652	6q25.1	6	152407150	T	A	0.6709	0.95 (0.92-0.97)	7.49E-05	8.18E-06	----------	Secondary signals
rs13201080	rs9397437,rs2747652	6q25.1	6	152407782	C	G	0.3296	1.06 (1.03-1.09)	7.97E-05	8.47E-06	++++++++++	Secondary signals
rs17082104	rs9397437,rs2747652	6q25.1	6	152407839	A	G	0.3296	1.06 (1.03-1.09)	7.96E-05	8.47E-06	++++++++++	Secondary signals
rs3778090	rs9397437,rs2747652	6q25.1	6	152408028	T	C	0.3296	1.06 (1.03-1.09)	7.94E-05	7.87E-06	++++++++++	Secondary signals
rs3778092	rs9397437,rs2747652	6q25.1	6	152408273	G	A	0.6704	0.95 (0.92-0.97)	7.99E-05	7.91E-06	----------	Secondary signals
rs9322355	rs9397437,rs2747652	6q25.1	6	152408649	T	C	0.3296	1.06 (1.03-1.09)	7.79E-05	7.50E-06	++++++++++	Secondary signals
rs9322357	rs9397437,rs2747652	6q25.1	6	152409236	T	G	0.3294	1.06 (1.03-1.09)	7.98E-05	7.15E-06	++++++++++	Secondary signals
rs9322359	rs9397437,rs2747652	6q25.1	6	152410022	T	A	0.6706	0.95 (0.92-0.97)	7.46E-05	7.11E-06	----------	Secondary signals
rs3778093	rs9397437,rs2747652	6q25.1	6	152411179	A	G	0.3283	1.06 (1.03-1.09)	7.90E-05	5.55E-06	++++++++++	Secondary signals
rs3778094	rs9397437,rs2747652	6q25.1	6	152411198	T	C	0.328	1.06 (1.03-1.09)	7.26E-05	5.06E-06	++++++++++	Secondary signals
rs1523396	rs6964587	7q21.2	7	91400387	C	G	0.3768	0.94 (0.91-0.97)	1.11E-05	5.37E-05	------?---	Better signals
rs11763307	rs6964587	7q21.2	7	91408398	G	A	0.6231	1.07 (1.04-1.1)	6.42E-06	5.82E-05	++++++?+++	Better signals
rs4729001	rs6964587	7q21.2	7	91413417	C	T	0.623	1.07 (1.04-1.1)	6.77E-06	6.18E-05	++++++?+++	Better signals
rs17164125	rs6964587	7q21.2	7	91417796	C	T	0.6222	1.07 (1.04-1.1)	6.54E-06	4.83E-05	++++++?+++	Better signals
rs4729006	rs6964587	7q21.2	7	91429600	C	T	0.6225	1.07 (1.04-1.1)	9.49E-06	4.66E-05	++++++?+++	Better signals
rs11772428	rs6964587	7q21.2	7	91435316	A	G	0.348	0.94 (0.92-0.97)	8.19E-05	1.74E-04	------?---	Better signals
rs10239125	rs6964587	7q21.2	7	91447692	G	T	0.6329	1.07 (1.04-1.1)	1.32E-05	7.08E-05	-+++++?+++	Better signals
rs974508	rs6964587	7q21.2	7	91448017	T	C	0.3667	0.94 (0.91-0.97)	1.75E-05	1.15E-04	+-----?---	Better signals
rs12666939	rs6964587	7q21.2	7	91448958	G	C	0.6335	1.06 (1.03-1.1)	1.91E-05	8.83E-05	-+++++?+++	Better signals
rs17164154	rs6964587	7q21.2	7	91474788	C	T	0.6415	1.06 (1.03-1.09)	4.76E-05	1.19E-04	++++++++++	Better signals
rs2282973	rs6964587	7q21.2	7	91666661	C	A	0.6513	1.06 (1.03-1.09)	5.69E-05	6.65E-04	+-++++++++	Better signals
rs2049900	rs6964587	7q21.2	7	91738788	G	C	0.6503	1.06 (1.03-1.09)	6.45E-05	6.97E-04	+-++++++++	Better signals
rs36190962	rs514192	8q22.3	8	102636399	T	TCACAGTA	0.0922	0.9 (0.85-0.95)	8.12E-05	1.47E-07	-+----???-	Secondary signals
rs13273977	rs514192	8q22.3	8	102639989	C	T	0.9084	1.1 (1.05-1.16)	4.59E-05	1.21E-07	+-++++?+-+	Secondary signals
rs57645087	rs514192	8q22.3	8	102644978	A	G	0.9106	1.11 (1.05-1.16)	4.38E-05	4.01E-07	+-++++?+-+	Secondary signals
rs2226400	rs514192	8q22.3	8	102646352	G	A	0.9084	1.11 (1.05-1.16)	3.11E-05	1.18E-07	+-++++?+-+	Better signals
rs2211917	rs514192	8q22.3	8	102646403	A	G	0.0915	0.91 (0.86-0.95)	3.77E-05	1.30E-07	-+----?-+-	Secondary signals

rs73699536	rs514192	8q22.3	8	102647183	A	G	0.0893	0.9 (0.86-0.95)	3.83E-05	4.30E-07	-+----?-+-	Secondary signals
rs7010796	rs514192	8q22.3	8	102647858	G	A	0.9085	1.1 (1.05-1.16)	3.99E-05	1.08E-07	+-++++?+-+	Secondary signals
rs57337051	rs514192	8q22.3	8	102648282	A	T	0.0913	0.9 (0.86-0.95)	3.41E-05	1.14E-07	-+----?-+-	Secondary signals
rs11993425	rs514192	8q22.3	8	102648755	T	C	0.0912	0.91 (0.86-0.95)	3.78E-05	1.01E-07	-+----?-+-	Secondary signals
rs2211916	rs514192	8q22.3	8	102649304	T	C	0.0911	0.9 (0.86-0.95)	3.48E-05	8.03E-08	-+----?-+-	Secondary signals
rs35234521	rs514192	8q22.3	8	102649427	C	G	0.0913	0.91 (0.86-0.95)	3.63E-05	1.12E-07	-+----?-+-	Secondary signals
rs11995278	rs514192	8q22.3	8	102650107	A	G	0.0913	0.9 (0.86-0.95)	3.39E-05	1.02E-07	-+----?-+-	Secondary signals
rs11995290	rs514192	8q22.3	8	102650321	T	C	0.0913	0.9 (0.86-0.95)	3.38E-05	9.88E-08	-+----?-+-	Secondary signals
rs11995361	rs514192	8q22.3	8	102650378	C	G	0.0913	0.9 (0.86-0.95)	3.41E-05	3.41E-05	-+----?-+?	Secondary signals
rs11995402	rs514192	8q22.3	8	102650508	T	G	0.0912	0.9 (0.86-0.95)	3.06E-05	8.91E-08	-+----?-+-	Better signals
rs11995403	rs514192	8q22.3	8	102650509	T	G	0.0912	0.9 (0.86-0.95)	3.05E-05	8.90E-08	-+----?-+-	Better signals
rs71518834	rs514192	8q22.3	8	102650594	G	A	0.9088	1.11 (1.05-1.16)	3.04E-05	8.86E-08	+-++++?+-+	Better signals
rs10087735	rs514192	8q22.3	8	102650701	C	T	0.9088	1.11 (1.05-1.16)	3.03E-05	8.84E-08	+-++++?+-+	Better signals
rs10101105	rs514192	8q22.3	8	102650750	G	C	0.9088	1.11 (1.06-1.16)	2.98E-05	8.74E-08	+-++++?+-+	Better signals
rs10101116	rs514192	8q22.3	8	102650795	T	C	0.0912	0.9 (0.86-0.95)	2.90E-05	8.55E-08	-+----?-+-	Better signals
rs2892327	rs514192	8q22.3	8	102650879	G	A	0.9088	1.11 (1.06-1.16)	2.82E-05	8.37E-08	+-++++?+-+	Better signals
rs2892326	rs514192	8q22.3	8	102650942	A	G	0.0912	0.9 (0.86-0.95)	2.80E-05	8.33E-08	-+----?-+-	Better signals
rs2186391	rs514192	8q22.3	8	102650993	T	C	0.0913	0.9 (0.86-0.95)	2.93E-05	2.93E-05	-+----?-+?	Better signals
rs2154651	rs514192	8q22.3	8	102651070	A	G	0.0912	0.9 (0.86-0.95)	2.74E-05	8.04E-08	-+----?-+-	Better signals
rs2154650	rs514192	8q22.3	8	102651199	G	C	0.9088	1.11 (1.05-1.16)	2.92E-05	1.06E-07	+-++++?+-+	Better signals
rs1111883	rs514192	8q22.3	8	102651383	G	A	0.9095	1.11 (1.06-1.16)	2.88E-05	1.41E-07	+-++++?+-+	Better signals
rs3029410	rs514192	8q22.3	8	102651532	AGT	A	0.9086	1.12 (1.07-1.18)	7.47E-06	5.47E-08	+-++++?+?+	Better signals
rs1111882	rs514192	8q22.3	8	102651611	T	C	0.0912	0.9 (0.86-0.95)	2.93E-05	8.64E-08	-+----?-+-	Better signals
rs35477966	rs514192	8q22.3	8	102651767	G	A	0.9088	1.11 (1.05-1.16)	2.97E-05	1.12E-07	+-++++?+-+	Better signals
rs6986280	rs514192	8q22.3	8	102651937	A	T	0.0912	0.9 (0.86-0.95)	2.82E-05	8.39E-08	-+----?-+-	Better signals
rs7004970	rs514192	8q22.3	8	102652236	C	G	0.0912	0.9 (0.86-0.95)	2.87E-05	8.52E-08	-+----?-+-	Better signals
rs6986959	rs514192	8q22.3	8	102652368	C	T	0.9088	1.11 (1.05-1.16)	3.13E-05	9.60E-08	+-++++?+-+	Better signals
rs34328475	rs514192	8q22.3	8	102652531	A	G	0.0911	0.9 (0.86-0.95)	2.74E-05	2.74E-05	-+----?-+?	Better signals
rs10955267	rs514192	8q22.3	8	102652602	C	T	0.9089	1.11 (1.06-1.16)	2.86E-05	4.65E-08	+-++++?+-+	Better signals
rs7839359	rs514192	8q22.3	8	102653318	C	G	0.0897	0.9 (0.86-0.95)	1.90E-05	7.14E-08	-+----?-+-	Better signals
rs7839123	rs514192	8q22.3	8	102653327	G	A	0.9103	1.11 (1.06-1.16)	1.88E-05	7.08E-08	+-++++?+-+	Better signals
rs7839494	rs514192	8q22.3	8	102653358	T	G	0.0897	0.9 (0.86-0.95)	1.90E-05	7.14E-08	-+----?-+-	Better signals
rs7839287	rs514192	8q22.3	8	102653487	G	A	0.9104	1.11 (1.06-1.16)	1.77E-05	5.09E-08	+-++++?+-+	Better signals
rs7839801	rs514192	8q22.3	8	102653530	C	G	0.0896	0.9 (0.86-0.94)	1.79E-05	5.13E-08	-+----?-+-	Better signals
rs7839563	rs514192	8q22.3	8	102653575	T	C	0.0896	0.9 (0.86-0.94)	1.80E-05	5.65E-08	-+----?-+-	Better signals
rs35628299	rs514192	8q22.3	8	102653881	A	G	0.0895	0.9 (0.86-0.94)	1.72E-05	6.13E-08	-+----?-+-	Better signals
rs35591604	rs514192	8q22.3	8	102654032	A	G	0.0895	0.9 (0.86-0.94)	1.68E-05	6.01E-08	-+----?-+-	Better signals
rs34302508	rs514192	8q22.3	8	102654384	C	CT	0.9106	1.12 (1.07-1.18)	4.53E-06	4.12E-08	+-++++?+?+	Better signals
rs36094595	rs514192	8q22.3	8	102654853	T	A	0.9105	1.11 (1.06-1.17)	1.63E-05	5.94E-08	+-++++?+-+	Better signals
rs76192582	rs1011970	9p21.3	9	21857469	T	C	0.9466	1.17 (1.1-1.24)	6.90E-07	5.34E-08	+-++++?+++	Better signals
rs118136736	rs1011970	9p21.3	9	21857738	T	G	0.9467	1.17 (1.1-1.24)	7.71E-07	5.09E-08	+-++++?+++	Better signals
rs116877148	rs1011970	9p21.3	9	21858276	T	C	0.9467	1.17 (1.1-1.24)	6.40E-07	4.28E-08	+-++++?+++	Better signals
rs117105750	rs1011970	9p21.3	9	21858649	A	G	0.9466	1.17 (1.1-1.24)	5.30E-07	2.08E-07	+-++++?+++	Better signals
rs117659691	rs1011970	9p21.3	9	21858829	A	G	0.0533	0.86 (0.81-0.91)	5.28E-07	4.01E-08	-+----?---	Better signals
chr9:21859844:D	rs1011970	9p21.3	9	21859844	G	GA	0.0576	0.86 (0.81-0.91)	4.29E-07	3.51E-08	-+----?---	Better signals
rs113176322	rs1011970	9p21.3	9	21860824	C	G	0.9457	1.17 (1.1-1.24)	5.01E-07	7.56E-09	+-++++?+++	Better signals
rs79054071	rs1011970	9p21.3	9	21861411	T	G	0.9467	1.17 (1.1-1.24)	5.83E-07	4.60E-08	+-++++?+++	Better signals
rs78443963	rs1011970	9p21.3	9	21861449	C	G	0.9478	1.17 (1.1-1.25)	2.92E-07	2.27E-08	++++++?+++	Better signals
rs4129861	rs1011970	9p21.3	9	21861552	T	C	0.053	0.85 (0.8-0.91)	3.84E-07	5.52E-09	------?---	Better signals
rs78195856	rs1011970	9p21.3	9	21862272	A	C	0.0535	0.85 (0.8-0.91)	3.73E-07	3.73E-07	------?--?	Better signals
rs78389853	rs1011970	9p21.3	9	21862406	C	G	0.0523	0.85 (0.8-0.91)	3.38E-07	2.64E-08	------?---	Better signals

rs79546636	rs1011970	9p21.3	9	21863656	T	G	0.0523	0.85 (0.8-0.91)	3.86E-07	3.02E-08	------?---	Better signals
rs75991963	rs1011970	9p21.3	9	21867244	T	C	0.0549	0.86 (0.81-0.92)	1.14E-06	1.19E-08	-+----?---	Better signals
rs75797591	rs1011970	9p21.3	9	21867321	A	G	0.0528	0.86 (0.81-0.91)	5.54E-07	4.20E-08	------?---	Better signals
rs79821126	rs1011970	9p21.3	9	21869138	T	C	0.055	0.86 (0.81-0.92)	1.28E-06	1.03E-07	-+----?---	Better signals
rs77068755	rs1011970	9p21.3	9	21870206	A	G	0.0525	0.86 (0.81-0.91)	8.03E-07	4.85E-08	------+---	Better signals
rs73438594	rs1011970	9p21.3	9	21870343	C	G	0.9462	1.17 (1.1-1.24)	5.76E-07	7.91E-09	++++++?+++	Better signals
rs76597469	rs1011970	9p21.3	9	21871151	C	G	0.9459	1.16 (1.09-1.23)	1.05E-06	7.50E-08	+-++++-+++	Better signals
rs61431854	rs1011970	9p21.3	9	21871169	A	T	0.0553	0.86 (0.81-0.91)	6.33E-07	9.40E-09	-+----?---	Better signals
rs7032349	rs1011970	9p21.3	9	21871638	A	G	0.9443	1.16 (1.1-1.23)	7.56E-07	1.04E-08	+-++++?+++	Better signals
rs73440407	rs1011970	9p21.3	9	21871812	T	C	0.0558	0.86 (0.81-0.91)	6.47E-07	1.07E-08	-+----?---	Better signals
rs79495898	rs1011970	9p21.3	9	21872024	T	C	0.9459	1.16 (1.09-1.23)	1.07E-06	7.65E-08	+-++++-+++	Better signals
rs10125945	rs1011970	9p21.3	9	21872173	A	G	0.9435	1.16 (1.09-1.23)	8.94E-07	6.71E-08	+-++++?+++	Better signals
rs79913997	rs1011970	9p21.3	9	21872318	A	G	0.9459	1.16 (1.09-1.23)	1.07E-06	7.73E-08	+-++++-+++	Better signals
rs76059716	rs1011970	9p21.3	9	21872615	T	C	0.9459	1.16 (1.09-1.23)	1.07E-06	7.63E-08	+-++++-+++	Better signals
rs73440418	rs1011970	9p21.3	9	21873525	A	T	0.0548	0.86 (0.81-0.91)	4.91E-07	9.93E-08	-+----?---	Better signals
9:21873619:C:G	rs1011970	9p21.3	9	21873619	C	G	0.9457	1.15 (1.09-1.23)	5.46E-06	3.90E-07	+-++++-+?+	Better signals
rs115417373	rs1011970	9p21.3	9	21874626	A	G	0.0536	0.85 (0.8-0.91)	2.63E-07	9.83E-08	------?---	Better signals
rs111740773	rs1011970	9p21.3	9	21875578	A	G	0.055	0.86 (0.81-0.91)	3.77E-07	2.75E-08	------?---	Better signals
rs143447579	rs1011970	9p21.3	9	21876086	T	C	0.054	0.86 (0.81-0.91)	1.03E-06	7.23E-08	-+----+---	Better signals
9:21876216:G:GT	rs1011970	9p21.3	9	21876216	G	GT	0.9475	1.15 (1.08-1.23)	1.21E-05	4.71E-07	++++++-+?+	Better signals
rs117168139	rs1011970	9p21.3	9	21878945	A	G	0.0532	0.86 (0.81-0.91)	4.89E-07	1.85E-08	------+---	Better signals
rs7863919	rs1011970	9p21.3	9	21879713	T	C	0.0539	0.85 (0.8-0.91)	2.35E-07	4.56E-08	------?---	Better signals
rs149793082	rs1011970	9p21.3	9	21879827	A	AT	0.0538	0.86 (0.8-0.91)	2.14E-06	1.58E-07	------?-?-	Better signals
rs116907830	rs1011970	9p21.3	9	21879921	C	G	0.9472	1.17 (1.1-1.24)	5.47E-07	3.73E-08	++++++-+++	Better signals
rs73440434	rs1011970	9p21.3	9	21880909	T	C	0.0539	0.85 (0.8-0.91)	2.24E-07	4.58E-08	------?---	Better signals
rs73440436	rs1011970	9p21.3	9	21880997	A	G	0.9461	1.17 (1.1-1.25)	2.24E-07	4.35E-08	++++++?+++	Better signals
rs73440437	rs1011970	9p21.3	9	21881946	C	G	0.0539	0.85 (0.8-0.91)	2.23E-07	4.39E-08	------?---	Better signals
rs73440438	rs1011970	9p21.3	9	21882594	T	G	0.054	0.85 (0.8-0.91)	2.23E-07	4.43E-08	------?---	Better signals
chr9:21882631:D	rs1011970	9p21.3	9	21882631	CTT	C	0.9459	1.17 (1.1-1.24)	1.34E-06	2.58E-07	++++++?+?+	Better signals
rs73440439	rs1011970	9p21.3	9	21882710	A	C	0.0542	0.85 (0.8-0.91)	2.30E-07	4.58E-08	------?---	Better signals
rs7858737	rs1011970	9p21.3	9	21883859	C	G	0.9452	1.17 (1.1-1.24)	5.27E-07	1.37E-07	+-++++?+++	Better signals
rs7874275	rs1011970	9p21.3	9	21883860	T	G	0.9452	1.17 (1.1-1.24)	5.23E-07	1.36E-07	+-++++?+++	Better signals
rs117509244	rs1011970	9p21.3	9	21886501	C	G	0.0539	0.86 (0.81-0.92)	1.15E-06	8.59E-08	-+----+---	Better signals
rs80329145	rs1011970	9p21.3	9	21886894	T	C	0.0539	0.86 (0.81-0.92)	1.19E-06	8.93E-08	-+----+---	Better signals
rs142442624	rs1011970	9p21.3	9	21887738	A	G	0.0521	0.86 (0.81-0.91)	8.90E-07	7.05E-08	-+----+---	Better signals
rs111644260	rs1011970	9p21.3	9	21888361	A	G	0.9455	1.17 (1.1-1.24)	4.87E-07	2.95E-08	+-++++?+++	Better signals
rs79008931	rs1011970	9p21.3	9	21889885	A	G	0.9461	1.16 (1.09-1.23)	1.30E-06	9.99E-08	+-++++-+++	Better signals
rs73440457	rs1011970	9p21.3	9	21890153	A	G	0.0537	0.86 (0.8-0.91)	6.83E-07	3.27E-08	-+----?---	Better signals
rs2518717	rs1011970	9p21.3	9	21959751	C	T	0.6257	0.94 (0.91-0.96)	1.40E-05	6.90E-13	+-+-------	Better signals
rs2106115	rs1011970	9p21.3	9	21959900	T	C	0.2162	1.09 (1.06-1.13)	1.74E-07	6.58E-09	++++++++++	Better signals
rs2106116	rs1011970	9p21.3	9	21959966	T	C	0.2164	1.09 (1.06-1.13)	1.71E-07	6.53E-09	++++++++++	Better signals
rs2263146	rs1011970	9p21.3	9	21963048	T	C	0.3748	1.07 (1.04-1.1)	7.34E-06	2.35E-13	-+-+++++++	Better signals
rs34011899	rs1011970	9p21.3	9	21968712	A	C	0.1829	1.1 (1.06-1.14)	1.96E-07	1.96E-07	+++++++++?	Better signals
rs3731245	rs1011970	9p21.3	9	21972445	T	C	0.1833	1.1 (1.06-1.14)	1.68E-07	1.68E-07	+++++++++?	Better signals
rs2811708	rs1011970	9p21.3	9	21973422	T	G	0.2157	1.1 (1.06-1.13)	4.17E-08	4.17E-08	+++++++++?	Better signals
rs36228836	rs1011970	9p21.3	9	21975141	A	T	0.8166	0.91 (0.88-0.94)	1.71E-07	1.71E-07	---------?	Better signals
chr9:21975681:D	rs1011970	9p21.3	9	21975681	CG	C	0.8164	0.91 (0.88-0.94)	1.88E-07	1.88E-07	---------?	Better signals
rs36228502	rs1011970	9p21.3	9	21975695	A	G	0.1836	1.1 (1.06-1.13)	1.88E-07	1.88E-07	+++++++++?	Better signals
rs186966895	rs1011970	9p21.3	9	21978085	A	G	0.1836	1.1 (1.06-1.13)	1.48E-07	1.48E-07	+++++++++?	Better signals
rs191598357	rs1011970	9p21.3	9	21978086	A	T	0.8164	0.91 (0.88-0.94)	1.49E-07	1.49E-07	---------?	Better signals
rs2518720	rs1011970	9p21.3	9	21978979	T	C	0.3298	1.08 (1.05-1.11)	4.11E-07	6.11E-13	-+++++++++	Better signals

rs77238175	rs1011970	9p21.3	9	21979042	A	C	0.8149	0.91 (0.88-0.95)	2.27E-07	2.27E-07	---------?	Better signals
rs7874405	rs1011970	9p21.3	9	21980944	C	T	0.4652	1.07 (1.04-1.11)	1.49E-05	2.11E-02	+++++++-?-	Better signals
rs35302371	rs1011970	9p21.3	9	21982478	A	AT	0.1941	1.08 (1.05-1.12)	6.86E-06	6.86E-06	++++++-+??	Better signals
rs3731217	rs1011970	9p21.3	9	21984661	C	A	0.8031	0.92 (0.89-0.95)	3.21E-07	3.03E-16	--+---+---	Better signals
rs2811710	rs1011970	9p21.3	9	21991923	T	C	0.324	1.08 (1.05-1.11)	5.24E-07	1.70E-12	-+++++++++	Better signals
rs78545330	rs1011970	9p21.3	9	21995941	A	T	0.2189	1.09 (1.06-1.13)	2.07E-08	1.10E-02	+++++++++-	Better signals
rs113646886	rs1011970	9p21.3	9	21996645	C	CCT	0.7812	0.92 (0.89-0.95)	7.64E-07	4.05E-02	--------?+	Better signals
rs3218010	rs1011970	9p21.3	9	21998733	T	C	0.8044	0.91 (0.88-0.94)	7.89E-08	7.89E-08	--+---+--?	Better signals
rs3218008	rs1011970	9p21.3	9	21998891	A	AAC	0.2195	1.09 (1.05-1.12)	6.28E-07	4.15E-02	++-+++++?-	Better signals
rs3218007	rs1011970	9p21.3	9	21999800	T	C	0.2202	1.1 (1.06-1.13)	9.86E-09	9.00E-03	++-++++++-	Better signals
rs3218005	rs1011970	9p21.3	9	22000247	C	T	0.7798	0.91 (0.88-0.94)	6.85E-09	8.25E-03	--+------+	Better signals
rs3218002	rs1011970	9p21.3	9	22000841	A	G	0.2199	1.1 (1.06-1.13)	7.91E-09	6.66E-03	++-++++++-	Better signals
chr9:22002556:D	rs1011970	9p21.3	9	22002556	T	TG	0.1949	1.1 (1.06-1.13)	5.14E-08	5.14E-08	++-+++-++?	Better signals
rs974336	rs1011970	9p21.3	9	22006348	T	C	0.2226	1.1 (1.06-1.13)	5.89E-09	6.74E-03	++-++++++-	Better signals
rs2285327	rs1011970	9p21.3	9	22007048	T	C	0.8024	0.91 (0.88-0.94)	4.88E-08	4.88E-08	--+---+--?	Better signals
chr9:22009623:I	rs1011970	9p21.3	9	22009623	CA	C	0.1961	1.1 (1.06-1.13)	4.53E-08	4.53E-08	++-++++++?	Better signals
rs3808845	rs1011970	9p21.3	9	22010575	A	G	0.1959	1.1 (1.06-1.13)	5.04E-08	5.04E-08	++-++++++?	Better signals
rs3808846	rs1011970	9p21.3	9	22010946	A	G	0.1959	1.1 (1.06-1.13)	4.86E-08	4.86E-08	++-++++++?	Better signals
rs545226	rs1011970	9p21.3	9	22012422	G	A	0.4404	1.06 (1.03-1.09)	4.47E-05	4.72E-14	+--+++++++	Better signals
rs34662415	rs1011970	9p21.3	9	22012620	G	GA	0.2682	1.08 (1.05-1.12)	1.58E-06	5.05E-03	++++++++?+	Better signals
chr9:22012789:D	rs1011970	9p21.3	9	22012789	G	GA	0.2214	1.1 (1.06-1.13)	7.21E-09	7.21E-09	++-++++++?	Better signals
rs116876910	rs1011970	9p21.3	9	22014411	A	G	0.196	1.1 (1.06-1.13)	4.39E-08	4.39E-08	++-++++++?	Better signals
rs643319	rs1011970	9p21.3	9	22017836	A	C	0.3501	1.06 (1.03-1.09)	2.83E-05	1.05E-09	++-+++++++	Better signals
rs149401414	rs1011970	9p21.3	9	22018125	C	G	0.8031	0.91 (0.88-0.94)	3.30E-08	3.30E-08	--+------?	Better signals
rs138417214	rs1011970	9p21.3	9	22018437	A	G	0.8029	0.91 (0.88-0.94)	3.75E-08	3.75E-08	--+------?	Better signals
rs76219447	rs1011970	9p21.3	9	22018718	C	G	0.8031	0.91 (0.88-0.94)	3.61E-08	3.61E-08	--+---+--?	Better signals
rs490005	rs1011970	9p21.3	9	22020493	A	G	0.3506	1.06 (1.03-1.09)	4.03E-05	4.30E-09	+--+++++++	Better signals
rs568447	rs1011970	9p21.3	9	22021615	G	A	0.438	1.06 (1.03-1.09)	4.98E-05	1.29E-14	++-+++++++	Better signals
rs567453	rs1011970	9p21.3	9	22021737	C	G	0.3506	1.06 (1.03-1.09)	3.12E-05	1.46E-09	++-+++++++	Better signals
rs117418282	rs1011970	9p21.3	9	22022454	T	C	0.1965	1.1 (1.06-1.13)	2.63E-08	2.63E-08	++-++++++?	Better signals
rs7039304	rs1011970	9p21.3	9	22022786	A	T	0.1964	1.1 (1.06-1.13)	3.03E-08	3.03E-08	++-++++++?	Better signals
rs76774391	rs1011970	9p21.3	9	22023775	C	G	0.1967	1.1 (1.06-1.13)	2.66E-08	2.66E-08	++-++++++?	Better signals
rs504318	rs1011970	9p21.3	9	22024023	T	A	0.6494	0.94 (0.92-0.97)	3.43E-05	1.01E-09	--+-------	Better signals
rs496892	rs1011970	9p21.3	9	22024351	T	C	0.3497	1.06 (1.03-1.09)	3.44E-05	6.73E-10	++-+++++++	Better signals
rs76810097	rs1011970	9p21.3	9	22027714	T	C	0.1959	1.1 (1.06-1.13)	2.83E-08	2.83E-08	++-++++++?	Better signals
rs79666073	rs1011970	9p21.3	9	22031440	A	G	0.1961	1.1 (1.06-1.13)	3.39E-08	3.39E-08	++-++++++?	Better signals
rs77706751	rs1011970	9p21.3	9	22031441	T	C	0.1961	1.1 (1.06-1.13)	3.39E-08	3.39E-08	++-++++++?	Better signals
rs74599268	rs1011970	9p21.3	9	22031778	A	T	0.8038	0.91 (0.88-0.94)	3.10E-08	3.10E-08	--+------?	Better signals
rs76521274	rs1011970	9p21.3	9	22032793	T	C	0.8098	0.91 (0.88-0.94)	2.60E-08	8.08E-07	--+------+	Better signals
rs80248631	rs1011970	9p21.3	9	22034109	T	C	0.1893	1.1 (1.06-1.14)	2.57E-08	2.57E-08	++-++++++?	Better signals
rs77953206	rs1011970	9p21.3	9	22038354	A	G	0.1857	1.1 (1.06-1.14)	2.06E-08	2.06E-08	++-++++++?	Better signals
rs78777686	rs1011970	9p21.3	9	22038615	A	G	0.1858	1.1 (1.06-1.14)	1.85E-08	1.85E-08	++-++++++?	Better signals
rs117561861	rs1011970	9p21.3	9	22040116	C	G	0.8141	0.91 (0.88-0.94)	1.96E-08	1.96E-08	--+------?	Better signals
rs75227345	rs1011970	9p21.3	9	22042297	T	C	0.1863	1.1 (1.06-1.14)	1.61E-08	1.61E-08	++-++++++?	Better signals
chr9:22042952:I	rs1011970	9p21.3	9	22042952	T	TA	0.8137	0.91 (0.88-0.94)	1.64E-08	1.64E-08	--+------?	Better signals
rs73652846	rs1011970	9p21.3	9	22043260	A	G	0.1871	1.1 (1.06-1.14)	1.55E-08	4.17E-07	++-++++++-	Better signals
rs77061973	rs1011970	9p21.3	9	22045218	C	G	0.814	0.91 (0.88-0.94)	2.53E-08	2.53E-08	--+------?	Better signals
rs17834131	rs1011970	9p21.3	9	22046168	A	G	0.1859	1.1 (1.06-1.14)	2.70E-08	2.70E-08	++-++++++?	Better signals
rs76184305	rs1011970	9p21.3	9	22046870	T	C	0.1858	1.1 (1.06-1.14)	2.95E-08	2.95E-08	++-++++++?	Better signals
rs74400540	rs1011970	9p21.3	9	22048342	A	G	0.1857	1.1 (1.06-1.14)	2.81E-08	2.81E-08	++-++++++?	Better signals
rs77792598	rs1011970	9p21.3	9	22051248	C	G	0.8143	0.91 (0.88-0.94)	3.72E-08	3.72E-08	--+------?	Better signals

chr9:22051873:D	rs1011970	9p21.3	9	22051873	T	TA	0.1868	1.1 (1.06-1.14)	4.75E-08	4.75E-08	++-++++++?	Better signals
rs117761422	rs1011970	9p21.3	9	22059054	T	G	0.1849	1.1 (1.06-1.13)	1.04E-07	1.04E-07	++-++++++?	Better signals
rs117914182	rs1011970	9p21.3	9	22059382	T	C	0.1859	1.09 (1.06-1.13)	1.24E-07	1.24E-07	++-++++++?	Better signals
rs150690757	rs1011970	9p21.3	9	22059623	T	C	0.1855	1.1 (1.06-1.13)	1.10E-07	1.10E-07	++-++++++?	Better signals
rs191148638	rs1011970	9p21.3	9	22063007	T	G	0.1703	1.1 (1.05-1.14)	9.30E-06	2.63E-09	++-+++-+?+	Better signals
rs75286189	rs10822013,rs10995201	10q21.2	10	64265965	C	T	0.786	1.08 (1.04-1.12)	1.28E-05	1.28E-06	+--+++?+++	Secondary signals
rs78993715	rs10822013,rs10995201	10q21.2	10	64266932	T	C	0.2181	0.92 (0.89-0.96)	1.09E-05	9.41E-07	-++---?---	Secondary signals
rs77818958	rs10822013,rs10995201	10q21.2	10	64269354	A	C	0.2193	0.93 (0.89-0.96)	9.53E-06	7.73E-07	-++---?---	Secondary signals
rs74232916	rs10822013,rs10995201	10q21.2	10	64270218	T	G	0.2154	0.92 (0.89-0.96)	7.99E-06	9.22E-07	-++---?---	Secondary signals
rs16917327	rs10822013,rs10995201	10q21.2	10	64272616	A	G	0.2208	0.93 (0.89-0.96)	1.20E-05	7.51E-08	-++---?---	Secondary signals
rs74205822	rs10822013,rs10995201	10q21.2	10	64282162	A	C	0.212	0.92 (0.89-0.95)	2.69E-06	1.57E-07	--+---?---	Secondary signals
rs77044930	rs10822013,rs10995201	10q21.2	10	64287935	A	G	0.2162	0.92 (0.89-0.95)	2.22E-06	1.30E-07	--+---?---	Secondary signals
rs12241616	rs10822013,rs10995201	10q21.2	10	64292879	T	C	0.2137	0.92 (0.89-0.95)	3.21E-06	1.50E-07	--+---?---	Secondary signals
rs78481535	rs10822013,rs10995201	10q21.2	10	64293803	C	T	0.7862	1.09 (1.05-1.12)	3.32E-06	1.54E-07	++-+++?+++	Secondary signals
rs78843249	rs10822013,rs10995201	10q21.2	10	64297094	G	A	0.782	1.08 (1.05-1.12)	4.39E-06	2.71E-07	+--+++?+++	Secondary signals
rs11812956	rs10822013,rs10995201	10q21.2	10	64299457	C	G	0.2175	0.92 (0.89-0.95)	3.44E-06	1.78E-07	-++---?---	Secondary signals
rs77925805	rs10822013,rs10995201	10q21.2	10	64300005	T	A	0.7826	1.08 (1.05-1.12)	4.54E-06	2.42E-07	+--+++?+++	Secondary signals
rs79297937	rs10822013,rs10995201	10q21.2	10	64300024	A	C	0.2176	0.92 (0.89-0.96)	5.36E-06	2.81E-07	-++---?---	Secondary signals
rs78053936	rs10822013,rs10995201	10q21.2	10	64300331	C	A	0.8022	1.11 (1.07-1.15)	1.74E-08	2.74E-09	++-+++?+++	Secondary signals
rs10762849	rs704010	10q22.3	10	80886061	T	C	0.4372	1.07 (1.04-1.10)	2.39E-06	2.37E-28	+-++++?+++	Better signals
rs10762850	rs704010	10q22.3	10	80886337	G	T	0.5588	0.93 (0.91-0.96)	2.62E-06	7.67E-28	-+----?---	Better signals
rs10824721	rs704010	10q22.3	10	80886599	C	G	0.4477	1.07 (1.04-1.10)	2.35E-06	3.99E-28	+-++++?+++	Better signals
rs4980029	rs704010	10q22.3	10	80886726	G	A	0.5231	0.94 (0.91-0.96)	5.43E-06	5.54E-28	-+----?---	Better signals
rs4980030	rs704010	10q22.3	10	80886835	C	T	0.5796	0.93 (0.91-0.96)	2.75E-06	4.80E-26	-+----?---	Better signals
rs7903793	rs704010	10q22.3	10	80887212	T	C	0.4178	1.07 (1.04-1.10)	1.94E-06	1.32E-25	+-++++?+++	Better signals
rs7904249	rs704010	10q22.3	10	80887337	A	G	0.4177	1.07 (1.04-1.10)	1.30E-06	7.72E-26	+-++++?+++	Better signals
rs11269768	rs704010	10q22.3	10	80887430	G	GGCCCCTGCTCA	0.413	1.07 (1.03-1.10)	4.34E-05	5.60E-25	+-++++???+	Secondary signals
rs7921002	rs704010	10q22.3	10	80887721	G	T	0.5561	0.93 (0.91-0.96)	1.65E-06	1.14E-25	-+----?---	Better signals
rs10762851	rs704010	10q22.3	10	80887957	G	A	0.556	0.93 (0.91-0.96)	2.33E-06	9.54E-26	-+----?---	Better signals
10:80888588:A:AAAAAG	rs704010	10q22.3	10	80888588	AAAAAG	A	0.5597	0.94 (0.91-0.97)	2.79E-05	1.45E-25	-+----???-	Secondary signals
rs7097066	rs704010	10q22.3	10	80888858	A	G	0.4437	1.07 (1.04-1.10)	7.92E-07	2.42E-26	+-++++?+++	Better signals
rs75167266	rs704010	10q22.3	10	80889177	T	C	0.416	1.08 (1.05-1.11)	4.70E-07	7.59E-26	+-++++?+++	Better signals
rs74385409	rs704010	10q22.3	10	80889211	T	G	0.4148	1.07 (1.04-1.11)	6.80E-07	5.37E-26	+-++++?+++	Better signals
rs72816252	rs704010	10q22.3	10	80889806	G	A	0.5813	0.93 (0.90-0.96)	6.94E-07	5.09E-26	-+----?---	Better signals
rs4979844	rs704010	10q22.3	10	80890671	A	T	0.402	1.08 (1.05-1.11)	5.09E-07	3.67E-26	+-++++?+++	Better signals
rs4979845	rs704010	10q22.3	10	80890674	C	T	0.599	0.93 (0.90-0.96)	6.01E-07	1.84E-26	-+----?---	Better signals
rs4980031	rs704010	10q22.3	10	80891631	T	C	0.3982	1.07 (1.04-1.11)	9.27E-07	6.83E-26	+-++++?+++	Better signals
rs1338427	rs7904519	10q25.2	10	115126117	A	C	0.7564	1.08 (1.04-1.12)	1.24E-05	6.09E-18	--+?++++?+	Better signals
rs10885438	rs7904519	10q25.2	10	115126868	G	A	0.2439	0.93 (0.90-0.96)	7.68E-06	2.74E-18	++-?----?-	Better signals
rs112400465	rs7904519	10q25.2	10	115127454	CAA	C	0.2015	0.93 (0.9-0.96)	8.93E-05	8.93E-05	-+-?----??	Better signals
rs12250948	rs7904519	10q25.2	10	115128491	T	C	0.731	1.08 (1.05-1.12)	2.34E-06	9.81E-20	--+?++++?+	Better signals
rs10579013	rs7904519	10q25.2	10	115131314	CAT	C	0.7907	1.08 (1.04-1.12)	4.22E-05	3.12E-11	+-+?++++?+	Better signals
rs11196311	rs7904519	10q25.2	10	115132212	G	T	0.2093	0.93 (0.90-0.96)	4.43E-05	1.11E-11	-+-?----?-	Better signals
rs10787484	rs7904519	10q25.2	10	115137668	A	G	0.7889	1.08 (1.04-1.12)	3.97E-05	1.17E-11	+-+?++++?+	Better signals
rs10886955	rs11199914,rs2981578,rs45631563	10q26.13	10	123370354	A	C	0.2629	1.07 (1.04-1.11)	7.87E-05	4.24E-04	+-++++?+?+	Secondary signals
rs4980395	rs3817198	11p15.5	11	1876594	T	C	0.0886	1.16 (1.09-1.23)	4.20E-07	1.81E-05	+-++++?+++	Better signals
rs72843933	rs3817198	11p15.5	11	1878406	T	C	0.172	1.10 (1.06-1.14)	2.96E-06	3.54E-05	++++++?+++	Better signals
rs57775872	rs3817198	11p15.5	11	1882079	T	ATGGGTGGATGAGTC	0.5461	0.91 (0.88-0.95)	9.29E-07	9.29E-07	-------???	Better signals
rs72843938	rs3817198	11p15.5	11	1884353	A	G	0.0912	1.16 (1.10-1.23)	8.86E-08	1.99E-07	+-++++?+++	Better signals
rs1810199	rs3817198	11p15.5	11	1886306	T	C	0.2949	1.07 (1.03-1.10)	4.94E-05	7.57E-16	+-++++++++	Better signals
rs61868778	rs3817198	11p15.5	11	1891319	T	C	0.0946	1.13 (1.07-1.20)	2.28E-05	4.70E-14	+-++++++++	Better signals

rs111528762	rs3817198	11p15.5	11	1893624	T	C	0.0968	1.13 (1.07-1.19)	2.39E-05	1.53E-14	+-++++++++	Better signals
rs1985624	rs3817198	11p15.5	11	1896777	C	T	0.9033	0.89 (0.84-0.94)	5.17E-05	8.96E-15	-+--------	Better signals
rs621679	rs3817198	11p15.5	11	1902768	A	G	0.497	1.06 (1.03-1.09)	5.82E-05	5.41E-13	+++++++---	Better signals
rs661348	rs3817198	11p15.5	11	1905292	C	T	0.5009	0.94 (0.91-0.97)	2.84E-05	2.52E-07	--------++	Better signals
rs3781962	rs3817198	11p15.5	11	1907609	G	C	0.5004	0.94 (0.91-0.97)	5.61E-05	1.42E-09	--------++	Better signals
rs3781956	rs3817198	11p15.5	11	1945884	A	G	0.5046	1.06 (1.03-1.10)	6.76E-05	1.98E-09	+++++++-?-	Better signals
rs540710	rs3817198	11p15.5	11	1947575	A	C	0.5006	1.06 (1.03-1.09)	9.92E-05	2.80E-10	+++++++-?-	Better signals
rs10894076	rs11820646	11q24.3	11	129463060	C	T	0.405	1.07 (1.03-1.10)	4.71E-05	4.71E-05	+-+++++++?	Better signals
rs12285545	rs11820646	11q24.3	11	129464443	A	G	0.5968	0.94 (0.91-0.97)	3.70E-05	1.64E-01	-+--------	Better signals
rs10791028	rs11820646	11q24.3	11	129467292	T	C	0.3908	1.07 (1.03-1.10)	3.20E-05	4.19E-02	+-++++++++	Better signals
rs11067617	rs1292011	12q24.21	12	115926630	A	G	0.4496	1.06 (1.03-1.09)	5.18E-05	3.75E-07	++++++?+++	Secondary signals
rs9788133	rs1292011	12q24.21	12	115979953	A	G	0.1685	1.08 (1.04-1.12)	4.27E-05	2.41E-10	+--+++?+++	Secondary signals
rs2434079	rs1292011	12q24.21	12	115989277	C	T	0.8333	0.92 (0.89-0.96)	4.46E-05	5.16E-10	-++---?---	Secondary signals
rs61929345	rs1292011	12q24.21	12	116001403	T	G	0.1608	1.11 (1.07-1.15)	4.90E-08	2.87E-04	++-+++++++	Secondary signals
rs11067657	rs1292011	12q24.21	12	116002390	G	T	0.8334	0.91 (0.88-0.94)	2.26E-07	1.17E-03	--+-------	Secondary signals
rs75041341	rs1292011	12q24.21	12	116007174	G	T	0.8347	0.91 (0.88-0.94)	4.00E-07	1.27E-03	--+-------	Secondary signals
rs75525437	rs1292011	12q24.21	12	116007358	A	G	0.1564	1.10 (1.06-1.15)	2.37E-07	5.78E-04	++-+++++++	Secondary signals
rs115377147	rs1292011	12q24.21	12	116008014	C	A	0.8374	0.91 (0.87-0.94)	1.94E-07	7.26E-04	--+-------	Secondary signals
rs181046969	rs1292011	12q24.21	12	116008018	A	C	0.1584	1.10 (1.06-1.15)	2.17E-07	7.48E-04	++-+++++++	Secondary signals
rs35327801	rs4784227	16q12.1	16	52545751	GA	G	0.828	1.08 (1.04-1.12)	7.98E-05	4.05E-03	+++-++++?+	Secondary signals
rs7193453	rs4784227	16q12.1	16	52565150	T	C	0.5055	1.07 (1.04-1.10)	1.31E-05	4.58E-05	++++++++++	Secondary signals
rs9926163	rs4784227	16q12.1	16	52565641	T	G	0.5051	1.07 (1.04-1.10)	1.30E-05	4.60E-05	++++++++++	Secondary signals
rs4783780	rs4784227	16q12.1	16	52571436	C	A	0.4784	0.93 (0.90-0.96)	1.06E-06	2.37E-05	----------	Secondary signals
rs9931232	rs4784227	16q12.1	16	52572832	A	G	0.527	1.08 (1.04-1.11)	1.44E-06	2.69E-05	++++++++++	Secondary signals
rs8052175	rs4784227	16q12.1	16	52577142	T	C	0.5225	1.07 (1.04-1.11)	2.12E-06	5.91E-06	++++++++++	Secondary signals
rs1123428	rs4784227	16q12.1	16	52577835	A	T	0.5227	1.07 (1.04-1.11)	2.20E-06	6.54E-06	++++++++++	Secondary signals
rs1420535	rs4784227	16q12.1	16	52578215	A	C	0.5224	1.07 (1.04-1.11)	2.14E-06	6.47E-06	++++++++++	Secondary signals
rs1477074	rs4784227	16q12.1	16	52578548	C	T	0.4773	0.93 (0.90-0.96)	2.20E-06	5.51E-06	----------	Secondary signals
rs8044585	rs4784227	16q12.1	16	52579906	A	C	0.5198	1.07 (1.04-1.11)	2.90E-06	2.12E-05	+++++++-++	Secondary signals
rs4784224	rs4784227	16q12.1	16	52580247	A	C	0.5222	1.07 (1.04-1.10)	9.30E-06	3.32E-05	++++++++++	Secondary signals
16:52582774:TAA:TA	rs4784227	16q12.1	16	52582774	TA	TAA	0.4692	0.93 (0.9-0.96)	3.30E-05	3.30E-05	-------???	Secondary signals
rs3803662	rs4784227	16q12.1	16	52586341	A	G	0.6279	1.08 (1.05-1.12)	4.13E-08	9.80E-14	++++++++++	Secondary signals
rs3803661	rs4784227	16q12.1	16	52586477	A	G	0.6278	1.08 (1.05-1.12)	3.69E-08	4.98E-14	++++++++++	Secondary signals
rs1109951	rs4784227	16q12.1	16	52612044	T	C	0.1684	0.93 (0.89-0.96)	9.40E-05	3.68E-03	---+----?-	Secondary signals
rs57277860	rs527616,rs1436904	18q11.2	18	24531525	G	GTT	0.3823	1.07 (1.04-1.11)	1.34E-06	1.34E-06	+-++--?+??	Better signals
rs1551821	rs6507583	18q12.3	18	42866470	C	A	0.7388	1.08 (1.04-1.11)	3.60E-06	1.30E-07	++++++?+++	Better signals
rs11662289	rs6507583	18q12.3	18	42868375	G	A	0.7105	1.07 (1.04-1.11)	2.34E-06	3.69E-08	++++++?+++	Better signals
rs11659252	rs6507583	18q12.3	18	42877575	G	A	0.7398	1.08 (1.05-1.12)	1.80E-07	2.66E-15	++++++?+++	Better signals
rs35451159	rs6507583	18q12.3	18	42877630	GA	G	0.7412	1.08 (1.04-1.11)	4.14E-06	1.31E-14	++++++?+?+	Better signals
rs7233577	rs6507583	18q12.3	18	42878504	C	T	0.7371	1.08 (1.05-1.12)	4.13E-07	4.30E-15	++++++?+++	Better signals
rs8093563	rs6507583	18q12.3	18	42879717	A	G	0.1473	0.9 (0.87-0.94)	2.27E-07	2.65E-12	------?---	Better signals
rs12456097	rs6507583	18q12.3	18	42880242	G	A	0.7114	1.09 (1.06-1.12)	3.10E-08	3.22E-17	++++++?+++	Better signals
rs112901920	rs6507583	18q12.3	18	42880349	CTCTG	C	0.2603	0.93 (0.9-0.96)	3.30E-05	7.73E-14	------???-	Better signals
rs9963678	rs6507583	18q12.3	18	42881314	T	C	0.1707	0.91 (0.88-0.94)	4.66E-07	9.32E-13	------?---	Better signals
rs12455117	rs6507583	18q12.3	18	42884026	T	A	0.6845	1.09 (1.06-1.12)	1.71E-08	3.16E-17	++++++?+++	Better signals
rs36043071	rs6507583	18q12.3	18	42888149	C	T	0.7467	1.06 (1.03-1.1)	8.42E-05	2.13E-07	++++++?+++	Better signals
rs9952980	rs6507583	18q12.3	18	42888797	C	T	0.6649	1.08 (1.05-1.11)	2.66E-07	1.22E-17	++++++?+++	Better signals
rs11082442	rs6507583	18q12.3	18	42889017	G	C	0.6708	1.07 (1.04-1.11)	8.04E-07	1.36E-15	++++++?+++	Better signals
rs12458939	rs6507583	18q12.3	18	42889835	A	G	0.2533	0.94 (0.91-0.97)	6.33E-05	1.09E-07	------?---	Better signals
rs12954053	rs6507583	18q12.3	18	42891853	A	G	0.227	0.93 (0.9-0.96)	3.74E-06	5.54E-08	------?---	Better signals
rs16978341	rs6507583	18q12.3	18	42891918	C	A	0.7725	1.08 (1.05-1.12)	3.68E-06	5.51E-08	++++++?+++	Better signals

rs8086981	rs6507583	18q12.3	18	42892099	T	C	0.2269	0.93 (0.9-0.96)	3.42E-06	5.59E-08	------?---	Better signals
rs8086861	rs6507583	18q12.3	18	42892127	G	A	0.7733	1.08 (1.04-1.12)	3.93E-06	6.52E-08	++++++?+++	Better signals
rs11314315	rs6507583	18q12.3	18	42892348	G	GA	0.2458	0.93 (0.9-0.96)	1.22E-05	2.58E-08	------?-?-	Better signals
rs2823095	rs2823093	21q21.1	21	16521269	A	G	0.2141	0.91 (0.88-0.94)	2.57E-08	7.30E-08	------?---	Better signals
rs2823120	rs2823093	21q21.1	21	16547722	A	G	0.1927	0.92 (0.89-0.95)	2.49E-06	5.88E-08	------?---	Better signals
rs7275186	rs2823093	21q21.1	21	16560348	C	T	0.6553	1.09 (1.06-1.12)	2.81E-09	5.13E-04	++++++++++	Better signals
rs2823126	rs2823093	21q21.1	21	16561704	A	G	0.2805	0.9 (0.88-0.93)	1.10E-10	1.17E-10	------?---	Better signals
rs73894936	rs2823093	21q21.1	21	16576566	A	G	0.3444	0.93 (0.9-0.95)	1.95E-07	7.63E-04	------?---	Better signals
rs2823148	rs2823093	21q21.1	21	16584992	A	G	0.4099	1.06 (1.03-1.09)	6.17E-05	4.54E-04	+-++++++++	Better signals
rs2823149	rs2823093	21q21.1	21	16585201	T	C	0.4086	1.06 (1.03-1.09)	6.04E-05	4.49E-04	+-++++++++	Better signals

Secondary signals: SNPs had $P < 0.0001$ in the conditional analysis but the magnitude was not 10 times smaller than all of the previously reported risk SNPs nearby.

Better signals: SNPs had $P < 0.0001$ in the conditional analysis and the magnitude was 10 times smaller than any of the previously reported risk SNPs nearby.
