# Supplementary material for: Identification of novel breast cancer susceptibility loci in meta-analyses conducted among Asian and European descendants
Source: Nat Commun. 2020 Mar 5;11:1217. doi: 10.1038/s41467-020-15046-w (PMC7057957; doi:10.1038/s41467-020-15046-w)
Supplement: Supplementary file 10 — Reporting Summary [file 41467_2020_15046_MOESM10_ESM.pdf]

nature research

Corresponding author(s): Wei Zheng

Last updated by author(s): 01-21-2020

Reporting Summary

Nature Research wishes to improve the reproducibility of the work that we publish. This form provides structure for consistency and transparency in reporting. For further information on Nature Research policies, see Authors & Referees and the Editorial Policy Checklist.

Statistics

For all statistical analyses, confirm that the following items are present in the figure legend, table legend, main text, or Methods section.

n/a	Confirmed	
☐	☒	The exact sample size (n) for each experimental group/condition, given as a discrete number and unit of measurement
☐	☒	A statement on whether measurements were taken from distinct samples or whether the same sample was measured repeatedly
☐	☒	The statistical test(s) used AND whether they are one- or two-sided<br>*Only common tests should be described solely by name; describe more complex techniques in the Methods section.*
☐	☒	A description of all covariates tested
☐	☒	A description of any assumptions or corrections, such as tests of normality and adjustment for multiple comparisons
☐	☒	A full description of the statistical parameters including central tendency (e.g. means) or other basic estimates (e.g. regression coefficient) AND variation (e.g. standard deviation) or associated estimates of uncertainty (e.g. confidence intervals)
☐	☒	For null hypothesis testing, the test statistic (e.g. F, t, r) with confidence intervals, effect sizes, degrees of freedom and P value noted<br>*Give P values as exact values whenever suitable.*
☒	☐	For Bayesian analysis, information on the choice of priors and Markov chain Monte Carlo settings
☒	☐	For hierarchical and complex designs, identification of the appropriate level for tests and full reporting of outcomes
☐	☒	Estimates of effect sizes (e.g. Cohen's d, Pearson's r), indicating how they were calculated

Our web collection on statistics for biologists contains articles on many of the points above.

Software and code

Policy information about availability of computer code

Data collection	NA
Data analysis	PLINK 2.0, https://www.cog-genomics.org/plink/2.0/<br>METAL, https://angus.readthedocs.io/en/2017/meta_GWAS.html<br>MR-MEGA, https://genomics.ut.ee/en/tools/mr-mega<br>GCTA, http://cnsgenomics.com/software/gcta/#Overview<br>GARFIELD, https://www.ebi.ac.uk/birney-srv/GARFIELD/<br>MetaXcan, https://github.com/hakyimlab/MetaXcan<br>R 3.6.0, https://www.r-project.org/

For manuscripts utilizing custom algorithms or software that are central to the research but not yet described in published literature, software must be made available to editors/reviewers. We strongly encourage code deposition in a community repository (e.g. GitHub). See the Nature Research guidelines for submitting code & software for further information.

Data

Policy information about availability of data

All manuscripts must include a data availability statement. This statement should provide the following information, where applicable:

- Accession codes, unique identifiers, or web links for publicly available datasets
- A list of figures that have associated raw data
- A description of any restrictions on data availability

Access to the ABCC data could be requested by submission of data sharing inquiry to Dr. Wei Zheng (wei.zheng@vanderbilt.edu). Request of access to the BCAC data could be submitted directly to BCAC (http://bcac.ccge.medschl.cam.ac.uk/). Access to other data: GTEx: https://gtexportal.org/home/datasets; TCGA - https://portal.gdc.cancer.gov/; METABRIC: https://www.ebi.ac.uk/ega/studies/EGAS00000000083.

Field-specific reporting

Please select the one below that is the best fit for your research. If you are not sure, read the appropriate sections before making your selection.

☒ Life sciences ☐ Behavioural & social sciences ☐ Ecological, evolutionary & environmental sciences

For a reference copy of the document with all sections, see nature.com/documents/nr-reporting-summary-flat.pdf

Life sciences study design

All studies must disclose on these points even when the disclosure is negative.

Sample size	No predetermination of sample size was performed for the current study. A ad-hoc calculation of statistical power was conducted. For the cross-ancestry meta-analysis (alpha set to 5.0×10-8), we had > 80% power to detect the association between SNP and breast cancer risk with an OR of > 1.06, 1.07, and 1.11 and EAF of 0.10 in the analysis of ER-positive, ER-negative cancer and all cancer combined, respectively.
Data exclusions	Samples were excluded if they (i) had genotyping call rate < 95%; (ii) were male based on genotype data; (ii) had a close relationship with a Pi-HAT estimate > 0.25; (iii) were heterozygosity outliers; (iv) were ancestry outliers. SNPs were excluded if they had (i) a call rate < 95%; (ii) no clear genotyping clusters; (iii) a minor allele frequency < 0.001; (iv) a Hardy-Weinberg equilibrium test of P < 1 ×10-6; (v) genotyping concordance < 95% among the duplicated QC samples.
Replication	Genotyping of the replication set of cases and controls was completed using the iPLEX Sequenom MassArray platform (Agena Bioscience Inc., San Diego, California, USA). One negative control (water), two blinded duplicates and two samples from the HapMap project were included as QC samples in each 96-well plate. Samples or SNPs that had a genotyping call rate of<95% were excluded. We also excluded SNPs that had a concordance with the QC samples of<95% or an unclear genotype call. If the assay could not be designed for the lead SNP, a surrogate SNP which is in LD with the lead SNP with r2 > 0.8 in Asians (1000 Genome) was selected.
Randomization	This is an observational study thus no randomization was performed.
Blinding	This is an observational study thus blinding is not applicable.

Reporting for specific materials, systems and methods

We require information from authors about some types of materials, experimental systems and methods used in many studies. Here, indicate whether each material, system or method listed is relevant to your study. If you are not sure if a list item applies to your research, read the appropriate section before selecting a response.

Materials & experimental systems

n/a	Involved in the study	
☒	☐	Antibodies
☒	☐	Eukaryotic cell lines
☒	☐	Palaeontology
☒	☐	Animals and other organisms
☐	☒	Human research participants
☒	☐	Clinical data

Methods

n/a	Involved in the study	
☒	☐	ChIP-seq
☒	☐	Flow cytometry
☒	☐	MRI-based neuroimaging

Human research participants

Policy information about studies involving human research participants

Population characteristics	The information regarding population characteristics can be found in Supplementary Text S1.
Recruitment	The information regarding recruitment can be found in Supplementary Text S1.
Ethics oversight	The information regarding ethics oversight can be found in Supplementary Text S1.

Note that full information on the approval of the study protocol must also be provided in the manuscript.
